# The effect of food taxes in the UK on health and economic outcomes, and equity: a modelling study

**DOI:** 10.1016/S2468-2667(26)00196-9

**Published:** 2026-10-20

**Authors:** Katharine Pidd, Luke B Wilson, Robert Pryce, Alan Brennan, Damon Morris, Christian Reynolds, Amelia A Lake, Helen J Moore, Andrea Burrows, Rebecca Wells, Christina Vogel, Natalie Connor, Penny Breeze

**Affiliations:** a Sheffield Centre for Health and Related Research, University of Sheffield, Sheffield, UK; b City St George's, University of London, London, UK; c School of Health and Life Sciences, Teesside, UK; d School of Social Sciences, Humanities and Law, Teesside, UK; e Teesside University, Teesside, UK; f Fuse, The Centre for Translational Research in Public Health, Newcastle, UK

## Abstract

**Background:**

WHO recommends using fiscal policies (such as taxes on unhealthy foods) that discourage consuming foods that contribute to unhealthy diets. Food tax evaluations require consideration for sociodemographic differences, consumer and industry behaviour, reformulation, and long-term health-related outcomes and health inequalities. We aimed to model the potential benefits and harms of three food tax policy designs, to health, costs, and inequalities, in the UK.

**Methods:**

Using a microsimulation model, we performed an economic evaluation of three UK food tax policy designs: (1) a Sugar Snacks tax targeting snacks, cakes, and milk drinks; (2) an Unhealthy Food tax high in fat, sugar, or salt; and (3) a Sugar and Salt tax. Consumption and nutritional changes for a UK representative synthetic population (aged ≥5 years) were estimated by applying a hierarchical Almost Ideal Demand System, accommodating key socio-demographic differences. Our primary analysis simulated outcomes for consumer response only. Secondary analyses incorporated industry responses in the model. The microsimulation estimated changes to weight, cardiometabolic risk factors, lifetime health, discounted National Health Service (NHS) costs, social care costs, unemployment or inactivity, and social benefit receipts.

**Findings:**

The estimated average reduction in daily calorie intake was –25 kcal (95% credible interval [95% CrI]; –31 to –19), –42 kcal (–51 to –33), and –58 kcal (–66 to –50) for the Sugar Snacks, Unhealthy Food, and Sugar and Salt taxes respectively, with reductions in sugar, salt, and saturated fat. Long-term modelling showed improved health, including reduced cardiovascular disease, and type 2 diabetes for all three tax policies. The taxes reduced NHS costs (Sugar Snacks –£168 [95% CrI –461 to –73], Unhealthy Food –£268 [–722 to –105], and Sugar and Salt –£374 [–1033 to –159]) and social care costs. The impact on health inequalities varied by policy design. Only the Sugar and Salt tax had a positive impact on health inequalities.

**Interpretation:**

The overall health impact of each of the three tax policy designs modelled was positive, but it can be associated with important nutritional trade-off—cross-price elasticities led to a reduction in fibre intake. Of the three policies modelled, the Sugar and Salt tax generated the greatest gains and reduced health inequalities. Policy design should consider potential impacts on health inequalities and should be part of a multi-component strategy.

**Funding:**

National Institute for Health and Care Research.

## Introduction

Globally, obesity has more than doubled in adults and quadrupled in children between 1990 and 2022, in part caused by environmental factors impacting purchases of healthy foods.^1^ In 2022, 2·5 billion adults and 390 million children aged 5–19 years were living with overweight and obesity, and the associated societal costs are substantial.^1^ Diet and nutrition are key drivers of obesity. In the UK, it is estimated that adults consume an excess of 200–300 calories per day and over 80% of the population consume more than the recommended amounts of saturated fat and sugar.^2^, ^3^

Health tax policies that target food and drinks have been implemented in numerous countries, reflecting a global effort to shift consumption away from less healthy options. A national tax on sugar-sweetened beverages has been applied in 119 countries by 2023.^4^ There is evidence that taxes on sugar-sweetened beverages reduce both purchasing and sugar consumption.^5^ Mexico, Hungary, and Colombia have all implemented food taxes.^6^, ^7^, ^8^ Reductions in sales of the taxed foods have been observed in many contexts, alongside reformulation by industry towards healthier products.^5^ However, unintended consequences have been identified, such as substitution towards non-taxed unhealthy foods or away from healthy foods, greater food spending burden, worsened inequalities, with initial gains possibly diminishing as industry and consumers respond over time.^5^, ^6^, ^7^

Real-world evidence for the effectiveness of food taxes is promising but might not be generalisable due to contextual complexities in the food systems, and cannot readily be used to compare alternative policy design specifications. Modelling studies can be used to compare food tax policy designs by simulating expected consumption change and health outcomes.^9^, ^10^, ^11^, ^12^ Previous modelling studies of taxes targeting nutritional components or unhealthy foods have estimated improvements to diet and health.^9^, ^10^, ^11^, ^12^, ^13^, ^14^, ^15^

**Research in context**

**Evidence before this study**

We searched PubMed, Health Management Information Consortium (HMIC), Scopus, Google, Mintel, and Business Source Premier Ultimate for peer-reviewed and grey literature published between Jan 1, 2010, and Dec 31, 2022. We used search terms related to fiscal policies (“tax”, “price”, “levy”, “fiscal”) applied to specific dietary categories (“food and beverages”, “take-away”, “junk food”, “fast food”, “sweet”, “confectionery”, “snack”, “sodium”, “salt”), excluding documents not written in the English language. Of the 1696 records screened, 67 studies in high-income countries were included (53 peer-reviewed; 14 grey literature), covering food taxes on high fat, salt, and sugar (30 studies), sugar-sweetened beverages (54 studies), and meat (two studies). The evidence consistently indicated that food taxes reduce consumption of targeted products and improve population health outcomes. Evidence also highlighted potential unintended consequences, including substitution towards non-targeted unhealthy nutrients and the regressive financial impact on lower-income households. We updated the search in PubMed, HMIC, and Scopus on June 10, 2026, and found 32 additional peer-reviewed original research studies with a similar distribution of studies examining tax options on high fat, salt, and sugar (n=11), sugar-sweetened beverages (n=24), and meat (n=1), with empirical evidence for the impact of taxes on marketing (n=2), reformulation (n=3), and passthrough (n=3). In total, seven simulation studies compared the effects of alternative tax designs. Only three studies included both a detailed cross-price elasticity framework across all food and drinks and simulated industry behaviours effects, and four simulated differences in baseline consumption and price elasticity of demand by social groups.

**Added value of this study**

This study introduces a novel approach to evaluate multiple tax policies in an equity informed model by simulating how sociodemographic groups experience such approach. Our study maps these variations from initial exposure (prices and consumption) through key mechanisms (price elasticity, reformulation, and tax pass-through), to long-term health outcomes (cardiometabolic trajectories and risk equations). By incorporating differential tax pass-through, product reformulation, and price elasticity of demand, the model captures competing dynamics that are otherwise difficult to predict and combines them with inequalities in health complications risks, which provides granular representation of socioeconomic factors, moving beyond stratification by baseline consumption and price elasticities alone to estimate health, economic, and health equity impacts. Our analysis highlights an important nutritional trade-off: cross-price elasticities led to a reduction in fibre intake, but the integrated evaluation modelling clarifies that the overall health impact of each of the three specific tax policy designs examined was positive. Of the three policies examined, the Sugar and Salt tax was estimated to generate the greatest health gains and the largest health and social care cost-savings, and was the only design to reduce health inequalities. By accounting for tax revenue, individual expenditure, and implications of health on labour market activity, this analysis contributes to the evidence base for a wide range of stakeholders across government, industry, and society, beyond direct healthcare savings.

**Implications of all the available evidence**

The combined evidence suggests that taxes targeting specific foods and nutrients can be an effective lever in tackling rising health consequences of obesity and unbalanced diets. However, the policy design needs to reflect the context of consumer demand, industry dynamics, population characteristics, and health within which it is intended to be implemented and the objectives of the tax. To generate larger changes to diet, policy makers should favour taxes with broad eligibility criteria that encourage reformulation to avoid consumption swapping to other non-taxed unhealthy foods. Given that policy variations impact population groups differently, fiscal measures should be part of a multi-component strategy, which includes pairing taxes with complementary subsidies or social programmes to offset potential financial burdens and reductions in fibre intake, particularly in low-income households.

The effectiveness of food taxes hinges on understanding not just the consumer response but also considering industry responses, unintended consequences, and the differential impact across population groups.^16^

We aimed to quantitatively evaluate the benefits and harms of three food tax policy designs within the UK across multi-sectoral health, health and social care costs, individual expenditure, tax revenue, and inequality. These tax policy designs were co-produced with stakeholders based on public acceptability, feasibility, and effectiveness. The designs varied by eligibility criteria and whether they applied a flat or variable tax based on nutrient content, which were: (1) threshold-based tax on sugar in snacks, cakes, and milk drinks; (2) flat rate tax on foods high in fat, salt, or sugar; and (3) tax per gram of sugar and salt in selected foods. The analysis plan compares policy designs to support decisions, testing sensitivity to key assumptions.

## Methods

### Model overview

Microsimulation modelling estimated consumption, expenditure, and nutritional intake, considering income and substitution effects driven by taxation. The term ‘food’ is hereafter used to represent food and non-alcoholic beverages. The analysis adopted a government perspective (ie, tax revenue, health outcomes, UK National Health Service [NHS] costs, social care costs, employment, and social benefit receipts) with additional outcomes for individual food spending and informal care costs, reflecting the prices, UK population, and costs for 2022. The model accommodated key system features, including industry responses, sociodemographic differences, and unintended consequences that affect the value of food taxation to decision makers.

Our primary analysis simulated outcomes for consumer response only. Secondary analyses incorporated industry responses. Additional sensitivity analyses explore the uncertainties and assumptions on findings.

### Tax policy designs

Three tax policies were formulated in 2022–23 following a rapid review, media analysis, public and stakeholder workshops, and interviews. Their design considered stakeholder perspectives on acceptability, feasibility, and effectiveness.^17^, ^18^ A diverse Patient and Public Involvement panel was consulted throughout the project, actively shaping the research design and interpreting findings.

The first tax policy is the Sugar Snacks tax for biscuits, cakes, and confectionery with a sugar content exceeding 18 g per 100 g of product taxed at a rate of £4/kg of sugar. Milk drinks with added sugar were taxed according to the existing Soft Drinks Industry Levy.^19^ The policy included food purchased in supermarkets and out-of-home establishments.

The second tax policy is the Unhealthy Food tax for retail foods high in fat, sugar, and/or salt including ready meals, snacks, breakfast cereals, desserts, cakes and confectionery, and milk drinks. Inclusion was conditional on nutrient profile score, which indicates the overall healthiness of foods by balancing their healthy and unhealthy components.^20^ Foods were included if they scored at least 4, and drinks if they scored at least 1. All included foods are taxed at 20% of their price.

The final tax policy is the Sugar and Salt tax, in which manufactured foods and prepared out-of-home products included in the UK Government's restrictions on the advertising and promotion of foods high in fat, salt, and sugar, and in the voluntary reformulation programme campaigns (calorie, salt, and sugar reduction), were taxed at £3/kg of added sugar and £6/kg of salt. This policy was adapted from the National Food Strategy's recommendations.^21^ Detailed policy descriptions are defined in the appendix (pp 30–34).

### Consumer responses

Consumer responses to changes in prices were simulated using a two-stage hierarchical almost ideal demand system assuming uncompensated demand. Own and cross-price elasticities of demand for the first stage higher-level food categories and the second stage lower-level food groups were estimated using data from the Living Costs and Food Survey.

Food expenditure was not assumed to be constant, but the demand system accounts for a reduction in real purchasing power from price increases. Complete price elasticities of demand matrices are provided in the appendix (pp 59–69).

### Modelling

Figure 1 describes the modelling processes used to estimate policy impacts. A synthetic population of 2 million individuals aged 5–99 years was generated using the Health Survey for England and census data, weighted by age, sex, and sociodemographic distribution.^22^, ^23^ Food consumption across 50 groups was assigned to the population using National Diet and Nutrition Survey data (2008–19) based on age, sex, and socioeconomic classification.^24^ Each demographic group received its average consumption pattern. Consumption of food groups were split into grams that are taxed and not taxed using regression analyses of the proportion of daily consumption taxed for each tax design by demographic group using National Diet and Nutrition Survey raw food diary data (2016–19).

**Figure 1:**
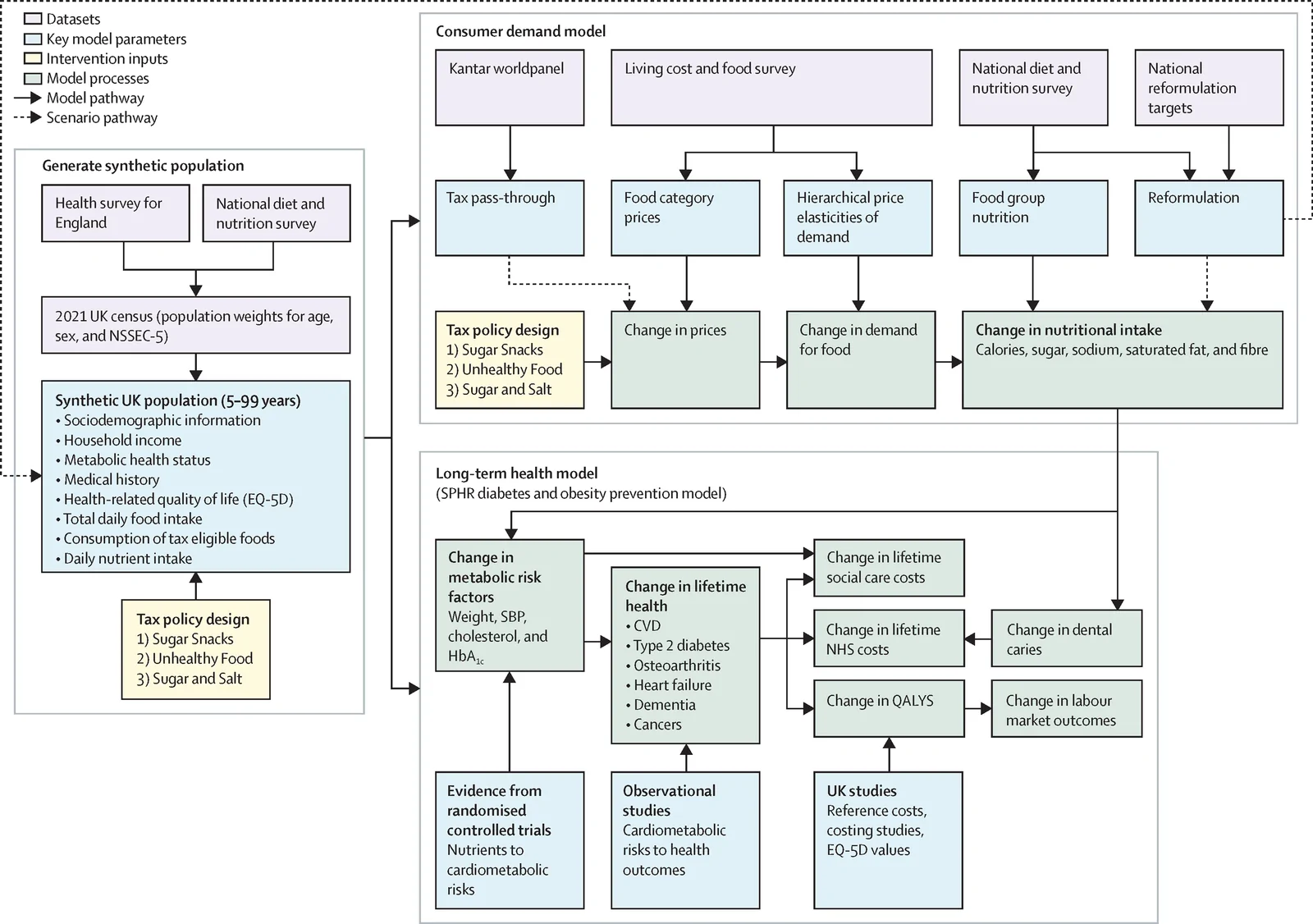
A model schematic presenting the pathways through which tax policies impact diet, health, economic, and equity outcomes EQ-5D=EuroQol-5D. CVD=cardiovascular disease. HBA_1c_=glycated haemoglobin A_1c_. NSSEC-5=National Statistics Socioeconomic classification, 5 class version. QALYs=quality-adjusted life-years. SPHR=School for Public Health Research. SBP=systolic blood pressure.

Food prices were estimated for each lower-level food group by equivalised income tertile using expenditure and consumption from Family Food datasets to reflect socioeconomic variation in price paid.^25^ The changes in prices were calculated for each policy design. Consumer responses to price changes were simulated using the price elasticities of demand matrices by estimating new expenditure allocation across 12 higher-level food categories given the weighted average percentage change in price of the food groups within that category, then estimating how this spending and consumption (g/day) was allocated between lower-level food groups within each category using individual price changes.

The change in calories, sugars, sodium, fats, carbohydrates, and fibre was estimated from changes in food consumption using the weighted mean nutritional content of each food group, which was estimated from the National Diet and Nutrition Survey data, separately for taxed and not taxed food. The resulting changes to cardiometabolic trajectories were estimated based on calorie-to-weight models for adults and children, and an umbrella review of systematic reviews for nutrient impacts on systolic blood pressure, cholesterol, and glycated haemoglobin A_1c_.^26^, ^27^, ^28^

Change in consumption was assumed permanent, and results in lifetime changes to metabolic trajectories. The lifetime impact on health outcomes, the NHS, and wider societal costs was simulated using an existing microsimulation model.^29^ The model predicted health outcomes were type 2 diabetes and its complications, cardiovascular disease, cancers, osteoarthritis, and dementia annually for adults conditional on their weight and cardiometabolic risk factors; mortality was estimated conditional on UK lifetables and health outcomes, and dental health was conditional on sugar intake. Additional labour market indicators including inactivity or unemployment and social benefit receipts were estimated conditional on individuals’ health-related quality of life (EQ-5D). Social care costs, both formal and informal costs for caring by family and friends, were estimated in older adults (aged ≥65 years) conditional on age and BMI. Lifetime quality-adjusted life-years (QALYs) were determined by health outcomes, age, and BMI, and NHS costs were dependent on health and ongoing dental costs (appendix pp 35–150).

All costs and QALYs were discounted at 3·5% and were 0% in a sensitivity analysis.^30^ Lifetime costs and QALYs associated with the three tax policy designs were compared with a no-tax scenario assuming the current context of consumption, and metabolic trajectories remained unchanged and existing policies are unaffected by new taxes.

### Industry response

In the additional scenario analyses, industry responses to food taxes altered the food group nutrients (reformulation) and prices (tax pass-through). Maximum reformulation by food group was estimated based on the government's voluntary reformulation programme targets.^31^ Reformulation scenarios were applied in the model after subtracting reformulation already achieved, and capping reformulation at 5% and 10% for food groups identified with low and very low reformulation potential. For the Sugar and Salt tax, all included food groups reduced their calories, sugar, and salt content according to government reformulation targets. For the Sugar Snacks and Unhealthy Food designs, reformulation was only simulated if doing so saved the manufacturer incurring the tax (appendix p 80). Reformulation also impacted the proportion of each food group affected by the tax. Industry pass-through was assumed conditional on price with less tax-pass through for cheaper foods, but more for more expensive products. Prices varied by income group generating variation in pass-through rates. We modelled three scenarios that accounted for: tax pass-through (I1); reformulation (I2); and combined reformulation and tax pass-through effects (I3; appendix pp 79–82).

### Sensitivity analyses

The base-case results employed fixed, single-point, mean estimates for all model parameters. Additional analyses were performed, changing model structures, data inputs, or assumption of the model. The price elasticities of demand matrices were altered in five ways: (1) additional controls for non-food expenditure were included to account for flexibility to balance food and non-food spending (S1); (2) non-statistically significant elasticities were set to 0 (S2); (3) no reallocation of spending proportions between lower-level foods groups was assumed (S3); (4) the national average higher-level price elasticities of demand for the whole population was used rather than income-tertile specific price elasticities of demand matrices (S4); and (5) substitution was removed by setting cross-price elasticities to zero (S5). Baseline consumption was altered in two further sensitivity analyses: the population was assigned, individualised consumption patterns based on broader demographic and health characteristics using a two-step imputation and nearest neighbour matching process (S6), and finally, under-reporting was accounted for by inflating consumption across all food groups such that total calories consumed was adjusted (S7). Each alteration and 27 combinations of these seven alterations was analysed. Finally, an alternative price elasticities of demand matrix, estimated previously using different UK purchasing data, was applied (S8).^32^ Full descriptions of the alterations made are in the appendix (pp 83–91).

An exploratory probabilistic sensitivity analysis was performed on the base-case analysis, in which parameter inputs were varied across 400 samples for a sub-population of 100 000 simulated individuals (appendix p 151). Credible intervals from the probabilistic sensitivity analysis are presented in figure 2 and table 1. These intervals are not centred on the deterministic base-case estimates and reflect parameter uncertainty only.

**Figure 2:**
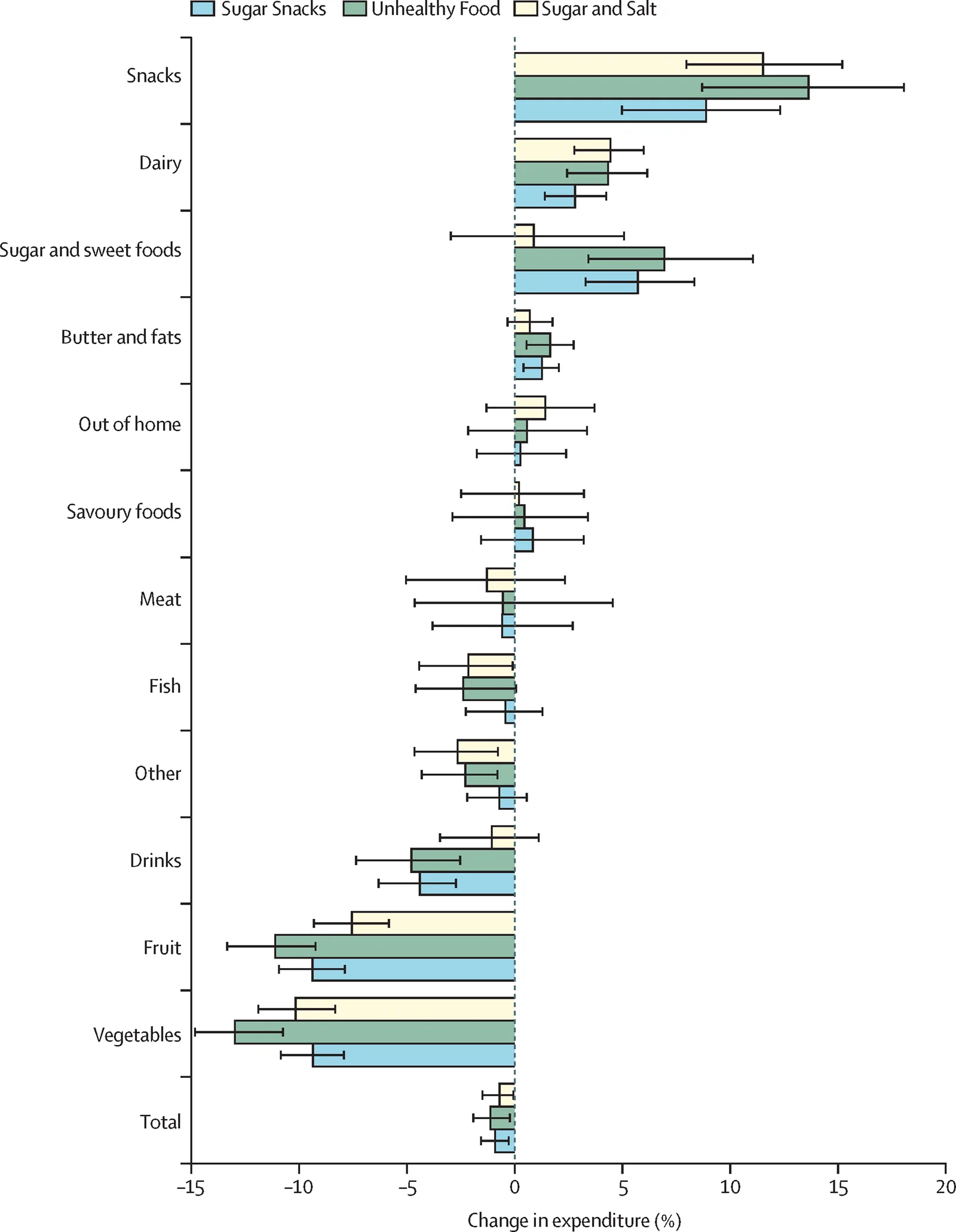
Average percentage change in expenditure by food categories for the three food tax policy designs Results are from the primary analysis, which included consumer responses only. Error bars represent 95% credible intervals from the exploratory probabilistic sensitivity analysis. All data, including absolute expenditure changes, are presented in the appendix (p 14).

**Table 1:** Mean daily change in calories, sugar, and salt, and incremental lifetime outcomes per person under the three food tax policies with and without industry response, overall and by Index of Multiple Deprivation quintiles

	**Primary analysis assuming no industry response**						**Secondary analysis incorporating reformulation and tax pass-through**					
	All	IMD 1 (most deprived)	IMD 2	IMD 3	IMD 4	IMD 5	All	IMD 1 (most deprived)	IMD 2	IMD 3	IMD 4	IMD 5
**Sugar Snacks**												
Calories (kcal)	−25	−21	−23	−25	−26	−28	−26	−21	−24	−27	−28	−30
Sugar (g)	−2·7	−2·7	−2·8	−2·7	−2·7	−2·7	−4·4	−4·4	−4·4	−4·4	−4·5	−4·5
Sodium (mg)	−12·9	−7	−10·7	−14	−15·8	−18·8	−16	−9·1	−13·4	−17·4	−19·4	−23·1
Saturated fat (g)	−0·2	−0·1	−0·2	−0·2	−0·2	−0·2	−0·2	−0·1	−0·2	−0·2	−0·2	−0·3
Fibre (g)	−0·7	−0·7	−0·7	−0·7	−0·7	−0·7	−0·7	−0·7	−0·7	−0·7	−0·8	−0·8
Incremental QALYs	0·033	0·03	0·032	0·035	0·033	0·033	0·034	0·03	0·033	0·037	0·035	0·036
Incremental NHS costs	−168	−182	−178	−166	−155	−151	−189	−198	−198	−189	−179	−175
**Unhealthy Food**												
Calories (kcal)	−42	−37	−41	−43	−45	−47	−44	−38	−42	−46	−48	−51
Sugar (g)	−3·1	−2·9	−3	−3·1	−3·2	−3·3	−4·9	−4·5	−4·8	−5	−5·1	−5·3
Sodium (mg)	−37·5	−31·7	−35·4	−38·6	−40·5	−43·2	−50·2	−43·2	−47·5	−51·6	−53·8	−57·5
Saturated fat (g)	−0·3	−0·2	−0·3	−0·4	−0·4	−0·5	−0·3	−0·2	−0·3	−0·3	−0·4	−0·5
Fibre (g)	−1	−1	−1	−1	−1·1	−1·1	−1	−0·9	−1	−1	−1	−1·1
Incremental QALYs	0·052	0·051	0·052	0·055	0·051	0·051	0·054	0·051	0·054	0·058	0·054	0·054
Incremental NHS costs	−268	−299	−287	−266	−245	−233	−296	−318	−312	−297	−277	−270
**Sugar and Salt**												
Calories (kcal)	−58	−57	−58	−58	−57	−57	−70	−69	−70	−70	−70	−70
Sugar (g)	−5	−5·1	−5·1	−5	−4·9	−4·7	−7	−7·1	−7·1	−7	−6·9	−6·8
Sodium (mg)	−42·6	−41·5	−42·7	−42·9	−42·8	−43·3	−149·5	−147·7	−148·9	−150·2	−150·1	−151·1
Saturated fat (g)	−0·5	−0·5	−0·5	−0·5	−0·5	−0·5	−0·5	−0·4	−0·4	−0·5	−0·5	−0·5
Fibre (g)	−1	−1	−1	−1	−1	−1	−1	−0·9	−1	−1	−1	−1
Incremental QALYs	0·066	0·073	0·069	0·068	0·061	0·058	0·079	0·086	0·082	0·082	0·073	0·07
Incremental NHS costs	−374	−470	−407	−362	−315	−285	−470	−583	−505	−458	−403	−368

Results are from the secondary analyses incorporating industry response, comprising reformulation and tax pass-through. Mean daily change in calories, sugar, and salt are relative to the no-tax scenario. In the model, nutritional changes alter cardiometabolic risk factors and simulated incidence of health outcomes including cardiovascular disease and type 2 diabetes. Health-specific NHS costs and utility are assigned annually and discounted lifetime per person costs and QALYs are estimated. Incremental lifetime QALYs and NHS costs are relative to the no-tax scenario. IMD=Index of Multiple Deprivation. NHS=National Health Service. QALY=quality-adjusted life-year.

### Population subgroups

The results are presented by Index of Multiple Deprivation quintiles for equity analyses. We performed a subgroup equity analysis using the equity informed microsimulation model. We compared the incremental per person health gains or financial impacts across quintiles to evaluate whether each policy had a progressive or regressive effect. Key results presented by age, sex, and income groups in the appendix (pp 13–29).

### Role of the funding source

The funder had no role in the design, data collection, analysis and interpretation, or writing of the report.

## Results

Table 2 presents the average baseline consumption, nutritional intake, expenditure, and outcomes for the no tax simulation. Additional detail including prices and price changes is presented in the appendix (pp 4–29). The Sugar Snacks tax affected the lowest proportion of total calorie intake (8·84% [156·58 of 1770·60 kcal], 159 kcal) and weekly food expenditure (5·7% [£2·15 of £37·67), followed by the Unhealthy Food tax (calories: 16·51% [292·30 of 1770·60 kcal]; expenditure: 8·47% [£3·19 of £37·67]). The Sugar and Salt tax affected the largest proportion of calories (65·87% [1169·29 of 1770·60 kcal]) and food expenditure (48·63% [£18·32 of £37·67]), although price changes implied a 2·2% (£0·83) change in baseline expenditure. Exposure to the tax policies differ by Index of Multiple Deprivation groups.

**Table 2:** Population characteristics, nutritional intake, and food spending at baseline, and the predicted long-term outcomes under a no-tax scenario, overall, and by Index of Multiple Deprivation quintiles

		**All (N=64 016 711)**	**Index of Multiple Deprivation quintiles**				
			1 (most deprived)	2	3	4	5
UK population in millions*		64·02 (100%)	14·73 (23%)	13·58 (21%)	12·23 (19%)	12·33 (19%)	11·15 (17%)
Population groups by BMI (kg/m^2^)							
	<25	27·96 (44%)	6·53 (10%)	5·79 (9%)	5·01 (8%)	5·52 (9%)	5·11 (8%)
	25–29	20·45 (32%)	4·11 (6%)	4·38 (7%)	4·11 (6%)	4·02 (6%)	3·83 (6%)
	30–34	10 (16%)	2·41 (4%)	2·14 (3%)	2·07 (3%)	1·86 (3%)	1·53 (2%)
	≥35	5·61 (9%)	1·68 (3%)	1·27 (2%)	1·04 (2%)	0·93 (1%)	0·68 (1%)
Daily nutritional intake, (calories [kcal])		1771 (252)	1760 (261)	1766 (254)	1775 (251)	1785 (253)	1770 (240)
Population groups by BMI (kg/m^2^)							
	<25	1740 (245)	1737 (255)	1724 (240)	1757 (247)	1758 (252)	1728 (224)
	25–29	1812 (256)	1799 (265)	1819 (258)	1801 (254)	1823 (249)	1816 (249)
	30–34	1785 (255)	1783 (266)	1776 (255)	1786 (255)	1785 (247)	1797 (244)
	≥35	1747 (251)	1726 (246)	1753 (261)	1733 (240)	1783 (257)	1760 (244)
Sodium (mg)		2099·4 (317·3)	2112·6 (330·6)	2104·4 (320·4)	2099·3 (313·8)	2105·8 (315·3)	2068·9 (299·3)
Saturated fat (g)		25 (3·2)	24·6 (3·4)	24·8 (3·3)	25·1 (3·1)	25·3 (3·1)	25·2 (2·9)
Total sugar (g)		91·8 (11)	91·1 (11·8)	91·3 (10·9)	91·9 (11)	92·7 (10·8)	92·3 (10)
Free sugar (g)		56·8 (12·4)	58·7 (13·3)	56·9 (12·1)	56·3 (12·3)	56·6 (12·4)	54·9 (11·2)
Fibre (g)		17·8 (2·3)	17·1 (2·2)	17·6 (2·4)	17·9 (2·1)	18·1 (2·2)	18·3 (2·1)
Weekly expenditure on food (£)		£37·67 (8·19)	£34·31 (6·79)	£36·36 (7·8)	£38·23 (8·22)	£39·37 (8·27)	£41·2 (8·21)
Spending on food affected by the tax policy							
	Sugar Snacks	£2·15 (1·24)	£2·01 (1·1)	£2·08 (1·18)	£2·16 (1·26)	£2·24 (1·31)	£2·31 (1·37)
	Unhealthy Food	£3·19 (0·93)	£3·03 (0·89)	£3·12 (0·91)	£3·21 (0·93)	£3·3 (0·95)	£3·37 (0·96)
	Sugar and Salt	£18·32 (4·38)	£17·04 (3·84)	£17·79 (4·21)	£18·52 (4·41)	£19·03 (4·46)	£19·67 (4·54)
20-year health outcomes per 1 000 000							
	Incidence diabetes	89 812	113 395	94 891	89 355	75 535	68 777
	Incidence cardiovascular disease	126 074	125 916	121 163	131 822	124 606	127 584
	Death	253 026	196 438	231 842	270 299	286 293	297 815
Discounted lifetime outcomes*							
	Total quality-adjusted life-years	998 549 270	237 480 546	213 335 781	187 636 263	189 422 816	170 673 863
	NHS costs (£billion)	1260·6	321·2	272·0	237·9	225·2	204·2
	Formal social care costs (£billion)	8488·2	2545·9	1967·4	1578·1	1282·5	1114·3
	Social benefit costs (£billion)	2615·9	692·3	585·8	473·9	467·8	396·1

Data are N (%) or mean (SD). NHS=National Health Service.

* The synthetic population samples 2 000 000. The counts and outcomes presented are scaled to the size of the UK population aged from at least 5 years. Variability in the long-term outcomes across population subsets of 10 000 is presented in the appendix (p 20).

The Sugar Snacks, Unhealthy Food, and Sugar and Salt taxes changed mean daily calories consumed by –25 kcal (95% credible interval [CrI] –31 to –19), –42 kcal (–51 to –33), and –58 kcal (–66 to –50) and average weekly expenditure on food by –£0·34 (–0·59 to –0·10), –£0·42 (–0·72 to –0·09), and –£0·26 (–0·56 to –0·02), respectively. Under all three taxes, spending was reallocated across food groups, and spending on taxed food increased and spending on fruit and vegetable consumption fell (figure 2). Greater reductions in expenditure and smaller increases in tax burden were seen in least deprived Index of Multiple Deprivation groups compared with more deprived groups (appendix pp 13–14). The annual net tax revenue was estimated at £1·2 billion (95% CrI 1·02 to 1·28), £1·9 billion (1·75 to 2·06), and £2·5 billion (2·53 to 2·80) for the Sugar Snacks, Unhealthy Food, and Sugar and Salt tax policies.

All policies modelled reduced sugar, sodium, and saturated fat intake (table 1). Fibre intake decreased in all Index of Multiple Deprivation groups, whereas sodium decreased less in more deprived groups than in less deprived groups (table 1; appendix p 16).

When we looked at health, health care, and labour outcomes, the Sugar Snacks tax was estimated to have the smallest mean reduction in weight of –0·65 kg (95% CrI –0·84 to –0·49), followed by the Unhealthy Food tax (–1·12 kg [–1·36 to –0·88]), and the Sugar and Salt tax (–1·52 kg [–1·76 to –1·31]). Small decreases in systolic blood pressure (Sugar Snacks –0·17 mm Hg [95% CrI –0·11 to –0·02], Unhealthy Foods –0·08 mm Hg [–0·20 to –0·07], Sugar and Salt –0·29 mm Hg [–0·32 to –0·14]) and HbA_1c_ (Sugar Snacks –0·01% [95% CrI 0·00 to 0·00], Unhealthy Food –0·00% [–0·01 to 0·00], Sugar and Salt –0·01% [–0·01 to –0·01]) were estimated and small increases in cholesterol (Sugar Snacks 0·01 mmol/L [95% CrI 0·00 to 0·01], Unhealthy Food 0·01 mmol/L [0·00 to 0·02], Sugar and Salt 0·01 mmol/L [0·00 to 0·01]) driven predominantly by decreased fibre intake (appendix pp 18–19).

All tax policies improved per person quality-adjusted life-years and reduced cost burdens to the NHS and social care (table 3). The largest health gains (incremental QALYs 0·066) and costs savings (NHS cost –£374) were seen in the Sugar and Salt tax, which also reduced health inequalities as individuals in more deprived groups benefited from higher average improvements to health, indicated by lifetime QALYs (table 1). Social benefit receipts also decreased slightly per person, reflecting fewer years out of work.

**Table 3:** Lifetime health, costs, and labour market outcomes for a no tax design and the change in outcomes for each of the three food tax policy designs

		**Mean QALYs lived (per person)**	**NHS lifetime costs discounted (£ per person)**	**Social care costs (formal; £ per person)**	**Social care costs (informal; £ per person)**	**Social security benefit costs (£ per person)**	**Deaths in first 20 years (per 100 000)**	**Cardiovascular disease events in first 20 years (per 100 000)**	**Diabetes diagnosis in first 20 years (per 100 000)**	**Years inactive or unemployed (per person)**
No tax (“business as usual”)		15·60 (15·04 to 15·80)	19 691 (16 373 to 36 503)	132 594 (3515 to 635 982)	262 721 (0 to 751 014)	40 863 (39 588 to 42 051)	25 303 (24 524 to 27 094)	12 607 (8266 to 20 113)	8981 (5236 to 17 892)	4·665 (4·436 to 4·865)
Change from no tax scenario										
	Sugar Snacks	0·033 (0·022 to 0·048)	−168 (−461 to −73)	−535 (−3462 to 7)	−1060 (−4352 to 0)	−0·73 (−3·63 to 2·95)	−29 (−69 to −2)	−39 (−182 to 61)	−198 (−467 to −20)	−0·002 (−0·004 to −0·001)
	Unhealthy Food	0·052 (0·034 to 0·074)	−268 (−722 to −105)	−902 (−5509 to 11)	−1787 (−7169 to 0)	−0·67 (−4·66 to 4·52)	−52 (−114 to −3)	−73 (−290 to 102)	−344 (−800 to −42)	−0·004 (−0·005 to −0·002)
	Sugar and Salt	0·066 (0·046 to 0·094)	−374 (−1033 to −159)	−1281 (−7765 to 14)	−2539 (−9831 to 18)	−0·21 (−4·91 to 5·43)	−75 (−159 to −9)	−110 (−445 to 156)	−487 (−1092 to −72)	−0·004 (−0·006 to −0·002)

Data are mean (95% credible interval), in which the mean is informed by the base-case deterministic analysis and the credible interval is informed by the probabilistic sensitivity analysis. Mean probabilistic sensitivity analysis results are presented in the appendix (p 20). Social care costs are divided into formal care costs (including care home) and informal costs from the opportunity cost of friends and family of not working due to hours spent caring. All costs and QALYs are discounted annually at 3·5%. The no tax scenario, representing a scenario in which no change to consumption, is assumed. Diabetes represents type 2 diabetes only. The outcomes assuming no discounting are presented in the appendix (p 21). NHS=National Health Service. QALY=quality-adjusted life-year.

Incorporating industry response (reformulation and tax pass-through) in our model, showed greater changes in calories: –26 kcal for the Sugar Snacks tax, –44 kcal for the Unhealthy Food tax, and –70 kcal for the Sugar and Salt tax. Improvements in sugar intake were modest, whereas improvements in sodium intake were largest in the Sugar and Salt tax (appendix p 22). Altering tax pass-through by price partially offset the improvements from reformulation due to the under-shifting of the tax on average, particularly for low-income households (appendix pp 24–29). Tax pass-through widened differences in outcomes by Index of Multiple Deprivation in the Sugar Snacks and Unhealthy Food tax as pass-through was lower for cheaper products. However, the Sugar and Salt tax maintained a progressive effect on health. With industry response, all tax policies were estimated to have greater improvements in health and reduced NHS costs in the long run (table 1).

All deterministic sensitivity analyses estimated a calorie decline and most estimated a decrease in monthly food expenditure (appendix pp 23–29). Removing price-substitution effects (S5) and controlling for non-food spending in the price elasticities of demand (S1) generally resulted in lower calorie decline and increased spending on average with larger percentage increases in food spending in more deprived groups (appendix pp 28–29). Conversely, adjusting baseline consumption for under-reporting (S7) estimated larger calorie declines.

Applying an alternative price elasticities of demand model (S8) estimated larger average daily changes in calories consumed of –41, –67, and –71 kcals for the Sugar Snacks, Unhealthy Food, and Sugar and Salt taxes, but smaller monthly food expenditure reductions. Fibre decreased, but less than the base case.

## Discussion

Our analyses showed that the three food tax designs improved diet and health. Although the policies differ by tax rate structure and eligibility, the Sugar and Salt tax delivered the largest improvements in weight and lifetime QALYs, consistent with the broad eligibility criteria and its per gram tax structure. Crucially, this tax was the only design found to reduce health inequalities**.** We estimated savings to lifetime NHS costs up to £374 per person, and savings to social care costs. Estimates suggested social benefit savings were modest as they were offset by increased spending from longer life expectancy. These novel exploratory analyses illustrate an interesting economic trade-off between reduced social benefit spending annually as health improves, but increased lifetime spending, assuming labour outcomes are linked to EuroQol-5D. Average spending on food declined slightly across all taxes, although this was not consistent across the sensitivity analyses. Incorporating industry responses yielded small increases in health gains, with the Sugar and Salt tax remaining the most beneficial. However, price-variable tax pass-through could be detrimental to the distributional effect as lower passthrough rates in cheaper foods, more commonly purchased by more deprived groups, result in smaller changes to diets.

Different health effects across the Index of Multiple Deprivation groups between policy designs could reflect multifactorial interactions between consumption patterns, prices, price elasticities of demand, and health risks. Although more deprived groups have higher baseline risk for poor health outcomes, they consumed less cake, biscuits, and out-of-home foods, and were less price sensitive to sweet foods. This could partially explain why more deprived groups had a smaller average reduction in calories compared with less deprived groups in the Sugar Snacks and Unhealthy Food tax policies. Price elasticities did not consistently indicate higher price sensitivity at low incomes; outcomes are influenced by nuances in price elasticities and substitution preferences between income groups. Previous studies found that food taxes often yield greater health benefits for disadvantaged groups;^11^, ^13^ however, some evidence suggests that food taxes do not always reduce health inequalities, and effects can vary by tax design.^12^, ^14^, ^15^

Previous models have estimated that unhealthy food taxes reduce calories and improve health, and that outcomes differ by policy design.^9^, ^10^, ^11^, ^12^, ^13^, ^14^, ^15^ The UK Soft Drinks Industry Levy was modelled to increase estimated quality-adjusted life expectancy by approximately 11 days, an increase similar in magnitude to that of the estimated Sugar Snacks tax.^33^ Studies of unhealthy food or snacks taxes report similar calorie reductions of ranging from approximately –0·4% up to –3·0%, with some studies finding greater calorie reductions in lower-income groups^13^, others finding the opposite.^12^, ^14^, ^15^

A simulated decrease in average fibre intake across all tax designs can be partly explained by reduced spending on taxed products containing fibre, such as fried potatoes, and modelled changes in expenditure allocation across food groups due to a real-income effect. Consumption of fibre in the UK is already below the recommended amount. Despite this finding, the model has estimated a net health gain after accounting for negative health effects of reduced fibre intake on cardiometabolic health. The decline in fruit and vegetables could be an artefact of the demand framework; other studies have simulated mixed impacts on fruit and vegetable consumption and reductions in fibre intake.^9^, ^10^, ^11^, ^13^ When applying alternative elasticities in sensitivity analysis S8, we estimated a decline in fibre intake approximately half that estimated in the primary analysis, suggesting sensitivity to the estimates of cross price elasticities of demand.

A strength of this study lies in its detailed approach to modelling multiple components of the food system. Incorporating consumer heterogeneity in baseline consumption, prices, and price elasticity of demand captures differences in responses across the population, moving beyond broad averages that can mask disparities. The hierarchical demand system accommodates substitution between a large range of foods and considers expenditure allocation, varying by income. However, it cannot accommodate direct within-group switching from taxed to non-taxed alternatives due to data constraints, and the system-specific assumptions could affect estimates of food expenditure.

Changes in nutrient intake affect health through weight and cardiometabolic risks factors, minimising potential overestimation of health events from applying multiple, independently estimated food to health relationships and accounts for unintended consequences of combined nutritional effects. However, broader nutrition to health relationships, such as the effects of reduced fibre intake on digestive health and colorectal cancer risk, were not incorporated in the analysis.

Further limitations should be considered. The depth of the multi-pathway architecture necessitates a substantial number of underlying assumptions and simplifications to bridge the gap between consumption, nutrients, metabolism, and long-term health. The model used food survey data that might not fully reflect temporal changes, and could be affected by sampling and reporting biases.^2^ Although correcting for under-reporting in calories (sensitivity analysis S7) resulted in estimates of greater health improvements, evidence indicates that under-reporting varies by food groups rather than by product healthiness, leaving its net impact uncertain.^34^ Industry reformulation focused on calories, sugar, and sodium reductions, potentially overlooking other secondary health implications of replacing sugar with sweeteners or untaxed fats. The degree of product processing and price implications of reformulation were not incorporated.

Additionally, the analysis assumed an instantaneous and permanent shift in diet. Dynamic changes to the wider food system (eg, changing incomes, further industry adaptations, and political context) were also omitted. Evidence that the effect of food taxes might diminish due to changes in the wider economy suggest the lifetime benefits might be overestimated and warrants future research into the longevity of impact in more detail.^7^

Uncertainty analyses were limited to individual sensitivity analyses and a small exploratory probabilistic sensitivity analysis to maintain computational feasibility. The probabilistic analysis reflected parameter uncertainty only and did not fully capture structural uncertainty.

Political acceptability was considered in the tax designs but remains a crucial challenge in implementing food taxation, especially at a time of falling real incomes. Progressive distributional effects are important for the justification of the policy.

Complementary policies, such as subsidies for fruit and vegetables, which have been proposed to mitigate substitution away from healthy food and the financial burden of taxation, have been previously simulated to improve health when implemented alongside food taxes.^9^, ^10^, ^11^, ^13^ Further research could explore how revenue from the Sugar and Salt tax could be directed to complementary food subsidies.

This analysis did not adopt a complete societal perspective. The costs of implementation and the downstream application of revenue from the policies were not incorporated. Potentially larger enforcement costs associated with the Sugar and Salt tax could mitigate some of the savings. Understanding the broader distributional impact beyond health requires consideration of affordability and welfare implications for households.

To conclude, the Sugar and Salt tax was estimated to produce the most health gains, was the only design to narrow health inequalities, and had the largest impact from reformulation. Complementary policies, particularly subsidies for fruit and vegetables, could mitigate unintended negative effects.

### Contributors

### Data sharing

Individual participant data, including the Health Survey for England, the National Diet and Nutrition Survey, and the Living Cost and Food Survey, were obtained from the UK Data Service and the UK Data Service Secure Lab. Due to the terms of the data share agreement, the authors are not permitted to redistribute these datasets. Prospective users would need to access these data by registering a project directly with the UK Data Service. All other data used in this study are available via the cited literature in the supplementary material. The code for the National Institute for Health Research (NIHR) School for Public Health Research Obesity and Diabetes Prevention Model used in this analysis is available at https://doi.org/10.15131/shef.data.33936055.

## Declaration of interests

AAL has reported unpaid roles on the advisory board for the British Nutrition Foundation, as a trustee for Nutrition North, and as the Deputy Director of Fuse. CV has reported a non-financial collaboration with a UK supermarket chain to evaluate a product placement natural experiment, and reports payment or honoraria for a Healthy Retail Standard expert workshop for the Jersey Government. CR has reported a role as Food Systems Lead for the Nutrition Society, receiving support for attending meetings of the Nutrition Society, consultancy fees from the Wellcome Trust and FORMAS, and receiving payment or honoraria for speaking and acting as a PhD examiner. PB reported receiving support for attending the annual scientific meeting for the LightCOM trials. All other authors declare no competing interests.
