# Supplementary material for: The effect of food taxes in the UK on health and economic outcomes, and equity: a modelling study
Source: Lancet Public Health. 2026 Oct 20;11(11):e868–77. doi: 10.1016/S2468-2667(26)00196-9 (PMC13713165; doi:10.1016/S2468-2667(26)00196-9)
Supplement: Supplementary file 1 [file mmc1.pdf]

THE LANCET
Public Health

Supplementary appendix

This appendix formed part of the original submission and has been peer reviewed. We post it as supplied by the authors.

Supplement to: Pidd K, Wilson LB, Pryce R, et al. The effect of food taxes in the UK on health and economic outcomes, and equity: a modelling study. *Lancet Public Health* 2026; **11:** e868–77.

Supplementary Materials for:

The effect of food taxes in the UK on health and economic outcomes, and equity: a modelling study

Katharine Pidd, Luke Wilson, Robert Pryce, Alan Brennan, Damon Morris, Christian Reynolds, Amelia Lake, Helen Moore, Andrea Burrows, Rebecca Wells, Christina Vogel, Natalie Conner, Penny Breeze

These supplementary materials provide details of the technical assumptions, sources, data, and methods supporting the economic evaluation of three food tax policy designs considered in the manuscript. The analysis presented was conducted as part of the Health Economic Analysis incorporating effects on Labour outcomes, Households, Environment and Inequalities (HEALTHEI) project for food taxes in the United Kingdom. The project registration and study protocol are published (1, 2).

Contents

1 Supplementary Outcomes 4

1.1 Baseline Population Characteristics 4

1.2 Baseline Consumption and Change 6

1.3 Prices and Expenditure 11

1.4 Nutritional Intake 15

1.5 Weight and Cardiometabolic Risk Factors 18

1.6 Long term Health Outcomes 20

1.7 Industry Response 22

1.8 Sensitivity Analyses 23

2 The Food Tax Policies 30

2.1 Design 1: Sugar Snacks Tax 30

2.2 Design 2: Unhealthy food tax 31

2.3 Design 3: Sugar and Salt 33

3 Synthetic Population 35

3.1 Overview 35

3.2 Baseline Population Characteristics: Health Survey for England 35

3.3 Baseline Population Characteristics: Food Consumption 43

4 The Demand Model 51

4.1 Overview 51

4.2 Estimate consumption affected by tax 53

4.3 Estimate prices 57

4.4 Estimate change in consumption 59

4.5 Estimating Net Tax Spending and Revenue 73

4.6 Estimating the change in nutritional intake 73
4.7 Estimating the change in metabolic health 75
4.8 Industry Response 79
4.9 Sensitivity Analyses 83
5 Health Model – SMPH Obesity and Diabetes Prevention Model 92
5.1 Overview 92
5.2 Model Structure 93
5.3 Base Year for Data input and Analysis 94
5.4 GP Attendance in the General Population 95
5.5 Longitudinal Trajectories of weight, height and BMI 97
5.6 Longitudinal Trajectories of Metabolic Risk Factors 97
5.7 Metabolic Risk factor screening 106
5.8 Diagnosis and Treatment Initiation 106
5.9 Diabetes Diagnosis 106
5.10 Comorbid Outcomes and Mortality 109
5.11 Dementia 121
5.12 Dental Caries 124
5.13 Mortality 127
5.14 Direct Health Care Costs 129
5.15 Social Care costs 139
5.16 Labour market Outcomes 144
5.17 Utilities 146
5.18 Model Validation 149
5.19 Quality Adjusted Life years (QALYs) 150
6 Parameter Uncertainty 151
6.1 The Demand Model Parameter Uncertainty 151
6.2 The Health Model – SMPH Obesity and Diabetes Prevention Model 152
7 References 154

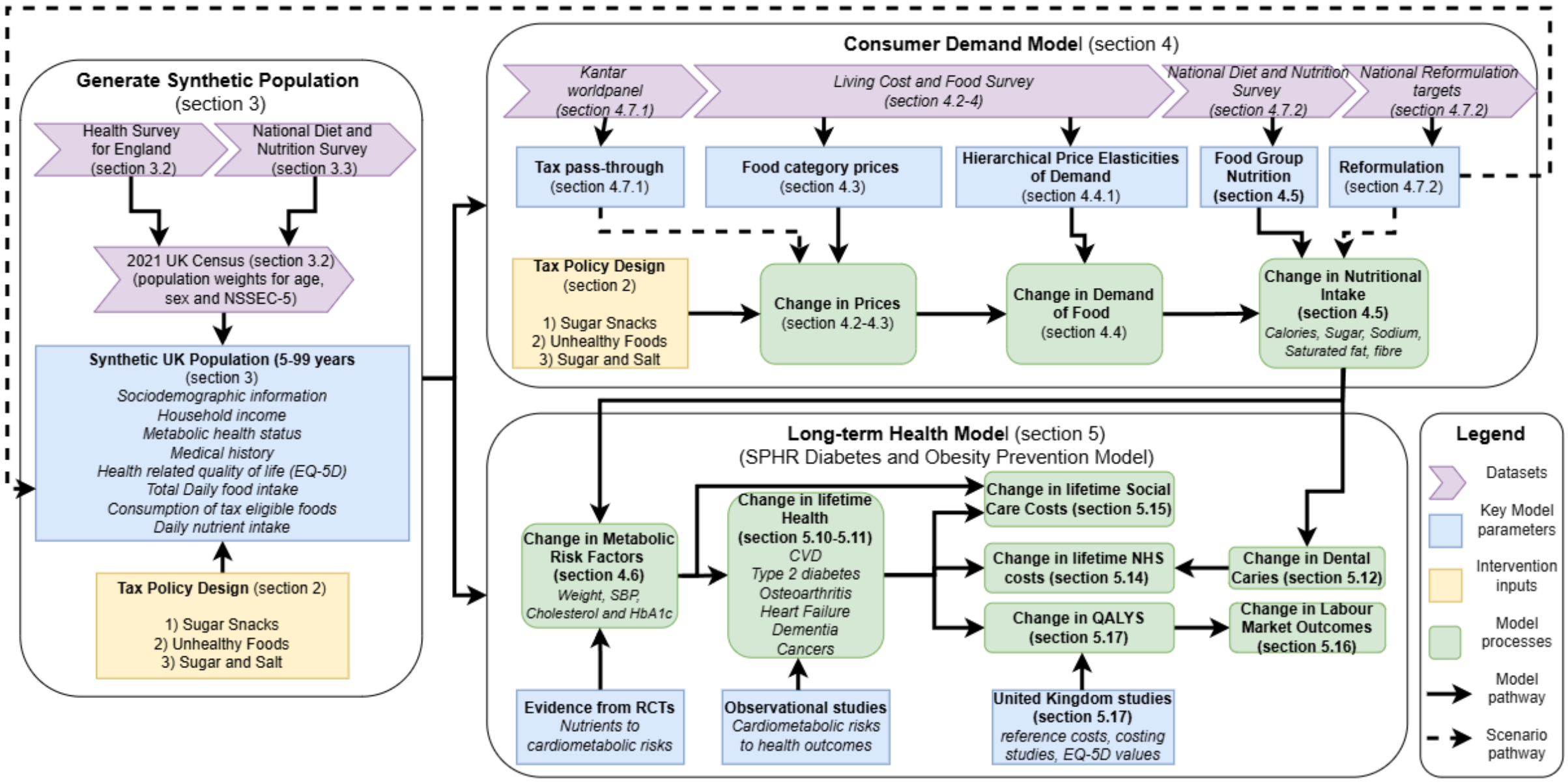

Figure 1.1.1: A model schematic presenting the pathways through which tax policies impact diet and health outcomes with supplementary section headings.

1 Supplementary Outcomes

1.1 Baseline Population Characteristics

Summary statistics of the model population are provided in Table 1.1.1.

Table 1.1.1: Baseline population characteristics (ages 5-100)

Category	Characteristic	Unit	Mean/N	SD/%
Sex	Male	N and %	975628	49%
	Female	N and %	1024372	51%
Age	Age	Mean and SD	43	22
Age group	Age 5 to 10	N and %	133939	7%
	Age 11 to 15	N and %	122945	6%
	Age 16 to 20	N and %	124178	6%
	Age 21 to 24	N and %	102146	5%
	Age 25 to 44	N and %	565396	28%
	Age 45 to 64	N and %	549203	27%
	Age 65 and over	N and %	402193	20%
Ethnicity	White	N and %	1639878	82%
	Black	N and %	82882	4%
	Asian	N and %	199822	10%
	Other (inc.Mixed)	N and %	77418	4%
Index of Multiple Deprivation	1	N and %	348414	17%
	2	N and %	385318	19%
	3	N and %	381969	19%
	4	N and %	424215	21%
	5	N and %	460084	23%
NS-SEC 5	1	N and %	682412	34%
	2	N and %	225797	11%
	3	N and %	210809	11%
	4	N and %	114856	6%
	5	N and %	472888	24%
	99	N and %	293238	15%
Smoking Status	Current Smoker	N and %	313109	16%
	Past Smoker	N and %	416841	21%
	Never Smoker	N and %	1270050	64%

Category	Characteristic	Unit	Mean/N	SD/%
Metabolic Risk Factors	BMI (kg/m2)	Mean and SD	27.6	5.8
	HbA1c (%)	Mean and SD	5.73	0.79
	Systolic Blood Pressure (mmHg)	Mean and SD	125	16.6
	Cholesterol (mmol/L)	Mean and SD	5.02	1.06
CVD History	History of CVD	N and %	35376	2%
	No History of CVD	N and %	1964624	98%
T2D Diagnosis	Diagnosis of T2D	N and %	140463	7%
	No Diagnosis of T2D	N and %	1859537	93%
QALY	QALY	Mean and SD	0.89	0.19

N refers to the number of people in the population subgroup. Abbreviations: SD; standard deviation. BMI; body mass index. CVD; cardiovascular disease. T2D; type 2 diabetes. QALY; Quality adjusted life years.

1.2 Baseline Consumption and Change

The mean and standard deviations of model population consumption assigned at baseline, before the tax is applied, and the change estimated for each tax policy design is reported by lower-level food group in Table 1.2.1. Total consumption at baseline by food groups is presented across Index of Multiple Deprivation population groups in Table 1.2.2. Total consumption is estimated from the National Diet and Nutrition Survey (2009-2019) (see section 3.2), with the proportion of food taxed in each scenario based on data from National Diet and Nutrition Survey (waves 9-11) (see section 4.2).

Table 1.2.1: Mean daily consumption at baseline and the estimated change due to each tax by food group (g)

	Unhealthy Food Tax				Sugar Snacks Tax				Sugar and Salt Tax			
Mean (SD)	Tax Eligible		Not Taxed		Tax Eligible		Not Taxed		Tax Eligible		Not Taxed	
	Pre-tax	Change	Pre-tax	Change	Pre-tax	Change	Pre-tax	Change	Pre-tax	Change	Pre-tax	Change
Bacon and ham	0		12.5 (3.4)	-0.1 (0.7)	0		12.5 (3.4)	0 (0.5)	12.5 (3.4)	-0.1 (0.4)	0	
Beef and veal	0		18.4 (5.6)	0 (1)	0		18.4 (5.6)	-0.1 (0.7)	0 (0.6)	0 (0)	18.3 (5.7)	-0.2 (0.6)
Powdered beverages	0.3 (0.7)	0 (0.1)	1.4 (1)	-0.1 (0.1)	0.4 (0.8)	-0.1 (0.1)	1.3 (1)	-0.1 (0.1)	0.6 (0.9)	0 (0.1)	1.2 (1)	0 (0)
Biscuits	11.2 (4.2)	-0.1 (0.4)	2.4 (3.8)	0.5 (0.8)	9.8 (4.8)	-2.2 (1.1)	3.8 (4.6)	0.2 (0.2)	13.5 (2.5)	-1.1 (0.4)	0.1 (0.5)	0
Breakfast Cereals	7.3 (9.6)	-0.9 (1.3)	18.2 (11.7)	0.1 (0.6)	0		25.4 (8.7)	0.2 (0.3)	23.6 (8.8)	-1.2 (1.2)	1.9 (4.9)	0.1 (0.2)
Brown Bread	0		28.4 (8.3)	0.4 (1)	0		28.4 (8.3)	0.2 (0.4)	28.1 (8.6)	0.7 (1)	0.3 (2.6)	0 (0.1)
Butter	0		4.4 (1.7)	0.1 (0.1)	0		4.4 (1.7)	0.1 (0)	3.7 (2.1)	0 (0)	0.7 (1.5)	0 (0)
Cakes and Puddings	19.1 (10.2)	-3.2 (1.7)	6.9 (8.9)	0.3 (0.4)	8.2 (8.7)	-1.4 (1.5)	17.8 (11.5)	1.2 (0.8)	24.3 (9.9)	-9.1 (7)	1.6 (4.7)	-0.4 (1.8)
Cheese	0		13.3 (2.4)	0.6 (0.3)	0		13.3 (2.4)	0.4 (0.2)	12.9 (2.7)	0.5 (0.2)	0.4 (1.4)	0 (0.1)
Poultry	0		32.7 (8)	0.4 (2.5)	0		32.7 (8)	-0.1 (1.3)	4.7 (9.2)	-0.1 (0.3)	28 (11.5)	-0.2 (0.9)
Chips and roasted potato products	0.5 (3.4)	-0.2 (1)	25.3 (10.1)	-3.2 (1.5)	0		25.8 (9.7)	-2.4 (1.1)	20 (12.2)	-1.3 (0.9)	5.8 (9.9)	-0.3 (0.5)
Chocolate confectionery	8.5 (3.3)	-0.7 (0.3)	0		8.4 (3.4)	-0.6 (0.4)	0.1 (0.9)	0 (0)	8.5 (3.3)	-0.1 (0.2)	0	
Crisps and savoury snacks	6.5 (3.5)	-0.4 (0.3)	0.4 (1.5)	0.1 (0.2)	0 (0.2)	0 (0)	7 (3.4)	0.9 (0.6)	6.9 (3.4)	0.9 (0.4)	0 (0.3)	0 (0)
Eggs and egg dishes	0		16.9 (4.3)	0.7 (0.3)	0		16.9 (4.3)	0.5 (0.3)	0.5 (2.6)	0 (0.1)	16.4 (4.9)	0.7 (0.3)
Fruit	0		91.3 (29.3)	-10.3 (3.6)	0		91.3 (29.3)	-8.7 (3.9)	0		91.3 (29.3)	-7.2 (3.2)
Fruit Juice	0		44.3 (16.9)	-2.4 (1.4)	0		44.3 (16.9)	-2.5 (1.4)	0		44.3 (16.9)	-0.9 (1.2)
Ice cream	5.2 (2.4)	-0.2 (0.2)	0.1 (0.7)	0 (0.1)	0 (0.3)	0 (0)	5.3 (2.4)	0.3 (0.2)	5.3 (2.4)	-0.9 (0.7)	0 (0.2)	0 (0)

	Unhealthy Food Tax				Sugar Snacks Tax				Sugar and Salt Tax			
Lamb	0		4.8 (2.3)	0 (0.3)	0		4.8 (2.3)	0 (0.2)	0		4.8 (2.3)	-0.1 (0.2)
Liver	0		0.9 (0.7)	0 (0.1)	0		0.9 (0.7)	0 (0)	0.6 (0.7)	0 (0)	0.3 (0.6)	0 (0)
Low fat milk	0		121.5 (27.6)	5.3 (2.5)	0		121.5 (27.6)	3.4 (1.9)	0		121.5 (27.6)	5.4 (2.2)
Margarine and oils	0		1.9 (0.6)	0 (0)	0		1.9 (0.6)	0 (0)	0 (0.1)	0 (0)	1.9 (0.6)	0 (0)
Meat pastries	0		8.9 (4.5)	0 (0.5)	0		8.9 (4.5)	0 (0.4)	8.1 (4.8)	-0.1 (0.3)	0.7 (2.5)	0 (0.1)
Meat ready meals	1.4 (4.8)	-0.7 (2.5)	17.9 (8.2)	0 (1)	0		19.3 (7.2)	0 (0.8)	17.2 (8.1)	-0.6 (0.6)	2.2 (5.3)	0 (0.2)
Milk drink	1 (2.6)	-0.2 (0.6)	5.5 (3.9)	-0.3 (0.2)	4.2 (4.1)	-0.6 (0.5)	2.3 (3.6)	-0.1 (0.2)	6.5 (3.5)	-0.7 (0.4)	0	
Nuts and seeds	0		4.1 (2.3)	-0.5 (0.3)	0 (0.2)	0 (0)	4.1 (2.3)	-0.4 (0.2)	1.1 (1.8)	-0.1 (0.1)	3 (2.4)	-0.2 (0.2)
Oily fish	0		8 (4.6)	-0.2 (0.3)	0		8 (4.6)	0 (0.2)	1.7 (3.6)	0 (0.2)	6.3 (5)	-0.1 (0.3)
Other breads	0		2 (1.1)	0 (0.1)	0		2 (1.1)	0 (0)	1.9 (1.1)	-0.2 (0.1)	0.1 (0.4)	0 (0)
Other meat and meat products	0		3.3 (2.7)	0 (0.2)	0		3.3 (2.7)	0 (0.2)	1.7 (2.5)	0 (0.1)	1.5 (2.4)	0 (0.1)
Other milk and creams	0		10 (4.7)	0.4 (0.3)	0		10 (4.7)	0.3 (0.2)	0		10 (4.7)	0.5 (0.3)
Potatoes and potato dishes	0		49.9 (15.4)	-6.5 (2.2)	0		49.9 (15.4)	-4.7 (2)	2.4 (9.2)	-0.3 (1)	47.4 (17.3)	-4.9 (2)
Other fish and fish dishes	0.1 (0.7)	0 (0.2)	9.8 (3.1)	-0.1 (0.3)	0		9.9 (3.1)	0 (0.2)	5.2 (4.8)	-0.1 (0.3)	4.7 (4.8)	-0.1 (0.3)
Out of home	0		133.9 (37.2)	1.7 (3.1)	7.8 (13.4)	-0.2 (0.3)	126.1 (37.2)	0.4 (2.1)	69 (29.7)	0.5 (1.2)	65 (26.1)	0.8 (1.2)
Pasta, rice and other cereals	6.1 (15.4)	-1.3 (3.2)	56.6 (27.7)	-0.3 (1.7)	0		62.7 (25.3)	0.5 (0.8)	33.2 (26.7)	-3.1 (2.6)	29.5 (26.4)	-2.2 (2.1)
Pork	0		5.9 (2.1)	0 (0.3)	0		5.9 (2.1)	0 (0.2)	0		5.9 (2.1)	-0.1 (0.2)
Reduced fat spread	0		6.7 (1.7)	0.1 (0.1)	0		6.7 (1.7)	0.1 (0.1)	6 (2.5)	0 (0)	0.7 (1.9)	0 (0)
Salad and raw vegetables	0		31.9 (11.6)	-4.2 (1.8)	0		31.9 (11.6)	-2.9 (1.3)	0		31.9 (11.6)	-4 (1.4)
Sausages, burgers and kebabs	0		13.3 (5.5)	0 (0.7)	0		13.3 (5.5)	0 (0.5)	12.7 (5.7)	-0.3 (0.4)	0.6 (2.2)	0 (0.1)
Savoury Condiments and pickles	0		24.9 (5)	-0.7 (0.7)	0		24.9 (5)	-0.2 (0.6)	16 (7.6)	-1.5 (1)	8.9 (7)	-0.3 (0.4)
Low calorie soft drinks	0		119.4 (58.5)	-9.6 (10.3)	0		119.4 (58.5)	-11.7 (14.8)	0		119.4 (58.5)	-4 (7.3)
Soft drinks	0		99.5 (65.4)	-4.2 (4)	0		99.5 (65.4)	-3.4 (3.6)	0		99.5 (65.4)	0.2 (3.1)
Soups	0		6.1 (3.3)	0 (0)	0		6.1 (3.3)	0 (0)	5.5 (3.6)	0 (0)	0.6 (2)	0 (0)

	Unhealthy Food Tax				Sugar Snacks Tax				Sugar and Salt Tax			
Sugar confectionery	2.5 (2.4)	-0.2 (0.2)	0.3 (1.1)	0 (0.1)	2.6 (2.5)	-0.2 (0.2)	0.2 (0.9)	0 (0.1)	2.7 (2.5)	-0.1 (0.1)	0.1 (0.6)	0 (0.1)
Sugar, preserves and sweet spreads	0		11.1 (3.2)	0.9 (0.5)	0		11.1 (3.2)	0.7 (0.3)	4.2 (4.3)	-2.5 (2.5)	6.9 (4.7)	-0.4 (0.7)
Tea, coffee and water	0		91.9 (35.5)	-3.4 (3.6)	0		91.9 (35.5)	-2.3 (4.5)	0		91.9 (35.5)	-0.4 (3)
Processed vegetables	0		102.8 (24.1)	-13.4 (4.1)	0		102.8 (24.1)	-9.6 (3.2)	30.6 (24.5)	-3.4 (2.8)	72.2 (31)	-7.5 (3.1)
Vegetable ready meal	1 (5.1)	-0.3 (1.3)	31.1 (9.2)	-4 (1.4)	0		32.1 (8)	-3 (1)	29.3 (10)	-3.9 (1.4)	2.8 (6.9)	-0.3 (0.7)
White bread	0.1 (1.4)	0 (0.1)	44.3 (12.2)	0.6 (1.5)	0		44.3 (12.1)	0.4 (0.6)	43.8 (12.6)	2.3 (1.7)	0.5 (3.8)	0 (0.3)
Coated or fried white fish	0.9 (2.4)	-0.3 (0.7)	5.6 (3)	-0.1 (0.2)	0		6.6 (2.3)	0 (0.1)	6.4 (2.5)	-0.2 (0.2)	0.2 (1.1)	0 (0)
Whole milk	0		36.5 (20.6)	1.5 (1)	0		36.5 (20.6)	1 (0.7)	0		36.5 (20.6)	1.6 (1.1)
Yoghurts and dairy desserts	7.4 (11.6)	-0.7 (1.2)	20.4 (13.4)	3.8 (2.4)	0		27.8 (9.1)	1.7 (0.9)	23.2 (12)	10.8 (14)	4.6 (9.5)	3 (8.4)

Consumption is presented as the mean (standard deviation) in grams (g).

Table 1.2.2: Mean daily consumption (g) before taxes are applied by Index of Multiple Deprivation population groups.

Mean (SD)	All	Index of Multiple Deprivation (Quintiles)				
		1 (Most Deprived)	2	3	4	5 (Least Deprived)
Bacon and ham	12.5 (3.4)	12.8 (3.7)	12.5 (3.5)	12.6 (3.3)	12.5 (3.2)	12.2 (3)
Beef and veal	18.4 (5.6)	18.6 (6.2)	18.4 (5.7)	18.5 (5.5)	18.5 (5.5)	17.7 (5)
Powdered beverages	1.7 (0.9)	1.6 (0.8)	1.7 (0.9)	1.8 (0.8)	1.8 (0.9)	1.9 (0.8)
Biscuits	13.6 (2.5)	12.9 (2.8)	13.4 (2.6)	13.7 (2.4)	13.8 (2.4)	14.2 (2)
Breakfast Cereals	25.4 (8.7)	22.7 (7.9)	24.7 (8.6)	26 (8.6)	26.6 (8.9)	28 (8.6)
Brown Bread	28.4 (8.3)	25.7 (8.5)	28 (8.5)	29 (7.8)	29.4 (8)	30.5 (7.6)
Butter	4.4 (1.7)	3.9 (1.7)	4.2 (1.7)	4.5 (1.8)	4.6 (1.7)	4.8 (1.7)
Cakes and Puddings	25.9 (9.7)	23.5 (8.3)	25.3 (9.2)	26.6 (9.9)	27 (10.2)	28.1 (10.3)
Cheese	13.3 (2.4)	12.8 (2.5)	13.3 (2.5)	13.5 (2.3)	13.5 (2.4)	13.8 (2.3)
Poultry	32.7 (8)	33.7 (7.7)	33.1 (7.8)	32.6 (8.1)	32.2 (8.1)	31.5 (8.2)

Mean<br>(SD)	All	Index of Multiple Deprivation (Quintiles)				
		**1 (Most Deprived)**	**2**	**3**	**4**	**5 (Least Deprived)**
Chips and roasted potato products	25.8<br>(9.7)	29.1<br>(10)	26.3<br>(9.4)	25.3<br>(9.3)	24.8<br>(10.4)	22.5<br>(7.7)
Chocolate confectionery	8.5<br>(3.3)	8.9<br>(3.6)	8.5<br>(3.1)	8.4<br>(3.1)	8.7<br>(3.6)	8.1<br>(2.6)
Crisps and savoury snacks	7<br>(3.4)	7.7<br>(3.5)	7.1<br>(3.3)	6.7<br>(3.3)	6.8<br>(3.5)	6.3<br>(3.2)
Eggs and egg dishes	16.9<br>(4.3)	16.6<br>(4.3)	16.7<br>(4.3)	17.3<br>(4.3)	17.2<br>(4.2)	17<br>(4.3)
Fruit	91.3<br>(29.3)	79.7<br>(27.7)	88.4<br>(29.1)	93.6<br>(27.8)	95.9<br>(29.4)	102.5<br>(27.1)
Fruit Juice	44.3<br>(16.9)	41.5<br>(15.6)	43.2<br>(16.5)	44<br>(17)	45.9<br>(17)	47.7<br>(17.9)
Ice cream	5.3<br>(2.4)	5<br>(2.4)	5.1<br>(2.4)	5.3<br>(2.4)	5.4<br>(2.4)	5.7<br>(2.4)
Lamb	4.8<br>(2.3)	4.6<br>(2.3)	4.6<br>(2.3)	5<br>(2.3)	4.9<br>(2.2)	5<br>(2.2)
Liver	0.9<br>(0.7)	0.8<br>(0.6)	0.9<br>(0.7)	0.9<br>(0.7)	0.9<br>(0.7)	1<br>(0.7)
Low fat milk	121.5<br>(27.6)	112.2<br>(27.8)	118.3<br>(27.7)	123.9<br>(26.8)	125.6<br>(26.4)	130.3<br>(24.8)
Margarine and oils	1.9<br>(0.6)	1.9<br>(0.6)	1.9<br>(0.6)	2<br>(0.6)	2<br>(0.6)	2<br>(0.6)
Meat pastries	8.9<br>(4.5)	9.5<br>(4.8)	9<br>(4.6)	8.8<br>(4.4)	8.8<br>(4.4)	8.1<br>(3.9)
Meat ready meals	19.3<br>(7.2)	19.6<br>(7.2)	19.4<br>(7)	19.3<br>(7.3)	19.7<br>(7.5)	18.7<br>(7.1)
Milk drink	6.5<br>(3.5)	6.4<br>(3.5)	6.5<br>(3.5)	6.5<br>(3.5)	6.5<br>(3.3)	6.7<br>(3.4)
Nuts and seeds	4.1<br>(2.3)	3.5<br>(2)	3.9<br>(2.2)	4.3<br>(2.3)	4.5<br>(2.3)	4.7<br>(2.4)
Oily fish	8<br>(4.6)	6.8<br>(4.8)	7.6<br>(4.6)	8.3<br>(4.4)	8.4<br>(4.3)	9.2<br>(4.4)
Other breads	2<br>(1.1)	1.7<br>(1)	1.9<br>(1.1)	2<br>(1.1)	2.1<br>(1.1)	2.3<br>(1)
Other meat and meat products	3.3<br>(2.7)	3<br>(2.7)	3.4<br>(3.4)	3.3<br>(2.4)	3.3<br>(2.7)	3.3<br>(2.3)
Other milk and creams	10<br>(4.7)	9.2<br>(4.9)	9.5<br>(4.5)	10.4<br>(4.7)	10.4<br>(4.3)	10.7<br>(4.6)
Potatoes and potato dishes	49.9<br>(15.4)	48.2<br>(15.9)	50.4<br>(17.2)	50.3<br>(14)	50.1<br>(15.1)	50.6<br>(13.7)
Other fish and fish dishes	9.9<br>(3.1)	9.1<br>(3.1)	9.7<br>(3.1)	10.1<br>(3)	10.2<br>(3)	10.6<br>(3)
Out of home	133.9<br>(37.2)	131.3<br>(35.7)	132.9<br>(36.8)	133.3<br>(37.8)	136<br>(37.7)	137.2<br>(38.1)
Pasta, rice and other cereals	62.7<br>(25.3)	65.9<br>(25.6)	63<br>(25.2)	62<br>(25.1)	62.6<br>(26.6)	59.1<br>(23.4)
Pork	5.9<br>(2.1)	5.6<br>(2.2)	5.9<br>(2.2)	6<br>(2)	6<br>(1.9)	6<br>(1.9)
Reduced fat spread	6.7<br>(1.7)	6.8<br>(1.8)	6.8<br>(1.8)	6.7<br>(1.7)	6.5<br>(1.7)	6.5<br>(1.6)
Salad and raw vegetables	31.9<br>(11.6)	28.1<br>(11)	30.8<br>(11.1)	33<br>(11.3)	33.2<br>(11.8)	35.4<br>(11.4)

Mean (SD)	All	Index of Multiple Deprivation (Quintiles)				
		**1 (Most Deprived)**	**2**	**3**	**4**	**5 (Least Deprived)**
Sausages, burgers and kebabs	13.3 (5.5)	14.6 (5.8)	13.7 (5.5)	13 (5.5)	12.7 (5.3)	12 (5)
Savoury Condiments and pickles	24.9 (5)	25.4 (5.1)	25.3 (5.1)	24.8 (5.1)	24.7 (4.9)	24.1 (4.9)
Low calorie soft drinks	119.4 (58.5)	126.1 (58.6)	122.5 (58.2)	118.5 (58.9)	114.4 (57.7)	113 (58)
Soft drinks	99.5 (65.4)	117.7 (73.6)	102.6 (64.2)	94.8 (63.4)	94.6 (64.3)	81.9 (51.3)
Soups	6.1 (3.3)	5.2 (3.1)	5.8 (3.3)	6.4 (3.2)	6.6 (3.3)	7.1 (3.3)
Sugar confectionery	2.8 (2.5)	2.9 (2.7)	2.8 (2.5)	2.7 (2.5)	2.7 (2.4)	2.8 (2.5)
Sugar, preserves and sweet spreads	11.1 (3.2)	11 (3.2)	11.1 (3.2)	11.2 (3.3)	11.2 (3.2)	11 (3.3)
Tea, coffee and water	91.9 (35.5)	93.5 (35.5)	92.9 (35.6)	91.4 (35.3)	91 (35.5)	89.9 (35.4)
Processed vegetables	102.8 (24.1)	95.9 (23.8)	101.8 (24.8)	104.5 (22.9)	105.9 (23.4)	107.8 (23.4)
Vegetable ready meal	32.1 (8)	32.5 (8.3)	32.2 (8.3)	32 (7.6)	32.5 (8.1)	31.1 (7.3)
White bread	44.3 (12.1)	47 (11.8)	45 (12)	44.1 (12.2)	43.2 (12.2)	41.5 (11.8)
Coated or fried white fish	6.6 (2.3)	6.7 (2.4)	6.7 (2.4)	6.6 (2.2)	6.4 (2.1)	6.3 (2)
Whole milk	36.5 (20.6)	41.2 (21.8)	37 (19.9)	35.4 (19.8)	35.6 (22.1)	31.6 (16.9)
Yoghurts and dairy desserts	27.8 (9.1)	25.1 (8.2)	26.7 (8.8)	28.4 (9)	29.1 (9.3)	30.6 (9.3)

Consumption is presented as the mean (standard deviation) in grams (g).

1.3 Prices and Expenditure

1.3.1 Pre-tax prices and change

Mean price per gram of each lower-level food group and the percentage change in these prices as a result of each tax policy design are reported in Table 1.3.1. Prices by income tertiles are presented in Table 4.3.2.

Table 1.3.1: Mean Price at Baseline and Price Change by food group for each Policy

Food Groups	All	Unhealthy Food Tax	Sugar Snacks Tax	Sugar and Salt Tax
	Pre-tax	Change	Change	Change
Bacon and ham	0.855	0%	0%	0.2%
Beef and veal	0.94	0%	0%	0.2%
Powdered beverages	1.013	20%	29.5%	11.9%
Biscuits	0.546	20%	24.5%	14.9%
Breakfast Cereals	0.351	20%	0%	12.2%
Brown Bread	0.149	0%	0%	7.6%
Butter	0.631	0%	0%	0.3%
Cakes and Puddings	0.579	20%	24.3%	11.3%
Cheese	0.757	0%	0%	0.4%
Poultry	0.905	0%	0%	0.8%
Chips and roasted potato products	0.18	20%	0%	1.6%
Chocolate confectionery	1.068	20%	20.7%	15.4%
Crisps and savoury snacks	0.838	20%	12.6%	1.1%
Eggs and egg dishes	0.355	0%	0%	1.2%
Fruit	0.276	0%	0%	0%
Fruit Juice	0.142	0%	0%	0%
Ice cream	0.357	20%	32.4%	20.4%
Lamb	1.134	0%	0%	0%
Liver	0.365	0%	0%	0.6%
Low fat milk	0.063	0%	0%	0%
Margarine and oils	0.358	0%	0%	0.4%
Meat pastries	0.645	0%	0%	0.7%
Meat ready meals	0.681	20%	0%	1.2%
Milk drink	0.248	20%	9.7%	10.8%
Nuts and seeds	0.848	0%	18.2%	2.1%
Oily fish	1.089	0%	0%	0.3%

Food Groups	All	Unhealthy Food Tax	Sugar Snacks Tax	Sugar and Salt Tax
Other breads	0.396	0%	0%	2.5%
Other meat and meat products	0.717	0%	0%	0.7%
Other milk and creams	0.234	0%	0%	72.5%
Potatoes and potato dishes	0.103	0%	0%	3.2%
Other fish and fish dishes	1.918	20%	0%	0.1%
Out of home	1.027	0%	10.6%	2.1%
Pasta, rice and other cereals	0.366	20%	0%	1.6%
Pork	0.589	0%	0%	0%
Reduced fat spread	0.356	0%	0%	0.5%
Salad and raw vegetables	0.235	0%	0%	4.3%
Sausages, burgers and kebabs	0.645	0%	0%	0.8%
Savoury Condiments and pickles	0.434	0%	0%	7.2%
Low calorie soft drinks	0.065	0%	0%	0%
Soft drinks	0.083	0%	0%	0%
Soups	0.266	0%	0%	1.5%
Sugar confectionery	0.798	20%	26.8%	19.6%
Sugar, preserves and sweet spreads	0.291	0%	0%	67.5%
Tea, coffee and water	0.19	0%	0%	0%
Processed vegetables	0.169	0%	0%	7.3%
Vegetable ready meal	0.564	20%	0%	1.5%
White bread	0.142	0%	0%	7.6%
Coated or fried white fish	0.82	20%	0%	0.4%
Whole milk	0.063	20%	0%	0%
Yoghurts and dairy desserts	0.287	20%	78.3%	12.3%

Prices are presented as pence per gram of food.

1.3.2 Food Expenditure and Tax Burden

The mean weekly food expenditure per person is presented in Table 1.3.2 and Table 1.3.3 by population subgroups and by higher-level food category. The change in total food spending, the change in spending on taxes related to foods and the new tax burden of the three tax policies by age, sex, deprivation, income and ethnicity is presented in Table 1.3.2. The methods of estimate tax spending are provided in Supplementary Materials Section 4.5. The change in spending by food category, for both the base-case, deterministic analysis and the variability across probability sensitivity analysis are presented in Table 1.3.3.

Table 1.3.2: Mean weekly food and food tax spending (£) per person pre-tax and simulated changes due to tax policies by population groups across age, sex, deprivation, income and ethnicity.

		Pre-Tax		Change due to tax policy								
				Sugar Snacks			Unhealthy Foods			Sugar & Salt		
Population group		Total Spend	Tax burden	Total Spend change	Net Tax Spend	Tax Burden	Total Spend change	Net Tax Spend	Tax Burden	Total Spend change	Net Tax Spend	Tax Burden
	**All**	**£37.67**	**5.63%**	**-£0.34**	**£0.34**	**0.92%**	**-£0.42**	**£0.57**	**1.51%**	**-£0.26**	**£0.80**	**2.12%**
Sex	Male	£40.67	5.60%	-£0.34	£0.37	0.91%	-£0.43	£0.61	1.51%	-£0.26	£0.85	2.10%
	Female	£34.81	5.66%	-£0.35	£0.32	0.92%	-£0.42	£0.53	1.52%	-£0.27	£0.74	2.14%
Age group	Under 16	£34.92	7.17%	-£0.11	£0.46	1.33%	-£0.04	£0.72	2.07%	£0.06	£0.90	2.57%
	16 to 24	£35.73	5.95%	-£0.10	£0.38	1.06%	-£0.09	£0.57	1.59%	£0.02	£0.77	2.16%
	25 to 64	£40.09	5.66%	-£0.45	£0.33	0.82%	-£0.58	£0.55	1.36%	-£0.36	£0.78	1.93%
	Over 64	£33.80	4.32%	-£0.34	£0.29	0.86%	-£0.42	£0.54	1.59%	-£0.37	£0.81	2.39%
Index of Multiple Deprivation	IMD 1 (most deprived)	£34.31	5.26%	-£0.15	£0.35	1.03%	-£0.15	£0.55	1.62%	-£0.08	£0.78	2.28%
	IMD 2	£36.36	5.46%	-£0.27	£0.35	0.95%	-£0.33	£0.56	1.55%	-£0.19	£0.79	2.18%
	IMD 3	£38.23	5.65%	-£0.38	£0.34	0.89%	-£0.48	£0.57	1.49%	-£0.30	£0.80	2.09%
	IMD 4	£39.37	5.80%	-£0.43	£0.34	0.88%	-£0.55	£0.58	1.48%	-£0.35	£0.81	2.05%
	IMD 5 (least deprived)	£41.20	6.02%	-£0.55	£0.34	0.81%	-£0.71	£0.59	1.42%	-£0.46	£0.81	1.96%
Income Tertiles	Lower	£31.16	4.60%	£0.06	£0.36	1.15%	£0.12	£0.55	1.75%	£0.08	£0.78	2.50%
	Middle	£36.90	5.59%	-£0.42	£0.34	0.91%	-£0.44	£0.56	1.53%	-£0.26	£0.82	2.23%
	Higher	£47.57	6.60%	-£0.82	£0.33	0.70%	-£1.16	£0.61	1.28%	-£0.75	£0.79	1.67%
Ethnicity	Black	£33.92	5.42%	-£0.14	£0.35	1.04%	-£0.14	£0.55	1.63%	-£0.06	£0.77	2.26%
	Asian	£35.98	5.55%	-£0.18	£0.37	1.02%	-£0.20	£0.58	1.60%	-£0.09	£0.79	2.21%

	Other (inc. White)	£38.05	5.65%	-£0.37	£0.34	0.90%	-£0.46	£0.57	1.50%	-£0.29	£0.80	2.10%

Results reflect base-case analysis. Changes to spending on tax (Net Tax Spend) is an estimate of net change in existing tax spend, including VAT and Soft Drinks Industry Levy (SDIL), plus the amount spent on the new tax designs, assuming 100% of tax is passed on to consumers via price increase. Tax burden is calculated as total post-tax tax spend as a proportion of pre-tax food spending.

Table 1.3.3: Mean Weekly Spending (£) changes per person by higher level food category across the base case, deterministic analysis and the estimates across the PSA analysis.

		**Sugar Snacks**			**Unhealthy Foods**			**Sugar & Salt**		
**Food Category**	**Pre-Tax**	**Det. (£)**	**Det. (%)**	**PSA (%)**	**Det. (£)**	**Det (%)**	**PSA (%)**	**Det. (£)**	**Det (%)**	**PSA (%)**
**Butter and fats**	£0.41	£0.01	1.3%	1.2% [0.4%, 2%]	£0.01	1.7%	1.7% [0.5%, 2.7%]	£0.00	0.7%	0.7% [-0.3%, 1.8%]
**Dairy**	£1.99	£0.06	2.8%	2.8% [1.4%, 4.2%]	£0.09	4.3%	4.3% [2.4%, 6.2%]	£0.09	4.4%	4.3% [2.8%, 6%]
**Drinks**	£3.03	-£0.13	-4.4%	-4.6% [-6.3%, -2.7%]	-£0.15	-4.8%	-5.1% [-7.4%, -2.5%]	-£0.03	-1.1%	-1.2% [-3.5%, 1.1%]
**Fish**	£2.32	-£0.01	-0.4%	-0.5% [-2.3%, 1.3%]	-£0.06	-2.4%	-2.3% [-4.6%, 0.1%]	-£0.05	-2.1%	-2.4% [-4.4%, -0.1%]
**Fruit**	£2.02	-£0.19	-9.4%	-9.5% [-10.9%, -7.9%]	-£0.22	-11.1%	-11.3% [-13.3%, -9.2%]	-£0.15	-7.6%	-7.6% [-9.3%, -5.8%]
**Meat**	£6.76	-£0.04	-0.6%	-0.6% [-3.8%, 2.7%]	-£0.04	-0.6%	-0.2% [-4.6%, 4.6%]	-£0.09	-1.3%	-1.4% [-5%, 2.3%]
**Other**	£0.76	-£0.01	-0.7%	-0.8% [-2.2%, 0.6%]	-£0.02	-2.3%	-2.7% [-4.3%, -0.8%]	-£0.02	-2.7%	-2.8% [-4.7%, -0.8%]
**Out of home**	£9.84	£0.03	0.3%	0.2% [-1.8%, 2.4%]	£0.06	0.6%	0.6% [-2.2%, 3.4%]	£0.14	1.4%	1.3% [-1.3%, 3.7%]
**Savoury foods**	£3.15	£0.03	0.8%	0.9% [-1.6%, 3.2%]	£0.01	0.5%	0.3% [-2.9%, 3.4%]	£0.01	0.2%	0.3% [-2.5%, 3.2%]
**Snacks**	£1.72	£0.15	8.9%	8.8% [5%, 12.3%]	£0.23	13.6%	13.6% [8.7%, 18.1%]	£0.20	11.5%	11.6% [8%, 15.2%]
**Sugar and sweet foods**	£1.97	£0.11	5.7%	5.8% [3.3%, 8.3%]	£0.14	6.9%	7.1% [3.4%, 11.1%]	£0.02	0.9%	1.1% [-3%, 5.1%]
**Vegetables**	£3.70	-£0.35	-9.4%	-9.4% [-10.8%, -7.9%]	-£0.48	-13.0%	-12.9% [-14.8%, -10.7%]	-£0.38	-10.2%	-10.2% [-11.9%, -8.3%]
**Total**	**£37.67**	**-£0.34**	**-0.9%**	**-0.9% [-1.6%, -0.3%]**	**-£0.42**	**-1.1%**	**-1.1% [-1.9%, -0.2%]**	**-£0.26**	**-0.7%**	**-0.8% [-1.5%, -0.1%]**

Det. refers to the mean per person results from the deterministic analysis (the base case). The PSA, probabilistic sensitivity analysis, results presented are the mean per person change across parameter runs and the 95% credible interval (CrI), presented in the format Mean [Lower CrI, Upper CrI].

1.4 Nutritional Intake

The mean percentage change in nutrient intake is presented in Figure 1.4.1. The mean change in daily calorie intake by age, sex, index of multiple deprivation (IMD) and income tertile groups are presented in Figure 1.4.2 a, b, c and d respectively. Nutrient intake of the model population and the change in nutrient intake by tax policy design is presented in Table 1.4.1 as mean and standard deviation. Energy is provided by population subgroups.

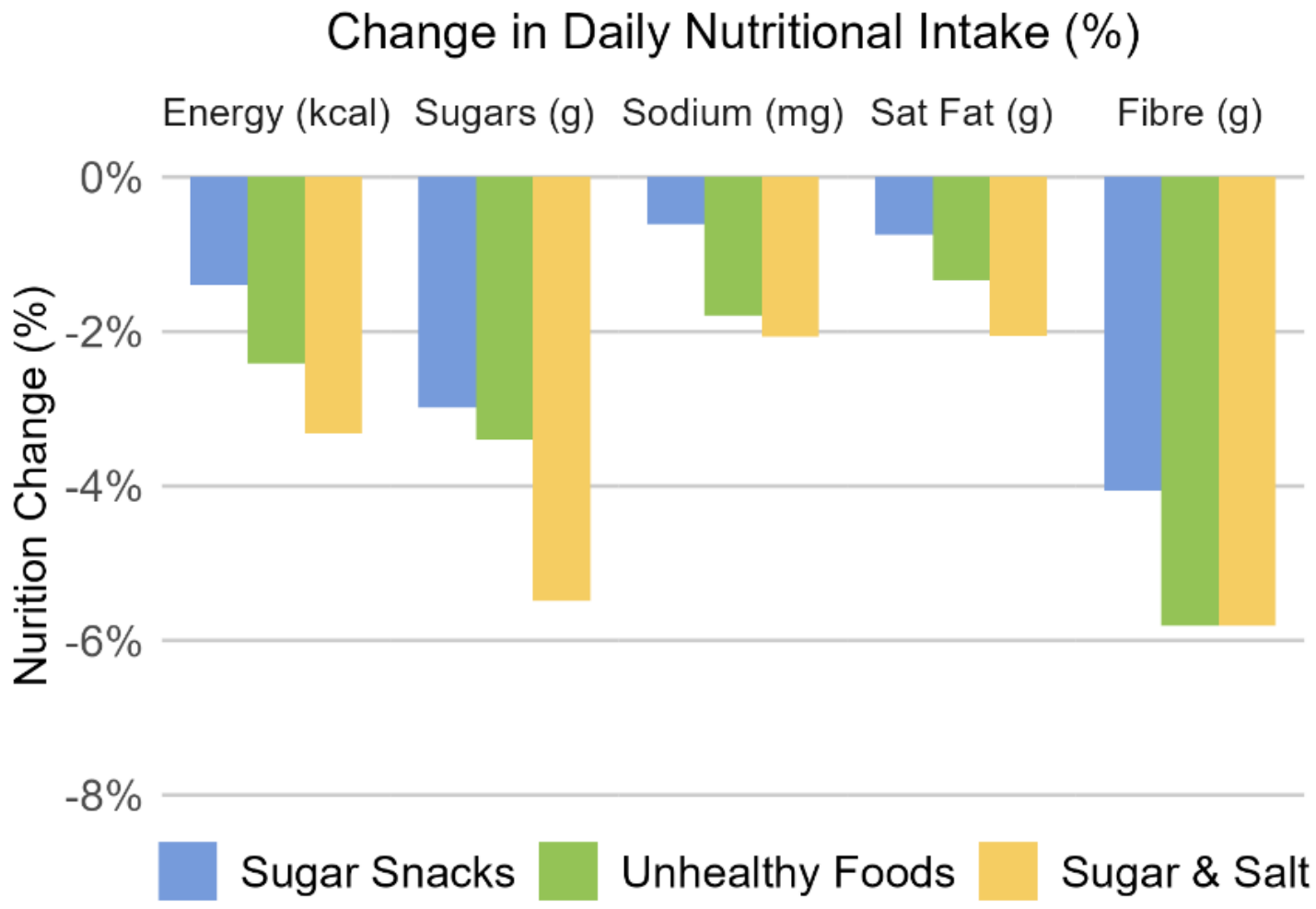

Figure 1.4.1: Average percentage change in total nutritional intake for the three food tax policy designs

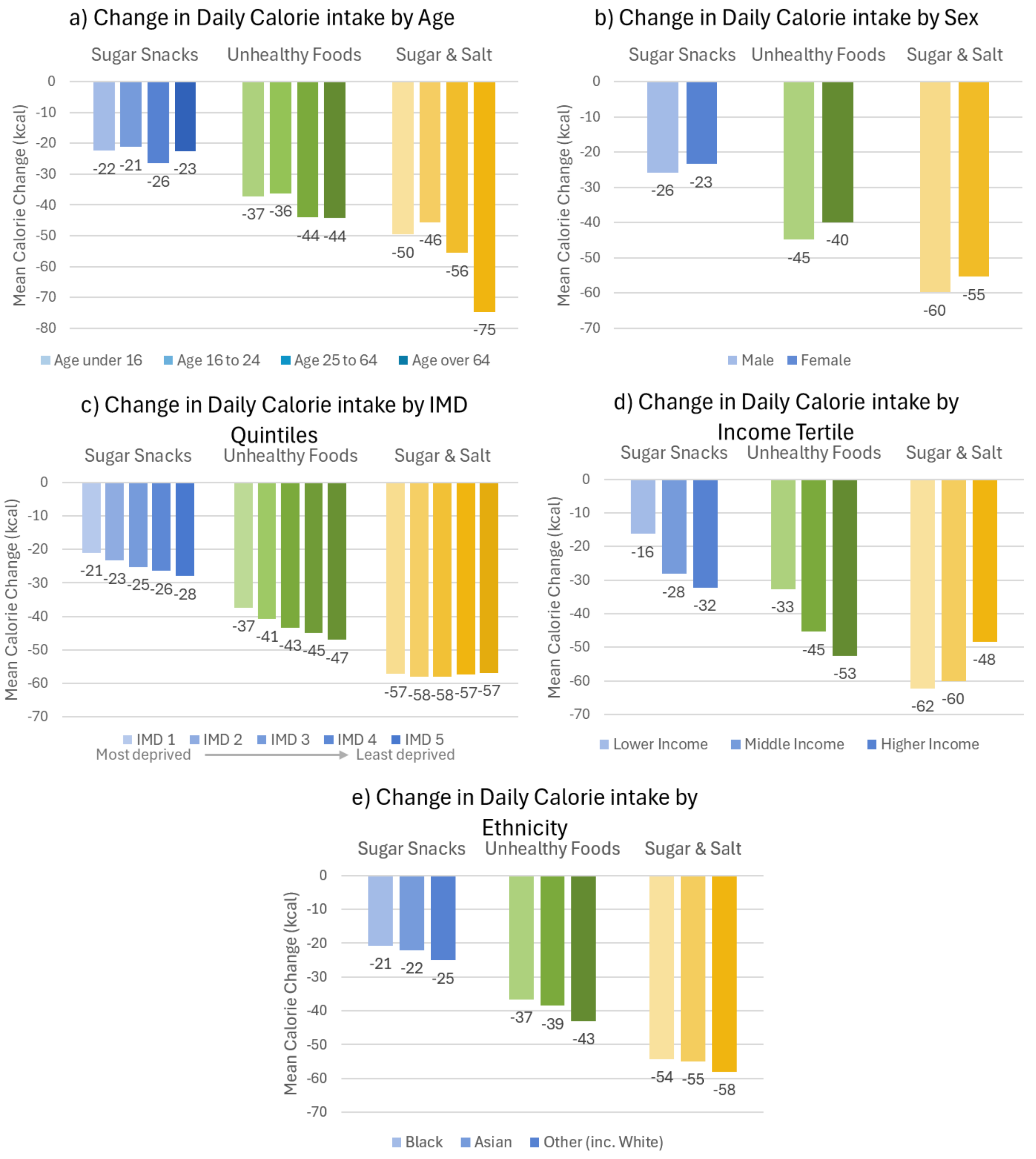

Figure 1.4.2: Mean daily change in calorie intake by age (a) and sex (b), index of multiple deprivation (IMD) (c), and income tertiles (d), ethnicity (e) groups for each food tax policy design. *Figure colours vary by tax design, the shade represents the population groups. In figure a, c and d the shade transitions from light to dark from lower to higher age, IMD quintile and income groups respectively. In figure b the lighter shade represents the mean for males, and the darker shade represents the mean for the females.*

Table 1.4.1: Mean Daily Nutritional Intake at Baseline and Change in Nutritional Intake for each Policy

Nutrient Component	Population		Unhealthy Food Tax	Sugar Snacks Tax	Sugar and Salt Tax
Mean (SD)		Pre-tax	Change	Change	Change
Energy (kcal)	All	1771 (252)	-42 (15)	-25 (11)	-58 (22)
	IMD 1	1760	-37 (15)	-21 (11)	-57 (22)
	IMD 2	1766	-41 (15)	-23 (11)	-58 (23)
	IMD 3	1775	-43 (15)	-25 (11)	-58 (22)
	IMD 4	1785	-45 (14)	-26 (11)	-57 (22)
	IMD 5	1770	-47 (13)	-28 (10)	-57 (22)
Carbohydrate (g)	All	218 (28)	-7.4 (1.9)	-4.7 (1.6)	-10.4 (5)
Sodium (mg)	All	2099 (317)	-37.5 (22.5)	-12.9 (16.2)	-42.6 (16)
Saturated fatty acids (g)	All	25 (3)	-0.3 (0.3)	-0.2 (0.2)	-0.5 (0.2)
Cis Monounsaturated fatty acids (g)	All	24 (4)	-0.5 (0.3)	-0.2 (0.2)	-0.7 (0.3)
Cis n-3 fatty acids (g)	All	2 (0)	-0.1 (0)	0 (0)	-0.1 (0)
Cis n-6 fatty acids (g)	All	9 (1)	-0.3 (0.1)	-0.1 (0.1)	-0.3 (0.1)
AOAC Fibre (g)	All	18 (2)	-1 (0.2)	-0.7 (0.2)	-1 (0.3)
Total sugars (g)	All	92 (11)	-3.1 (0.8)	-2.7 (0.8)	-5 (2.3)
Free Sugars (g)	All	57 (12)	-1.4 (0.7)	-1.3 (0.7)	-4 (2.6)

Energy (kcal) is presented for the whole population (All) and by Index of Multiple Deprivation (IMD) Quintiles (1= Most Deprived, 5= Least Deprived). The remaining nutrients are presented only for the whole population. SD stands for standard deviation. SD for change in nutritional intake estimated across 200 model runs, each with 10,000 people from the synthetic population.

1.5 Weight and Cardiometabolic Risk Factors

Mean baseline weight, systolic blood pressure, cholesterol and glycated haemoglobin are presented in Table 1.5.1. Weight is presented by broad age groups, those under 25 years and those above, as well as by sex and IMD group encompassing all ages. The remain metabolic risk factors are only estimated in those 25 years and over and therefore the average for these ages is presented. The change in metabolic risk factors is estimated from the change in nutritional intake as a result of changing consumption in response to the tax. The methods used to translate changes in nutritional intake to metabolic risk factors in presented in the supplementary materials section 4.6.

Table 1.5.1: Metabolic Risk Factors at Baseline and simulated change from each tax policy by social groups using the consumer demand model

Mean (SD)			Unhealthy Food Tax	Sugar Snacks Tax	Sugar and Salt Tax
**Risk factor**	**Population**	**Pre-tax**	**Change**	**Change**	**Change**
Weight (g)	All	73.3 (21.71)	-1.12 (0.39)	-0.65 (0.29)	-1.52 (0.61)
	Male	77.88 (22.43)	-1.16 (0.43)	-0.67 (0.32)	-1.55 (0.62)
	Female	68.94 (20.06)	-1.08 (0.35)	-0.63 (0.27)	-1.50 (0.59)
	Ages 25 and over	79.08 (18.19)	-1.16 (0.38)	-0.67 (0.3)	-1.61 (0.62)
	Ages 5 to 24	55.16 (21.87)	-0.98 (0.41)	-0.59 (0.28)	-1.26 (0.49)
	IMD 5	80.21 (19.6)	-1.04 (0.39)	-0.58 (0.29)	-1.61 (0.62)
	IMD 4	79.15 (18.29)	-1.12 (0.38)	-0.64 (0.3)	-1.62 (0.63)
	IMD 3	79.58 (17.66)	-1.18 (0.37)	-0.69 (0.29)	-1.61 (0.62)
	IMD 2	78.66 (17.8)	-1.23 (0.36)	-0.72 (0.29)	-1.61 (0.62)
	IMD 1	77.63 (17.26)	-1.27 (0.34)	-0.75 (0.28)	-1.59 (0.6)
Systolic Blood Pressure (mmHg)	Ages 25 and over	124.99 (16.55)	-0.17 (0.22)	-0.08 (0.14)	-0.29 (0.35)
	IMD 5	124.58 (16.58)	-0.16 (0.22)	-0.07 (0.13)	-0.31 (0.36)
	IMD 4	124.52 (16.35)	-0.17 (0.22)	-0.07 (0.13)	-0.3 (0.36)
	IMD 3	125.49 (16.82)	-0.18 (0.22)	-0.08 (0.14)	-0.29 (0.34)
	IMD 2	125.36 (16.44)	-0.18 (0.23)	-0.09 (0.14)	-0.28 (0.34)
	IMD 1	125.03 (16.54)	-0.17 (0.23)	-0.08 (0.14)	-0.26 (0.34)
Total Cholesterol (mmol/L)	Ages 25 and over	5.02 (1.06)	0.01 (0.01)	0.01 (0.01)	0.01 (0.01)
	IMD 5	4.91 (1.06)	0.01 (0.01)	0.01 (0.01)	0.01 (0.01)
	IMD 4	4.96 (1.06)	0.01 (0.01)	0.01 (0.01)	0.01 (0.01)
	IMD 3	5.04 (1.07)	0.01 (0.01)	0.01 (0.01)	0.01 (0.01)
	IMD 2	5.1 (1.06)	0.01 (0.01)	0.01 (0.01)	0.01 (0.01)

Mean (SD)			**Unhealthy Food Tax**	**Sugar Snacks Tax**	**Sugar and Salt Tax**
	IMD 1	5.11 (1.04)	0.01 (0.01)	0.01 (0.01)	0.01 (0.01)
High-density Lipoproteins (mmol/L)	Ages 25 and over	1.45 (0.42)	0 (0)	0 (0)	0 (0)
	IMD 5	1.38 (0.4)	0 (0)	0 (0)	0 (0)
	IMD 4	1.43 (0.41)	0 (0)	0 (0)	0 (0)
	IMD 3	1.45 (0.41)	0 (0)	0 (0)	0 (0)
	IMD 2	1.49 (0.43)	0 (0)	0 (0)	0 (0)
	IMD 1	1.53 (0.44)	0 (0)	0 (0)	0 (0)
HbA1c (%)	Ages 25 and over	5.73 (0.79)	-0.01 (0.01)	0 (0.01)	-0.01 (0.02)
	IMD 5	5.8 (0.87)	0 (0.02)	0 (0.01)	-0.01 (0.02)
	IMD 4	5.76 (0.85)	0 (0.01)	0 (0.01)	-0.01 (0.02)
	IMD 3	5.72 (0.76)	-0.01 (0.01)	0 (0.01)	-0.01 (0.02)
	IMD 2	5.7 (0.75)	-0.01 (0.01)	0 (0.01)	-0.01 (0.02)
	IMD 1	5.67 (0.67)	-0.01 (0.01)	0 (0.01)	-0.01 (0.02)

Data is presented as mean (SD) where SD is standard deviation. SD for change in cardiometabolic risk estimated across 200 model runs, each with 10,000 people from the synthetic population. IMD represents Quintiles Index of multiple deprivation (1= Most Deprived, 5= Least Deprived) determined within the Health Survey for England data. Weight is presented by Sex, age group and by IMD quintile. Sex and IMD Quintile outcomes are not divided by age group. All other risk factors are only estimated including the population aged 25 and over as this data is not observed for those under 25 years.

1.6 Long term Health Outcomes

1.6.1 Variability in long term health outcomes across population.

An estimate of variability in population outcomes is presented in Table 1.6.1. The standard deviation outcomes across 200 model runs, each with 10,000 people from the synthetic population are presented in brackets in the form Mean/total (SD).

Table 1.6.1: Variability in lifetime health, costs and labour market outcomes in a no tax scenario across smaller population subsets of 10,000 people across 200 model runs.

	All	Index of Multiple Deprivation Quintiles (1 = most deprived)				
		1	2	3	4	5
**20 Year Health outcomes per 1,000,000**						
Incidence Diabetes	89,812 (2,910)	113,395 (6,336)	94,891 (5,920)	89,355 (6,562)	75,535 (6,584)	68,777 (6,009)
Incidence CVD	126,074 (3,038)	125,916 (6,255)	121,163 (6,316)	131,822 (7,747)	124,606 (7,966)	127,584 (7,605)
Death	253,026 (4,859)	196,438 (7,946)	231,842 (9,955)	270,299 (10,051)	286,293 (10,659)	297,815 (11,515)
**Discounted Lifetime Outcomes for UK population age 5-100 (n = 64,016,711) as scaled from model population outcomes***						
QALYs	998,549,270 (4,982,856)	237,480,546 (2,540,739)	213,335,781 (2,255,983)	187,636,263 (1,880,949)	189,422,816 (2,124,223)	170,673,864 (1,964,474)
NHS Costs (£billion)	1,260.6 (15.9)	321.2 (7.5)	272 (6.5)	237.9 (6.8)	225.2 (6.9)	204.2 (6.6)
Formal Social Care Costs (£billion)	8,488.2 (125.4)	2,545.9 (71.8)	1,967.4 (59.4)	1,578.1 (54.8)	1,282.5 (43.4)	1,114.3 (39.8)
Social Benefit Costs (£billion)	2,615.9 (19.4)	692.3 (8.5)	585.8 (8.5)	473.9 (7.5)	467.8 (8.5)	396.1 (7.8)

This table presents the outcomes estimated for the whole 2,000,000 synthetic population, scaled. Diabetes refers to type 2 diabetes only. Abbreviations: SD; Standard deviation. CVD; cardiovascular disease. QALY; quality adjusted life years. NHS: National Health Service.

1.6.2 Long term health outcomes in probabilistic sensitivity analysis

An additional probabilistic sensitivity analysis of 400 parameter samples in a representative population subset of 100,000 was performed. The results, including mean and credible interval across parameter samples are presented in Table 1.6.3.

Table 1.6.2: Long run outcomes estimated in probabilistic sensitivity analysis.

	Mean QALYs lived (pp)	NHS Lifetime Costs Discounted (£pp)	Social Care Costs (£pp)		Social security Benefit Costs (£pp)	Deaths in first 20 years (per 100,000)	CVD events in first 20 years (per 100,000)	Diabetes diagnosis in first 20 years (per 100,000)	Years Inactive or Unemployed (pp)
			Formal	Informal					
No Tax ("Business As Usual")	15.449 [15.042, 15.795]	23675 [16373, 36503]	165869 [3515, 635982]	259808 [0, 751014]	40803 [39588, 42051]	25692 [24524, 27094]	13542 [8266, 20113]	10827 [5236, 17892]	4.649 [4.436, 4.865]

Tax Design	Change from No Tax Scenario								
Sugar Snacks	0.034 [0.022, 0.048]	-219 [-461, -73]	-750 [-3462, 7]	-1199 [-4352, 0]	-0.31 [-3.63, 2.95]	-31 [-69, -2]	-45 [-182, 61]	-218 [-467, -20]	-0.002 [-0.004, -0.001]
Unhealthy Food	0.053 [0.034, 0.074]	-345 [-722, -105]	-1239 [-5509, 11]	-1977 [-7169, 0]	-0.21 [-4.66, 4.52]	-51 [-114, -3]	-76 [-290, 102]	-367 [-800, -42]	-0.003 [-0.005, -0.002]
Sugar and Salt	0.068 [0.046, 0.094]	-482 [-1033, -159]	-1716 [-7765, 14]	-2762 [-9831, 18]	0.17 [-4.91, 5.43]	-74 [-159, -9]	-116 [-445, 156]	-521[-1092, -72]	-0.004 [-0.006, -0.002]

Results are presented in the format mean 95% credible interval [Lower CrI, Upper CrI], where the mean the credible interval is informed by the probabilistic sensitivity analysis.
Social Care Costs are divided into formal care costs (including care home) and informal costs from the opportunity cost of friends and family of not working due to hours spent caring.
All Costs and QALYS (quality adjusted life years) are discounted annually at 3.5%.
The no tax scenario, representing a scenario in which no change to consumption is assumed.
Abbreviations: pp; per person. NHS; National Health Service. CVD; cardiovascular disease.
Diabetes represents type 2 diabetes only.

1.6.3 Assuming no Discounting in Long Run Modelling

An additional analysis was performed, removing all discounting of costs and QALYs. The results of this analysis are presented in Table 1.6.3.

Table 1.6.3: Lifetime Cost and Quality Adjusted Life Years (QALYs) for a no tax design and the change in outcomes for each of the three food tax policy designs when no discounting is applied.

	Mean QALYs lived (pp)	NHS Lifetime Costs (£pp)	Social Care Costs (£pp)		Social security Benefit Costs (£pp)
			Formal	Informal	
No Tax (“Business As Usual”)	29.64	56,503	425,184	842,459	71,781
**Tax Design**	**Change from No Tax Scenario**				
Sugar Snacks	0.07	- 504	- 1,227	- 2,432	0.60
Unhealthy Food	0.12	- 786	- 2,055	- 4,071	1.73
Sugar and Salt	0.15	- 1,065	- 2,718	- 5,385	3.46

Social Care Costs are divided into formal care costs (including care home) and informal costs from the opportunity cost of friends and family of not working due to hours spent caring.
All Costs and QALYS (quality adjusted life years) are not discounted.
The no tax scenario, representing a scenario in which no change to consumption is assumed Abbreviations: Pp; per person. NHS, National Health Service.

1.7 Industry Response

Reformulation changes nutritional intake by altering the nutritional content of food. This results in a reduction in intake of calories, sugar and salt in the population's baseline consumption before considering the changes as a result of the price increases. Table 1.7.1 presents the mean change in nutritional intake that was estimated to occur if reformulation altered products, but there was no change in consumption. Detail of the methods and data sources used to inform this estimation are provided in supplementary materials section 4.8.

Table 1.7.1: Simulated Mean Change in Daily Nutritional Intake due to Reformulation Alone using the consumer demand model

Policy	Population	Change in Calorie intake due to Reformulation (g)	Change in Sugar intake due to Reformulation (g)	Change in Sodium intake due to Reformulation (mg)
Sugar Snacks	All	-1.21	-1.64	-3.88
	IMD 1	-1.14	-1.66	-3.87
	IMD 2	-1.18	-1.63	-3.85
	IMD 3	-1.22	-1.63	-3.87
	IMD 4	-1.25	-1.62	-3.88
	IMD 5	-1.3	-1.64	-3.92
Unhealthy Food	All	-4.71	-1.85	-16.56
	IMD 1	-4.56	-1.73	-17.52
	IMD 2	-4.65	-1.81	-16.82
	IMD 3	-4.74	-1.89	-16.37
	IMD 4	-4.77	-1.87	-16.1
	IMD 5	-4.86	-1.98	-15.71
Sugar and Salt	All	-16.21	-2.39	-109.31
	IMD 1	-16.8	-2.42	-110.49
	IMD 2	-16.34	-2.39	-109.55
	IMD 3	-16.1	-2.36	-109.5
	IMD 4	-16.04	-2.39	-108.82
	IMD 5	-15.58	-2.39	-107.81

IMD represents Quintiles Index of multiple deprivation (1= Most Deprived, 5= Least Deprived).

1.8 Sensitivity Analyses

A summary of mean daily calorie change and mean monthly food expenditure change for each sensitivity analysis (S1 to S7), and the combinations of these sensitivity analyses is presented in Table 1.8.1. The sensitivity analysis applying an alternative price-elasticities model (S8), is not presented in Table 1.8.1 due to structural differences in this specification preventing it being performed in combination with other sensitivity analyses. The mean change in key dietary outcomes for the base case and for all sensitivity analyses, including S8, are presented separately in Table 1.8.2 to Table 1.8.4 for the Sugar Snacks, Unhealthy Food and Sugar and Salt taxes respectively. Mean calorie and spending changes of the core sensitivity analyses are presented by IMD groups in Table 1.8.5.

Table 1.8.1: Mean change in Calories per day and Expenditure per month across sensitivity analyses for three food tax policy designs

**a) Sugar Snacks**		**Calorie Change (kcal) per day**								**Expenditure Change (£) per month**							
		**Base Case = 25kcal**								**Base Case = -£1.49**							
		I3	S1	S2	S3	S4	S5	S6	S7	I3	S1	S2	S3	S4	S5	S6	S7
Industry Response	I3	-26	-	-	-	-	-	-	-	-1.59	-	-	-	-	-	-	-
Controlling for non-food expenditure	S1	-25	-23	-	-	-	-	-	-	-1.19	-1.00	-	-	-	-	-	-
Removing insignificant PED	S2	-30	-13	-28	-	-	-	-	-	-1.74	0.19	-1.69	-	-	-	-	-
PED by food category only	S3	-26	-23	-28	-24	-	-	-	-	-1.59	-1.00	-1.69	-1.49	-	-	-	-
PED does not differ by income tertiles	S4	-26	-	-19	-25	-25	-	-	-	-1.45	-	-1.09	-1.42	-1.42	-	-	-
Removing substitution effects	S5	-17	-12	-14	-16	-15	-16	-	-	0.76	0.91	0.96	0.76	0.80	0.76	-	-
Individualised consumption	S6	-24	-22	-25	-23	-23	-17	-23	-	-1.19	-0.74	-1.16	-1.09	-1.01	0.73	-1.09	-
Adjust consumption for underreporting	S7	-36	-32	-41	-35	-36	-23	-32	-35	-2.28	-1.34	-2.41	-2.13	-2.03	1.08	-1.55	-2.13

**b) Unhealthy foods**		**Calorie Change (kcal) per day**								**Expenditure Change (£) per month**							
		**Base Case = 42kcal**								**Base Case = -£1.84**							
		I3	S1	S2	S3	S4	S5	S6	S7	I3	S1	S2	S3	S4	S5	S6	S7
Industry Response	I3	-44	-	-	-	-	-	-	-	-1.87	-	-	-	-	-	-	-
Controlling for non-food expenditure	S1	-35	-30	-	-	-	-	-	-	-0.23	0.08	-	-	-	-	-	-
Removing insignificant PED	S2	-48	-20	-46	-	-	-	-	-	-2.12	1.14	-2.13	-	-	-	-	-
PED by food category only	S3	-49	-20	-49	-46	-	-	-	-	-1.87	0.55	-2.13	-1.84	-	-	-	-
PED does not differ by income tertiles	S4	-43	-	-34	-45	-41	-	-	-	-1.44	-	-1.41	-1.47	-1.47	-	-	-
Removing substitution effects	S5	-37	-26	-30	-34	-31	-33	-	-	0.79	1.23	1.03	0.81	0.93	0.81	-	-
Individualised consumption	S6	-44	-31	-44	-44	-40	-34	-42	-	-1.54	0.04	-1.65	-1.48	-1.12	0.78	-1.48	-
Adjust consumption for underreporting	S7	-61	-43	-66	-66	-59	-47	-59	-60	-2.68	0.08	-3.04	-2.64	-2.09	1.16	-2.11	-2.64

**c) Sugar and Salt**		**Calorie Change (kcal) per day**								**Expenditure Change (£) per month**							
		**Base Case = 58kcal**								**Base Case = -£1.15**							
		I3	S1	S2	S3	S4	S5	S6	S7	I3	S1	S2	S3	S4	S5	S6	S7
Industry Response	I3	-70	-	-	-	-	-	-	-	-1.17	-	-	-	-	-	-	-
Controlling for non-food expenditure	S1	-61	-46	-	-	-	-	-	-	0.21	0.54	-	-	-	-	-	-
Removing insignificant PED	S2	-69	-29	-56	-	-	-	-	-	-1.14	2.42	-1.14	-	-	-	-	-
PED by food category only	S3	-72	-23	-56	-59	-	-	-	-	-1.17	1.40	-1.14	-1.15	-	-	-	-

PED does not differ by income tertiles	S4	-68	-	-50	-57	-56	-	-	-	-0.96	-	-1.14	-0.99	-0.99	-	-	-
Removing substitution effects	S5	-57	-34	-41	-43	-41	-44	-	-	1.07	1.47	1.30	1.10	1.19	1.10	-	-
Individualised consumption	S6	-69	-38	-54	-59	-55	-46	-57	-	-0.82	1.05	-0.74	-0.78	-0.58	1.06	-0.78	-
Adjust consumption for underreporting	S7	-93	-67	-80	-84	-80	-62	-82	-82	-1.68	0.66	-1.64	-1.65	-1.41	1.57	-1.12	-1.65

Table 1.8.2: Mean Change in Consumption by Sensitivity Analysis in the Sugar Snacks Tax

SA	Population	Mean Daily Change							
**SA**	**Population**	**Consumption (g)**	**Food Spending (pence)**	**Calorie (kcal)**	**Sugar (g)**	**Sodium (mg)**	**Saturated Fat (g)**	**Fibre (g)**	**F&V (g)**
Base	All	-45.30	-4.91	-24.58	-2.72	-12.87	-0.19	-0.72	-32
	IMD 1	-45.01	-2.13	-21.06	-2.74	-7.03	-0.14	-0.69	-31
	IMD 2	-45.65	-3.9	-23.32	-2.75	-10.65	-0.16	-0.72	-32
	IMD 3	-45.45	-5.45	-25.26	-2.7	-14.03	-0.19	-0.73	-32
	IMD 4	-45.46	-6.19	-26.44	-2.73	-15.76	-0.22	-0.74	-32
	IMD 5	-44.90	-7.8	-27.97	-2.67	-18.8	-0.25	-0.74	-32
I1	All	-46.63	-5.09	-26.48	-4.5	-16.83	-0.19	-0.75	-33
I2	All	-43.75	-5.06	-23.82	-2.66	-12.07	-0.18	-0.7	-31
I3	All	-45.04	-5.24	-25.69	-4.43	-16.02	-0.18	-0.72	-32
S1	All	-52.59	-3.29	-23.01	-3.14	-0.23	-0.14	-0.9	-40
S1S2	All	-39.20	0.64	-13.32	-1.64	1.86	-0.07	-0.66	-28
S1S3	All	-51.17	-3.29	-22.83	-2.85	-4.99	-0.02	-0.9	-40
S1S5	All	-3.48	3	-11.58	-1.12	-3.49	-0.29	-0.08	0
S1S6	All	-41.20	-2.42	-21.59	-2.8	-2.71	-0.16	-0.73	-32
S1S7	All	-74.37	-4.41	-32.01	-4.45	0.68	-0.19	-1.28	-57
S2	All	-48.66	-5.55	-28.46	-2.64	-20.14	-0.24	-0.76	-32
S2S3	All	-46.75	-5.55	-28.42	-2.37	-24.83	-0.12	-0.75	-32
S2S4	All	-46.97	-3.59	-19.24	-2.18	-11.97	-0.04	-0.73	-33
S2S5	All	-3.38	3.16	-13.66	-1.44	-3.39	-0.33	-0.08	0
S2S6	All	-37.46	-3.82	-25.28	-2.41	-16.53	-0.21	-0.62	-26
S2S7	All	-69.51	-7.93	-40.65	-3.76	-28.77	-0.34	-1.08	-46
S3	All	-43.87	-4.91	-24.41	-2.43	-17.53	-0.07	-0.72	-32
S3S4	All	-44.56	-4.68	-24.79	-2.4	-17.73	-0.06	-0.74	-33
S3S5	All	-6.45	2.49	-16.37	-1.51	-9.29	-0.29	-0.09	0
S3S6	All	-35.87	-3.58	-22.53	-2.3	-14.51	-0.12	-0.6	-26
S3S7	All	-62.66	-7.02	-34.87	-3.48	-25.05	-0.1	-1.03	-45
S4	All	-45.99	-4.68	-24.97	-2.69	-13.03	-0.18	-0.75	-33
S4S5	All	-3.86	2.64	-15.24	-1.53	-4.59	-0.36	-0.09	0
S4S6	All	-35.85	-3.32	-22.54	-2.47	-10.86	-0.17	-0.61	-27
S4S7	All	-65.71	-6.69	-35.66	-3.84	-18.61	-0.25	-1.07	-47
S5	All	-4.08	2.49	-16.32	-1.61	-5.24	-0.38	-0.1	0
S5S6	All	-4.24	2.41	-16.54	-1.67	-5.68	-0.37	-0.09	0

SA	Population	Mean Daily Change							
S5S7	All	-5.83	3.56	-23.32	-2.31	-7.49	-0.54	-0.14	0
S6	All	-35.33	-3.58	-22.56	-2.52	-10.99	-0.18	-0.6	-26
S6S7	All	-50.47	-5.11	-32.23	-3.61	-15.69	-0.26	-0.85	-37
S7	All	-64.71	-7.02	-35.12	-3.89	-18.39	-0.27	-1.03	-45
S8	All	-18.07	-3.43	-41.31	-3.03	-28.73	-0.76	-0.34	-4
I1S1	All	-55.26	-3.7	-25.17	-4.93	-7.39	-0.15	-0.94	-42
I2S1	All	-52.08	-3.77	-23.14	-3.1	-3.98	-0.15	-0.89	-39
I3S1	All	-53.61	-3.9	-24.97	-4.88	-7.76	-0.15	-0.92	-41
I3S2	All	-48.02	-5.73	-29.66	-4.32	-23.43	-0.23	-0.76	-32
I3S3	All	-43.93	-5.24	-25.53	-4.07	-20.97	-0.07	-0.72	-32
I3S4	All	-45.45	-4.76	-25.83	-4.38	-16.06	-0.17	-0.75	-33
I3S5	All	-3.93	2.49	-17.26	-3.3	-8.72	-0.37	-0.09	0
I3S6	All	-35.58	-3.92	-23.74	-4.21	-14.63	-0.18	-0.6	-26
I3S7	All	-64.34	-7.48	-36.18	-5.62	-21.23	-0.26	-1.03	-46

IMD represents Quintiles Index of multiple deprivation (1= Most Deprived, 5= Least Deprived).
F&V represents fruit and vegetables.

Table 1.8.3: Mean Change in Consumption by Sensitivity Analysis in the Unhealthy Food Tax

SA	Population	Mean Daily Change							
SA	Population	Consumption (g)	Food Spending (pence)	Calorie (kcal)	Sugar (g)	Sodium (mg)	Saturated Fat (g)	Fibre (g)	F&V (g)
Base	All	-55.47	-6.07	-42.35	-3.09	-37.49	-0.33	-1.04	-42
	IMD 1	-52.72	-2.18	-37.44	-2.87	-31.66	-0.22	-0.99	-40
	IMD 2	-54.95	-4.67	-40.65	-3.04	-35.39	-0.29	-1.03	-42
	IMD 3	-56.16	-6.8	-43.34	-3.12	-38.62	-0.35	-1.05	-43
	IMD 4	-56.88	-7.81	-44.88	-3.2	-40.47	-0.39	-1.06	-43
	IMD 5	-57.44	-10.16	-47	-3.27	-43.21	-0.45	-1.07	-45
I1	All	-55.47	-6.07	-47.21	-4.97	-54.42	-0.34	-1.03	-42
I2	All	-54.14	-6.16	-39.57	-3.06	-33.35	-0.3	-0.99	-41
I3	All	-54.14	-6.16	-44.41	-4.93	-50.23	-0.31	-0.99	-41
S1	All	-59.25	0.27	-30.06	-2.92	-9.17	-0.08	-1.2	-51
S1S2	All	-44.43	3.74	-20.36	-1.87	-5.91	-0.02	-0.85	-33
S1S3	All	-57.66	1.8	-20.39	-3.35	3.92	0.16	-1.1	-52
S1S5	All	-8.44	4.05	-26.04	-0.85	-25.04	-0.5	-0.2	-1
S1S6	All	-52.47	0.14	-31.42	-2.96	-13.24	-0.16	-1.02	-42
S1S7	All	-85.06	0.25	-43.4	-4.19	-13.65	-0.13	-1.72	-74
S2	All	-61.42	-7.01	-46.22	-3.6	-42.49	-0.37	-1.05	-43
S2S3	All	-63.79	-7.01	-48.83	-3.71	-46.52	-0.21	-1.15	-43
S2S4	All	-59.34	-4.64	-34.16	-2.96	-32.81	-0.13	-0.99	-43
S2S5	All	-8.73	3.4	-29.98	-1.69	-23.6	-0.6	-0.18	0
S2S6	All	-52.89	-5.42	-43.87	-3.13	-39.85	-0.39	-0.92	-36

SA	Population	Mean Daily Change							
S2S7	All	-87.74	-10.01	-66.02	-5.14	-60.71	-0.52	-1.5	-61
S3	All	-59.25	-6.07	-46.09	-3.76	-40.57	-0.17	-1.13	-43
S3S4	All	-59.89	-4.82	-44.9	-3.68	-39.01	-0.15	-1.13	-43
S3S5	All	-13.28	2.68	-34.48	-2.78	-24.28	-0.52	-0.25	0
S3S6	All	-51.34	-4.86	-43.84	-3.49	-36.92	-0.27	-0.96	-35
S3S7	All	-84.64	-8.66	-65.84	-5.37	-57.95	-0.24	-1.61	-61
S4	All	-56.10	-4.82	-41.13	-3	-35.92	-0.32	-1.04	-43
S4S5	All	-8.69	3.06	-31	-1.73	-24.13	-0.63	-0.19	0
S4S6	All	-49.52	-3.68	-40.11	-3.04	-33.2	-0.34	-0.9	-36
S4S7	All	-80.14	-6.88	-58.76	-4.29	-51.32	-0.45	-1.49	-62
S5	All	-9.43	2.68	-33.24	-1.91	-25.68	-0.66	-0.21	0
S5S6	All	-9.86	2.58	-33.99	-2.15	-25.14	-0.67	-0.21	0
S5S7	All	-13.48	3.82	-47.49	-2.73	-36.69	-0.95	-0.29	0
S6	All	-49.10	-4.86	-41.53	-3.14	-34.88	-0.36	-0.9	-35
S6S7	All	-70.14	-6.95	-59.33	-4.48	-49.82	-0.52	-1.28	-50
S7	All	-79.25	-8.66	-60.5	-4.41	-53.56	-0.48	-1.48	-61
S8	All	-21.37	-4.58	-66.87	-4	-53.37	-1.15	-0.47	-2
I1S1	All	-60.85	-0.14	-35.4	-4.81	-31.23	-0.1	-1.21	-51
I2S1	All	-60.26	-0.77	-30.38	-2.97	-14.05	-0.09	-1.18	-50
I3S1	All	-60.26	-0.77	-35.26	-4.85	-31.1	-0.1	-1.18	-50
I3S2	All	-59.13	-6.96	-48.31	-5.23	-56.04	-0.34	-1.01	-42
I3S3	All	-57.44	-6.16	-48.66	-5.46	-54.61	-0.16	-1.08	-41
I3S4	All	-54.43	-4.73	-43.24	-4.82	-49.15	-0.29	-1	-42
I3S5	All	-8.91	2.58	-36.59	-3.72	-41.08	-0.63	-0.2	0
I3S6	All	-47.68	-5.05	-44.39	-4.93	-48.97	-0.34	-0.86	-34
I3S7	All	-77.35	-8.8	-61.43	-6.26	-64.65	-0.44	-1.41	-59

IMD represents Quintiles Index of multiple deprivation (1= Most Deprived, 5= Least Deprived).
F&V represents fruit and vegetables.

Table 1.8.4: Mean Change in Consumption by Sensitivity Analysis in the Sugar and Salt Tax

SA	Population	Mean Daily Change							
SA	Population	Consumption (g)	Food Spending (pence)	Calorie (kcal)	Sugar (g)	Sodium (mg)	Saturated Fat (g)	Fibre (g)	F&V (g)
Base	All	-36.67	-3.78	-57.52	-4.97	-42.61	-0.51	-1.03	-33
	IMD 1	-33.71	-1.1	-57.15	-5.15	-41.47	-0.47	-1.03	-32
	IMD 2	-36.29	-2.79	-58.06	-5.08	-42.74	-0.49	-1.05	-33
	IMD 3	-37.60	-4.29	-58.03	-4.98	-42.95	-0.51	-1.04	-34
	IMD 4	-38.05	-4.98	-57.42	-4.88	-42.82	-0.52	-1.02	-34
	IMD 5	-38.46	-6.62	-56.89	-4.67	-43.34	-0.55	-1.01	-34
I1	All	-36.64	-3.78	-73.67	-7.35	-151.83	-0.51	-1.03	-33
I2	All	-37.06	-3.85	-53.61	-4.6	-40.24	-0.46	-0.98	-32

SA	Population	Mean Daily Change							
I3	All	-37.04	-3.85	-69.77	-6.99	-149.48	-0.46	-0.98	-32
S1	All	-40.62	1.77	-46.45	-4.58	-14.27	-0.26	-1.22	-42
S1S2	All	-21.01	7.97	-29.22	-3.35	-1.22	-0.05	-0.84	-25
S1S3	All	-44.01	4.61	-22.82	-3.19	-8.12	0.04	-0.98	-44
S1S5	All	-14.33	4.83	-33.88	-1.99	-34.45	-0.46	-0.36	0
S1S6	All	-39.82	3.44	-37.96	-3.48	-17.66	-0.12	-1.09	-37
S1S7	All	-59.13	2.17	-67.43	-6.6	-21.67	-0.38	-1.75	-60
S2	All	-40.64	-3.76	-55.8	-5.27	-41.86	-0.52	-0.97	-33
S2S3	All	-51.62	-3.76	-56.27	-3.98	-61.61	-0.35	-1.09	-35
S2S4	All	-41.03	-3.73	-49.96	-4.85	-38.52	-0.39	-0.94	-33
S2S5	All	-16.43	4.28	-41.27	-2.92	-37.64	-0.56	-0.39	0
S2S6	All	-41.25	-2.42	-53.64	-4.21	-50.85	-0.41	-0.99	-31
S2S7	All	-58.16	-5.39	-79.76	-7.53	-59.86	-0.74	-1.38	-48
S3	All	-49.68	-3.78	-58.91	-4.1	-62.73	-0.33	-1.15	-35
S3S4	All	-50.77	-3.24	-57.36	-3.92	-62.05	-0.32	-1.15	-36
S3S5	All	-21.14	3.61	-43.41	-3.08	-40.51	-0.53	-0.41	-2
S3S6	All	-44.85	-2.57	-59.1	-4.21	-63.17	-0.32	-1.12	-31
S3S7	All	-71.07	-5.42	-84.21	-5.87	-89.66	-0.47	-1.64	-50
S4	All	-37.66	-3.24	-56.12	-4.8	-41.87	-0.5	-1.03	-34
S4S5	All	-16.29	3.92	-41.14	-2.9	-37.6	-0.57	-0.39	0
S4S6	All	-40.20	-1.89	-55.24	-4.08	-52.17	-0.39	-1.04	-31
S4S7	All	-53.89	-4.65	-80.21	-6.85	-59.85	-0.71	-1.47	-49
S5	All	-17.16	3.61	-43.66	-3.06	-39.47	-0.6	-0.41	0
S5S6	All	-18.69	3.5	-46.21	-3.35	-40.94	-0.6	-0.46	-1
S5S7	All	-24.51	5.16	-62.36	-4.37	-56.37	-0.86	-0.59	0
S6	All	-39.10	-2.57	-57.07	-4.26	-53.34	-0.4	-1.05	-31
S6S7	All	-55.92	-3.68	-81.54	-6.08	-76.22	-0.56	-1.51	-44
S7	All	-52.47	-5.42	-82.21	-7.1	-60.91	-0.73	-1.47	-48
S8	All	-31.00	-1.08	-71.42	-4.67	-70.06	-0.89	-0.76	-5
I1S1	All	-42.17	1.36	-63.02	-6.97	-128.83	-0.26	-1.23	-42
I2S1	All	-43.11	0.68	-44.52	-4.31	-20.03	-0.25	-1.18	-40
I3S1	All	-43.07	0.68	-60.66	-6.7	-129.25	-0.25	-1.18	-40
I3S2	All	-40.04	-3.74	-68.51	-7.14	-149.54	-0.47	-0.93	-32
I3S3	All	-47.72	-3.85	-71.69	-6.21	-168.06	-0.31	-1.09	-33
I3S4	All	-37.75	-3.17	-68.44	-6.83	-148.76	-0.45	-0.98	-33
I3S5	All	-15.79	3.52	-56.62	-5.18	-145.36	-0.56	-0.38	0
I3S6	All	-37.95	-2.71	-69.46	-6.37	-158.2	-0.37	-0.99	-29
I3S7	All	-53.01	-5.52	-92.76	-8.96	-166.74	-0.66	-1.4	-46

IMD represents Quintiles Index of multiple deprivation (1= Most Deprived, 5= Least Deprived).
F&V represents fruit and vegetables.

Table 1.8.5: Mean change in calories and food spending for core sensitivity analyses by IMD population groups.

		Baseline Calorie Intake (kcal) per day					Baseline Food Expenditure (£) per month				
		IMD 1	IMD 2	IMD 3	IMD 4	IMD 5	IMD 1	IMD 2	IMD 3	IMD 4	IMD 5
		1760	1766	1775	1785	1770	£148.68	£157.56	£165.66	£170.60	£178.53

a) Sugar Snacks		Calorie Change (kcal) per day					Expenditure Change (£) per month				
		IMD 1	IMD 2	IMD 3	IMD 4	IMD 5	IMD 1	IMD 2	IMD 3	IMD 4	IMD 5
Base Case		-21.1	-23.3	-25.2	-26.4	-28.0	-0.65	-1.19	-1.66	-1.88	-2.38
Industry Response - Reformulation	I1	-22.7	-25.1	-27.2	-28.4	-30.1	-0.67	-1.23	-1.72	-1.95	-2.46
Industry Response - Pass Through	I2	-19.4	-22.2	-24.6	-26.1	-28.3	-0.68	-1.22	-1.71	-1.93	-2.45
Industry Response - Reformulation and Pass Through	I3	-21.1	-23.9	-26.5	-28.1	-30.4	-0.70	-1.26	-1.76	-2.00	-2.53
Controlling for non-food expenditure	S1	-23.2	-23.2	-22.9	-23.1	-22.7	-0.68	-0.90	-1.05	-1.13	-1.34
Removing insignificant PED	S2	-29.1	-28.9	-28.2	-28.2	-27.7	-1.53	-1.66	-1.70	-1.73	-1.86
PED by food category only	S3	-21.1	-23.2	-25.0	-26.2	-27.6	-0.65	-1.19	-1.66	-1.88	-2.38
PED does not differ by income tertiles	S4	-25.8	-25.4	-24.8	-24.8	-23.9	-1.33	-1.42	-1.44	-1.46	-1.49
Removing substitution effects	S5	-17.1	-16.6	-16.2	-16.1	-15.4	0.75	0.75	0.75	0.78	0.77
Individualised consumption	S6	-19.7	-21.5	-23.0	-24.2	-25.6	-0.40	-0.83	-1.21	-1.42	-1.89
Adjust consumption for underreporting	S7	-30.1	-33.3	-36.1	-37.8	-40.0	-0.93	-1.69	-2.37	-2.69	-3.39
Alternative PED	S8	-46.0	-40.5	-40.2	-41.3	-48.2	-0.97	-1.21	-1.06	-0.83	-0.93

b) Unhealthy foods		Calorie Change (kcal) per day					Expenditure Change (£) per month				
		IMD 1	IMD 2	IMD 3	IMD 4	IMD 5	IMD 1	IMD 2	IMD 3	IMD 4	IMD 5
Base Case		-37.4	-40.6	-43.3	-44.9	-47.0	-0.66	-1.42	-2.07	-2.37	-3.09
Industry Response - Reformulation	I1	-42.2	-45.4	-48.2	-49.8	-52.0	-0.66	-1.42	-2.07	-2.37	-3.09
Industry Response - Pass Through	I2	-32.8	-37.0	-40.8	-43.0	-46.3	-0.68	-1.44	-2.10	-2.41	-3.15
Industry Response - Reformulation and Pass Through	I3	-37.6	-41.8	-45.7	-47.9	-51.3	-0.68	-1.44	-2.10	-2.41	-3.15
Controlling for non-food expenditure	S1	-28.7	-29.5	-30.2	-31.0	-31.3	0.44	0.19	0.03	-0.06	-0.30
Removing insignificant PED	S2	-45.5	-46.1	-46.1	-46.6	-47.0	-1.78	-2.02	-2.16	-2.25	-2.57
PED by food category only	S3	-41.4	-44.4	-47.0	-48.6	-50.5	-0.66	-1.42	-2.07	-2.37	-3.09
PED does not differ by income tertiles	S4	-40.2	-40.9	-41.3	-41.8	-41.7	-1.36	-1.46	-1.50	-1.50	-1.54
Removing substitution effects	S5	-33.4	-33.3	-33.1	-33.4	-33.0	0.75	0.79	0.82	0.85	0.88
Individualised consumption	S6	-36.3	-40.0	-42.5	-44.5	-46.4	-0.45	-1.12	-1.65	-1.96	-2.62
Adjust consumption for underreporting	S7	-53.5	-58.0	-61.9	-64.1	-67.1	-0.95	-2.03	-2.95	-3.39	-4.42
Alternative PED	S8	-77.3	-64.9	-65.4	-65.1	-80.1	-1.50	-1.34	-1.41	-1.24	-1.57

c)Sugar and Salt		Calorie Change (kcal) per day					Expenditure Change (£) per month				
		IMD 1	IMD 2	IMD 3	IMD 4	IMD 5	IMD 1	IMD 2	IMD 3	IMD 4	IMD 5
Base Case		-57.2	-58.1	-58.0	-57.4	-56.9	-0.34	-0.85	-1.30	-1.51	-2.02
Industry Response - Reformulation	I1	-73.9	-74.4	-74.1	-73.4	-72.4	-0.34	-0.85	-1.30	-1.51	-2.01

Industry Response - Pass Through	I2	-51.8	-53.4	-54.2	-54.2	-54.9	-0.33	-0.85	-1.33	-1.55	-2.08
Industry Response - Reformulation and Pass Through	I3	-68.5	-69.7	-70.3	-70.2	-70.5	-0.33	-0.85	-1.33	-1.55	-2.07
Controlling for non-food expenditure	S1	-47.3	-47.1	-46.4	-46.2	-44.8	0.89	0.64	0.49	0.39	0.16
Removing insignificant PED	S2	-56.6	-56.8	-55.9	-54.9	-54.4	-0.76	-1.01	-1.19	-1.28	-1.61
PED by food category only	S3	-58.7	-59.3	-59.3	-59.0	-58.3	-0.34	-0.85	-1.30	-1.51	-2.02
PED does not differ by income tertiles	S4	-56.9	-57.1	-56.4	-55.5	-54.3	-0.94	-1.01	-1.01	-0.99	-0.99
Removing substitution effects	S5	-45.6	-44.7	-43.5	-42.7	-41.2	1.04	1.07	1.10	1.14	1.16
Individualised consumption	S6	-56.8	-57.5	-57.3	-57.8	-56.6	-0.06	-0.49	-0.89	-1.13	-1.63
Adjust consumption for underreporting	S7	-81.7	-83.0	-82.9	-82.1	-81.3	-0.49	-1.21	-1.87	-2.17	-2.89
Alternative PED	S8	-73.9	-78.7	-69.3	-67.2	-74.9	-0.23	-0.53	-0.33	-0.21	-0.19

IMD represents Quintiles Index of multiple deprivation (1= Most Deprived, 5= Least Deprived).

Colour gradients in calories are presented separately for each sensitivity analysis with darker green representing a larger decline in calories. Colour gradients in food spending is presented separately for each sensitivity analysis from negative in blue to positive in red.

2 The Food Tax Policies

2.1 Design 1: Sugar Snacks Tax

This policy extends the sugar drinks industry levy (SDIL) to affect non-essential sugary snack food and milk drink consumption. The policy applies £4 per kg of sugar to confectionery, cakes and biscuits with 18 grams or more sugar per 100g, including those currently exempt from VAT. A threshold of 18g/100g for the sugary snacks tax was selected to correspond with the 'Total Sugar' 4-point allocation within the Nutrient Profiling Model scoring system, a benchmark explicitly recommended by stakeholders. Milk drinks would be taxed according to the original SDIL rates (pre 2025 changes) (3). This policy would adopt the food categories for confectionery (category 4), cakes (category 6) and biscuits (category 7) defined in schedule 1 of the Food (Promotion and Placement) (England) Regulations 2021 (4).

The Food (Promotion and Placement) (England) Regulations 2021 - Schedule 1

- Category 4: Confectionery including chocolates and sweets.
- Category 6: Cakes and cupcakes.
- Category 7: Sweet biscuits and bars based on one or more of nuts, seeds or cereal.

A drink is liable for the Soft Drinks Industry Levy if it meets all of the following conditions:

- it has had sugar added during production, or anything (other than fruit juice, vegetable juice and milk) that contains sugar, such as honey
- it contains at least 5 grams (g) of sugar per 100 millilitres (ml) in its ready to drink or diluted form
- it's either ready to drink, or to be drunk it must be diluted with water, mixed with crushed ice or processed to make crushed ice, mixed with carbon dioxide, or a combination of these
- it's bottled, canned or otherwise packaged so it's ready to drink or be diluted
- it has a content of 1.2% alcohol by volume (ABV) or less

In this tax design Milk drinks will be eligible for the tax if it contains 5 grams (g) of sugar per 100 millilitres (ml) in its ready to drink or diluted form. Drink powders will be included in the policy and will be assessed according to their made-up nutrition. In contrast to the existing SDIL it will include drinks served in an open cup.

In the sugar reformulation programme, the following categories are included as milk-based drinks:

- Pre-packaged milk-based drinks
- Coffee and tea powders, syrups and pods
- Hot chocolate and malt powders
- Pre-packaged fermented (yoghurt) drinks
- Open cup hot or cold milk-based drinks
- Pre-packed milk substitute drinks

2.2 Design 2: Unhealthy food tax

HFSS foods are identified using the Nutrient Profile Model (Version 1). The policy is designed to extend/complement the postponed advertising and promotions policies (2021). Within Schedule 1 categories, with some exceptions, any produce defined as less healthy (Nutrient Profile Score <4 for food and 1 for drinks). A tax of 20% is applied to these products.

Draft legislation The Food (Promotion and Placement) (England) Regulations 2021: Schedule 1

Category 1: Prepared soft drinks containing added sugar ingredients (other than the exempt soft drinks listed in paragraph 4(1)).

The following provisions apply for the purposes of this category.

1.

1. "Soft drink" means—
   - (a) a beverage of an alcoholic strength not exceeding 1.2%, or
   - (b) a liquid or a powder which, when prepared in a specified manner, constitutes a beverage of an alcoholic strength not exceeding 1.2%.
2. A liquid or a powder is prepared in a specified manner if it is—
   - (a) diluted,
   - (b) combined with crushed ice, or processed so as to create crushed ice,
   - (c) combined with carbon dioxide, or
   - (d) prepared by way of a process that involves any combination of the processes mentioned in paragraphs (a) to (c).

2.

1. A soft drink is "prepared" if it is—
   - (a) a soft drink within paragraph 1(1)(a), or
   - (b) a beverage that would result from preparing a liquid or a powder within paragraph 1 (1)(b)— in a specified manner and in accordance with the relevant dilution ratio.
2. The "relevant dilution ratio" means—
   - (a) the dilution ratio stated on, or calculated by reference to information stated on, the packaging of the soft drink, or
   - (b) where no such dilution ratio or information is stated, the dilution ratio of similar drinks on the market.

3.

1. A soft drink contains "added sugar ingredients" if any of the following are combined with other ingredients at any stage in the production of the soft drink—
   - (a) calorific mono-saccharides or di-saccharides;
   - (b) a substance containing calorific mono-saccharides or di-saccharides.
2. But a soft drink does not contain "added sugar ingredients" only by reason of containing fruit juice, vegetable juice or milk (or any combination of them).
3. For the purposes of sub-paragraph (2)—
   - (a) "fruit juice" is to be construed in accordance with regulation 5 (sugar content condition: fruit juice) of the Soft Drinks Industry Levy Regulations 2018(1) ("the SDIL Regulations");
   - (b) "vegetable juice" is to be construed in accordance with regulation 6 (sugar content condition: vegetable juice) of the SDIL Regulations;
   - (c) "milk" is to be construed in accordance with regulation 7 (sugar content condition and exempt soft drinks: milk and milk-based drinks) of the SDIL Regulations.

4.

1. The following are “exempt soft drinks”—
    (a) alcohol substitute drinks which meet specified conditions, and
    (b) soft drinks of a specified description which are for use for medicinal or other specified purposes.
2. For the purposes of sub-paragraph (1)(a), the specified conditions are—
    (a) condition 1 provided for by paragraph (2) of regulation 9 (exempt soft drinks: alcohol substitute drinks) of the SDIL Regulations, and
    (b) one or more of conditions 2, 3 and 4 provided for by paragraphs (3) to (5) of regulation 9.
3. For the purposes of sub-paragraph (1)(b)—
    (a) the specified purposes are the purposes provided for by paragraph (1) of regulation 10 (exempt soft drinks: for medicinal or other purposes) of the SDIL Regulations, and
    (b) the specified descriptions are the descriptions provided for in paragraphs (2) and (3) of regulation 10.

Category 2: Any of the following:

1. Savoury snacks whether intended to be consumed alone or as part of a complete meal including—
    a. products made from potato, other vegetables, grain or pulses;
    b. extruded, sheeted and pelleted products;
    c. bagged savoury crackers, rice cakes or biscuits, such as crisps, pitta bread based snacks, pretzels, poppadums, salted popcorn and prawn crackers (but not raw, roasted, coated or flavoured nuts).
2. Pork rind-based snacks whether intended to be consumed alone or as part of a complete meal.

Category 3: Breakfast cereals including ready-to-eat cereals, granola, muesli, porridge oats and other oat-based cereals.

Category 4: Confectionery including chocolates and sweets.

Category 5: Ice cream, ice lollies, frozen yogurt, water ices and similar frozen products.

Category 6: Cakes and cupcakes.

Category 7: Sweet biscuits and bars based on one or more of nuts, seeds or cereal.

Category 8: Morning goods, including croissants, pains au chocolat and similar pastries, crumpets, pancakes, buns, teacakes, scones, waffles, Danish pastries and fruit loaves.

Category 9: Desserts and puddings, including pies, tarts and flans, cheesecake, gateaux, dairy desserts, sponge puddings, rice pudding, crumbles, fruit fillings, powdered desserts, custards, jellies and meringues.

Category 10: Sweetened (whether with sugar or otherwise) yoghurt and fromage frais.

Category 11: Pizza (except plain pizza bases).

Category 12: Roast potatoes, potato and sweet potato chips, fries and wedges, potato waffles, novelty potato shapes (such as smiley faces), hash browns, rostis, crispy potato slices, potato croquettes.

Category 13: Any of the following:

1. Products that are marketed as ready for cooking or reheating without requiring further preparation and intended to be consumed as a complete meal.
2. Products, other than products that contain pastry, in or with a sauce (but not a marinade, glaze, dressing, seasoning or similar accompaniment) that are marketed as ready for cooking or reheating without requiring further preparation and intended to be consumed as the main element of a meal.

3. Breaded or battered—
    (a) vegetable, fish, shellfish, meat, or poultry products;
    (b) substitute fish, shellfish, meat or poultry products, including fish fingers, fish cakes, chicken nuggets and breaded meat substitute.

2.3 Design 3: Sugar and Salt

The National Food Strategy proposed a tax to be applied at a rate of £3/kg of added sugar and £6/kg of salt on all products. This proposal has been modified to include only retail and out of home products and food categories used by Government for restricting advertising and promotions and in the voluntary reformulation programme (salt, sugar and calories) (4, 5), presented in Table 2.3.1. Targeting foods for inclusion in the levy helps to focus the policy on less nutrition foods, and less likely to have unintended consequences on core foods.

Table 2.3.1: Products covered by existing legislation and voluntary programmes

	**Promotions and advertising**	**Salt target *(6)***	**Sugar target *(7)***	**Calorie target *(8)***
Bacon		X		
Ham/other cured meat		X		
Sausages		X		
Meat pies		X		X
Cooked uncured meat (included roast meat)		X		
Burgers and grill steaks		X		
Frankfurters, hotdogs and burgers		X		
Breaded and battered products	X			X
Bread and rolls		X		
Bread and rolls with additions		X		X
Garlic/cheesy bread				X
Morning goods – yeast raised	X	X	X	
Morning goods -powder raised	X	X	X	
Breakfast cereal	X	X	X	
Cheddar and similar hard cheese		X		
Fresh cheeses		X		
Mozzerella		X		
Blue cheese		X		
Processed cheese		X		
Salted butters and buttery spreads		X		
Lightly salted butter		X		
Margarine /other spreads		X		
Baked beans		X		
Baked beans and canned pasta		X		
Ready meals and meal centres	X	X		X
Soups		X		
Pizza	X	X		X
Standard potato crisp	X	X		X
Extruded and sheeted snacks	X	X		X
Pelleted snacks	X	X		X
Salt and vinegar products	X	X		X
Salty popcorn	X	X		X
Sweet/salty popcorn		X		
Flavoured nuts		X		
Pork rind products	X			
Cakes	X	X	X	
Pastries		X	X	
Sweet pies and other pastry based desserts	X	X	X	
Yoghurt and fromage frais	X		X	
Sweet spreads and sauces			X	
Sandwiches		X		X
Tomato ketchup		X		
Brown sauce		X		
Salad cream		X		
Mayonnaise		X		
Salad dressing		X		

Cook in pasta sauces		X		X
Pesto and other thick sauces		X		X
Thick pastes		X		X
Sweet confectionery	X		X	
Chocolate confectionery	X		X	
Sweet biscuits	X	X	X	
Savoury biscuits		X		X
Ice-cream	X			
Lollies and sorbets	X		X	
Fresh, canned and frozen pasta and noodles		X		
Rice and cous cous dried or cooked		X		
Other cereal including ready made pastry, Yorkshire puddings, dumplings, crumble, taco shells, flan, tempura, and pizza bases.		X		
Dessert mises as consumed	X	X	X	
Cheesecake	X	X	X	
Sponge-based processed puddings	X	X	X	
All other processed puddings	X	X	X	
Quiche		X		
Scotch eggs		X		
Canned fish		X		
Canned vegetables		X		
Plain meat alternatives		X		
Meat free products		X		
Meat-free bacon		X		
Dehydrated instant mashed potato	X	X		X
Other processed potato products	X	X		X
Dried beverages		X		
Stocks		X		
Gravy		X		
Childrens meal bundles				X
Takeaway starter, side, small plate				X
Takeaway mains				
Milk drinks with added sugar	X			
Juices with added sugar	X			
Prepared soft drink	X			

3 Synthetic Population

3.1 Overview

The model required dietary, demographic, anthropometric and metabolic characteristics that would be representative of the UK general population. To provide this breadth of information, multiple sources of data are utilised. Demographic, anthropometric and metabolic characteristics are informed from Health Survey for England data. Information about dietary patterns are provided by the National Diet and Nutrition Survey. Finally, to represent the age, sex and socioeconomic classification of the UK, census data is used to reweight the data sample.

3.1.1 Key assumptions

Key assumptions:

- **Temporal changes in sociodemographic:** It assumes that the sociodemographic characteristics of the UK have not changed between the 2021 census and the model base year (2022).
- **Temporal changes in health and consumption:** It assumes that there are no changes in health status and consumption over the waves of the sample datasets.
- **Cross-Nation Health Equivalency:** Assumes that anthropometric and metabolic characteristics within specific age, sex, and socioeconomic groups are consistent between England and the other devolved nations (Scotland, Wales, and Northern Ireland), meaning any minor regional differences are unlikely to significantly bias the overall UK-wide findings.

3.2 Baseline Population Characteristics: Health Survey for England

The Heath Survey for England (HSE) was chosen because they collect detailed cross-sectional data on the characteristics of all ages of the English and UK populations. It also benefits from being a reasonably good representation across sociodemographic profiles. A major advantage of the HSE dataset is that includes important clinical risk factors such as HbA1c, SBP, and cholesterol and health related quality of life (HSE 2018 only). The characteristics of individuals included in the cost-effectiveness model were based sampled from the HSE 2018 and HSE 2019 datasets (9, 10). Alternative surveys from the devolved nations were considered for inclusion into the model to be merged with the England data. However, given that a process to merge these two datasets was needed, incorporating additional datasets would add complexity to these newly developed processes. The baseline population based on the HSE can be merged with the National Diet and Nutrition Survey to create a synthetic population with dietary information. The data were obtained from the UK Data Service https://ukdataservice.ac.uk/. Re-weighting the Health Survey for England data by age, sex and socioeconomic classification to the UK captures variations in the health profiles of the devolved nations by social groups. However, this assumes the anthropometric and metabolic characteristics in England are similar, within age, sex and socioeconomic classification groups, to that in Scotland, Wales and Northern Ireland. There may be some systemic differences in health profile, particularly observed differences in obesity prevalence, that would lead to bias.

3.2.1 Exclusion Criteria

The total sample size of the HSE 2018 and HSE 2019 were 10,250 and 10,299 respectively. Individuals who were younger than 5 years (N=1,271) were excluded from the sample. This left a final sample size of 19,278 individuals. Summary statistics for the data extracted and combined from the HSE 2018 and HSE 2019 data are reported in Table 3.2.1.

Table 3.2.1: Summary statistics of sample from HSE 2018 and HSE 2019 (N=20,549)

Variable		Unit	Mean or N	SD or %
		N	20549	
Sex	Female	N (%)	11116	54%
	Male	N (%)	9433	46%
Ethnicity	White	N (%)	17290	84%
	Asian	N (%)	1837	9%
	Black	N (%)	690	3%
	Other	N (%)	732	4%
Index of Multiple Deprivation	1	N (%)	3994	19%
	2	N (%)	4036	20%
	3	N (%)	4050	20%
	4	N (%)	4240	21%
	5	N (%)	4229	21%
Age	Age 5 to 7	N (%)	820	4%
	Age 8 to 10	N (%)	825	4%
	Age 11 to 12	N (%)	545	3%
	Age 13 to 15	N (%)	706	3%
	Age 16 to 19	N (%)	689	3%
	Age 20 to 39	N (%)	4411	21%
	Age 40 to 69	N (%)	8102	39%
	Age 70 or above	N (%)	3180	15%
National statistics socio-economic classification	NS-SEC1	N (%)	8633	42%
	NS-SEC2	N (%)	2189	11%
	NS-SEC3	N (%)	2330	11%
	NS-SEC4	N (%)	1641	8%
	NS-SEC5	N (%)	5082	25%
	NS-SEC99	N (%)	462	2%
Smoking Status	Current Smoker	N (%)	2570	13%
Weight		Mean (SD)	69.90	25.88
		Missing (%)	3661	18%
Height		Mean (SD)	1.62	0.18
		Missing (%)	3901	19%
Body mass index (BMI) kg/m^2		Mean (SD)	26.17	6.54
		Missing (%)	4369	21%
BMI Classification	Underweight	N (%)	609	3%
	Healthy Weight	N (%)	6387	31%

	Overweight	N (%)	5236	25%
	Obese	N (%)	3948	19%
Systolic blood pressure (SBP)		Mean (SD)	122.97	17.30
		Missing (%)	11233	55%
Diastolic blood pressure (DBP)		Mean (SD)	71.13	11.14
		Missing (%)	11233	55%
Hypertension		N (%)	1860	9%
Cholesterol		Mean (SD)	5.00	1.07
		Missing (%)	13472	66%
High-density lipoproteins (HDL)		Mean (SD)	1.48	0.42
		Missing (%)	13472	66%
Statins		N (%)	1799	9%
HbA1c		Mean (SD)	5.71	0.78
		Missing (%)	13557	66%
Type 2 Diabetes		N (%)	1438	7%
Past cardiovascular disease (CVD)		N (%)	211	1%
Depression		N (%)	1219	6%
Arthritis		N (%)	840	4%
EQ-5D		Mean (SD)	0.87	0.19
		Missing (%)	13305	65%

N refers to the number of people in the population subgroup. Abbreviations: SD; standard deviation. IMD; Index of Multiple Deprivation. EQ-5D is a measure of health-related quality of life.

A complete dataset was required for all individuals at baseline. In addition, the questionnaire did not collect information about individual family history of diabetes. A variable was generated from the Whitehall II dataset (see below) (11). The proportion of the population with a family history of cardiovascular disease was generated from QRISK2 documentation (12). Health Survey England 2019 did not collect information on cardiovascular history or health-related quality of life (EQ-5D). These missing values were also imputed, informed by the data collected in HSE 2018.

Many individuals were lacking responses to some questions but had data for others. One way of dealing with this was to exclude all individuals with incomplete data from the sample. However, this would have reduced the sample size and representativeness dramatically, which would have been detrimental to the analysis. It was decided that it would be better to make use of all the data available to represent a broad range of individuals within the UK population. With this in mind, we decided to use assumptions and imputation models to replace missing data.

3.2.2 Re-weighting HSE data to UK population characteristics

Sample weights were generated for the Health Survey for England dataset so that the populations used in the model were representative of the United Kingdom general using iterative proportional fittings methods (13). We generate sample weights to reflect age, sex and NS-SEC across England, Wales, Scotland and Northern Ireland in 2022, combining census data from multiple sources. Counts by Age, Sex and NS-SEC 5 were available for Scotland and Northern Ireland (14, 15). However, counts by this combination was not available for England and Wales. Instead, 2022 population counts by age and sex were weighted by the proportion in each NS-SEC group for each age group (16, 17). Proportions in age, sex and NS-SEC 5 categories were applied to UK Counts (17). The population counts used to generate these weights are reported in Table 3.2.2. The summary statistics and simulated population are reported in Table 3.2.3. Missing data were imputed using the techniques described below.

Table 3.2.2: Population counts by sex and age group from UK Census data

		**Sex**											
		Male						**Female**					
**NS-SEC 5**		1	2	3	4	5	99	1	2	3	4	5	99
**Age**	5 to 7	1191984						1136827					
	8 to 10	1238247						1181473					
	11 to 12	845232						805309					
	13 to 15	1243560						1183732					
	16 to 19	169469	113936	43917	59030	288934	921113	161038	111971	38987	47136	266535	878593
	20 to 24	218659	147007	56665	76164	372800	1188475	214954	149460	52040	62917	355772	1172751
	25 to 29	849799	253677	184722	136862	482631	245088	883537	275092	178614	122993	492444	254828
	30 to 34	894211	266935	194376	144014	507854	257897	963869	300104	194854	134176	537218	277998
	35 to 39	913751	235852	270238	135679	464373	170905	984946	275150	271267	125483	494951	186792
	40 to 44	880521	227275	260411	130745	447485	164690	934345	261014	257331	119036	469524	177196
	45 to 49	841422	217183	248847	124939	427615	157377	877471	245126	241667	111791	440944	166410
	50 to 54	782339	262734	313907	141107	593275	162119	815132	305177	301094	127380	622669	170613
	55 to 59	785134	263673	315029	141611	595395	162698	819005	306627	302525	127986	625628	171424
	60 to 64	694127	233109	278513	125196	526380	143839	722732	270583	266964	112941	552086	151273
	65 to 69	493126	197869	196663	104933	443502	224569	510650	232378	188478	96495	485961	245196
	70 to 74	452027	181378	180273	96188	406539	205853	485776	221058	179297	91795	462290	233252
	75 to 79	376318	150999	150079	80077	338449	171375	422215	192134	155837	79784	401802	202732
	80 to 84	224225	89971	89423	47713	201661		279112	127013	103019	52742	265618	134020
	85 to 89	128909	51725	51410	27431	115937		185331	84337	68405	35021	176371	88989
	90 to 100	59447	23853	23708	12650	53465	187889	118931	54121	43897	22474	113181	57106

Table 3.2.3: Summary statistics for the simulation populations

			**HSE 2018 and HSE 2019 combined**		**Model Population (sampled and MI)**	
Variable		Unit	Mean or N	SD or %	Mean or N	SD or %
		N	20549		2,000,000	
Sex	Female	N (%)	11116	54%	1024372	51%
	Male	N (%)	9433	46%	975628	49%

Ethnicity	White	N (%)	17290	84%	1639878	82%
	Asian	N (%)	1837	9%	199822	10%
	Black	N (%)	690	3%	82882	4%
	Other	N (%)	732	4%	77418	4%
IMD	1 (lease deprived)	N (%)	3994	19%	348414	17%
	2	N (%)	4036	20%	385318	19%
	3	N (%)	4050	20%	381969	19%
	4	N (%)	4240	21%	424215	21%
	5 (most deprived)	N (%)	4229	21%	460084	23%
Age		Mean (SD)			43	22
	Age 5 to 7	N (%)	820	4%	51492	3%
	Age 8 to 10	N (%)	825	4%	82447	4%
	Age 11 to 12	N (%)	545	3%	46606	2%
	Age 13 to 15	N (%)	706	3%	76339	4%
	Age 16 to 19	N (%)	689	3%	98610	5%
	Age 20 to 39	N (%)	4411	21%	556492	28%
	Age 40 to 69	N (%)	8102	39%	793937	40%
	Age 70 or above	N (%)	3180	15%	294077	15%
National statistics socio-economic classification	NSSEC1	N (%)	8633	42%	682412	34%
	NSSEC2	N (%)	2189	11%	225797	11%
	NSSEC3	N (%)	2330	11%	210809	11%
	NSSEC4	N (%)	1641	8%	114856	6%
	NSSEC5	N (%)	5082	25%	472888	24%
	NSSEC99	N (%)	462	2%	293238	15%
Smoking	Current Smoker	N (%)	2570	13%	313109	16%
Weight		Mean (SD)	69.90	25.88	78.2[1]	18.3
Height		Mean (SD)	1.62	0.18	1.7[1]	0.1
BMI		Mean (SD)	26.17	6.54	27.6	5.8
BMI Classification	Underweight	N (%)	609	3%	170390	9%
	Healthy Weight	N (%)	6387	31%	702997	35%
	Overweight	N (%)	5236	25%	639001	32%
	Obese	N (%)	3948	19%	487612	24%
SBP		Mean (SD)	122.97	17.30	125[2]	16.6
DBP		Mean (SD)	71.13	11.14		
Hypertension		N (%)	1860	9%	166371	8%
Chol		Mean (SD)	5.00	1.07	5.02[2]	1.06
HDL		Mean (SD)	1.48	0.42	1.45[2]	0.42
Statins		N (%)	1799	9%	162064	8%
HbA1c (%)		Mean (SD)	5.71	0.78	5.73[2]	0.79
Diabetes		N (%)	1438	7%	140463	7%
Past CVD		N (%)	211	1%	35376	2%
Depression		N (%)	1219	6%		
Arthritis		N (%)	840	4%		
EQ-5D		Mean (SD)	0.87	0.19	0.89[3]	0.19

CVD cardiovascular disease; EQ-5D Euroqol 5 dimensions; HDL high-density lipoprotein; MI multiple Imputation; SBP systolic blood pressure; DBP diastolic blood pressure; SD standard deviation; NSSEC National Socioeconomic classification; HSE Health Survey for England

[1] Includes population aged 18 and over.

[2] Includes population aged 25 and over.

[3] Children under 16 are assigned EQ-5D of 1 as not estimated in HSE.

3.2.3 HSE 2018 Missing data imputation

Ethnicity

Only a small number of individuals had missing data for ethnicity. In the QRISK2 algorithm the indicator for white included individuals for whom ethnicity is not recorded. In order to be consistent with the QRISK2 algorithm we assumed that individuals with missing ethnicity data were white.

Anthropometric data

Data were imputed using multiple imputation methods from the mice package in R. Predictive mean methods imputation methods were used to impute missing data based on age, socioeconomic status, anti-hypertensive

treatment, diabetes diagnosis, anthropometric, metabolic data and EQ-5D within the dataset. Limiting the number of relationships to include in the imputation method was necessary to reduce the computational time required by the imputation. Figure 3.2.1 and Figure 3.2.2 illustrate the distribution of height and weight after imputation.

Metabolic data

Data were imputed using multiple imputation methods from the mice package in R. Predictive mean matching methods imputation methods were used to impute missing data based on age, socioeconomic status, anti-hypertensive treatment, diabetes diagnosis, anthropometric, metabolic data and EQ-5D within the dataset. Predictive mean matching is a semi-parametric method. Rather than calculating a mean, it identified 30 of donor cases with observed values closest to the predicted value of the missing case, and then randomly draws an actual observed value from this pool to impute. Limiting the number of relationships to include in the imputation method was necessary to reduce the computational time required by the imputation. Figure 3.2.3 illustrates the distribution of systolic blood pressure, HbA1c, and total cholesterol after imputation.

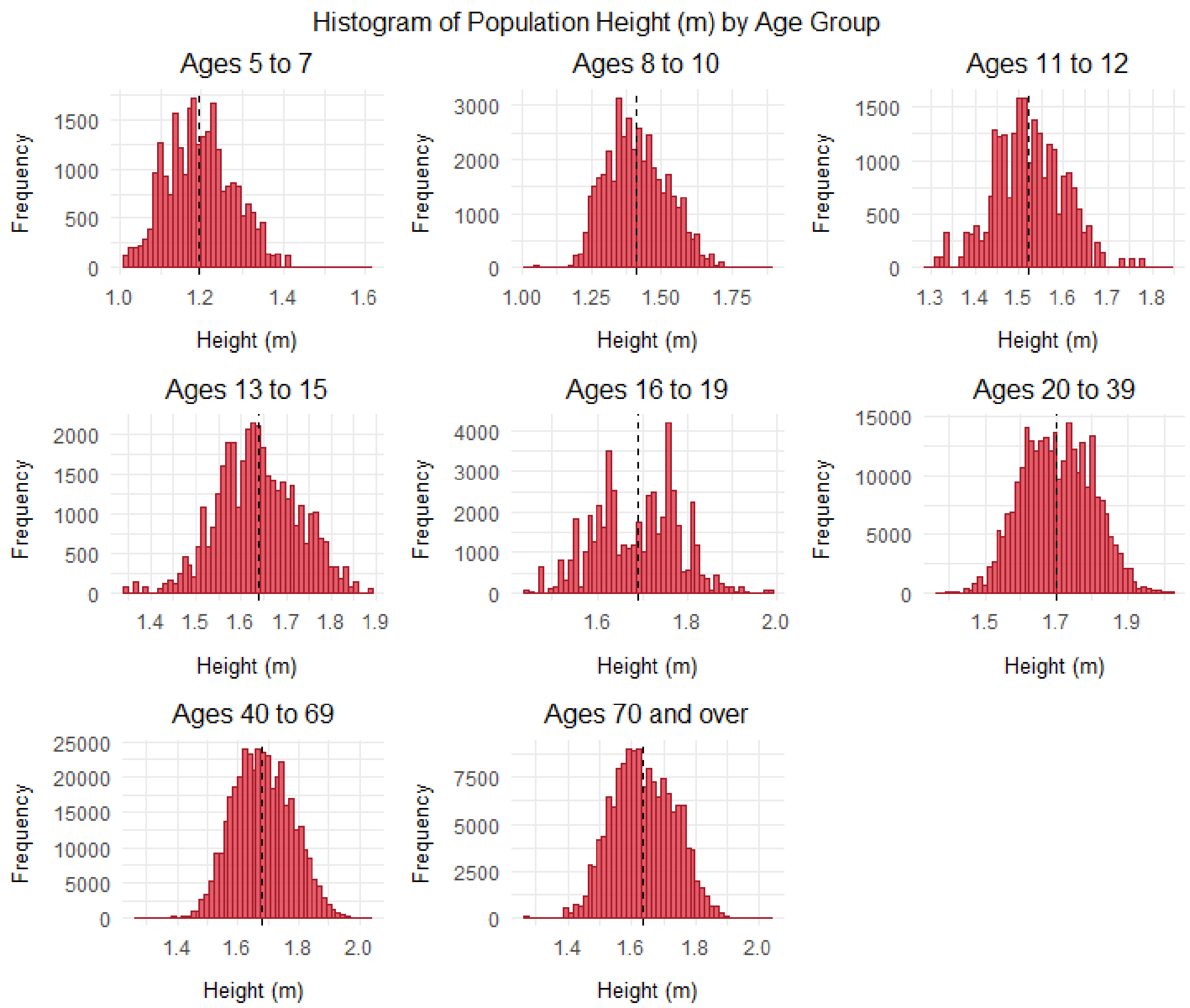

Figure 3.2.1: Histogram illustrating post-imputation height by age groups

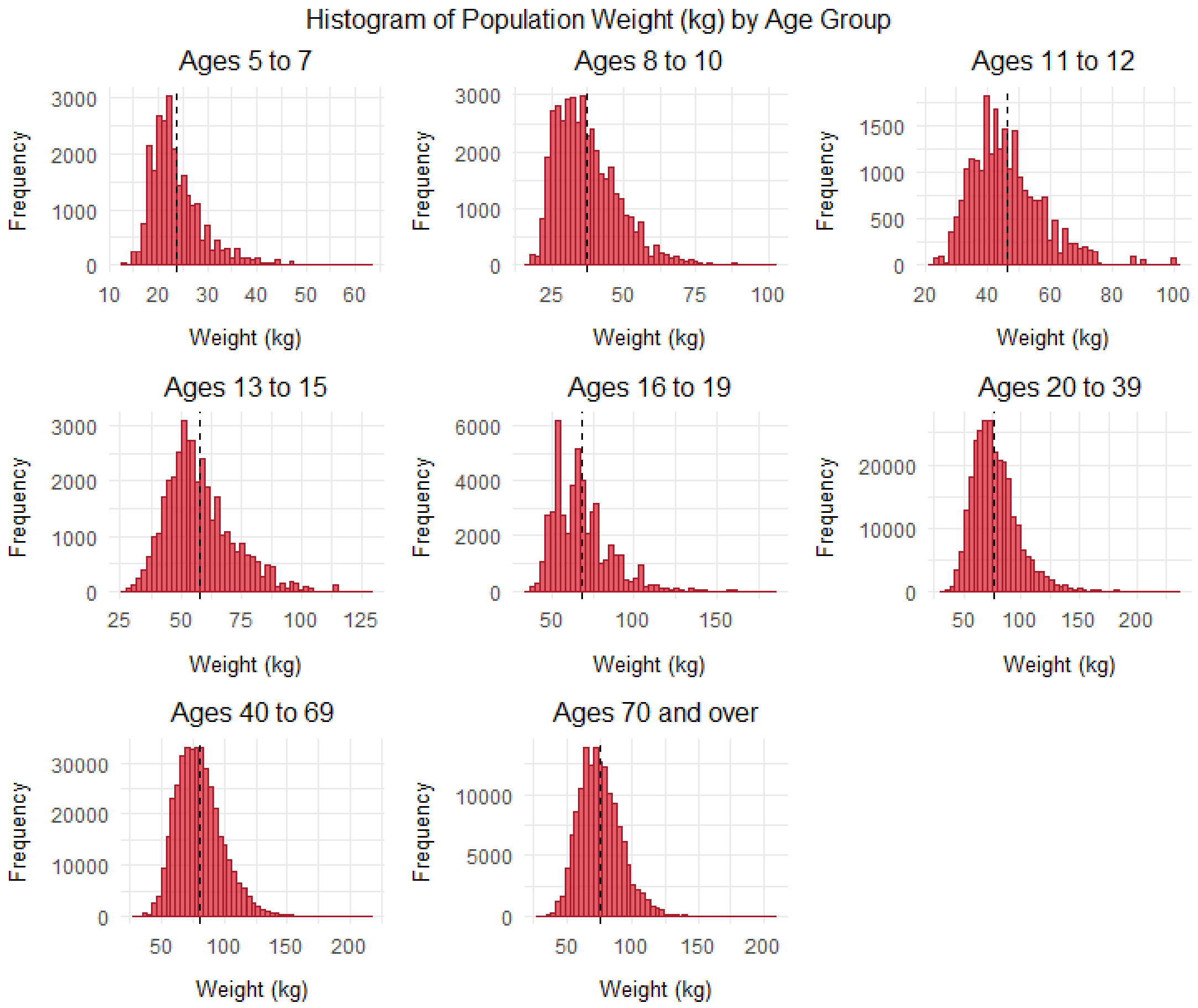

Figure 3.2.2: Histogram illustrating post-imputation weight by age groups

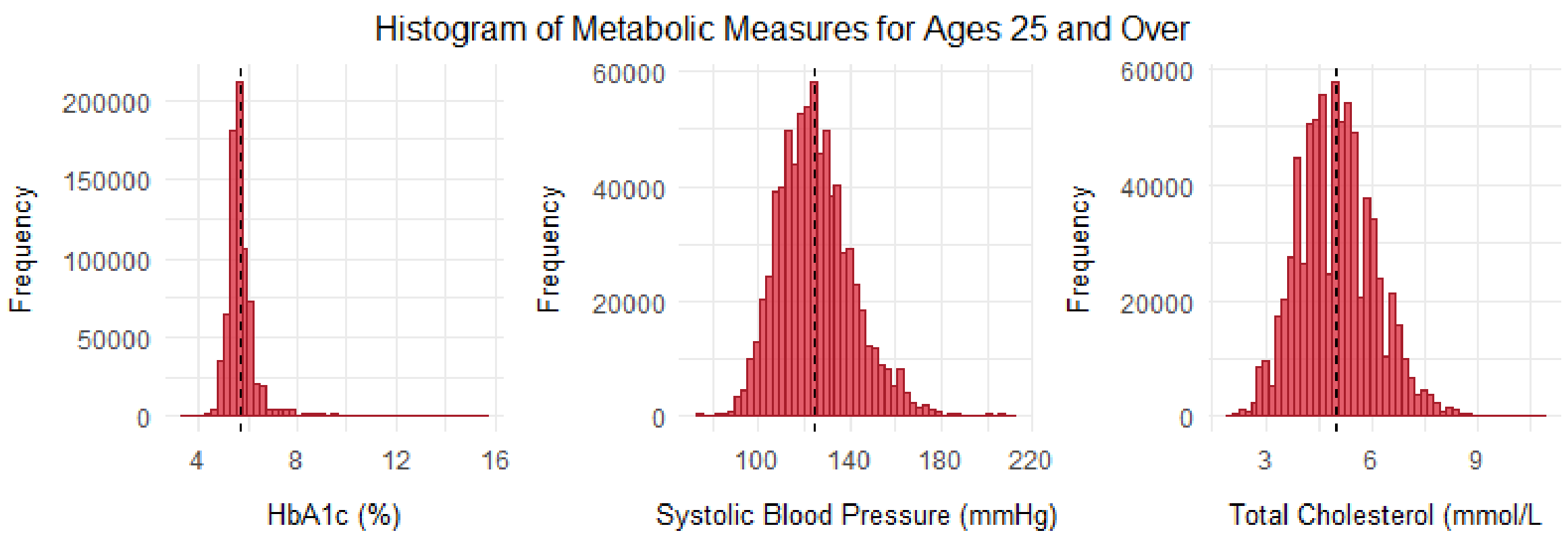

Figure 3.2.3: Histogram of HbA1c, systolic blood pressure and total cholesterol post-imputation for individuals aged 25 or more

Treatment for Hypertension and Statins

A large proportion of individuals had missing data for questions relating to whether they received treatment for hypertension or high cholesterol. The majority of non-responses to these questions were coded to suggest that the question was not applicable to the individual. As a consequence, it was assumed that individuals with missing treatment data were not taking these medications.

Anxiety/Depression

Most individuals who had missing data for anxiety and depression did so because the question was not applicable. A small sample N=69 refused to answer the question. We assumed that individuals with missing data for anxiety and depression did not have severe anxiety/depression.

Smoking

Individuals with missing data for smoking status were assumed to be non-smokers, without a history of smoking.

Rheumatoid Arthritis

Individuals reporting existing arthritis/rheumatism were assigned to a history rheumatoid arthritis.

Atrial Fibrillation

Individuals reporting "other heart conditions" in response to questions about long-standing illnesses were assumed to have a history of Atrial Fibrillation.

Family history of diabetes

No questions in the HSE referred to the individual having a family history of diabetes, so this data had to be imputed. It was important that data was correlated with other risk factors for diabetes, such as HbA1c and ethnicity. Current HbA1c as a biological proxy to impute and allocate the correct proportions of historical risk across a population where family history data is missing. We analysed a cross-section of the Whitehall II dataset to generate a logistic regression to describe the probability that an individual has a history of diabetes conditional on their HbA1c and ethnic origin. The model is described in Table 3.2.4 .

Table 3.2.4: Imputation model for history of diabetes

	**Coefficient**	**Standard error**
Intercept	-3.29077	0.4430
HbA1c	0.28960	0.0840
Non-white ethnicity	0.81940	0.1388

While the model exhibits low discriminative power at the individual level (AUROC = 0.55), its primary utility is population-level risk stratification rather than individual diagnosis. The model captures the baseline epidemiological associations of ethnicity and HbA1c to allocate the aggregate proportion of diabetes history across the population. Therefore, despite low individual classification accuracy, the model remains valid for population modelling.

History of Cardiovascular disease

Individuals with a history of cardiovascular disease were assigned to a health status of either stable angina, unstable angina, myocardial infarction, or stroke based on responses to health survey for England responses to long standing conditions. Individuals reporting stroke were assigned to stroke, heart attack/angina to unstable angina and MI at random using distributions estimated in the statins HTA (18).

Baseline EQ-5D

Data were imputed using multiple imputation methods from the mice package in R. Predictive mean methods imputation methods were used to impute missing data based on age, socioeconomic status, anti-hypertensive treatment, diabetes diagnosis, anthropometric, metabolic data and EQ-5D within the dataset. Limiting the number of relationships to include in the imputation method was necessary to reduce the computational time required by the imputation. Figure S3.2.4 illustrates the distribution of EQ-5D after imputation.

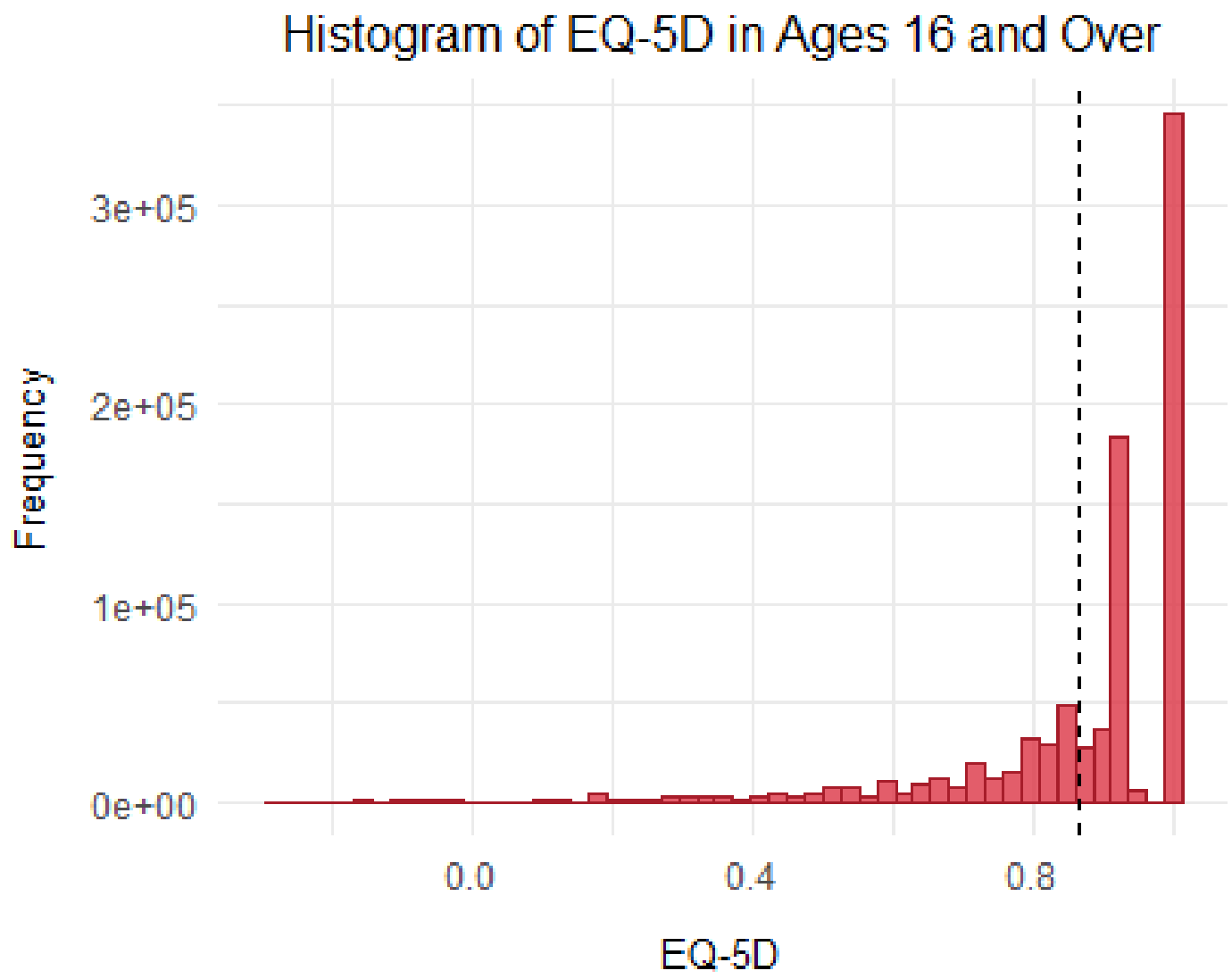

Figure S3.2.4: Histogram of post-imputation EQ-5D for individuals aged 16 and over

3.3 Baseline Population Characteristics: Food Consumption

3.3.1 Baseline food and drink consumption

The National Diet Nutrition Survey (NDNS), combining all available years (2008-2019), provided the data to generate the baseline consumption for the model population (19). We chose to include all waves of survey data to capture variability in diets across demographic groups as limited waves may suffer underrepresentation of certain populations. Foods were allocated across 50 food groups. Individual's total grams per day were recorded for each food group along with detailed daily nutritional intake. This represents a modification and simplification of the NDNS Main Food Groups to improve alignment with the Living Costs and Food Survey and food tax designs evaluated in HEALTHEI. However, attempts to simplify food groups against the need to retain variation in the nutritional value and price across food groups in order to capture the effects and benefits of shifts in consumption. Food groups relating to alcoholic beverage consumption and food supplements were not retained in the model.

We summarise and justify the main modifications to the food groups.

1. Remove tap water for drinking, dilution and cooking. As water bills are not included in the Living Costs and Food Survey food and drink survey it was important to modify the NDNS to align with the

purchasing patterns. Water used in purchased and retail recipes (not homemade) was reassigned to the retain the accuracy of the nutritional information for retail products. All remaining entries for tap water, water used as a dilutant or for infusions within the tea coffee and water food group were excluded.

2. Generate new categories for meat and vegetable ready meals. We identified misalignment between the NDNS and LCFS with regards to the categories for meats and ready meals. In the NDNS ready meals were distributed across multiple food groups by meat type, whereas the LCFS used a separate category for meat ready meals. We reassigned foods using the text content of the *FoodName* and *RecipeName* variables. Also assigned foods labelled manufactured from relevant *Subfoodgroupdesc* name variables.
3. Eggs and Egg products were assigned to other food groups, such as meringue to puddings, and purchased quiche to ready meals.
4. The Commercial toddler food group entries were reassigned to other food groups according to food type.
5. Out of home foods were identified by *FoodName* labels, *RecipeName* labels and responses to the 'Where' variable in the NDNS Food level dataset which indicates the location in which the food was eaten. Foods consumed in restaurants, cafes, hotels, canteen and leisure facilities were assigned to the out of home group. Foods from the food groups confectionery, soft drinks were not re-assigned as these are more frequently purchased pre-packaged in the out of home sector. Alcohol beverages were also excluded from the re-assignment.

The NDNS food-level dataset combines food entries for established foods and individual ingredients for recipes. For example, a purchased chicken curry ready meal could separate individual ingredients for the rice, chicken and sauce ingredients. In order to capture the nutritional content of purchased foods it was necessary to aggregate recipes from the manufacturer and retail setting, so that the foods could be evaluated for tax eligibility based on their nutritional content. NDNS foods were aggregated where foods were matched by participant id, date, time and for a limited set of sub-Recipe food group description terms that would predominantly include manufactured foods, such as "MANUFACTURED BEEF PRODUCTS INCLUDING READY MEALS". In NDNS years 9-11 the variable *RecipeName* provided additional detail on manufactured products using text content. Manufactured foods identifiable from the text entries were aggregated. The aim was to aggregate purchased foods but leave homemade recipes in a disaggregated format.

Table 3.3.1: Assigning NDNS Food groups

Name	Description/Examples	Source
Bacon and Ham	Pork products including bacon, gammon and ham. Including canned, smoked and unsmoked	NDNS MainfoodGroupDesc BACON AND HAM
Beef Veal and dishes	Beef and veal products used in home cooking, such as stewing steak, mince and brisket. Beef ready meals re-assigned to Meat Readymeals.	NDNS MainfoodGroupDesc BEEF AND VEAL DISHES. Retail meat ready meals removed.
Beverages dry weight	Hot and cold powdered drinks including drinks pods.	NDNS SubFoodGroupDesc BEVERAGE DRY WEIGHT within the MISCELLANEOUS MainFoodGroupDesc.
Biscuits	Sweet and savoury biscuits, including cereal bars.	NDNS MainfoodGroupDesc BISCUITS and RecipeSubfoodGroupDesc BISCUITS MANUFACTURED / RETAIL.
Breakfast cereals	All breakfast cereals, i.e Museli, Weetabix, rice crispies and cocoa pops. Branded and own brand.	NDNS MainfoodGroupDesc HIGH FIBRE BREAKFAST CEREALS and OTHER BREAKFAST CEREALS
Brown and wholemeal bread	Brown and wholemeal bread including sliced bread, specialist breads ie. Pitta, tortilla or rolls and 50% wholemeal.	NDNS MainfoodGroupDesc BROWN GRANARY AND WHEATGERM BREAD and WHOLEMEAL BREAD
Butter	Butter salted and unsalted. Includes Ghee and butter spreads.	NDNS MainfoodGroupDesc BUTTER
Cakes and Puddings	Purchased and homemade cakes, desserts and puddings. Includes sponge, tarts, meringue, pancakes, morning goods, jelly, doughnuts,	NDNS MainfoodGroupDesc PUDDINGS and BUNS CAKES PASTRIES & FRUIT PIES. Some Foodnames reassigned from EGGS AND EGG DISHES for meringues and pavlovas. RecipeSubfoodGroupDesc BUNS CAKES PASTRIES & FRUIT PIES and PUDDINGS assigned to this group.
Cheese	Hard and soft cheese.	NDNS MainfoodGroupDesc CHEESE
Chicken and Turkey dishes	Chicken and turkey products and dishes cooked or raw and used in home cooking, such as wings, breast and casserole. Chicken purchased ready meals re-assigned to Meat Readymeals, whereas coated chicken products retained in this food group.	NDNS MainfoodGroupDesc CHICKEN AND TURKEY DISHES and COATED CHICKEN. Retail meat ready meals removed.
Chips fried & roast potatoes & potato products	Fried, roast potatoes including chips. Homemade and retailed purchased and ready made.	NDNS MainfoodGroupDesc CHIPS FRIED & ROAST POTATOES AND POTATO PRODUCTS
Chocolate confectionery	Chocolates solid and bars with additions, includes chocolate covered nuts and raisins. Includes milk chocolate covered biscuits such as twix and munchies.	NDNS MainfoodGroupDesc CHOCOLATE CONFECTIONERY
Crisps and savoury snacks	Includes crisps, popcorn, pork scratchings, pretzels and coated peanuts. Include toddler snacks	NDNS MainfoodGroupDesc CRISPS AND SAVOURY SNACKS
Eggs and Egg dishes	Eggs prepared in various methods.	NDNS MainfoodGroupDesc EGGS AND EGG DISHES. Most egg dishes re-assigned to other food groups.
Fruit	All fruits raw and cooked. Include dried fruit and fruit in cans.	NDNS MainfoodGroupDesc FRUIT.
Fruit Juice	Fruit juices and fruit-based smoothies.	NDNS MainfoodGroupDesc SMOOTHIES 100% FRUIT AND/OR JUICE and FRUIT JUICE.
Ice cream	Ice cream and ice lollies including choc ices, sorbet and cones.	NDNS MainfoodGroupDesc ICE CREAM
Lamb and lamb dishes	Lamb and lamb products and dishes cooked or raw and used in home cooking, such as shoulder, chops and casserole. Chicken purchased ready meals re-assigned to Meat Readymeals.	NDNS MainfoodGroupDesc LAMB AND LAMB DISHES
Liver & dishes	Liver and pate	NDNS MainfoodGroupDesc LAMB & DISHES

Low Fat Milk	Skimmed, semi skimmed and 1% milk	NDNS MainfoodGroupDesc 1% Fat Milk and SKIMMED MILK and SEMI SKIMMED MILK.
Margarine and Oils	Cooking oils, suet hard block margarines lards.	NDNS MainfoodGroupDesc PUFA MARGARINE & OILS and OTHER MARGARINE FATS AND OILS
Meat pies and pastries	Meat pies including pasty, potato pies, pork pie, meat samosa.	NDNS MainfoodGroupDesc MEAT PIES AND PASTRIES
Meat ready meals	Prepared meals purchased ready to eat in retail.	Analyst compiled list of FoodName and RecipeNames for eligible foods from the NDNS datasets. In additions foods identified from the RecipeSubFoodGroupDesc categories for manufactured meat dishes.
Milk drinks	Flavoured milks, milkshakes and prepared milky coffee drinks	Analyst compiled list of FoodName and RecipeNames.
Nuts and seeds	Raw nuts and seeds and related products including tahini, peanut butter and desiccated coconut.	NDNS MainfoodGroupDesc NUTS AND SEEDS
Oily Fish	Anchovies, Mackerel, salmon, kippers, herring, sardines, and trout. Fresh and canned products, including pate and paste.	NDNS MainfoodGroupDesc OILY FISH. Some products reclassified to the meat ready meal categories, ie purchased fish pie.
Other bread	Gluten free bread and rye bread.	NDNS MainfoodGroupDesc OTHER BREAD
Other meat and meat products	Duck, venison, black pudding, corned beef, tongue, haggis, pheasant, and meat paste	NDNS MainfoodGroupDesc OTHER MEAT AND MEAT PRODUCT
Other milk and Cream	Non-cow milks including goat and vegetable-based milk alternatives. Also includes other dairy products such as cream, condensed and evaporated milk	NDNS MainfoodGroupDesc OTHER MILK AND CREAM
Other potatoes and Potato salads & Dishes	Baked, mashed boiled and other prepared potatoes	NDNS MainfoodGroupDesc OTHER POTATOES POTATO SALADS & DISHES.
Other white fish Shellfish and Fish dishes	Other white fish including cod, sea bass, hake, seafood, tuna and haddock. Raw and prepared dishes.	NDNS MainfoodGroupDesc OTHER WHITE FISH SHELLFISH & FISH DISHES
Out of home	Food determined to be purchased from take-aways and restaurants.	Analyst compiled list of FoodName and RecipeNames. Also identified food eaten in out of home settings using the Where variable, excluding confectionery, soft drinks and alcoholic drinks.
Pasta rice and other cereals	Pizza, pasta, noodles, cous cous and rice dishes. Includes plain flour and other cereals.	NDNS MainfoodGroupDesc PASTA RICE AND OTHER CEREALS
Pork and Dishes	Uncured pork products used in home cooking, such as loin, mince and ribs. pork ready meals re-assigned to Meat Readymeals.	NDNS MainfoodGroupDesc LOW FAT SPREAD and REDUCED FAT SPREAD
Reduced fat spread	Reduced and low fat spreads such as benecol, polyunsaturated fat, flora, sunflower or olive oil spread.	NDNS MainfoodGroupDesc PORK AND DISHES
Salad and other raw vegetables	Raw vegetables and uncooked vegetable dishes such as coleslaw.	NDNS MainfoodGroupDesc SALAD AND OTHER RAW VEGETABLES
Sausages burger and kebabs	Sausages, burgers kebabs. Include frankfurter sausages.	NDNS MainfoodGroupDesc BURGERS AND KEBABS and SAUSAGES
Savoury sauces pickles gravies & condiments	Pasta sauces, pesto, stir fry or curry sauces, gravy and stock cubes. Ketchup, mayonnaise, pickles, chutney, salad cream, and salad dressing. Includes salt and pepper and spices. Baking ingredients for baking powder and bicarbonate of soda.	NDNS SubFoodGroupDesc SAVOURY SAUCES PICKLES GRAVIES & CONDIMENTS
Soft drinks low calorie	Soft drinks with low sugar, such as diet or zero sugar products.	NDNS MainFoodGroupDesc SOFT DRINKS LOW CALORIE
Soft drink not low calorie	Soft drinks with sugar.	NDNS MainFoodGroupDesc SOFT DRINKS NOT LOW CALORIE
Soup homemade and retail	Soups including vegetable and meat soups. Dry mixes as served, canned and homemade included.	NDNS SubFoodGroupDesc SOUP MANUFACTURED/ RETAIL and SOUP HOMEMADE

Sugar confectionery	Sweets including boiled, fudge, chewy, chewing gum, yoghurt covered raisins and marshmallows.	NDNS MainFoodGroupDesc SUGAR CONFECTIONERY
Sugars preserves and sweet spreads	Preserves include jams, lemon curd, marmalade. Chocolate spread and cake toppings such as marzipan and icing. Sugar (i.e. white, demerara and dark) and syrups.	NDNS MainFoodGroupDesc SUGARS PRESERVES AND SWEET SPREADS
Tea coffee and Water	Tea coffee and mineral water. Tea includes infusion black tea, herbal tea. Coffee includes infusion coffee. Coffee and tea powders included in dry weight beverages.	NDNS MainFoodGroupDesc TEA COFFEE AND WATER
Vegetables not raw	Cooked, canned, frozen, dried and pickled vegetables,	NDNS MainFoodGroupDesc VEGETABLES NOT RAW
Veg readymeal	Retail prepared vegetable ready meals.	Analyst compiled list of FoodName and RecipeNames for eligible foods from the NDNS datasets. In additions foods identified from the RecipeSubFoodGroupDesc categories for manufactured vegetable dishes.
White bread	White bread including sliced bread, specialist breads ie. Pitta, naan, tortilla or rolls, English muffins and bagels.	NDNS MainFoodGroupDesc WHITE BREAD
White fish coated or fried	White fish (ie Cod or haddock) coated in flour or breadcrumbs or batter. Includes scampi and fish cakes	NDNS MainFoodGroupDesc WHITE FISH COATED OR FRIED
Whole milk	Whole milk	NDNS MainFoodGroupDesc WHOLE MILK
Yoghurt fromage frais and dairy desserts	Yoghurt including probiotic, kefir and lassi. Dairy desserts with cream or mousse.	NDNS MainFoodGroupDesc YOGURT FROMAGE FRAIS AND DAIRY DESSERTS and RecipeSubfoodGroupDesc YOGURT FROMAGE FRAIS AND DAIRY

3.3.2 Assigning Consumption to the population

Food and non-alcoholic drink consumption data from the National Diet and Nutrition Survey (NDNS) was collated across all available years of data (2008-2019) to generate a dataset containing both demographic and consumption data (19). Earlier NDNS samples may not capture recent trends in food consumption. However, in order to merge dietary data with the HSE individuals, and capture associations with sociodemographic characteristics, it was necessary to have good representation across a broad spectrum of individuals. Sample weights were generated for the NDNS dataset so that the populations used to allocate consumption to the model population were also representative of the United Kingdom general using iterative proportional fittings methods (13). The same age and sex and NS-SEC population counts, taken from census data, determined these weights (Table 3.2.2).

3.3.3 Assigning Consumption by Demographic Group

Suitable consumption data was assigned to each individual within the simulated population based on their characteristics. The two populations, the sampled population using HSE, and the sampled combined NDNS population, were categorized into 96 mutually exclusive groups based on age, sex and national statistics socio-economic classification (NS-SEC 5) (Table 3.2.2). These groupings were chosen to balance important determinants of consumption behaviour, while facing limitations in data availability for certain populations. Ethnic group may also influence consumption behaviour, however, test revealed that it could not be considered within the grouping without generating large numbers of sociodemographic groups with very small samples. Ethnicity could only be included at this stage if another variable was removed. The mean consumption in grams across the food groups of each demographic group was assigned to all those within that group. While assigning mean consumption per group may limit representation of extreme consumption patterns, this method mitigates the potential for consumption patterns in which only a few foods are consumed (the NDNS collected a food diary over 3 days) that may excessively influence outcomes. We acknowledge that average consumption patterns may obscure skewed consumption patterns that could impact consumer responses. Therefore, a sensitivity analysis using an alternative method assigning individualised consumption was included (See section 4.8.6).

Table 3.2.2: Variable categories used to determine 96 demographic groupings.

Age (years)	Sex	NS-SEC 5
5 to 7	Female	Managerial and professional occupations (higher or lower)
8 to 10	Male	Intermediate occupations
11 to 12		Small employers and own account workers
13 to 15		Low Supervisory and technical occupations
16 to 19		Semi-routine or routine occupations
20 to 39		Other
40 to 69		
70 or above		

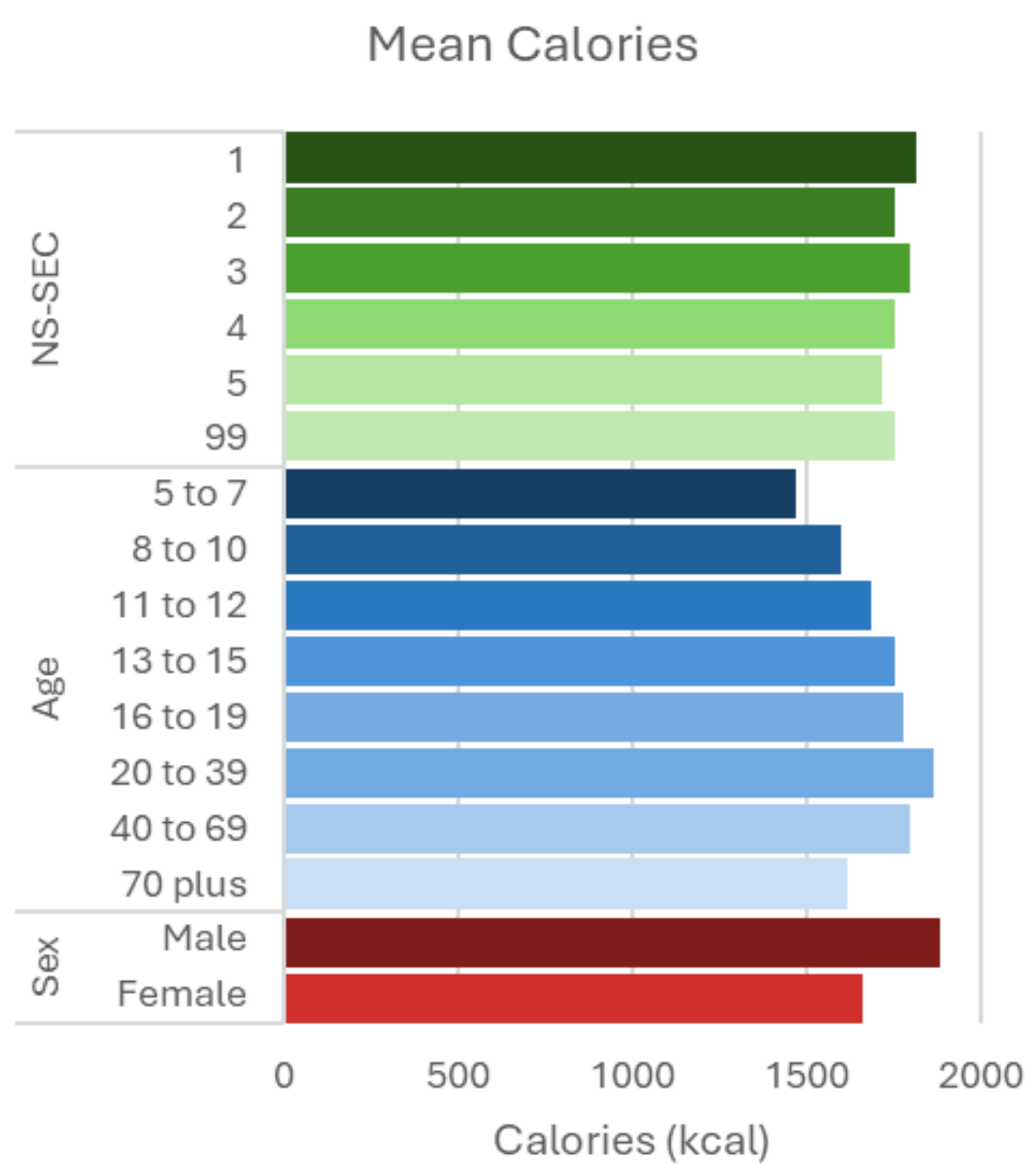
Mean Calories
NS-SEC
1
2
3
4
5
99
Age
5 to 7
8 to 10
11 to 12
13 to 15
16 to 19
20 to 39
40 to 69
70 plus
Sex
Male
Female
0
500
1000
1500
2000
Calories (kcal)

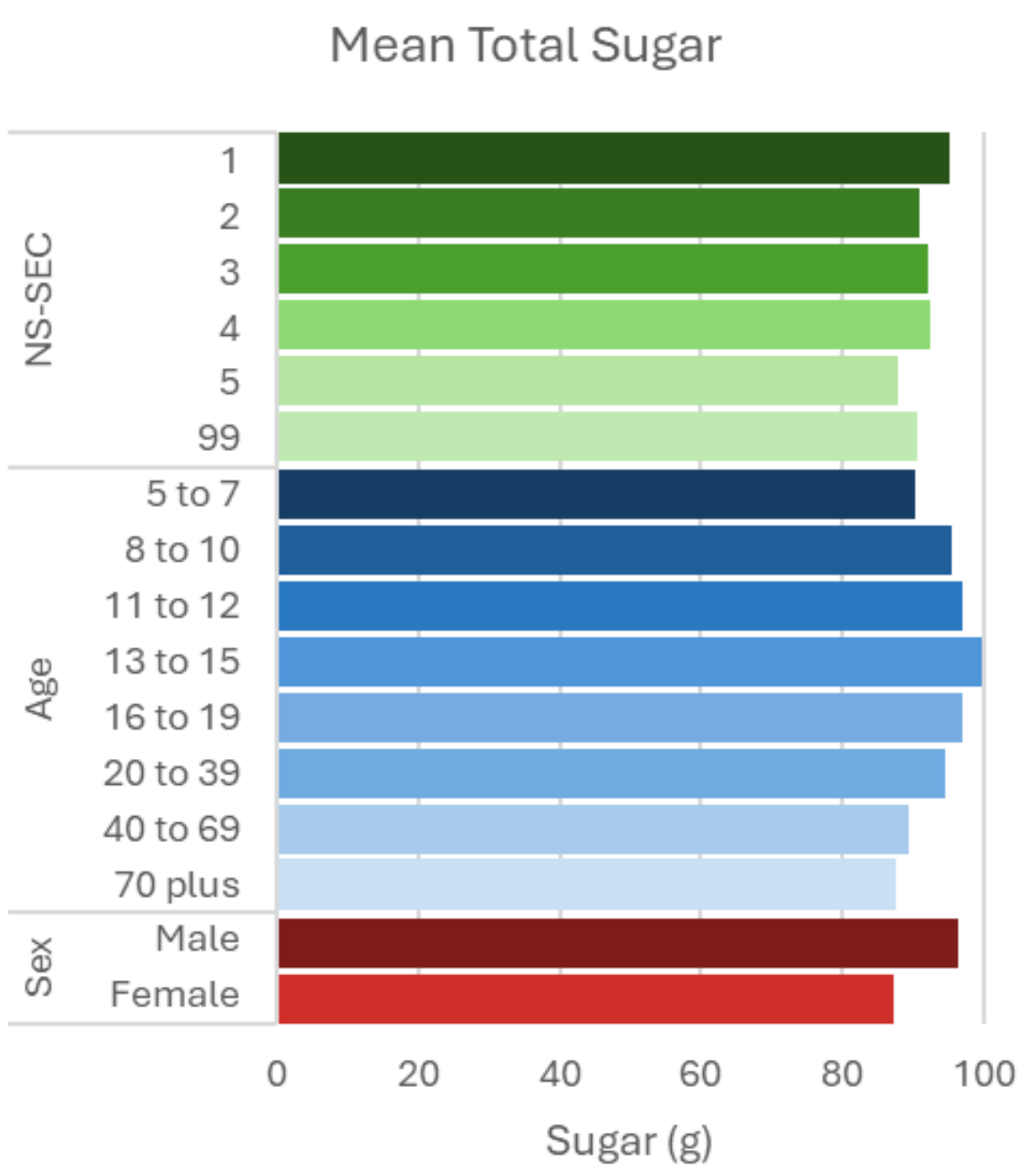
Mean Total Sugar
NS-SEC
1
2
3
4
5
99
Age
5 to 7
8 to 10
11 to 12
13 to 15
16 to 19
20 to 39
40 to 69
70 plus
Sex
Male
Female
0
20
40
60
80
100
Sugar (g)

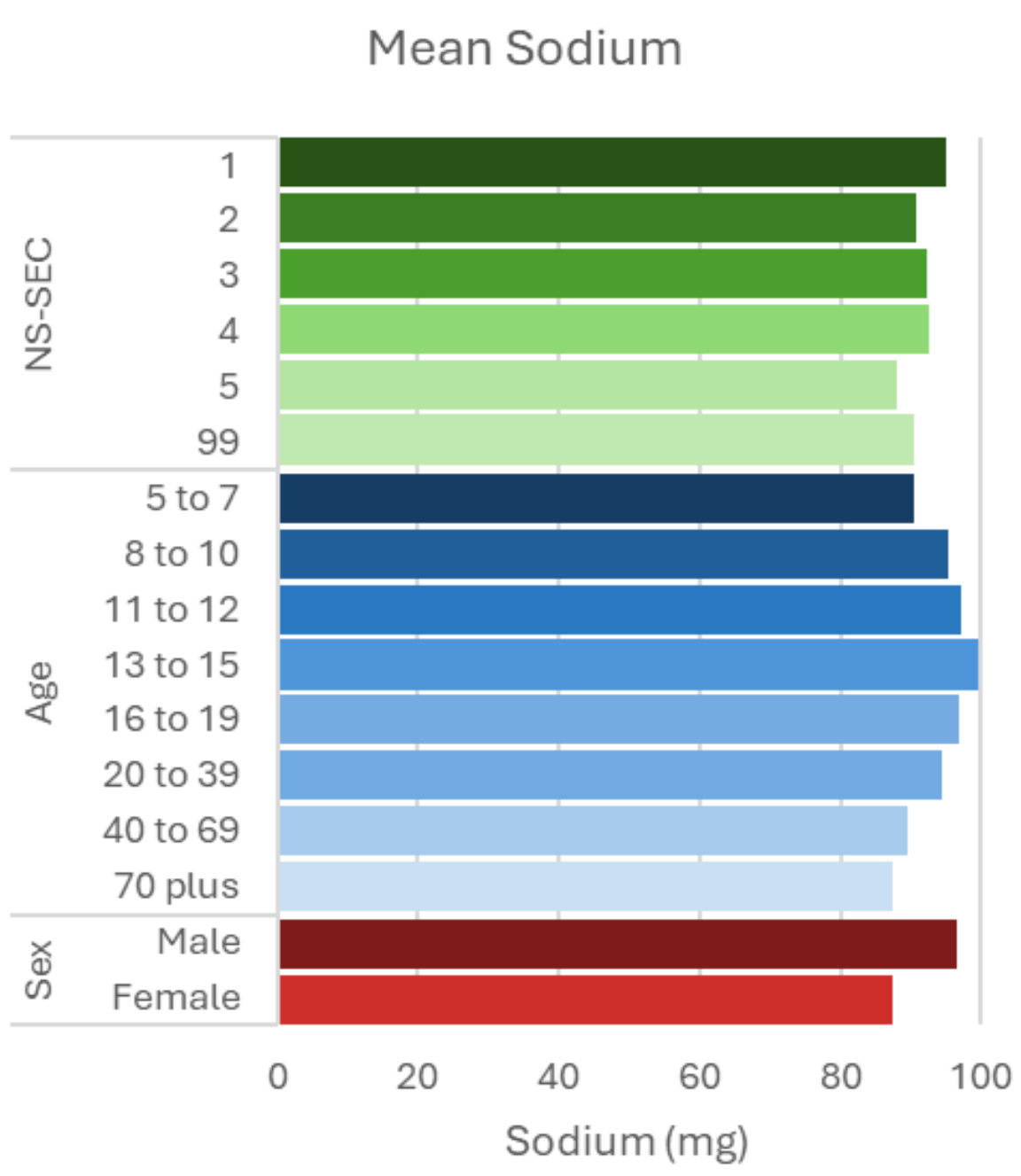
Mean Sodium
NS-SEC
1
2
3
4
5
99
Age
5 to 7
8 to 10
11 to 12
13 to 15
16 to 19
20 to 39
40 to 69
70 plus
Sex
Male
Female
0
20
40
60
80
100
Sodium (mg)

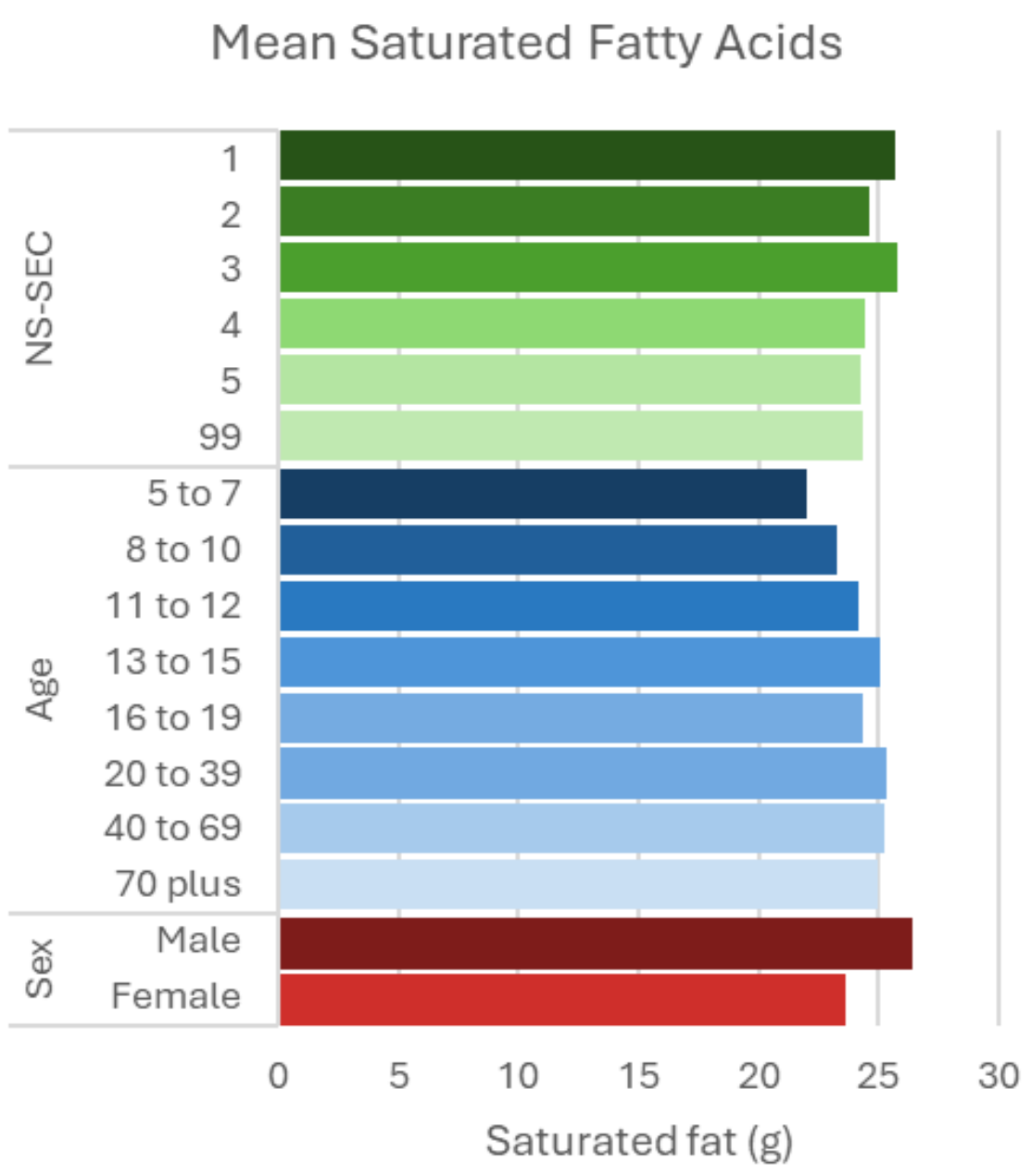

Mean Saturated Fatty Acids
NS-SEC
1
2
3
4
5
99
Age
5 to 7
8 to 10
11 to 12
13 to 15
16 to 19
20 to 39
40 to 69
70 plus
Sex
Male
Female
0
5
10
15
20
25
30
Saturated fat (g)

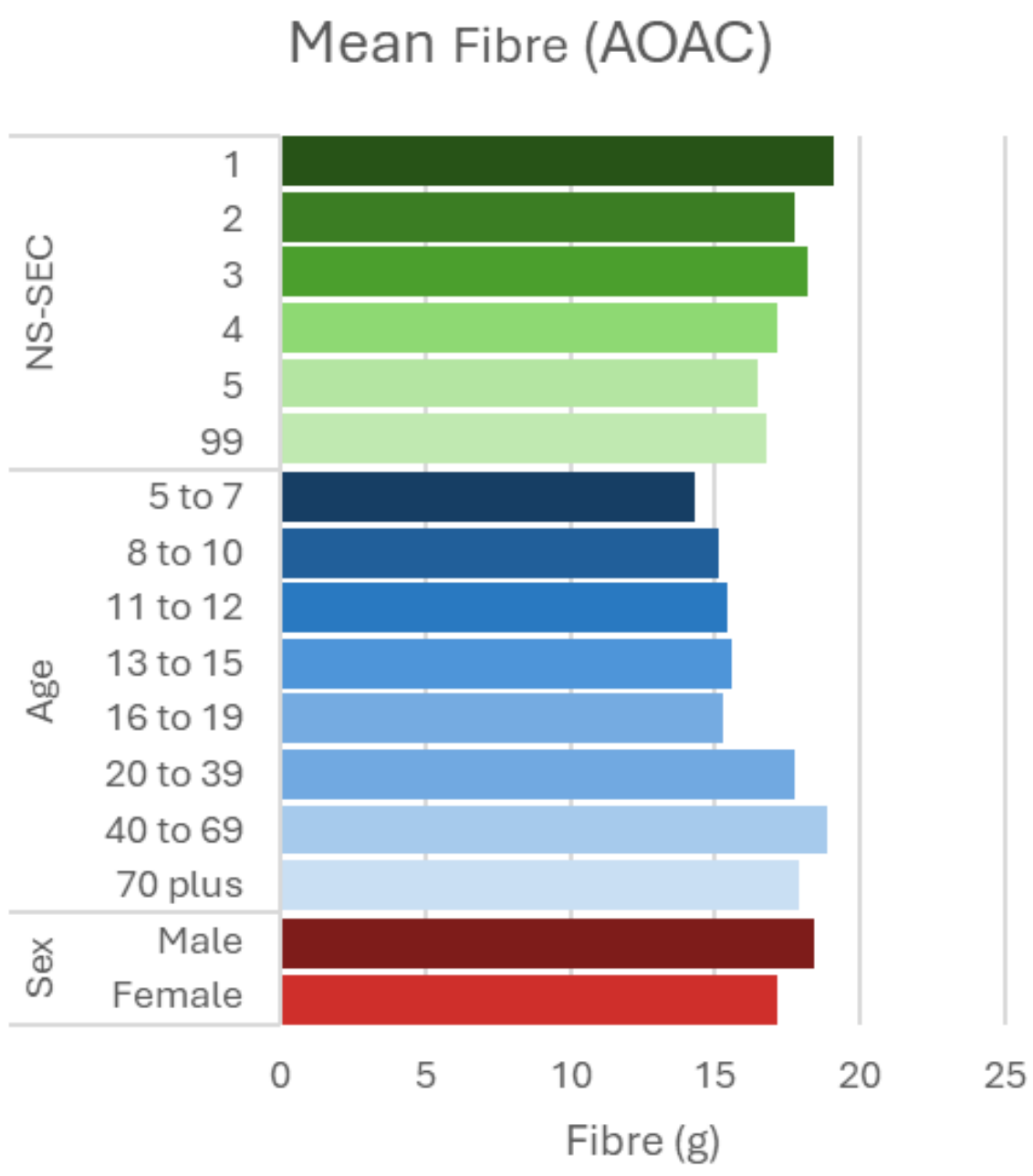

Mean Fibre (AOAC)
NS-SEC
1
2
3
4
5
99
Age
5 to 7
8 to 10
11 to 12
13 to 15
16 to 19
20 to 39
40 to 69
70 plus
Sex
Male
Female
0
5
10
15
20
25
Fibre (g)

4 The Demand Model

4.1 Overview

This demand model estimates how food tax policies affect prices, consumer purchasing behaviour via elasticities, expenditure (food and tax), and subsequent nutritional and health outcomes, while also accounting for industry price responses and reformulation.

4.1.1 Key assumptions (Base case)

- **No budget adjustments:** The model assumes households don't adjust their overall income or spending power when food prices change.
- **No switching within food groups:** It assumes people won't swap a premium brand for a cheaper brand within the same food category. This might overlook the health impacts of switching similar products.
- **Simplified pricing:** Food prices are based entirely on average costs for different income groups, ignoring other personal characteristics that can influence shopping habits.
- **Average eating habits:** The model uses broad averages for food intake based only on age, sex, and social/job status.
- **Simplified** calorie to weight model**:** Weight changes from calories are modified by age, current weight, sex, carbohydrate intake, and sodium. Height and physical activity levels to not impact the relationship between calorie intake and weight.
- **Instant reactions:** It assumes consumers react to price changes immediately, and that their new purchasing habits are maintained over the lifetime.

4.1.2 Demand Model Structure

Figure S4.1.1 represents the structure of the demand model. The baseline consumption of the model population is split into taxed and not taxed consumption for each tax policy design. The policy determines the price change for each food group. Consumer behaviour in response to price changes is determined through price elasticities of demand, generating estimated consumption changes. Nutritional changes and their impact on metabolic risk factors are then estimated. Additional analyses consider the industry response to the tax on price change, through tax pass through, and product change, through reformulation.

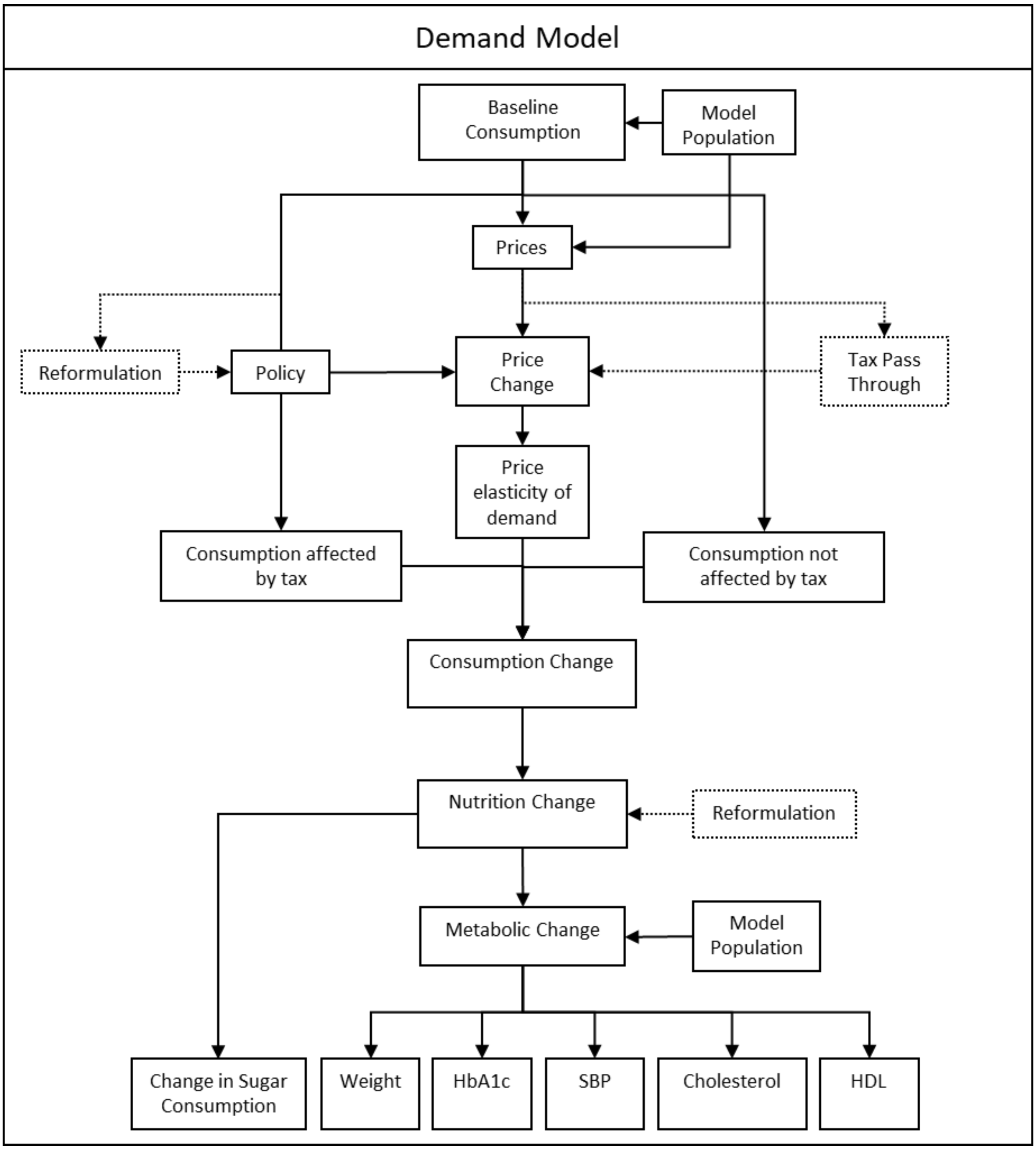

Figure S4.1.1: Schematic of Demand Model Structure

4.2 Estimate consumption affected by tax

Foods that would be taxed under each policy design were identified from the food-level NDNS data for years 9 to 11(19). Additional detail provided in these later waves enabled more accurate identification of eligible foods. For each tax design we identified a list *FoodNames* and *RecipeName* based on the food type rather the nutritional content. The lists were grouped by food categories defined by the Schedule B and UK voluntary reformulation guidelines. Once the eligible foods were identified a second process of selection applied criteria on the nutritional content of the foods.

For each individual the total grams of tax eligible food was summed by food group. To estimate the percentage of consumption within each food group that is impacted by the tax policies for the model population, a series of regressions were performed. The percentage of each food group that is taxed under each policy design is calculated using food-level data for individuals within NDNS waves 9 to 11. This sample was used to estimate linear regression models predicting the percentage of grams consumed that are taxed for each food group conditional on age, sex and socioeconomic classification (NS-SEC) in individuals that consumed some taxed food for that category. The categorical demographic variables used are described in Table 4.2.1.

Table 4.2.1: Demographic groups used as covariates in regressions used to estimate percentage of consumption taxed

Age (years)	Sex	NS-SEC 5
5 to 7	Female	Managerial and professional occupations (higher or lower)
8 to 10	Male	Intermediate occupations
11 to 12		Small employers and own account workers
13 to 15		Low Supervisory and technical occupations
16 to 19		Semi-routine or routine occupations
20 to 39		Other
40 to 69		
70 or above		

This was performed for each tax design. In food groups where there is not enough data to inform the regression (at least 130 observations above 0), a mean percent is recorded to be applied to all individuals that consumed some taxed food. Regression coefficients were sampled assuming a multivariate normal distribution defined by the variance-covariance matrices. The mean percent, when a regression is not performed, is sampled from a normal distribution, restricted between 0 and 1. The regression outputs for some key food groups across the tax designs are provided in Table 4.2.2 .

Table 4.2.2: Regression outputs for the proportion of consumption taxed of those consuming taxed foods affected by tax policy for some key food groups.

		Cakes and Puddings			Biscuits			Chocolate Confectionary		
		Sugar Snacks	Unhealthy Food	Sugar and Salt	Sugar Snacks	Unhealthy Food	Sugar and Salt	Sugar Snacks	Unhealthy Food	Sugar and Salt
	Intercept	0.598 (0.035)***	0.842 (0.019)***	0.976 (0.009)***	0.838 (0.018)***	0.880 (0.015)***	0.994 (0.004)***	0.995 (0.005)***	1	1
Age Group	5 to 7	-	-	-	-	-	-	-	-	-
	8 to 10	0.030 (0.044)	0.026 (0.024)	-0.008 (0.011)	0.026 (0.022)	0.006 (0.018)	-0.003 (0.005)	-0.003 (0.006)	-	-
	11 to 12	0.117 (0.051)*	0.036 (0.028)	-0.009 (0.013)	0.065 (0.026)*	0.032 (0.021)	-0.005 (0.006)	-0.001 (0.007)	-	-
	13 to 15	0.100 (0.053).	0.058 (0.029)*	-0.027 (0.013)*	0.043 (0.024).	0.021 (0.020)	-0.001 (0.005)	0.004 (0.006)	-	-
	16 to 19	0.098 (0.055).	0.063 (0.029)*	0.003 (0.013)	0.037 (0.025)	0.031 (0.021)	-0.006 (0.006)	0.003 (0.007)	-	-

	**20 to 39**	0.129 (0.043)**	0.047 (0.023)*	-0.002 (0.010)	0.051 (0.021)*	0.037 (0.017)*	0.000 (0.005)	0.000 (0.005)	-	-
	**40 to 69**	0.034 (0.038)	0.007 (0.021)	-0.010 (0.009)	-0.015 (0.019)	0.006 (0.015)	-0.001 (0.004)	-0.006 (0.005)	-	-
	**70 or above**	-0.113 (0.045)*	-0.064 (0.025)*	-0.033 (0.011)**	-0.048 (0.024)*	-0.019 (0.019)	-0.003 (0.005)	-0.005 (0.007)	-	-
**Sex**	**Female**	-	-	-	-	-	-	-	-	-
	**Male**	0.014 (0.023)	-0.009 (0.013)	0.006 (0.006)	0.011 (0.011)	0.020 (0.009)*	-0.000 (0.002)	0.002 (0.003)	-	-
**NS-SEC**	**1**	-	-	-	-	-	-	-	-	-
	**2**	0.022 (0.039)	0.020 (0.022)	-0.013 (0.010)	-0.002 (0.019)	-0.007 (0.015)	0.002 (0.004)	0.007 (0.005)	-	-
	**3**	0.025 (0.041)	0.032 (0.021)	-0.002 (0.009)	0.013 (0.019)	0.017 (0.015)	0.005 (0.004)	-0.010 (0.005)*	-	-
	**4**	-0.086 (0.042)*	-0.019 (0.024)	-0.001 (0.011)	0.026 (0.021)	0.027 (0.017)	-0.003 (0.005)	0.006 (0.006)	-	-
	**5**	0.013 (0.030)	0.030 (0.016).	0.009 (0.007)	0.038 (0.014)**	0.016 (0.012)	0.005 (0.003)	0.002 (0.004)	-	-
	**99**	0.048 (0.072)	0.037 (0.040)	0.028 (0.018)	0.089 (0.034)**	0.065 (0.028)*	0.008 (0.007)	0.006 (0.009)	-	-

		**Breakfast Cereal**			**Crisps and Savoury Snacks**			**Out of Home**		
		**Sugar Snacks**	**Unhealthy Food**	**Sugar and Salt**	**Sugar Snacks**	**Unhealthy Food**	**Sugar and Salt**	**Sugar Snacks**	**Unhealthy Food**	**Sugar and Salt**
**Intercept**		0	0.636 (0.032)***	0.989 (0.010)***	0.404 (0.236)	0.976 (0.009)***	0.999 (0.002)***	0.105 (0.026)***	0	0.478 (0.023)***
**Age Group**	**5 to 7**	-	Ref	Ref	-	Ref	Ref	Ref	-	Ref
	**8 to 10**	-	0.045 (0.037)	-0.003 (0.013)	-	-0.010 (0.011)	0.001 (0.003)	0.024 (0.033)	-	0.014 (0.029)
	**11 to 12**	-	0.111 (0.045)*	-0.024 (0.014).	-	-0.004 (0.012)	0.001 (0.003)	0.041 (0.039)	-	0.022 (0.032)
	**13 to 15**	-	0.193 (0.044)***	-0.002 (0.014)	-	-0.031 (0.012)**	-0.008 (0.003)**	0.100 (0.038)**	-	0.067 (0.031)*
	**16 to 19**	-	0.172 (0.045)***	-0.020 (0.015)	-	0.008 (0.012)	0.001 (0.003)	0.107 (0.043)*	-	0.049 (0.031)
	**20 to 39**	-	0.098 (0.042)*	-0.021 (0.012).	-	-0.005 (0.010)	0.001 (0.003)	0.097 (0.037)**	-	0.043 (0.026).
	**40 to 69**	-	0.098 (0.036)**	-0.032 (0.011)**	-	-0.017 (0.009).	-0.002 (0.003)	0.046 (0.032)	-	-0.015 (0.024)
	**70 or above**	-	-0.056 (0.050)	-0.088 (0.013)***	-	0.016 (0.015)	0.001 (0.004)	0.056 (0.045)	-	-0.115 (0.032)***
**Sex**	**Female**		Ref	Ref		Ref	Ref	Ref		Ref
	**Male**	-	-0.018 (0.022)	0.004 (0.006)	-	0.004 (0.006)	-0.001 (0.002)	0.023 (0.020)	-	0.039 (0.014)**
**NS-SEC**	**1**	-	Ref	Ref	-	Ref	Ref	Ref	-	Ref
	**2**	-	0.052 (0.038)	0.003 (0.011)	-	0.006 (0.010)	0.003 (0.003)	0.021 (0.035)	-	0.072 (0.024)**
	**3**	-	0.072 (0.037).	0.019 (0.011).	-	0.019 (0.009)*	0.003 (0.003)	0.002 (0.033)	-	0.044 (0.024).
	**4**	-	0.069 (0.040).	0.018 (0.012)	-	0.011 (0.011)	0.003 (0.003)	0.064 (0.040)	-	0.087 (0.027)**
	**5**	-	0.057 (0.029)*	0.009 (0.008)	-	0.011 (0.007)	0.001 (0.002)	0.060 (0.026)*	-	0.098 (0.019)***
	**99**	-	0.117 (0.061).	0.035 (0.019).	-	0.014 (0.016)	0.002 (0.004)	-0.000 (0.073)	-	0.082 (0.043).

		**Yoghurt, Fromage Frais and Dairy Dessert**			**Sugar Confectionary**			**Pasta, Rice, Cereals (including Pizza)**		
		**Sugar Snacks**	**Unhealthy Food**	**Sugar and Salt**	**Sugar Snacks**	**Unhealthy Food**	**Sugar and Salt**	**Sugar Snacks**	**Unhealthy Food**	**Sugar and Salt**
**Intercept**		0	0.621 (0.038)***	0.969 (0.011)***	0.967 (0.009)***	0.930 (0.014)***	1.002 (0.003)***	0	0.523 (0.044)***	0.681 (0.022)***
**Age Gro**	**5 to 7**	-	Ref	Ref	Ref	Ref	Ref	-	Ref	Ref
	**8 to 10**	-	0.091 (0.049).	0.005 (0.014)	0.022 (0.011)*	0.015 (0.016)	-0.000 (0.003)	-	-0.068 (0.055)	-0.004 (0.028)

	**11 to 12**	-	0.122 (0.060)*	0.006 (0.017)	0.024 (0.013).	0.042 (0.019)*	-0.003 (0.004)	-	-0.026 (0.066)	0.043 (0.032)
	**13 to 15**	-	0.187 (0.065)**	-0.011 (0.018)	0.015 (0.014)	0.050 (0.021)*	-0.012 (0.004)**	-	-0.032 (0.060)	0.027 (0.030)
	**16 to 19**	-	0.165 (0.065)*	-0.008 (0.020)	-0.001 (0.015)	0.022 (0.022)	-0.000 (0.005)	-	-0.047 (0.060)	0.032 (0.030)
	**20 to 39**	-	0.247 (0.047)***	-0.026 (0.013)*	0.006 (0.013)	0.029 (0.019)	-0.001 (0.004)	-	-0.003 (0.051)	0.026 (0.024)
	**40 to 69**	-	0.162 (0.041)***	-0.032 (0.012)**	0.018 (0.011)	0.054 (0.017)**	-0.001 (0.003)	-	0.065 (0.053)	0.067 (0.024)**
	**70 or above**	-	0.144 (0.050)**	-0.009 (0.015)	0.007 (0.017)	0.043 (0.026).	-0.001 (0.005)	-	0.193 (0.131)	0.066 (0.036).
**Sex**	**Female**	-	Ref	Ref	Ref	Ref	Ref	-	Ref	Ref
	**Male**	-	0.051 (0.028).	0.000 (0.008)	0.012 (0.007).	0.011 (0.011)	-0.002 (0.002)	-	0.074 (0.032)*	0.012 (0.014)
**NS-SEC**	**1**	-	Ref	Ref	Ref	Ref	Ref	-	Ref	Ref
	**2**	-	0.076 (0.044).	0.019 (0.013)	0.008 (0.012)	0.006 (0.017)	0.000 (0.004)	-	0.021 (0.052)	0.013 (0.024)
	**3**	-	0.103 (0.050)*	-0.005 (0.013)	-0.005 (0.011)	-0.015 (0.017)	0.001 (0.004)	-	0.110 (0.052)*	0.049 (0.022)*
	**4**	-	0.049 (0.052)	0.029 (0.014)*	0.007 (0.014)	0.018 (0.021)	0.001 (0.004)	-	-0.055 (0.061)	0.002 (0.026)
	**5**	-	0.060 (0.034).	0.025 (0.010)*	-0.006 (0.009)	0.009 (0.014)	-0.006 (0.003)*	-	0.037 (0.038)	0.032 (0.018).
	**99**	-	0.021 (0.086)	0.006 (0.023)	0.017 (0.022)	0.013 (0.033)	-0.000 (0.007)	-	0.103 (0.089)	0.073 (0.038).

Presented as Coefficient (Standard error), where p-values are indicated by . =0.1, *=0.05, **=0.01, ***=0.001. Food groups where there is not enough data to inform the regression (at least 130 observations above 0), a mean percent is recorded to be applied to all individuals that consumed some taxed food.

Alongside this, a proportion of people who consume the food group that will be affected by the tax is recorded in Table 4.2.3 (column 1). This proportion is high, as seen here, because the population assigned consumption that is the average by demographic group. Individuals in the model population that consume the relevant food group are simulated whether any of this consumption is affected by the tax by comparing the proportions in Table 4.2.3 (columns 3 to 5) to a random number. Food groups not included in any tax policy include: fruit, salad and raw vegetables, milk, other milk and cream, fruit juice, soft drinks (those in question for Sugary Drinks Industry Levy) and tea, coffee and water. For those that are affected by the tax in that food group the regressions coefficients are used to estimate the percentage of grams of food that are taxed. This is used to estimate total consumption of taxed and not taxed in grams for the population.

Table 4.2.3: Proportion of the population impacted by each tax policy by food group.

	**Proportion consuming food group (%)**	**Proportion of those consuming impacted by tax (%)**		
		**Sugar Snacks**	**Unhealthy Foods**	**Sugar and Salt**
Bacon and Ham	100%	0%	0%	100%
Beef Veal and dishes	100%	0%	0%	0%
Beverages dry weight	100%	26%	22%	36%
Biscuits	100%	83%	90%	100%
Breakfast cereals	100%	0%	39%	96%
Brown and wholemeal bread	100%	0%	0%	99%
Butter	100%	0%	0%	87%
Cakes and Puddings	100%	50%	86%	97%
Cheese	100%	0%	0%	99%
Chicken and Turkey dishes	100%	0%	0%	22%
Chips fried & roast potatoes & potato products	100%	0%	3%	83%

Chocolate confectionery	100%	99%	100%	100%
Crisps and savoury snacks	100%	0%	96%	100%
Eggs and Egg dishes	100%	0%	0%	4%
Ice cream	100%	0%	99%	100%
Lamb and lamb dishes	99%	0%	0%	0%
Liver & dishes	93%	0%	0%	64%
Margarine and Oils	100%	0%	0%	1%
Meat pies and pastries	100%	0%	0%	93%
Meat ready meals	100%	0%	9%	92%
Milk drinks	99%	66%	17%	100%
Nuts and seeds	100%	0%	0%	37%
Oily Fish	100%	0%	0%	25%
Other bread	95%	0%	0%	96%
Other meat and meat products	100%	0%	0%	56%
Other milk and Cream	100%	0%	0%	4%
Other potatoes and Potato salads & Dishes	100%	0%	0%	7%
Other white fish Shellfish and Fish dishes	100%	0%	1%	59%
Out of home	100%	29%	0%	94%
Pasta rice and other cereals	100%	0%	16%	70%
Pork and Dishes	100%	0%	0%	0%
Reduced fat spread	100%	0%	0%	91%
Salad and other raw vegetables	100%	0%	0%	2%
Sausages burger and kebabs	100%	0%	0%	98%
Savoury sauces pickles gravies & condiments	100%	0%	0%	85%
Soup homemade and retail	99%	0%	0%	91%
Sugar confectionery	100%	93%	91%	97%
Sugars preserves and sweet spreads	100%	0%	0%	53%
Tea coffee and Water	100%	0%	0%	64%
Vegetable ready meal	100%	0%	4%	95%
White bread	100%	0%	0%	99%
White fish coated or fried	100%	0%	15%	97%
Yoghurt fromage frais and dairy desserts	100%	0%	31%	87%

4.3 Estimate prices

The weighted average price, in pence per gram, was estimated conditional on equivalised income tertile, for each of the 50 lower-level food groups using purchasing and expenditure data from the Family Foods datasets (20). Food groups within the family foods datasets were assigned to each of the 50 food groups in the model by mapping according to the LCF codes in Table 4.4.15. Data by income deciles were combined, weighted by size of each group, to provide prices by income tertiles. Consumption presented in units other than grams were converted to grams following the conversion presented in Table 4.3.1. Mean pence per gram by income groups are presented in Table 4.3.2.

Table 4.3.1: Conversion rates from alternative units of measurement to grams

Food	Unit	Conversion rate (grams per unit)	Source
Milk	ml	1.04	Milk (19)
Cream	ml	1	Density of cream bridged 1 dependent on fat content (19)
Evaporated milk	ml	1.07	Evaporated Milk (19)
Baby Milk/ Dried milk	Eq ml	0.44	
Yoghurt	ml	1.06	
Egg	Per egg	60	Medium egg. egginfo.co.uk/egg-facts-and-figures
Oil	ml	0.92	Olive Oil (19)
Sweet Syrups	ml	1.32	Maple syrup (19)
Ice Cream	ml	0.55	Tub Ice cream
Savoury Sauces and Salad Dressings	ml	1.15	Ketchup
Fruit/Vegetable Juice	ml	1.06	

Table 4.3.2: Mean prices in pence per gram for each food group by equivalised income tertiles from Family Food dataset

	Mean pence per gram		
Food Group	Lowest Income Tertile	Middle Income Tertile	Highest Income Tertile
Bacon and Ham	0.741	0.801	0.885
Beef Veal and dishes	0.862	0.845	0.944
Beverages dry weight	0.980	0.892	0.967
Biscuits	0.454	0.516	0.586
Breakfast cereals	0.310	0.324	0.361
Brown and wholemeal bread	0.130	0.138	0.156
Butter	0.565	0.591	0.628
Cakes and Puddings	0.490	0.509	0.651
Cheese	0.676	0.694	0.772
Chicken and Turkey dishes	0.799	0.826	0.940
Chips fried & roast potatoes & potato products	0.159	0.162	0.190
Chocolate confectionery	0.934	0.982	1.114
Crisps and savoury snacks	0.743	0.783	0.845
Eggs and Egg dishes	0.313	0.322	0.371
Fruit	0.239	0.256	0.289
Fruit Juice	0.118	0.130	0.157
Ice cream	0.310	0.326	0.378
Lamb and lamb dishes	0.994	1.110	1.107

Liver & dishes	0.291	0.476	0.269
Low Fat Milk	0.059	0.058	0.061
Margarine and Oils	0.290	0.337	0.396
Meat pies and pastries	0.555	0.606	0.672
Meat ready meals	0.580	0.632	0.726
Milk drinks	0.225	0.240	0.234
Nuts and seeds	0.793	0.777	0.816
Oily Fish	0.859	1.036	1.228
Other bread	0.341	0.367	0.419
Other meat and meat products	0.649	0.661	0.713
Other milk and Cream	0.220	0.207	0.233
Other potatoes and Potato salads & Dishes	0.092	0.094	0.105
Other white fish Shellfish and Fish dishes	1.810	1.797	1.776
Out of home	1.524	1.646	2.022
Pasta rice and other cereals	0.303	0.326	0.417
Pork and Dishes	0.580	0.501	0.568
Reduced fat spread	0.311	0.341	0.356
Salad and other raw vegetables	0.208	0.215	0.243
Sausages burger and kebabs	0.563	0.605	0.659
Savoury sauces pickles gravies & condiments	0.343	0.402	0.502
Soft drinks low calorie	0.063	0.056	0.065
Soft drink not low calorie	0.070	0.079	0.087
Soup homemade and retail	0.245	0.237	0.266
Sugar confectionery	0.670	0.730	0.873
Sugars preserves and sweet spreads	0.232	0.260	0.344
Tea coffee and Water	0.154	0.171	0.220
Vegetables not raw	0.464	0.532	0.614
Vegetable ready meal	0.161	0.146	0.168
White bread	0.126	0.132	0.143
White fish coated or fried	0.709	0.757	0.863
Whole milk	0.056	0.058	0.065
Yoghurt fromage frais and dairy desserts	0.259	0.268	0.285

4.4 Estimate change in consumption

The change in consumption due price changes within each tax designs was estimated using a hierarchical Almost Ideal Demand System (unpublished data; Wilson L et al). The process follows the concept of multi-stage budget allocation (21). Individuals choose how they allocate food spending across broader food types, for example between meats, savoury foods or dairy. The budget of that broader food categories (described as the higher-level food categories) is then divided across the more granular foods within, for example Breakfast cereals or bread (described as lower-level food groups).

Stage 1: The higher-level price elasticities (Table 4.4.1– Table 4.4.3) of demand determine the revised budget of each higher-level food category by estimating the change in consumption due to the weighted average price change of the food groups within the category and multiplying the updated volume by the new weighted average price.

Stage 2: This revised budget is then shared across lower-level food groups. The budget share within each higher-level category is estimated from the lower-level elasticities (Table 4.4.4 - Table 4.4.14) and individual food group price changes. Own-price elasticities are applied to taxed consumption within a food group only. Cross-price elasticities are applied to all consumption allowing substitution to both taxed and non-taxed food within a food group. The overall consumption change across lower-level groups is estimated by calculating new consumption after sharing out the higher categories budgets and comparing to original consumption.

4.4.1 Almost Ideal Demand System Specification

Data

The price elasticities of demand within the Almost Ideal Demand system were estimated using data from the Living Cost and Food (LCF) survey from 2006 to 2020 (22, 23). The data from the pooled years of the survey contains 84,287 eligible households with aggregated purchasing data over a two-week period including product size or weight and amount spent. The average number of observations per household over the two-week collection period was 103 purchases. Households were divided by household income into tertiles with 28,101, 28,094 and 28,092 households in the lower, middle income and highest income tertiles respectively. Those without relevant data who had not completed a spending diary were excluded.

Food Groups

To estimate the hierarchical demand system, foods were grouped into 55 appropriate lower-level groups, and each lower-level group assigned to overarching higher-level food categories that represents consumption and spending patterns while minimising small samples and the presence of zero purchase observations. The higher-level food categories aim to represent foods types which are directly substituted with each other based on price. For example, you may not expect an individual to directly change their consumption of chicken based on the price of cakes. Therefore prices of these affect each other instead through a broader spending allocation adjustment across higher-level categories of meat and sweet foods, rather than a direct substitution. Products were grouped at the LCF survey 5-level code level.

Prices

Prices in pence per gram of product were estimated from expenditure and quantity purchased. Nominal prices were applied as the regression model controlled for time. To account for endogeneity in prices paid by households, region and period-level price indices are applied to each household (24). The median price per gram for each higher-level food category and each lower-level food group is estimated by government region and time period. This ensures households in the same region at each time face the same median prices and accounts for choices of prices paid.

Model Specification

The uncompensated Almost Ideal Demand System assesses both income and substitution effects (23).As such, the price elasticity of demand matrices considering food, account for a food budget. The higher-level price elasticities are estimated for population groups split by equivalised income tertiles to accommodate difference in price-spending preferences. The demand system was estimated using the demandsys package in Stata and the demandsys aids command. This function imposes homogeneity of degree zero in prices and expenditure (via the constraint that the price coefficients sum to zero across goods within each equation) and Slutsky symmetry directly in the model's parameterisation at estimation time. The adding-up constraint is imposed following Barten (1969): because expenditure shares sum to one by construction, one share equation (in our case, the equation for snacks) is dropped prior to estimation to avoid a singular error covariance matrix, and its parameters are recovered residually from the constraint that the remaining shares' parameters sum appropriately (25). This is the standard approach in AIDS estimation and is invariant to which equation is dropped. The code can be provided upon request.

The multi-stage budgeting system of equations for expenditure share for each household h is presented below. Equation (1) estimates the higher-level food category share.

(1)

$$w_{ih} = \alpha_i + \sum_{k=1}^{K=12} \gamma_{ik} ln(p_k) + \beta_i ln\left(\frac{x_h}{\bar{P}_{i,rmy(h)}}\right) + \sum_{q=1}^{Q} \delta_{iq}\, z_{qh} + \sigma_{ih}$$

Where w_{ih} is the budget share of high-food category i in household h, p_{kh} is the price per gram paid of good k within food category i by household h, x_h is the total expenditure on food in household h, $rmy(h)$ is the region, month and year in which the household h data is collected in the LCFS, P_{iRmy} is the price index for food category i, in Region R, in month m, and year y, and z_{mh} are our q control variables. Control variables included the number of adults in the household, age of the oldest household member, the Government Office Region of residence, and the survey month and year. Standard errors are clustered at the household level.

The 12 higher-level food groups are represented by k. The change in the expenditure share on the higher-level food category i, given a 1% change in price of food category k (including i=k), is presented by γ_{ik}.

The uncompensated demand model in equation (2) estimated the own-price and cross-price elasticity of demand (ε_{ik}^{u}) for the higher and lower-level food groups.

(2)

$$\varepsilon_{ik}^{u} = -\Delta_{ik} + \frac{\gamma_{ik} - \beta_i \bar{w}_k}{\bar{w}_i}$$

Where $\bar{w}_k$ is the sample mean of kth expenditure share and $-\Delta_{ik}$ = 1, for own price elasticities, and $-\Delta_{ik}$ = 0 for cross price elasticities.

The same method estimates the household, h, budget share of the lower-level food groups, j, within each of the 12 higher-level food category i, represented by w_{ijh} in equation (3). The functional form of the regression model is the same as for higher-level food categories shown in equation 1. The equations are estimated separately for each i and j:

(3)

$$w_{ijh} = \alpha_{ij} + \sum_{l=1}^{L} \gamma_{ijl} ln(p_{lh}) + \beta_{ij} ln\left(\frac{x_h}{\bar{P}_{ij,rmy(h)}}\right) + \sum_{s=1}^{S} \delta_{ijs}\, z_{sh} + \sigma_{ijh}$$

Where w_{ijh} is the budget share of lower-food group j, within each higher-level food category i, p_{lh} is the price of good l, where l indexes each of 1 to L lower-level food groups within higher-level food group i, x_h is total food expenditure, P_{ijRmy} is the price index, and z_{sh} are the control variables defined in equation 1.

Sample Selection Bias

The hierarchical system enabled minimisation of zero purchase observations at the higher, more influential level. However, while there were low rates zero purchase rates generally across the primary higher-level categories (Savoury (2%), Meat (6%), Dairy (2%), Fruit (11%), Vegetables (4%), Sweet Foods (8%), Snacks (8%), and Drinks (9%)), we do observe higher rates of zero in some food categories (Fish (44%), Butter and Spreads (32%), Sauces and Spices (21%) and Out-of-Home (56%)), and many food groups at the more granular lower level. To maintain mathematical consistency across the hierarchical demand model, no Heckman correction was applied. However, we recognise the potential selection bias introduced due to the presence of zeros may be a limitation in the demand system.

Uncertainty

Precision was assessed using standard errors and p-values, calculated via Gauss-Newton regression (the default variance estimator within the demandsys package in Stata). Standard errors and statistical significance are reported alongside the price elasticities of demand in Table 4.4.1Table 4.4.14. Price elasticities for the higher-level food categories were sampled assuming a multivariate normal distribution specified by the variance-covariance matrices. Price elasticities for the lower-level substitution effects were not varied in the PSA, and we held at the mean point estimate.

Table 4.4.1: Price elasticity of demand between higher-level food categories for lowest income tertile.

	Savoury	Meat	Fish and Seafood	Dairy	Butter and Fats	Fruit and Nuts	Vegetables	Sweet Foods	Other	Drinks	Out of Home	Snacks
**Savoury**	-1.091***<br>(0.186)	-0.034<br>(0.088)	-0.738**<br>(0.241)	0.179*<br>(0.087)	0.124<br>(0.228)	-0.011<br>(0.124)	-0.046<br>(0.083)	-0.179<br>(0.182)	-0.049<br>(0.299)	0.151<br>(0.136)	0.527*<br>(0.204)	0.069<br>(0.162)
**Meat**	0.004<br>(0.159)	-0.512**<br>(0.163)	-0.858**<br>(0.299)	0.511***<br>(0.119)	-0.333<br>(0.287)	-0.26<br>(0.167)	-0.246*<br>(0.108)	0.027<br>(0.21)	0.043<br>(0.319)	-0.275<br>(0.174)	-1.113***<br>(0.282)	0.283<br>(0.199)
**Fish and Seafood**	-0.265**<br>(0.092)	-0.179**<br>(0.063)	-0.948***<br>(0.238)	-0.068<br>(0.062)	-0.347*<br>(0.16)	0.237**<br>(0.09)	0.203**<br>(0.059)	-0.162<br>(0.124)	0.747***<br>(0.199)	0.151<br>(0.096)	0.121<br>(0.147)	0.12<br>(0.112)
**Dairy**	0.148+<br>(0.077)	0.208***<br>(0.059)	-0.206<br>(0.144)	-1.067***<br>(0.09)	0.566***<br>(0.138)	-0.541***<br>(0.08)	0.124*<br>(0.054)	-0.052<br>(0.096)	0.545***<br>(0.145)	-0.28**<br>(0.084)	-0.563***<br>(0.144)	0.164+<br>(0.092)
**Butter and Fats**	0.03<br>(0.048)	-0.043<br>(0.034)	-0.197*<br>(0.089)	0.141***<br>(0.033)	-0.641***<br>(0.117)	0.004<br>(0.046)	-0.103**<br>(0.032)	0.027<br>(0.065)	-0.268**<br>(0.102)	0.065<br>(0.051)	-0.162*<br>(0.076)	0.134*<br>(0.057)
**Fruit and Nuts**	0.013<br>(0.084)	-0.101<br>(0.063)	0.415**<br>(0.159)	-0.382***<br>(0.061)	0.02<br>(0.148)	-0.327**<br>(0.121)	0.175**<br>(0.062)	0.052<br>(0.111)	-0.269<br>(0.173)	0.186+<br>(0.1)	0.458**<br>(0.151)	-0.771***<br>(0.103)
**Vegetables**	-0.017<br>(0.083)	-0.143*<br>(0.06)	0.519**<br>(0.154)	0.18**<br>(0.061)	-0.476**<br>(0.149)	0.256**<br>(0.091)	-0.658***<br>(0.08)	-0.225*<br>(0.108)	0.065<br>(0.165)	0.303**<br>(0.09)	-0.222<br>(0.152)	-0.551***<br>(0.101)
**Sweet Foods**	-0.11<br>(0.129)	0.002<br>(0.083)	-0.314<br>(0.232)	-0.016<br>(0.076)	0.095<br>(0.217)	0.05<br>(0.117)	-0.164*<br>(0.077)	-0.692**<br>(0.255)	-1.274***<br>(0.307)	0.18<br>(0.129)	0.054<br>(0.2)	0.36*<br>(0.156)
**Other**	-0.002<br>(0.074)	0.007<br>(0.044)	0.489***<br>(0.13)	0.166***<br>(0.041)	-0.311*<br>(0.12)	-0.097<br>(0.064)	0.019<br>(0.041)	-0.443***<br>(0.108)	-0.774**<br>(0.279)	0.146*<br>(0.071)	-0.08<br>(0.101)	-0.043<br>(0.088)
**Drinks**	0.121<br>(0.096)	-0.118+<br>(0.068)	0.267<br>(0.178)	-0.198**<br>(0.066)	0.22<br>(0.171)	0.187+<br>(0.104)	0.21**<br>(0.064)	0.178<br>(0.128)	0.398*<br>(0.202)	-1.132***<br>(0.142)	-0.108<br>(0.162)	-0.358**<br>(0.117)
**Out of Home**	0.3**<br>(0.108)	-0.324***<br>(0.083)	0.168<br>(0.205)	-0.307***<br>(0.086)	-0.395*<br>(0.192)	0.363**<br>(0.118)	-0.112<br>(0.081)	0.049<br>(0.149)	-0.169<br>(0.214)	-0.071<br>(0.122)	-0.246<br>(0.291)	0.123<br>(0.136)
**Snacks**	0.073<br>(0.131)	0.115<br>(0.09)	0.24<br>(0.24)	0.178*<br>(0.084)	0.519*<br>(0.221)	-0.394***<br>(0.124)	-0.456***<br>(0.082)	0.411*<br>(0.178)	-0.156<br>(0.286)	-0.413**<br>(0.135)	0.174<br>(0.209)	-0.517*<br>(0.217)

Standard errors are in parentheses. + $p < 0.1$, * $p < 0.05$), ** $p < 0.01$), *** $p < 0.001$.

Table 4.4.2: Price elasticity of demand between higher-level food categories for middle income tertile.

	Savoury	Meat	Fish and Seafood	Dairy	Butter and Fats	Fruit and Nuts	Vegetables	Sweet Foods	Other	Drinks	Out of Home	Snacks
**Savoury**	-0.578***<br>(0.162)	-0.11<br>(0.077)	-0.387+<br>(0.208)	0.04<br>(0.075)	0.11<br>(0.209)	-0.203*<br>(0.102)	0.04<br>(0.071)	-0.189<br>(0.164)	-0.062<br>(0.253)	-0.051<br>(0.112)	0.204<br>(0.145)	0.035<br>(0.137)
**Meat**	-0.146<br>(0.139)	-0.545***<br>(0.146)	-0.395<br>(0.269)	0.468***<br>(0.11)	-0.416<br>(0.26)	0.208<br>(0.143)	-0.287**<br>(0.096)	0.036<br>(0.192)	0.091<br>(0.274)	-0.131<br>(0.148)	-0.924***<br>(0.21)	-0.042<br>(0.174)
**Fish and Seafood**	-0.142+<br>(0.082)	-0.087<br>(0.058)	-0.728**<br>(0.219)	-0.13*<br>(0.059)	-0.192<br>(0.151)	0.165*<br>(0.078)	0.122*<br>(0.052)	-0.194+<br>(0.115)	-0.207<br>(0.171)	0.186*<br>(0.085)	0.245*<br>(0.113)	-0.016<br>(0.099)
**Dairy**	0.024<br>(0.064)	0.186***<br>(0.052)	-0.315*<br>(0.128)	-0.802***<br>(0.076)	0.495***<br>(0.123)	-0.522***<br>(0.068)	0.044<br>(0.047)	0.2*<br>(0.087)	0.131<br>(0.122)	-0.37***<br>(0.07)	-0.398***<br>(0.107)	0.226**<br>(0.08)
**Butter and Fats**	0.025<br>(0.04)	-0.046+<br>(0.028)	-0.095<br>(0.074)	0.117***<br>(0.028)	-0.502***<br>(0.102)	-0.009<br>(0.036)	-0.088***<br>(0.025)	0.045<br>(0.056)	0.008<br>(0.084)	-0.018<br>(0.041)	-0.1+<br>(0.051)	0.053<br>(0.046)
**Fruit and Nuts**	-0.138+<br>(0.074)	0.071<br>(0.057)	0.296*<br>(0.145)	-0.424***<br>(0.058)	-0.04<br>(0.135)	-0.416***<br>(0.104)	0.211***<br>(0.054)	0.004<br>(0.102)	-0.039<br>(0.148)	0.159+<br>(0.087)	0.065<br>(0.115)	-0.625***<br>(0.09)
**Vegetables**	0.056<br>(0.074)	-0.183**<br>(0.056)	0.313*<br>(0.14)	0.08<br>(0.058)	-0.488***<br>(0.137)	0.307***<br>(0.079)	-0.741***<br>(0.07)	-0.32**<br>(0.099)	-0.087<br>(0.143)	0.438***<br>(0.078)	0.036<br>(0.115)	-0.491***<br>(0.089)
**Sweet Foods**	-0.12<br>(0.113)	0.004<br>(0.073)	-0.349+<br>(0.203)	0.18*<br>(0.07)	0.159<br>(0.203)	0.006<br>(0.097)	-0.21**<br>(0.065)	-0.938***<br>(0.23)	-0.49+<br>(0.257)	-0.123<br>(0.109)	0.244+<br>(0.143)	0.361**<br>(0.132)
**Other**	-0.011<br>(0.071)	0.012<br>(0.042)	-0.149<br>(0.122)	0.053<br>(0.04)	0.013<br>(0.123)	-0.012<br>(0.057)	-0.021<br>(0.038)	-0.197+<br>(0.104)	-0.675**<br>(0.245)	0.112+<br>(0.065)	-0.018<br>(0.078)	0.005<br>(0.081)
**Drinks**	-0.027<br>(0.083)	-0.064<br>(0.06)	0.343*<br>(0.16)	-0.3***<br>(0.06)	-0.071<br>(0.157)	0.164+<br>(0.088)	0.309***<br>(0.055)	-0.132<br>(0.117)	0.289+<br>(0.172)	-0.783***<br>(0.121)	-0.03<br>(0.12)	-0.346**<br>(0.101)
**Out of Home**	0.146<br>(0.095)	-0.344***<br>(0.077)	0.408*<br>(0.189)	-0.287**<br>(0.083)	-0.346*<br>(0.176)	0.063<br>(0.105)	0.025<br>(0.073)	0.235+<br>(0.137)	-0.046<br>(0.184)	-0.025<br>(0.107)	-0.221<br>(0.234)	-0.106<br>(0.121)
**Snacks**	0.041<br>(0.116)	-0.034<br>(0.082)	-0.047<br>(0.216)	0.246**<br>(0.079)	0.228<br>(0.205)	-0.374***<br>(0.106)	-0.399***<br>(0.072)	0.442**<br>(0.163)	0.006<br>(0.245)	-0.4**<br>(0.116)	-0.141<br>(0.156)	-0.401*<br>(0.188)

Standard errors are in parentheses. + $p < 0.1$, * $p < 0.05$), ** $p < 0.01$), *** $p < 0.001$.

Table 4.4.3: Price elasticity of demand between higher-level food categories for highest income tertile

	Savoury	Meat	Fish and Seafood	Dairy	Butter and Fats	Fruit and Nuts	Vegetables	Sweet Foods	Other	Drinks	Out of Home	Snacks
**Savoury**	-0.597***<br>(0.147)	-0.027<br>(0.073)	-0.41*<br>(0.182)	0.061<br>(0.071)	0.611**<br>(0.219)	-0.09<br>(0.087)	-0.111+<br>(0.062)	0.043<br>(0.157)	-0.124<br>(0.212)	0.017<br>(0.102)	-0.331**<br>(0.113)	0.132<br>(0.129)
**Meat**	-0.009<br>(0.128)	-0.384**<br>(0.141)	-0.292<br>(0.24)	0.167<br>(0.105)	-0.291<br>(0.276)	-0.047<br>(0.125)	-0.016<br>(0.086)	-0.027<br>(0.182)	-0.12<br>(0.231)	-0.168<br>(0.136)	-0.661***<br>(0.17)	-0.358*<br>(0.165)
**Fish and Seafood**	-0.16*<br>(0.074)	-0.07<br>(0.056)	-0.791***<br>(0.194)	-0.039<br>(0.057)	-0.075<br>(0.16)	0.115+<br>(0.068)	0.125**<br>(0.047)	-0.246*<br>(0.11)	-0.249+<br>(0.144)	0.14+<br>(0.078)	0.298**<br>(0.09)	-0.108<br>(0.094)
**Dairy**	0.041<br>(0.057)	0.051<br>(0.048)	-0.1<br>(0.111)	-0.71***<br>(0.068)	0.202<br>(0.127)	-0.403***<br>(0.057)	0.013<br>(0.041)	0.056<br>(0.081)	0.014<br>(0.102)	-0.247***<br>(0.062)	-0.125<br>(0.083)	0.223**<br>(0.073)
**Butter and Fats**	0.103**<br>(0.036)	-0.03<br>(0.026)	-0.033<br>(0.065)	0.045+<br>(0.026)	-0.696***<br>(0.107)	-0.033<br>(0.031)	-0.053*<br>(0.022)	-0.036<br>(0.054)	-0.125+<br>(0.071)	0.071+<br>(0.037)	-0.089*<br>(0.04)	0.046<br>(0.043)
**Fruit and Nuts**	-0.067<br>(0.067)	-0.035<br>(0.056)	0.205<br>(0.129)	-0.38***<br>(0.055)	-0.157<br>(0.144)	-0.212*<br>(0.091)	0.024<br>(0.049)	0.106<br>(0.097)	0.063<br>(0.125)	0.03<br>(0.08)	0.099<br>(0.095)	-0.547***<br>(0.085)
**Vegetables**	-0.117+<br>(0.068)	-0.029<br>(0.054)	0.321*<br>(0.125)	0.033<br>(0.056)	-0.352*<br>(0.145)	0.035<br>(0.069)	-0.689***<br>(0.062)	-0.295**<br>(0.094)	-0.024<br>(0.121)	0.38***<br>(0.072)	0.177+<br>(0.095)	-0.644***<br>(0.084)
**Sweet Foods**	0.034<br>(0.098)	-0.016<br>(0.065)	-0.381*<br>(0.167)	0.057<br>(0.063)	-0.131<br>(0.201)	0.09<br>(0.078)	-0.163**<br>(0.053)	-0.865***<br>(0.212)	-0.382+<br>(0.209)	0.015<br>(0.093)	0.064<br>(0.104)	0.257*<br>(0.118)
**Other**	-0.032<br>(0.065)	-0.022<br>(0.041)	-0.187+<br>(0.108)	0.014<br>(0.039)	-0.226+<br>(0.13)	0.029<br>(0.05)	-0.002<br>(0.034)	-0.185+<br>(0.103)	-0.827***<br>(0.205)	0.139*<br>(0.06)	0.009<br>(0.061)	0.131+<br>(0.077)
**Drinks**	0.021<br>(0.075)	-0.079<br>(0.058)	0.247+<br>(0.141)	-0.212***<br>(0.057)	0.317+<br>(0.164)	0.035<br>(0.076)	0.261***<br>(0.049)	0.018<br>(0.11)	0.329*<br>(0.144)	-0.984***<br>(0.111)	-0.2*<br>(0.096)	-0.186*<br>(0.095)
**Out of Home**	-0.248**<br>(0.086)	-0.302***<br>(0.075)	0.551**<br>(0.169)	-0.107<br>(0.08)	-0.41*<br>(0.185)	0.102<br>(0.094)	0.126+<br>(0.066)	0.075<br>(0.128)	0.014<br>(0.153)	-0.211*<br>(0.1)	-0.131<br>(0.201)	-0.094<br>(0.114)
**Snacks**	0.115<br>(0.105)	-0.177*<br>(0.077)	-0.223<br>(0.187)	0.243**<br>(0.075)	0.225<br>(0.213)	-0.569***<br>(0.09)	-0.474***<br>(0.062)	0.335*<br>(0.155)	0.342+<br>(0.205)	-0.207*<br>(0.104)	-0.098<br>(0.121)	-0.411<br>(0.174)

Standard errors are in parentheses. + p < 0.1, * p < 0.05), ** p < 0.01), *** p < 0.001.

Table 4.4.4: Price elasticities of demand of lower-level food groups within Savoury higher-level food category

Savoury	White Sliced Bread	Wholemeal Sliced Bread	Wraps and Rolls (Other Breads)	Breakfast Cereals	Pasta Rice Pizza Noodles	Soup
White Sliced Bread	-0.285*** (0.118)	0.896*** (0.127)	-0.587*** (0.042)	0.027+ (0.06)	-0.143*** (0.034)	-0.217** (0.103)
Wholemeal Sliced Bread	0.649*** (0.091)	-0.581*** (0.132)	-0.184*** (0.039)	0.096*** (0.055)	-0.366*** (0.029)	0.237*** (0.09)
Wraps and Rolls (Other Breads)	-0.812* (0.067)	-0.316** (0.086)	-1.192** (0.048)	-0.189*** (0.047)	0.551*** (0.031)	0.461* (0.09)
Breakfast Cereals	0.169 (0.08)	0.295*** (0.104)	-0.125*** (0.04)	-0.753*** (0.078)	-0.162*** (0.035)	-0.225*** (0.097)
Pasta Rice Pizza Noodles	-0.104 (0.084)	-1.056** (0.1)	0.932*** (0.049)	-0.293* (0.064)	-1.179*** (0.05)	0.44*** (0.105)
Soup	-0.054 (0.052)	0.217 (0.063)	0.166 (0.029)	-0.079 (0.037)	0.095 (0.022)	-1.97 (0.091)

Standard errors are in parentheses. + p < 0.1, * p < 0.05), ** p < 0.01), *** p < 0.001.

Table 4.4.5: Price elasticities of demand of lower-level food groups within Meat higher-level food category

Meat	Beef	Pork	Bacon Ham Gammon	Lamb	Poultry	Offal	Sausages and Burgers	Meat Pies	Other Meats	Meat Ready Meals
Beef	-0.834*** (0.089)	-0.197+ (0.118)	-0.274*** (0.075)	-0.224 (0.152)	-0.047 (0.043)	-0.21 (0.396)	-0.232+ (0.123)	0.546*** (0.146)	0.012 (0.127)	0.259** (0.079)
Pork	-0.114+ (0.06)	-0.986*** (0.17)	0.051 (0.076)	0.112 (0.144)	0.013 (0.04)	0.127 (0.416)	0.134 (0.124)	-0.522*** (0.147)	0.095 (0.104)	0.145+ (0.078)
Bacon Ham Gammon	-0.389*** (0.084)	0.064 (0.169)	-0.479*** (0.136)	0.144 (0.201)	-0.235*** (0.057)	0.617 (0.54)	0.36* (0.165)	-0.018 (0.194)	-0.088 (0.143)	-0.122 (0.109)
Lamb	-0.069 (0.048)	0.082 (0.091)	0.066 (0.057)	-1.039*** (0.161)	0.052+ (0.031)	-0.565+ (0.311)	0.095 (0.094)	0.051 (0.109)	-0.29** (0.086)	-0.056 (0.06)
Poultry	-0.122* (0.062)	0.022 (0.113)	-0.254** (0.073)	0.169 (0.14)	-1.342*** (0.058)	-0.068 (0.363)	-0.829*** (0.112)	-0.233+ (0.131)	0.38*** (0.109)	1.301*** (0.076)
Offal	-0.022 (0.025)	0.009 (0.051)	0.03 (0.03)	-0.121* (0.061)	-0.009 (0.016)	-1.243*** (0.237)	-0.265*** (0.051)	0.253*** (0.06)	-0.021 (0.044)	0.07* (0.032)
Sausages and Burgers	-0.139* (0.06)	0.115 (0.119)	0.162* (0.071)	0.113 (0.143)	-0.286*** (0.037)	-2.012*** (0.396)	-0.417* (0.171)	0.181 (0.142)	0.102 (0.105)	0.002 (0.077)
Meat Pies	0.215** (0.069)	-0.518*** (0.137)	-0.018 (0.081)	0.027 (0.162)	-0.102* (0.043)	1.931*** (0.457)	0.158 (0.138)	-0.805*** (0.231)	-0.188 (0.115)	-0.024 (0.091)
Other Meats	-0.028 (0.04)	0.036 (0.065)	-0.035 (0.04)	-0.325*** (0.086)	0.063** (0.024)	-0.092 (0.225)	0.052 (0.069)	-0.129+ (0.077)	-0.584*** (0.09)	-0.032 (0.044)
Meat Ready Meals	0.209** (0.075)	0.26+ (0.145)	-0.072 (0.091)	-0.213 (0.178)	0.852*** (0.05)	1.163* (0.483)	0.018 (0.15)	0.004 (0.182)	-0.035 (0.132)	-2.577*** (0.131)

Standard errors are in parentheses. + p < 0.1, * p < 0.05), ** p < 0.01), *** p < 0.001.

Table 4.4.6: Price elasticities of demand of lower-level food groups within Fish and seafood higher-level food category

	Oily Fish	Coated/Fried Fish	Other
Oily Fish	-0.714*** (0.064)	0.006 (0.032)	-0.182*** (0.044)
Coated/Fried Fish	-0.184*** (0.044)	-1.312*** (0.045)	0.382*** (0.04)
Other	-0.405*** (0.072)	0.556*** (0.046)	-1.228*** (0.065)
Standard errors are in parentheses. + p < 0.1, * p < 0.05), ** p < 0.01), *** p < 0.001.			

Table 4.4.7: Price elasticities of demand of lower-level food groups within Dairy higher-level food category

	Whole Milk	Semi/Skim	Other Milk	Cheese	Eggs
Whole Milk	-0.407*** (0.097)	0.003 (0.011)	-0.208*** (0.045)	-0.153*** (0.018)	-0.244*** (0.051)
Semi/Skim	0.026 (0.043)	-0.973*** (0.015)	-0.081** (0.026)	-0.131*** (0.011)	0.361*** (0.02)
Other Milk	-0.208*** (0.058)	0.027** (0.009)	-0.862*** (0.053)	0.049** (0.017)	-0.181*** (0.035)
Cheese	-0.516*** (0.088)	0.039** (0.014)	0.185** (0.065)	-0.856*** (0.031)	-0.336*** (0.055)
Eggs	-0.372*** (0.087)	0.196*** (0.009)	-0.276*** (0.046)	-0.155*** (0.019)	-0.567*** (0.067)
Standard errors are in parentheses. + p < 0.1, * p < 0.05), ** p < 0.01), *** p < 0.001.					

Table 4.4.8: Price elasticities of demand of lower-level food groups within Butter and fats higher-level food category

	Butter	Margarine	Olive Oil	Other	Lard
Butter	-1.028***<br>(0.061)	0.07<br>(0.051)	-0.491***<br>(0.07)	0.218**<br>(0.074)	0.18<br>(0.235)
Margarine	-0.142*<br>(0.058)	-0.768***<br>(0.06)	0.196*<br>(0.078)	-0.48***<br>(0.076)	-0.499*<br>(0.248)
Olive Oil	-0.071***<br>(0.019)	0.156***<br>(0.018)	-1.298***<br>(0.043)	-0.118***<br>(0.032)	0.138<br>(0.097)
Other	0.067**<br>(0.023)	-0.07**<br>(0.021)	-0.209***<br>(0.037)	-0.802***<br>(0.053)	0.131<br>(0.118)
Lard	-0.004<br>(0.012)	-0.027*<br>(0.011)	0.002<br>(0.019)	0.008<br>(0.02)	-0.392***<br>(0.094)
Standard errors are in parentheses. + p < 0.1, * p < 0.05), ** p < 0.01), *** p < 0.001.					

Table 4.4.9: Price elasticities of demand of lower-level food groups within Fruit and Nuts higher-level food category

	Fruit	Nuts
Fruit	-0.844***<br>(0.014)	-0.798***<br>(0.07)
Nuts	-0.134***<br>(0.014)	-0.314***<br>(0.07)
Standard errors are in parentheses. + p < 0.1, * p < 0.05), ** p < 0.01), *** p < 0.001.		

Table 4.4.10: Price elasticities of demand of lower-level food groups within Vegetable higher-level food category

	Chips	Other Potato	Salad	Veg Not Raw	Vegetable Ready Meals
Chips	-0.868***<br>(0.096)	-0.222***<br>(0.039)	-0.121***<br>(0.016)	0.113**<br>(0.039)	0.649***<br>(0.082)
Other Potato	-0.299***<br>(0.055)	-0.305***<br>(0.048)	0.033*<br>(0.016)	-0.356***<br>(0.033)	-0.246***<br>(0.056)
Salad	-0.447***<br>(0.082)	0.228***<br>(0.057)	-0.827***<br>(0.028)	-0.168**<br>(0.052)	-0.505***<br>(0.079)
Veg Not Raw	0.238**<br>(0.069)	-0.436***<br>(0.041)	-0.085***<br>(0.018)	-0.401***<br>(0.057)	-0.267***<br>(0.069)
Vegetable Ready Meals	0.646***<br>(0.077)	-0.137***<br>(0.036)	-0.087***<br>(0.014)	-0.121**<br>(0.036)	-0.769***<br>(0.091)
Standard errors are in parentheses. + p < 0.1, * p < 0.05), ** p < 0.01), *** p < 0.001.					

Table 4.4.11: Price elasticities of demand of lower-level food groups within Sweet foods higher-level food category

	Sugar	Preserves	Syrups + Toppings	Ice Cream	Cakes and Puddings	Yoghurts
Sugar	-0.463*** (0.073)	-0.115* (0.058)	-0.291** (0.093)	0.152 (0.098)	-0.087*** (0.014)	0.081*** (0.023)
Preserves	-0.081 (0.061)	-0.932*** (0.124)	-0.867*** (0.14)	-0.558*** (0.134)	0.101*** (0.022)	-0.08** (0.03)
Syrups + Toppings	-0.081* (0.032)	-0.276*** (0.045)	-0.643*** (0.099)	-0.161* (0.072)	-0.042*** (0.011)	0.145*** (0.015)
Ice Cream	0.063* (0.031)	-0.167*** (0.041)	-0.153* (0.067)	-0.593*** (0.092)	-0.014 (0.009)	0.033* (0.015)
Cakes and Puddings	-0.369** (0.12)	0.846*** (0.173)	-1.122*** (0.268)	-0.369 (0.248)	-1.083*** (0.051)	0.147* (0.066)
Yoghurts	0.566*** (0.105)	-0.32* (0.129)	1.883*** (0.204)	0.468* (0.216)	0.083* (0.036)	-1.377*** (0.067)
Standard errors are in parentheses. + p < 0.1, * p < 0.05), ** p < 0.01), *** p < 0.001.						

Table 4.4.12: Price elasticities of demand of lower-level food groups within Other higher-level food category

	Sauces	Spices	Mixed
Sauces	-1.018*** (0.028)	-0.008 (0.056)	0.086 (0.098)
Spices	-0.028* (0.013)	-0.574*** (0.047)	-0.307*** (0.042)
Mixed	0.009 (0.023)	-0.291*** (0.042)	-0.745*** (0.095)
Standard errors are in parentheses. + p < 0.1, * p < 0.05), ** p < 0.01), *** p < 0.001.			

Table 4.4.13: Price elasticities of demand of lower-level food groups within Drinks higher-level food category

	Fruit Juice	Low Cal Soft	Soft	Milk-based	Tea coffee water	Dry Weight
Fruit Juice	-1.196*** (0.028)	0.195*** (0.026)	-0.037*** (0.009)	-0.071 (0.045)	0.036* (0.014)	-0.121 (0.081)
Low Cal Soft	0.221*** (0.028)	-1.226*** (0.094)	0.055*** (0.01)	0.099 (0.063)	0.019 (0.043)	-0.928*** (0.237)
Soft	-0.071*** (0.014)	0.059*** (0.015)	-1.038*** (0.011)	0.021 (0.032)	0.022* (0.009)	0.146** (0.048)
Milk-based	-0.012 (0.009)	0.017 (0.011)	0.005 (0.004)	-0.939*** (0.027)	-0.01+ (0.006)	-0.045 (0.035)
Tea coffee water	0.095** (0.031)	0.035 (0.084)	0.058*** (0.012)	-0.115+ (0.068)	-1.125*** (0.048)	0.413+ (0.248)
Dry Weight	-0.017 (0.011)	-0.119*** (0.03)	0.012** (0.004)	-0.034 (0.025)	0.025 (0.016)	-0.426** (0.15)

Standard errors are in parentheses. + p < 0.1, * p < 0.05), ** p < 0.01), *** p < 0.001.

Table 4.4.14: Price elasticities of demand of lower-level food groups within Snacks higher-level food category

	Biscuits and Crackers	Crisps	Confectionery
Biscuits and Crackers	-1.1*** (0.044)	0.532*** (0.041)	-0.228*** (0.044)
Crisps	0.387*** (0.029)	-1.082*** (0.046)	-0.277*** (0.035)
Confectionery	-0.145** (0.053)	-0.372*** (0.061)	-0.66*** (0.068)

Standard errors are in parentheses. + p < 0.1, * p < 0.05), ** p < 0.01), *** p < 0.001.

4.4.2 Mapping Consumption to Price Elasticities

Consumption across 50 food groups in the population needed to be converted into the 55 food groups presented in the Almost Ideal Demand System. Family foods dataset presents mean purchasing per person across food products (20). Estimating total consumption by food group provided the proportions of each 50 NDNS focused food groups that made up the food groups in the price elasticities of demand. For example, 100% of the NDNS group beef and beef products were assigned to the elasticity group beef. However, the NDNS group margarine and oils were divided between 4 PED food groups including margarine, oils and lard according to proportion consumed of each within the Family Foods datasets. The mapping between the model food groups to the PED food groups, and the LCF codes used to determine the proportion within each are presented in Table 4.4.15.

Table 4.4.15: Mapping food groups within the model to food groups presented in the Demand system.

Model Food Group	Elasticities Food Group	Higher-level food category	LCFS code
**Bacon and Ham**	Bacon Ham Gammon	Meat	1.1.2.3.1, 1.1.2.3.2, 1.1.2.11.2
**Beef Veal and dishes**	Beef	Meat	1.1.2.1.1, 1.1.2.1.2, 1.1.2.1.3, 1.1.2.1.4, 1.1.2.1.5, 1.1.2.1.6
**Beverages dry weight**	Dry Weight	Drinks	1.1.4.3.5, 1.2.1.3.1, 1.2.1.3.2
**Biscuits**	Biscuits and Crackers	Snacks	1.1.1.4.2, 1.1.1.4.4, 1.1.1.4.5, 1.1.1.4.3
**Breakfast cereals**	Breakfast Cereals	Savoury	1.1.1.5.1, 1.1.1.5.2, 1.1.1.5.3, 1.1.1.5.4, 1.1.1.5.5, 1.1.9.3.2
**Brown and wholemeal bread**	Wholemeal Sliced Bread	Savoury	1.1.1.2.1, 1.1.1.2.2
**Butter**	Butter	Butter and Fats	1.1.5.1.1
**Cakes and Puddings**	Cakes and Puddings	Sugar	1.1.4.6.3, 1.1.1.4.1, 1.1.1.6.1, 1.1.1.6.5, 1.1.1.6.6, 1.1.1.6.4, 1.1.1.6.2, 1.1.1.6.3
	Preserves	Sugar	1.1.8.2.3
**Cheese**	Cheese	Dairy	1.1.4.5.1, 1.1.4.5.2, 1.1.4.5.3, 1.1.4.5.4, 1.1.4.5.5, 1.1.4.5.6
**Chicken and Turkey dishes**	Poultry	Meat	1.1.2.11.1, 1.1.2.5.1, 1.1.2.5.2
**Chips fried & roast potatoes & potato products**	Chips	Vegetables	1.1.7.9.2
**Chocolate confectionery**	Confectionery	Snacks	1.1.8.4.1, 1.1.8.4.2
**Crisps and savoury snacks**	Crisps	Snacks	1.1.8.6.1, 1.1.8.6.2
**Eggs and Egg dishes**	Eggs	Dairy	1.1.4.7.1,
**Fruit**	Fruit	Fruit and Nuts	1.1.6.1.1, 1.1.6.1.2, 1.1.6.3.1, 1.1.6.3.2, 1.1.6.3.3, 1.1.6.4.1, 1.1.6.4.2, 1.1.6.2.1, 1.1.6.5.1, 1.1.6.5.2, 1.1.6.8.1, 1.1.6.8.2, 1.1.6.7.1, 1.1.6.6.1
**Fruit Juice**	Fruit Juice	Drinks	1.2.2.4.1
**Ice cream**	Ice cream	Sugar	1.1.8.7.1, 1.1.8.7.2, 1.1.8.7.3
**Lamb and lamb dishes**	Lamb	Meat	1.1.2.4.1, 1.1.2.4.2, 1.1.2.4.3, 1.1.2.4.4

**Liver & dishes**	Offal	Meat	1.1.2.6.1, 1.1.2.6.2, 1.1.2.6.3, 1.1.2.6.4
**Low Fat Milk**	Semi/Skim	Dairy	1.1.4.2.1, 1.1.4.2.2
**Margarine and Oils**	Margarine	Butter and Fats	1.1.5.2.1, 1.1.5.2.2
	Lard	Butter and Fats	1.1.5.5.1
	Olive Oil	Butter and Fats	1.1.5.3.1
	Other Oil	Butter and Fats	1.1.5.4.1
	Lard	Butter and Fats	1.1.5.5.2
**Meat pies and pastries**	Meat Pies	Meat	1.1.2.12.2, 1.1.2.12.1, 1.1.2.12.3
**Meat ready meals**	Meat Ready Meals	Meat	1.1.2.13.1, 1.1.2.13.2
**Milk drinks**	Milk based		1.1.4.6.4
**Nuts and seeds**	Nuts	Fruit and Nuts	1.1.6.7.2, 1.1.6.7.3
**Oily Fish**	Oily Fish	Fish and Seafood	1.1.3.2.3, 1.1.3.2.4, 1.1.3.2.1, 1.1.3.2.2, 1.1.3.4.2, 1.1.3.5.1, 1.1.3.5.2
**Other bread**	Wraps and Rolls	Savoury	1.1.1.3.1, 1.1.1.3.2, 1.1.1.3.3, 1.1.1.3.4, 1.1.1.3.6, 1.1.1.3.5
**Other meat and meat products**	Offal	Meat	1.1.2.6.5
	Other Meats	Meat	1.1.2.10.1, 1.1.2.11.3, 1.1.2.10.2, 1.1.2.5.3, 1.1.2.7.1
	Offal	Meat	1.1.2.9.1, 1.1.2.9.2
**Other milk and Cream**	Other Milk	Dairy	1.1.4.3.1, 1.1.4.3.2, 1.1.4.3.3, 1.1.4.3.4, 1.1.4.6.5, 1.1.4.6.1, 1.1.4.6.2
**Other potatoes and Potato salads & Dishes**	Other Potato	Vegetables	1.1.7.4.2, 1.1.7.4.4, 1.1.7.4.1
	Chips	Vegetables	1.1.7.9.1
	Other Potato	Vegetables	1.1.7.8.5
	Chips	Vegetables	1.1.7.9.3
**Other white fish Shellfish and Fish dishes**	Other Fish	Fish and Seafood	1.1.3.1.1, 1.1.3.1.2, 1.1.3.4.1, 1.1.3.3.1, 1.1.3.3.2
**Out of home**	Out of home	Out of Home	1.3.1.1.1, 1.3.1.1.2, 1.3.1.3.1, 1.3.1.3.2, 1.3.1.3.3, 1.3.1.4.1, 1.3.1.3.4, 1.3.1.1.3, 1.3.1.1.4, 1.3.1.4.2, 1.3.1.2.1, 1.3.1.5.1, 1.3.1.7.1, 1.3.1.2.4, 1.3.1.2.5, 1.3.1.2.2, 1.3.1.2.3, 1.3.1.6.1, 1.3.1.8.2, 1.3.1.8.6, 1.3.1.8.5, 1.3.1.8.7, 1.3.1.8.4, 1.3.1.8.3, 1.3.1.8.1
**Pasta rice and other cereals**	Pasta Rice Pizza Noodles	Savoury	1.1.1.8.3, 1.1.1.8.1, 1.1.1.8.2, 1.1.9.3.3, 1.1.1.7.1, 1.1.1.7.2, 1.1.1.7.3, 1.1.1.7.4, 1.1.1.7.5, 1.1.1.8.4
**Pork and Dishes**	Pork	Meat	1.1.2.2.1, 1.1.2.2.2, 1.1.2.2.3, 1.1.2.2.4
**Reduced fat spread**	Margarine	Butter and Fats	1.1.5.2.3, 1.1.5.2.4

**Salad and other raw vegetables**	Salad	Vegetables	1.1.7.2.1, 1.1.7.2.2, 1.1.7.2.3, 1.1.7.1.1, 1.1.7.1.4, 1.1.7.3.3, 1.1.7.3.4, 1.1.7.1.2, 1.1.7.5.1, 1.1.7.5.2, 1.1.7.5.4, 1.1.7.5.3, 1.1.7.3.1, 1.1.7.5.5, 1.1.7.3.5, 1.1.7.5.6, 1.1.7.1.3, 1.1.7.3.2
	Spices	Vegetables	1.1.9.2.3
**Sausages burger and kebabs**	Sausages and Burgers	Meat	1.1.2.8.1, 1.1.2.8.2, 1.1.2.8.4
	Other Meats	Meat	1.1.2.8.3
**Savoury sauces pickles gravies & condiments**	Sauces	Other	1.1.9.1.3, 1.1.9.1.1, 1.1.9.1.2
	Mixed	Other	1.1.9.6.2, 1.1.9.2.1
**Soft drinks low calorie**	Low Cal Soft	Drinks	1.2.2.3.2, 1.2.2.2.2
**Soft drink not low calorie**	Soft	Drinks	1.2.2.3.1, 1.2.2.2.1
**Soup homemade and retail**	Soups	Savoury	1.1.9.5.1, 1.1.9.5.2
**Sugar confectionery**	Confectionery	Snacks	1.1.8.5.1, 1.1.8.5.2, 1.1.8.5.3, 1.1.8.5.4
**Sugars preserves and sweet spreads**	Sugar	Sweet foods	1.1.8.1.1
	Preserves	Sweet foods	1.1.8.2.1
	Preserves	Sweet foods	1.1.8.2.2
	Syrups and Toppings	Sweet foods	1.1.8.3.1
	Preserves	Sweet foods	1.1.8.2.4
	Sugar	Sweet foods	1.1.8.3.2
**Tea coffee and Water**	Tea coffee and Water	Drinks	1.2.1.2.1, 1.2.1.1.1, 1.2.1.1.2, 1.2.1.1.3, 1.2.2.1.1
**Vegetables not raw**	Vegetables not raw	Vegetables	1.1.7.8.1, 1.1.7.8.2, 1.1.7.8.3, 1.1.7.8.4, 1.1.7.8.6, 1.1.7.7.2, 1.1.7.7.1, 1.1.7.8.7, 1.2.2.5.1, 1.1.7.6.1, 1.1.7.6.2, 1.1.7.6.3, 1.1.9.4.1
**Vegetable ready meal**	Vegetable ready meal	Vegetables	1.1.7.10.1, 1.1.9.3.1
**White bread**	White Sliced Bread	Savoury	1.1.1.1.1, 1.1.1.1.2, 1.1.1.1.3, 1.1.1.1.4
**White fish coated or fried**	Coated Fried Fish	Fish and Seafood	1.1.3.6.1
**Whole milk**	Other Milk	Dairy	1.1.4.1.3
	Whole Milk	Dairy	1.1.4.1.2, 1.1.4.1.1
**Yoghurt fromage frais and dairy desserts**	Yoghurts	Sugar	1.1.4.4.1, 1.1.4.4.2

4.5 Estimating Net Tax Spending and Revenue

The estimate tax spending per person is estimated at baseline, including value added tax (VAT) and Soft Drinks Industry Levy (SDIL), and after each tax policy in applied, including changes in VAT and SDIL and the new added tax. VAT is estimated assuming a 20% tax on all food within the food groups: biscuits, chocolate confectionary, crisps and savoury snacks, ice cream, out of home, sugar confectionary and yoghurt fromage frais and dairy desserts. To estimate the amount spent on SDIL, we first estimated a weighted mean tax rate is based on previously reported proportions of soft drinks eligible for the tax at each tax rate, after reformulation (not considering drinks that were never included in SDIL, ie starting below 5g/ 100ml) (26). This rate is applied to non-taxed food in the food group 'soft drink not low calorie'. The weighted mean tax rate is presented in Table 4.5.1.

Table 4.5.1: Weighted mean tax rate in Soft Drinks Industry Levy uprating (3).

	Tax rate (Pence per gram)	Proportion
Above 8g/100ml	0.0194	29%
5 to 8g /100ml	0.0259	21%
Below 5g / 100ml	0	50%
**Weighted Tax Rate**	**0.011065**	

Tax spent under each tax policy is calculated based on the change in price and the amount of consumption of taxed foods, by food group, after the demand model is applied. Individual net spending on tax is estimated for each tax policy by combining the changes in VAT and SDIL spent after consumption has changed, plus the additional food tax. Net tax revenue is estimated by scaling the per-person changes in tax spending to the UK population. An indicator of tax burden is estimated by finding the proportion of total baseline food spending, used as a proxy for food budget, spent on tax.

4.6 Estimating the change in nutritional intake

The nutritional components of each food group were determined using food-level NDNS data for taxed and non-taxed foods separately. A weighted mean nutrient content per gram was recorded for each food group in Table 4.6.1. For each individual, based on their estimated change in consumption, the mean nutrient content of each food group was used to estimate their total change in: calories (energy kcal), carbohydrate, sodium, saturated fat, unsaturated fat, fibre, and total sugar.

Table 4.6.1: Mean energy in calories (kcal) per gram of food by food group, separately for taxed and non-taxed foods by policy design.

Policy	Sugar Snacks Tax		Unhealthy Food Tax		Sugar and Salt Tax	
	**Not Taxed**	**Taxed**	**Not Taxed**	**Taxed**	**Not Taxed**	**Taxed**
**Bacon and Ham**	1.75		1.75			1.75
**Beef Veal and dishes**	1.45		1.45		1.45	1.36
**Beverages dry weight**	2.19	2.20	1.33	3.83	1.02	2.47
**Biscuits**	4.46	4.58	4.29	4.60	4.56	4.55
**Breakfast cereals**	2.66		2.32	3.94	0.03	3.22
**Brown and wholemeal bread**	2.49		2.49		0.42	2.51
**Butter**	7.32		7.32		7.44	7.31

**Cakes and Puddings**	2.59	3.88	1.86	3.41	2.78	2.90
**Cheese**	3.56		3.56		2.71	3.59
**Chicken and Turkey dishes**	1.61		1.61		1.54	2.05
**Chips fried & roast potatoes & potato products**	1.85		1.84	2.33	1.54	1.96
**Chocolate confectionery**	5.69	5.03		5.03		5.03
**Crisps and savoury snacks**	5.06	4.75	4.25	5.12	4.63	5.06
**Eggs and Egg dishes**	1.52		1.52		1.50	1.97
**Fruit**	0.60		0.60		0.60	
**Fruit Juice**	0.37		0.37		0.37	
**Ice cream**	2.15	3.49	1.05	2.17	0.00	2.15
**Lamb and lamb dishes**	1.81		1.81		1.81	
**Liver & dishes**	2.19		2.19		1.42	2.79
**Low Fat Milk**	0.43		0.43		0.43	
**Margarine and Oils**	8.78		8.78		8.81	6.61
**Meat pies and pastries**	3.02		3.02		2.32	3.09
**Meat ready meals**	1.53		1.49	2.15	1.33	1.55
**Milk drinks**	0.40	0.79	0.59	0.90		0.64
**Nuts and seeds**	4.18	3.80	4.18		3.60	5.83
**Oily Fish**	2.17		2.17		2.21	2.02
**Other bread**	2.32		2.32		0.00	2.36
**Other meat and meat products**	2.38		2.38		2.28	2.47
**Other milk and Cream**	0.76		0.76		0.75	5.40
**Other potatoes and Potato salads & Dishes**	0.80		0.80		0.78	1.11
**Other white fish Shellfish and Fish dishes**	1.07		1.06	2.49	0.90	1.25
**Out of home**	1.16	2.30	1.21		0.55	2.13
**Pasta rice and other cereals**	1.78		1.68	2.66	1.82	1.75
**Pork and Dishes**	1.97		1.97		1.97	
**Reduced fat spread**	5.00		5.00		4.92	5.01
**Salad and other raw vegetables**	0.28		0.28		0.27	1.23
**Sausages burger and kebabs**	2.59		2.59		2.05	2.62
**Savoury sauces pickles gravies & condiments**	1.38		1.38		0.63	1.80
**Soft drinks low calorie**	0.01		0.01		0.01	
**Soft drink not low calorie**	0.29		0.29		0.29	
**Soup homemade and retail**	0.39		0.39		0.34	0.39
**Sugar confectionery**	1.52	3.02	2.81	2.98	0.00	2.96
**Sugars preserves and sweet spreads**	3.65		3.65		3.85	3.39

**Tea coffee and Water**	0.01		0.01		0.01	
**Vegetables not raw**	0.49		0.49		0.38	0.76
**Vegetable ready meal**	0.95		0.92	1.75	0.90	0.96
**White bread**	2.61		2.61	3.25	1.20	2.63
**White fish coated or fried**	2.21		2.16	2.43	1.52	2.22
**Whole milk**	0.65		0.65		0.65	
**Yoghurt fromage frais and dairy desserts**	0.90	4.66	0.74	1.44	0.65	0.94

4.7 Estimating the change in metabolic health

4.7.1 Weight

4.7.1.1 Adults

Weight change in conditional on changes in calories, based on the Hall et al. equations (27). To be compatible with the structures, inputs, and computational demands of the SPHR Diabetes Prevention model we approximate the Hall et al equations, using the NIDDIK online tool, to estimate the impact that other characteristics have on weight changes for a given change in calories (28).

We estimated weight change conditional on change in calories, % carbohydrate, sodium intake, considering demographic characteristics. Based on the online tool we estimated the impact that an increase in calories had on weight, and the relationship was slightly stronger for females, higher baseline weight and age. Changes in % carbohydrate and sodium also increase weight change. Physical activity and height are included in the Hall weight change calculation at default values. For the purposes of our simulation physical activity and height are assumed to stay constant and do not impact on the weight change estimation We used regression analyses based on the data from the NIDDIK online tool to approximate the impact of change in calories, sodium, % carbohydrate, sex, age and baseline weight on change in weight. The final parameters and precision of our estimates are reported in Table 4.7.1. Parameters were sampled according to a multivariate normal distribution defined by the variance-covariance matrix of the regression.

All coefficients were statistically significantly associated with changes in weight, and the model reported good fit to the data.

This model includes a natural trajectory of weight. Instead, the model requires a change in weight as a result of consumption only. As a results, only the relevant nutritional components were used to simulate weight change conditional on change in Energy kcal, carbohydrate, and sodium intake, presented in Table 4.7.1.

Table 4.7.1: Estimated change in weight for a given change in calories, % carbohydrate and sodium

	**Mean**	**Standard error**
Change in calories	0.024161	0.0001778
Change in calories by baseline weight	0.000012	1.70e-06
Change in calories by age	0.000021	2.06e-06
Change in calories by male	-0.000941	0.000082
Changes in % carbohydrate	0.033863	0.0004127
Changes in sodium	0.000243	0.0000206

4.7.1.2 *Children*

The resulting weight change from a change energy in kilocalories consumed in children under 18 years old was simulated using validated model of childhood energy balance (29). The model provides a sex specific relationship between weight and energy kilocalories conditional on age.

$$Male: kcal\ per\ day\ per\ kg = 68 - 2.5 * age$$

$$Female: kcal\ per\ day\ per\ kg = 62 - 2.2 * age$$

These equations were developed for ages 7-18 years, however, they are applied to ages 5-17 in the model. The parameters used within this equation are presented in Table 4.7.2. Parameters were sampled from a normal distribution in which standard errors are assumed to be 10% of the mean.

Table 4.7.2: Model parameters for calorie to weight change in children

	Mean coefficient	Derived SE	Distribution
Male Intercept	68	6.80	Normal
Male β	2.5	0.25	Normal
Female Intercept	62	6.20	Normal
Female β	2.2	0.22	Normal

4.7.2 Other metabolic outcomes

The association between changes in nutritional intake and cardiometabolic risks was identified in an umbrella review (30).

All associations identified in this review with a statistically significant relationship and assessed to be based on a high certainty of evidence were included in the dietary change model. Relationships meeting these criteria for sub-group populations (type 2 diabetes, hypertension) were also included. In some cases, a statistically significant relationship was identified, but not consistently reported across studies to meet the criteria for high certainty evidence. A critical appraisal of the individual studies was taken to determine whether these relationships should be included as part of the review. In some cases, individual studies were selected if they were determined to be of superior quality to the contractor evidence. Where multiple sources of evidence for the association were identified a process of deliberation was used to identify the mean estimate to include in the model. Due to the number of relationships to include it was not possible to pool the results from multiple studies in a statistical analysis. For each relationship considered, the data from the meta-analysis was converted into a linear relationship between exposure and metabolic outcome.

Table 4.7.3 reports the linear relationship parameters for nutrients and systolic blood pressure. The table indicate whether the relationship is conditional on the type of nutrient substituted. For example, the significant relationship between monounsaturated fats and systolic blood pressure is observed where carbohydrates are being replaced. Other substitutions, such as those away from polyunsaturated fats may not result in the same benefits to individuals. Parameter samples were taken according to a normal distribution specified by their respective standard errors.

In some instances, the evidence is based on the volume of a nutrient consumed (sodium, fibre), however in others the studies have assessed whether the proportion of a nutrient as the percentage of Total Energy Intake (TEI), is substituted between nutrient types will impact health. For example, a study in which saturated fat is replaced with mono-unsaturated fats. In this case the change across nutrients is based on the simulated change in % TEI, rather than change in grams per day.

Table 4.7.3: The association between changes in nutrient intake to metabolic risks assumed in the model

Nutrient	Population characteristics		Mean change in weight (kg)	Standard error	Mean change in blood pressure	Standard error	Mean change in total cholesterol	Standard error	Mean change in HDL cholesterol	Standard error	Mean change in HbA1c	Standard error	Source
Calories (kcals)	General Population		0.02416	0.0002									(27)
Sodium (mg)	Non-hypertensive	White			0.00035	0.00008	-0.00005	0.00002					(31)
		Black			0.00136	0.00058							
		Asian			0.00057	0.00031							
	Hypertensive	White			0.00177	0.00015	-0.00003	0.00002					
		Black			0.00224	0.00041							
		Asian			0.00296	0.00072							
	General population		0.00024	0.00002									(27)
Monounsaturated fat vs carbohydrate (%TEI)	Type 2 diabetes				-0.16071	0.06851							(32)
	General population										-0.02400	0.00714	(33)
Polyunsaturated fat vs carbohydrate (g)	Non-hypertensive				-1.18684	0.21617							(34)
	Hypertensive				-0.32895	0.10741							
Polyunsaturated fat vs unspecified substitute (%TEI)	General population						-0.04528	0.02118					(35)
Saturated fat vs polyunsaturated fat (%TEI)	General population						0.06600	0.00357	0.00500	0.00102			(36)
Saturated fat vs monounsaturated fat (%TEI)	General population						0.04900	0.00306	0.00300	0.00051			(36)
Saturated fat vs carbohydrate (%TEI)	General population						0.04500	0.00306	-0.00501	0.00128			(36)
Carbohydrate vs unspecified substitute (%TEI)	General population		0.03386	0.00041			-0.00626	0.00288	-0.00501	0.00128			(27, 37)
Fibre (g)	General population				-0.09319	0.04605	-0.01172	0.00279					(38)
	Type 2 diabetes										-0.05596	0.01498	(39)
Sugar vs starch (%TEI)	General population						0.01150	0.00281	0.00100	0.00051			(40)

4.7.2.1 *Indirect metabolic effects through weight*

An additional mechanism of change to metabolic risk factors, other than diet alone, is through a weight effect. A previous analysis defined the relationship between weight change and changes in systolic blood pressure by type 2 diabetes status and cholesterol levels (41). This effect is added to the changes as a result of diet. Table 4.7.4 reports the relationship between a 1 kg change in weight and these outcomes. Samples of these parameters were taken assuming a normal distribution specified by their respective standard errors.

Table 4.7.4: Relationship between changes in weight and changes in wider metabolic risk factors

1 kg change in weight relates to	Systolic Blood Pressure (mm Hg)		Total Cholesterol (mmol/l)	
	**Mean**	**SE**	**Mean**	**SE**
Normoglycemia	0.77	0.16	0.03	0.01
Non-diabetic hyperglycaemia	1.3	0.4		
Type 2 diabetes	1.33	0.28		

4.8 Industry Response

4.8.1 Tax Pass Through

Evidence suggests that industry chooses to vary how much of the tax they pass onto consumers dependent on the type of product, more specifically the price of the product. An analysis of the Kantar's Worldpanel (2006 to 2013) data found that industry opted to increase the price by less than the full tax amount in products of a lower price but increase the prices by more than the tax in more expensive products (Wilson L, University of Sheffield, unpublished). Table 4.8.1 reports the percentage pass through rate by price quantiles. The price change estimated by the tax was adjusted based on the pass-through estimate associated with the quantile of that individuals' prices.

Table 4.8.1: The percentage tax-passthrough by the quantile of price.

Price Quantile	Pass through (%)
0.05	0.6582
0.15	0.8436
0.25	0.9112
0.35	0.9514
0.45	0.9754
0.5	0.9833
0.55	0.9895
0.65	0.9984
0.75	1.0209
0.85	1.0710
0.95	1.1889

Prices by food group in the model are conditional on income tertile. To estimate price quantile by income groups, prices by equivalent food groups was estimated from the LCF Survey (22). The most common price quantile within each income group was used to define the pass-through rate. Where prices didn't significantly differ between income groups, the same quantile was used. Where multiple modes were found, the lowest quantile of the modes was chosen. One mode contained fewer than 10 observations and was redacted (denoted -). The mode across income tertile 1 and 2 was used for income tertile 3 where the quantile was redacted. The out of home food group was assumed to have the median pass-through rate.

Table 4.8.2: Most common quantile of purchase price by equivalised income tertiles for each food group.

Food group	Most common quantile of Pence per gram by Income			Food group	Most common quantile of Pence per gram by Income		
	Tertile 1	Tertile 2	Tertile 3		Tertile 1	Tertile 2	Tertile 3
**White Sliced Bread**	8	8	12	**Fruit**	2	2	20
**Wholemeal Sliced Bottom**	1	7	1	**Nuts**	1	5	19
**Wraps and Rolls (Other Breads)**	7	3	14	**Chips**	3	3	20
**Breakfast Cereals**	17	17	19	**Other Potato**	9	9	12
**Pasta Rice Pizza Noodles**	8	8	3	**Salad**	2	3	20
**Soups**	11	11	11	**Veg Not Raw**	6	4	20
**Beef**	8	18	20	**Vegetable Ready Meals**	1	1	20
**Pork**	16	11	16	**Sugar**	3	3	3
**Bacon Ham Gammon**	7	7	20	**Preserves**	1	1	18
**Lamb**	12	12	20	**Syrups and Toppings**	9	9	9
**Poultry**	4	4	20	**Ice Cream and Ice Lollies**	1	1	1
**Offal**	6	3	20	**Cakes and Puddings**	1	11	20
**Sausages and Burgers**	1	19	16	**Yoghurts**	18	18	7
**Meat Pies**	2	8	20	**Savoury Sauces**	5	1	20
**Other Meats**	1	3	19	**Spices**	1	1	16
**Meat Ready Meals**	1	13	18	**Mixes**	14	14	14
**Oily Fish**	2	2	18	**Fruit Juice**	8	8	11

**Coated/Fried Fish**	1	1	19	**Low Cal Soft**	12	12	12
**Other white fish**	9	11	20	**Soft Drinks**	9	11	15
**Whole Milk**	3	3	3	**Milk**	2	2	15
**Skimmed and Semi-Skimmed Milk**	3	3	3	**TCW**	10	10	20
**Other Milk and Cream**	1	2	6	**Dry**	1	1	17
**Cheese**	13	2	20	**Biscuits and Crackers**	4	9	20
**Eggs**	5	13	13	**Crisps**	8	8	7
**Butter**	5	5	5	**Chocolate Confectionery**	12	12	12
**Margarine**	5	5	19	**Sugar Confectionery**	10	14	4
**Olive Oil**	15	15	19				
**Other Oil**	6	6	20				
**Lard**	4	4	-				

4.8.2 Reformulation

When industry responds to taxation through product reformulation, the tax will change individual's nutritional intake. The first is through changing the nutritional content of the foods they are producing. Food level consumption data is collated by individuals using food level data from NDNS years 9-11 (19). Additional detail provided in these later waves, within the RecipeName variable, enabled more accurate identification of eligible foods. Additionally, later waves capture some existing progress towards voluntary reformulation that occurred across this time frame. To understand the impact of a potential food tax, we simulated reformulation of calories, salt, and sugar for a subset of food groups. The reformulation scenario draws on a review of the government's voluntary reformulation program and a NESTA study of UK reformulation efforts (5, 42).

For each food group and nutrient, we specified a maximum reformulation amount. This was calculated by taking the government's current reformulation target, applying it to the initial nutritional content. For foods not included in the relevant nutrient-based reformulation programme we assumed no reformulation for that food group. We subtracted observed reformulation already achieved according to the latest government reformulation reports to determine potential reformulation across sugar, salt and calories. We assumed a reformulation feasibility level to indicate the practicality of reformulation for each nutrient within each food group, which were assigned to "moderate", "low" or "very low" feasibility score. Evidence for the feasibility of reformulation was limited, with a few isolated sources indicating the reformulation potential for individual food groups such as confectionery and ice cream, which indicated some potential methods for sugar reformulation (43, 44). As such, we assumed the feasibility for sugar reformulation was moderate. The feasibility of calorie was based on analysis reported in the NESTA study (42). All foods were considered "moderate" for salt reformulation based on observational studies demonstrating the progress of reformulation efforts on salt, and in recognition of the potential use of low-sodium substitutes in manufacturing, for which price is an identified barrier to implementation (45, 46). For food groups where reformulation was deemed "very low", we assumed food taxes have no additional impact on reformulation. We also set upper limits on potential reformulation: 5% for food groups with "low" feasibility and 10% for those with "moderate" feasibility.

In the sugar snacks tax design only sugar reformulation was implemented. For the unhealthy food tax calorie, salt and sugar reformulation was assumed. In the sugar and salt tax design sugar and salt reformulation was implemented. In each design reformulation effects were applied before the proportion of foods eligible for the tax were exported. The reformulation rates indicated in Table 4.8.3 were implemented for all foods. All foods were assessed against the food tax threshold or criteria, where reformulation would impact on tax eligibility or rate the reformulation effects were retained, however if reformulation did not impact the tax burden, reformulation was not implemented.

Table 4.8.3: Feasibility, reformulation potential and resulting reformulation implemented in model scenario for calorie, salt and sugar content.

	Feasibility			Reformulation Potential (%)			Final reformulation scenario (%)		
	**Calorie**	**Salt**	**Sugar**	**Calorie**	**Salt**	**Sugar**	**Calorie**	**Salt**	**Sugar**
Beverages dry weight	Moderate	Moderate	Moderate	6%	-5%	-12%	0%	-5%	-12%
Milk drink	Moderate	Moderate	Moderate	6%	-5%	-12%	0%	-5%	-12%
Crisps and savoury snacks	Very low	Moderate	Moderate	-9%	-27%	0%	0%	-20%	0%
Breakfast cereals	Low	Moderate	Moderate	0%	0%	-6%	0%	0%	-6%
Chocolate confectionery	Very low	Moderate	Moderate	-7%	0%	-19%	0%	0%	-19%
Ice cream	Low	Moderate	Moderate	-3%	0%	-14%	-3%	0%	-14%
Cakes and puddings	Moderate	Moderate	Moderate	-6%	-6%	-17%	-6%	-6%	-17%
Biscuits	Very low	Moderate	Moderate	-8%	-13%	-17%	0%	-13%	-17%
Yogurt fromage frais and dairy desserts	Moderate	Moderate	Moderate	-3%	0%	-8%	-3%	0%	-8%
Pasta rice and other cereals	Low	Moderate	Moderate	-9%	-10%	0%	-5%	-10%	0%
Chips fried & roast potatoes and potato products	Low	Moderate	Moderate	-12%	-2%	0%	-5%	-2%	0%
Meat ready meal	Low	Moderate	Moderate	-11%	-2%	0%	-5%	-2%	0%
Other white fish shellfish & fish dishes	Low	Moderate	Moderate	-11%	-2%	0%	-5%	-2%	0%
Sugar confectionery	Very low	Moderate	Moderate	-14%	0%	-18%	0%	0%	-18%
Veg ready meal	Low	Moderate	Moderate	-11%	-2%	0%	-5%	-2%	0%
White fish coated or fried	Low	Moderate	Moderate	-11%	-2%	0%	-5%	-2%	0%

The overall change in an individual's baseline nutritional intake was adjusted based on these changes in foods. Average nutritional content per gram of food was also updated.

In the reformulation scenarios, individual nutritional intake is also affected by the price response. This effect is considered to be the primary mechanism through which taxes can impact consumer behaviours and nutritional intake. However, when considering reformulation, fewer foods meet the criteria to be affected by the tax and increase prices. Therefore, the price effect alone will be diminished, but it is additive to the reformulation effect. Scenarios were performed considering each industry response, tax pass-through (I1) and reformulation (I2), separately and an analysis with both together (I3). The combined industry response scenario (I3) was also estimated across sensitivity analyses. Table 1.7.1 presents the mean change in baseline nutrient intake due to reformulation alone, without considering consumption changes driven by the tax.

4.9 Sensitivity Analyses

The model inputs are altered across a series of analyses. Combinations of up to two of these alterations were performed. These sensitivity analyses were also performed on the analysis accounting for industry responses. A description of the sensitivity analyses and additional parameters inputs are provided below.

4.9.1 S1 Controlling for non-food spending

An alternative Almost Ideal demand system structure included substitution and income effects considering an individual's total spending behaviour. Decisions relating to spending on food may consider non-food spending. The population may change non-food expenditure to accommodate changes in food expenditure across higher level food groups in response to the taxes. The alternative higher-level price elasticity of demand, controlling for consideration of non-food spending is provided in Table 4.9.3.

4.9.2 S2 Removing non-significant PED

In this analysis, any price elasticities that were not found to be statistically significant at the 10% level are replaced with 0. Consequently, the PED values retained in this analysis are those with a p-value less than 0.1, indicated by a +, *, ** or *** symbol in Table 4.4.1 to Table 4.4.14.

4.9.3 S3 Applying higher-level food price elasticities only

This analysis removes the lower-level price elasticities such that consumption change only occurs through changes in weighted averages prices of broader higher level food categories. The distribution of spending across food groups (at lower level) within these food categories remains constant.

4.9.4 S4 No income variation in PED

Consumer response to prices is assumed to be the same across all incomes. A single higher-level elasticity matrix that was estimated from the whole population was applied to the model population (Table **4.9.4**).

4.9.5 S5 Removing cross-price elasticities

In this analysis, we assume no substitution due to the price of one good to the consumption of another. All cross-price elasticities are removed, such that a change in consumption can only respond to its own price change, and is not impact by price changes in other food groups. However, the hierarchical PED structure means that some reallocation of consumption within a food group (i.e. to non-taxed foods) can occur. Consumption of lower-level food groups changes according to the own-PEDs at the lower level. As a result, the proportion of expenditure across the lower food groups within each higher-level food category can shift. When the new higher level food category expenditure is updated, this can create a shift in non-taxed foods as the new expenditure is spread among food groups.

4.9.6 S6 Alternative Individualised Consumption

The population were assigned alternative, more individualised consumption based on two-step imputation and nearest neighbour matching process allowing greater variation in consumption patterns. The aim was to account for skewness in the consumption patterns and the influence of wider determinants of consumption patterns, such as ethnicity. A two-step procedure was adopted when allocating the NDNS consumption data to the models simulated population generated from HSE data. A consumption variable from NDNS, considering food groups or nutrients, that was highly correlated with population characteristics was selected. The chosen correlated consumption variable was sodium intake due to the variability in sodium intake by social groups of interest (ethnicity, BMI, household size, medical history) that were not included in the sociodemographic stratification. Daily intake of sodium in milligrams was imputed into the model population using multiple imputation by chained equations. The "mice" (Multivariate Imputation by Chained Equations) package in R was used to fill in

missing data using a highly robust method called Predictive Mean Matching (PMM). After combining datasets by related demographic and health data including: sex, age category, BMI category, ethnic group, NSSEC5, whether then are on Statin or antihypertensive medication and whether they have diabetes. Once allocated data on the intake of sodium, a nearest neighbour matching process allocated the consumption data for the individual in the NDNS dataset that had the nearest intake of sodium, that were within the same demographic group. The entire consumption data of that individual was allocated to ensure that the correlation between consumption food groups were held, and to ensure consistency in nutritional data. The imputed sodium data was replaced with the selected individual's (donor) sodium intake, along with full details of the donor consumption and nutritional intake.

Table 4.9.1: Proportion of population consuming each food group that may be affected by tax

Food Group	Proportion (%)	Food Group	Proportion (%)
Bacon and Ham	56%	Oily Fish	21%
Beef Veal and dishes	38%	Other bread	6%
Beverages dry weight	22%	Other meat and meat products	13%
Biscuits	63%	Other milk and Cream	24%
Breakfast cereals	65%	Other potatoes and Potato salads & Dishes	69%
Brown and wholemeal bread	54%	Other white fish Shellfish and Fish dishes	29%
Butter	39%	Out of home	60%
Cakes and Puddings	55%	Pasta rice and other cereals	76%
Cheese	64%	Pork and Dishes	16%
Chicken and Turkey dishes	63%	Reduced fat spread	60%
Chips fried & roast potatoes & potato products	48%	Salad and other raw vegetables	70%
Chocolate confectionery	45%	Sausages burger and kebabs	36%
Crisps and savoury snacks	47%	Savoury sauces pickles gravies & condiments	89%
Eggs and Egg dishes	52%	Soft drinks low calorie	36%
Fruit	76%	Soft drink not low calorie	42%
Fruit Juice	38%	Soup homemade and retail	12%
Ice cream	18%	Sugar confectionery	18%
Lamb and lamb dishes	11%	Sugars preserves and sweet spreads	70%
Liver & dishes	4%	Tea coffee and Water	87%
Low Fat Milk	76%	Vegetables not raw	91%
Margarine and Oils	57%	Vegetable ready meal	37%
Meat pies and pastries	20%	White bread	74%
Meat ready meals	25%	White fish coated or fried	18%
Milk drinks	6%	Whole milk	24%
Nuts and seeds	23%	Yoghurt fromage frais and dairy desserts	40%

4.9.7 S7 Adjust for underreporting

There is evidence of underreporting of consumption in the NDNS. The daily calories consumed in the NDNS data has been estimated to be 70% of the true consumption (42). To adjust for this underreporting, consumption

in grams across food groups was altered, weighted by the calories within each food, scaling consumption such that the resulting total calories was increased (by $\frac{1}{0.7}$) to account for the estimated 30% underreporting.

4.9.8 S8 Alternative Price Elasticities of Demand

An analysis using Kantar data estimated the own and cross price elasticities of demand across food groups. These offer an alternative set of elasticities for comparison (43). Table 4.9.5 presents the alternative PED. The food groups included in this elasticity model are divided into 15 food types: dairy, dessert, red meat, processed meat and fish, ready meals, white meats and fish, fats, vegetables, condiments, fruit, drinks, bread, pasta and grains, sweet snacks, savoury snacks and cereals. Some of the food types are divided by health and less healthy dependent on Nutrient Profile Score. Model food groups were reallocated to these elasticity food groups. It was assumed less healthy better represented taxed foods where relevant, while healthy better represented non-taxed foods. As a result, the structure of this sensitivity analysis better aligns for the Unhealthy food tax policy, in which tax inclusion is based on the Nutrient profile model. Out of home consumption was not considered in this publication, therefore it was assumed to change to out of home consumption due to prices. The mapping between the model food groups to the alternative PED food groups, and the LCF codes used to determine the proportion within each are presented in Table 4.9.2.

Table 4.9.2: Mapping food groups within the model to food groups presented in the alternative Demand system.

Model Food Group	Alternative elasticities food group	LCF code
Bacon and Ham	Processed meat & fish	1.1.2.3.1, 1.1.2.3.2, 1.1.2.11.2
Beef Veal and dishes	Red Meat	1.1.2.1.1, 1.1.2.1.2, 1.1.2.1.3, 1.1.2.1.4, 1.1.2.1.5, 1.1.2.1.6
Beverages dry weight	Dairy	1.1.4.3.5
	Desserts and Puddings	1.2.1.3.1, 1.2.1.3.2
Biscuits	Bread	1.1.1.4.2, 1.1.1.4.5
	Sweet snacks	1.1.1.4.3, 1.1.1.4.4
Breakfast cereals	Cereals	1.1.1.5.1, 1.1.1.5.2, 1.1.1.5.3, 1.1.1.5.4, 1.1.1.5.5, 1.1.9.3.2
Brown and wholemeal bread	Bread	1.1.1.2.1, 1.1.1.2.2
Butter	Fats	1.1.5.1.1
Cakes and Puddings	Bread	1.1.1.4.1
	Desserts and Puddings	1.1.4.6.3, 1.1.1.6.1, 1.1.1.6.5, 1.1.1.6.6, 1.1.1.6.4, 1.1.1.6.2, 1.1.1.6.3, 1.1.8.2.3
Cheese	Dairy	1.1.4.5.1, 1.1.4.5.2, 1.1.4.5.3, 1.1.4.5.4, 1.1.4.5.5, 1.1.4.5.6
Chicken and Turkey dishes	Ready Meal	1.1.2.11.1
	White Meat Fish	1.1.2.5.1, 1.1.2.5.2
Chips fried & roast potatoes & potato products	Vegetables	1.1.7.9.2
Chocolate confectionery	Sweet snacks	1.1.8.4.1, 1.1.8.4.2
Crisps and savoury snacks	Vegetables	1.1.8.6.1
	Savoury	1.1.8.6.2
Eggs and Egg dishes	Dairy	1.1.4.7.1,

Fruit	fruit	1.1.6.1.1, 1.1.6.1.2, 1.1.6.3.1, 1.1.6.3.2, 1.1.6.3.3, 1.1.6.4.1, 1.1.6.4.2, 1.1.6.2.1, 1.1.6.5.1, 1.1.6.5.2, 1.1.6.8.1, 1.1.6.8.2, 1.1.6.7.1, 1.1.6.6.1
Fruit Juice	Drinks	1.2.2.4.1
Ice cream	Desserts and Puddings	1.1.8.7.1, 1.1.8.7.2, 1.1.8.7.3
Lamb and lamb dishes	Red Meat	1.1.2.4.1, 1.1.2.4.2, 1.1.2.4.3, 1.1.2.4.4
Liver & dishes	Red Meat	1.1.2.6.1, 1.1.2.6.2, 1.1.2.6.3, 1.1.2.6.4
Low Fat Milk	Dairy	1.1.4.2.1, 1.1.4.2.2
Margarine and Oils	Fats	1.1.5.2.1, 1.1.5.2.2, 1.1.5.5.1, 1.1.5.3.1, 1.1.5.4.1, 1.1.5.5.2
Meat pies and pastries	Ready Meal	1.1.2.12.2, 1.1.2.12.1, 1.1.2.12.3
Meat ready meals	Ready Meal	1.1.2.13.1, 1.1.2.13.2
Milk drinks	Dairy	1.1.4.6.4
Nuts and seeds	fruit	1.1.6.7.2, 1.1.6.7.3
Oily Fish	White Meat Fish<br>Processed meat & fish	1.1.3.2.3, 1.1.3.2.4, 1.1.3.2.1, 1.1.3.2.2, 1.1.3.4.2<br>1.1.3.5.1, 1.1.3.5.2
Other bread	Bread	1.1.1.3.1, 1.1.1.3.2, 1.1.1.3.3, 1.1.1.3.4, 1.1.1.3.6, 1.1.1.3.5
Other meat and meat products	Red Meat<br>Ready Meal<br>Processed meat & fish<br>White Meat Fish	1.1.2.6.5<br>1.1.2.9.1, 1.1.2.9.2, 1.1.2.10.1<br>1.1.2.11.3, 1.1.2.10.2<br>1.1.2.5.3, 1.1.2.7.1
Other milk and Cream	Dairy	1.1.4.3.1, 1.1.4.3.2, 1.1.4.3.3, 1.1.4.3.4, 1.1.4.6.5, 1.1.4.6.1, 1.1.4.6.2
Other potatoes and Potato salads & Dishes	Vegetables	1.1.7.4.2, 1.1.7.4.4, 1.1.7.4.1, 1.1.7.9.1, 1.1.7.8.5, 1.1.7.9.3
Other white fish Shellfish and Fish dishes	White Meat Fish	1.1.3.1.1, 1.1.3.1.2, 1.1.3.4.1, 1.1.3.3.1, 1.1.3.3.2
Out of home	Out of Home	1.3.1.1.1, 1.3.1.1.2, 1.3.1.3.1, 1.3.1.3.2, 1.3.1.3.3, 1.3.1.4.1, 1.3.1.3.4, 1.3.1.1.3, 1.3.1.1.4, 1.3.1.4.2, 1.3.1.2.1, 1.3.1.5.1, 1.3.1.7.1, 1.3.1.2.4, 1.3.1.2.5, 1.3.1.2.2, 1.3.1.2.3, 1.3.1.6.1, 1.3.1.8.2, 1.3.1.8.6, 1.3.1.8.5, 1.3.1.8.7, 1.3.1.8.4, 1.3.1.8.3, 1.3.1.8.1
Pasta rice and other cereals	Pasta Grains<br>Cereals<br>Ready Meal	1.1.1.7.1, 1.1.1.7.2, 1.1.1.7.5, 1.1.1.8.1, 1.1.1.8.2, 1.1.1.8.3, 1.1.1.8.4,<br>1.1.9.3.3,<br>1.1.1.7.3,<br>1.1.1.7.4,
Pork and Dishes	Red Meat	1.1.2.2.1, 1.1.2.2.2, 1.1.2.2.3, 1.1.2.2.4
Reduced fat spread	Fats	1.1.5.2.3, 1.1.5.2.4
Salad and other raw vegetables	Vegetables<br>Condiments	1.1.7.2.1, 1.1.7.2.2, 1.1.7.2.3, 1.1.7.1.1, 1.1.7.1.4, 1.1.7.3.3, 1.1.7.3.4, 1.1.7.1.2, 1.1.7.5.1, 1.1.7.5.2, 1.1.7.5.4, 1.1.7.5.3, 1.1.7.3.1, 1.1.7.5.5, 1.1.7.3.5, 1.1.7.5.6, 1.1.7.1.3, 1.1.7.3.2<br>1.1.9.2.3

Sausages burger and kebabs	Processed meat & fish	1.1.2.8.1, 1.1.2.8.2, 1.1.2.8.4
	Ready Meal	1.1.2.8.3,
Savoury sauces pickles gravies & condiments	Condiments	1.1.9.1.3, 1.1.9.1.1, 1.1.9.1.2
	Other	1.1.9.6.2, 1.1.9.2.1
Soft drinks low calorie	Drinks	1.2.2.3.2, 1.2.2.2.2
Soft drink not low calorie	Drinks	1.2.2.3.1, 1.2.2.2.1
Soup homemade and retail	Ready Meal	1.1.9.5.1, 1.1.9.5.2
Sugar confectionery	Sweet snacks	1.1.8.5.1, 1.1.8.5.2, 1.1.8.5.3, 1.1.8.5.4
Sugars preserves and sweet spreads	Other	1.1.8.1.1, 1.1.8.2.1, 1.1.8.2.2, 1.1.8.3.1, 1.1.8.2.4, 1.1.8.3.2
Tea coffee and Water	Drinks	1.2.1.2.1, 1.2.1.1.1, 1.2.1.1.2, 1.2.1.1.3, 1.2.2.1.1
Vegetables not raw	Vegetables	1.1.7.8.1, 1.1.7.8.2, 1.1.7.8.3, 1.1.7.8.4, 1.1.7.8.6, 1.1.7.7.2, 1.1.7.7.1, 1.1.7.8.7, 1.2.2.5.1, 1.1.7.6.1, 1.1.7.6.2, 1.1.7.6.3
	Other	1.1.9.4.1
Vegetable ready meal	Ready Meal	1.1.7.10.1, 1.1.9.3.1
White bread	Bread	1.1.1.1.1, 1.1.1.1.2, 1.1.1.1.3, 1.1.1.1.4
White fish coated or fried	Processed meat & fish	1.1.3.6.1
Whole milk	Dairy	1.1.4.1.3, 1.1.4.1.2, 1.1.4.1.1
Yoghurt fromage frais and dairy desserts	Dairy	1.1.4.4.1, 1.1.4.4.2

Table 4.9.3: Price Elasticities of demand of higher-level food categories controlling for non-food spending

	Savoury	Meat	Fish and Seafood	Dairy	Butter/Spreads	Fruit	Vegetables	Sweet Foods	Others	Drinks	Out of Home	Snacks	Non-Food
**Savoury**	-0.463	0.179	-0.323	0.102	0.604	-0.147	0.052	0.008	0.082	0.020	0.178	0.164	-0.023
**Meat**	0.338	-0.115	-0.177	0.132	-0.083	0.280	0.044	0.530	0.378	0.253	-0.346	0.533	-0.062
**Fish and Seafood**	-0.132	-0.039	-0.376	-0.115	-0.045	0.089	0.157	-0.272	0.247	0.278	0.369	0.146	-0.012
**Dairy**	0.086	0.058	-0.239	-0.597	0.496	-0.251	0.194	-0.032	0.153	-0.317	-0.074	0.149	-0.008
**Butter/Spreads**	0.119	-0.009	-0.022	0.115	-0.421	-0.014	-0.098	0.081	-0.091	-0.005	-0.098	0.111	-0.005
**Fruit**	-0.106	0.113	0.159	-0.215	-0.051	-0.496	0.163	-0.027	-0.060	0.139	-0.161	-0.734	0.002
**Vegetables**	0.058	0.026	0.401	0.246	-0.526	0.233	-0.592	-0.243	0.044	0.515	-0.219	-0.548	-0.012
**Sweet Foods**	0.006	0.196	-0.457	-0.025	0.285	-0.027	-0.159	-0.438	-0.324	0.068	0.100	0.525	-0.017
**Others**	0.024	0.058	0.170	0.053	-0.130	-0.023	0.012	-0.132	-0.662	0.232	-0.043	0.097	-0.009
**Drinks**	0.016	0.100	0.490	-0.272	-0.019	0.136	0.355	0.073	0.596	-0.723	-0.119	-0.117	-0.018
**Out of Home**	0.116	-0.115	0.566	-0.051	-0.312	-0.136	-0.129	0.095	-0.094	-0.101	-0.582	-0.032	0.000
**Snacks**	0.139	0.239	0.293	0.148	0.472	-0.829	-0.430	0.632	0.282	-0.134	-0.046	-0.245	-0.025
**Non-Food**	-0.680	-1.271	-1.173	0.039	-0.729	0.491	-0.193	-0.814	-1.253	-0.836	0.229	-1.133	-0.885

Table 4.9.4: Price Elasticities of demand of higher-level food categories for the whole population

	Savoury	Meat	Fish and Seafood	Dairy	Butter/Spreads	Fruit	Vegetables	Sweet Foods	Others	Drinks	Out of Home	Snacks
**Savoury**	-0.741	-0.058	-0.488	-0.021	0.296	-0.099	0.001	-0.121	-0.060	0.042	0.113	0.075
**Meat**	-0.081	-0.472	-0.408	0.322	-0.337	-0.119	-0.233	0.042	-0.013	-0.077	-0.827	0.009
**Fish and Seafood**	-0.181	-0.087	-0.874	-0.042	-0.201	0.143	0.134	-0.190	0.055	0.137	0.190	0.001
**Dairy**	-0.022	0.144	-0.117	-0.967	0.372	-0.383	0.107	-0.007	0.332	-0.300	-0.244	0.216
**Butter/Spreads**	0.055	-0.038	-0.100	0.082	-0.617	-0.020	-0.087	0.033	-0.134	0.060	-0.117	0.078
**Fruit**	-0.055	-0.042	0.267	-0.307	-0.063	-0.290	0.136	0.024	-0.068	0.085	0.168	-0.652
**Vegetables**	0.018	-0.135	0.348	0.151	-0.472	0.187	-0.689	-0.266	-0.058	0.362	-0.021	-0.581
**Sweet Foods**	-0.076	0.013	-0.336	0.002	0.121	0.011	-0.176	-0.772	-0.716	0.049	0.120	0.329
**Others**	-0.010	0.001	0.039	0.114	-0.192	-0.026	-0.013	-0.291	-0.769	0.126	-0.039	0.035
**Drinks**	0.039	-0.031	0.244	-0.240	0.240	0.076	0.248	0.057	0.322	-1.012	-0.156	-0.305
**Out of Home**	0.092	-0.299	0.320	-0.165	-0.391	0.154	-0.005	0.132	-0.089	-0.131	-0.273	-0.040
**Snacks**	0.069	0.002	-0.009	0.217	0.349	-0.378	-0.460	0.406	0.094	-0.348	-0.066	-0.415

Table 4.9.5: Price Elasticities of demand of Alternative Demand model

	1 - Bread & morning goods (healthier)	2 - Bread & morning goods (less healthy)	3 - cereals & cereal bars (healthier)	4 - cereals & cereal bars (less healthy)	5 - Sauces & condiments (healthier)	6 - Sauces & condiments (less healthy)	7 - Dairy (healthier)	8 - Dairy (less healthy)	9 - Desserts & puddings	10 - Non-alcoholic drinks (excl. dairy) (healthier)	11 - Non-alcoholic drinks (excl. dairy) (less healthy)	12 - Fat & oil	13 - Fruits	14 - Other	15 - Out of Home	16 - Pasta, rice, grains, dry pulses	17 - Processed meat & fish (healthier)	18 - Processed meat & fish (less healthy)	19 - Ready meals & convenience foods (healthier)	20 - Ready meals & convenience foods (less healthy)	21 - Fresh & frozen red meat (healthier)	22 - Fresh & frozen red meat (less healthy)	23 - Snacks - savoury	24 - Snacks - sweet	25 - Vegetables	26 - Fresh & frozen white meat & fish, eggs
1 - Bread & morning goods (healthier)	-0.78	-0.02	0.00	0.00	0.03	0.01	-0.02	-0.03	-0.01	0.01	0.02	0.04	0.03	0.01	0.00	0.02	0.00	-0.04	-0.01	-0.02	-0.01	-0.01	0.01	-0.08	0.15	-0.02
2 - Bread & morning goods (less healthy)	-0.02	-0.82	0.03	-0.01	-0.01	0.03	-0.19	-0.02	-0.03	0.01	-0.04	-0.04	0.03	-0.02	0.00	0.01	-0.02	-0.05	-0.02	-0.05	0.02	0.02	0.02	-0.07	-0.04	0.00
3 - cereals & cereal bars (healthier)	0.03	0.00	-0.85	0.04	-0.01	0.00	0.29	0.04	-0.03	0.09	0.03	0.03	-0.18	0.01	0.00	0.03	-0.03	-0.08	-0.03	-0.07	-0.01	-0.03	0.02	-0.05	-0.07	-0.02
4 - cereals & cereal bars (less healthy)	-0.01	-0.01	0.04	-0.90	0.01	-0.02	0.24	0.03	-0.09	0.03	0.03	-0.07	-0.16	0.02	0.00	0.02	-0.08	-0.09	0.02	-0.09	-0.03	-0.02	0.10	-0.08	-0.02	-0.02
5 - Sauces & condiments (healthier)	0.02	0.06	0.01	0.01	-0.93	0.01	-0.29	0.06	0.09	-0.07	-0.02	-0.06	0.14	-0.01	0.00	-0.02	0.10	0.02	-0.02	0.15	-0.02	-0.01	-0.12	0.05	0.05	-0.01
6 - Sauces & condiments (less healthy)	0.02	0.03	-0.01	-0.01	-0.03	-0.90	-0.09	0.04	0.05	-0.02	-0.02	-0.07	0.07	0.00	0.00	-0.07	0.04	0.05	0.03	0.09	-0.06	0.00	-0.10	0.04	0.05	0.01
7 - Dairy (healthier)	0.02	-0.04	0.00	0.04	0.01	0.00	-0.72	-0.04	-0.05	0.03	0.03	0.01	-0.10	0.01	0.00	0.03	-0.05	-0.03	0.01	-0.01	0.00	0.02	0.08	-0.07	0.07	-0.02
8 - Dairy (less healthy)	0.04	0.05	-0.01	0.01	-0.01	-0.03	-0.05	-0.78	-0.03	-0.03	-0.02	-0.03	0.08	0.01	0.00	-0.05	0.07	0.08	-0.03	0.13	0.01	-0.02	-0.01	0.04	-0.07	-0.01
9 - Desserts & puddings	-0.02	0.02	0.00	-0.01	-0.02	-0.01	-0.15	0.02	-0.81	-0.07	-0.04	-0.02	0.01	-0.01	0.00	-0.05	0.02	0.02	0.06	0.08	0.01	0.02	-0.09	0.03	-0.06	-0.01
10 - Non-alcoholic drinks (excl. dairy) (healthier)	0.01	-0.01	-0.01	0.01	-0.01	-0.05	0.12	-0.02	0.00	-0.87	-0.03	0.01	-0.03	0.00	0.00	0.00	-0.01	-0.03	-0.02	-0.03	-0.01	0.00	-0.01	0.02	0.16	-0.02
11 - Non-alcoholic drinks (excl. dairy) (less healthy)	-0.03	-0.01	0.02	-0.01	0.00	-0.03	-0.06	-0.02	-0.04	-0.09	-0.91	0.04	-0.14	0.03	0.00	-0.01	0.00	-0.10	0.10	0.07	0.02	0.04	-0.01	0.03	0.20	-0.01
12 - Fat & oil	0.03	-0.02	0.01	0.01	0.01	0.04	0.07	0.01	0.02	0.07	0.01	-0.88	0.00	-0.01	0.00	-0.05	0.00	0.02	-0.01	-0.02	0.00	-0.02	0.04	0.00	-0.12	-0.02
13 - Fruits	-0.02	-0.01	0.02	0.02	0.00	-0.02	0.14	-0.03	-0.07	-0.02	-0.01	-0.01	-0.76	0.00	0.00	0.06	-0.05	-0.03	0.03	0.02	0.01	-0.02	0.09	-0.07	0.14	-0.01
14 - Other	0.02	0.01	-0.05	0.02	0.03	-0.02	0.18	-0.03	-0.03	0.05	0.00	0.04	-0.09	-0.78	0.00	-0.05	-0.05	-0.03	0.02	-0.04	-0.01	-0.01	0.08	-0.08	-0.03	-0.03
15 - Out of Home	0.00	0.00	0.00	0.00	0.00	0.00	0.00	0.00	0.00	0.00	0.00	0.00	0.00	0.00	0.00	0.00	0.00	0.00	0.00	0.00	0.00	0.00	0.00	0.00	0.00	0.00
16 - Pasta, rice, grains, dry pulses	0.03	-0.05	-0.01	0.03	0.02	0.03	0.32	-0.07	0.00	0.07	0.03	0.01	-0.18	0.01	0.00	-0.78	-0.01	-0.10	-0.06	-0.12	-0.07	0.01	-0.01	-0.07	-0.05	-0.01

17 - Processed meat & fish (healthier)	-0.01	-0.03	-0.01	0.01	0.02	-0.05	0.22	-0.09	-0.06	-0.01	-0.02	-0.05	-0.10	-0.01	0.00	0.04	-0.90	-0.16	-0.04	-0.15	0.05	-0.03	0.06	-0.05	0.08	0.00
18 - Processed meat & fish (less healthy)	-0.07	0.01	0.00	-0.02	-0.01	-0.03	-0.09	-0.04	0.03	-0.03	-0.02	-0.01	0.09	0.00	0.00	0.00	0.00	-0.80	-0.08	-0.03	-0.05	0.02	-0.07	0.06	0.08	-0.01
19 - Ready meals & convenience foods (healthier)	-0.04	-0.05	-0.01	0.05	0.01	-0.01	0.18	-0.08	-0.05	0.06	0.03	-0.01	-0.10	0.00	0.00	0.04	-0.05	-0.09	-0.83	-0.17	-0.05	-0.05	0.11	-0.10	-0.02	-0.01
20 - Ready meals & convenience foods (less healthy)	-0.04	-0.01	-0.01	-0.02	0.01	0.00	0.04	-0.05	-0.03	0.01	0.01	-0.01	-0.04	0.01	0.00	0.03	0.00	-0.03	-0.02	-0.92	0.01	-0.01	0.05	-0.02	0.07	0.00
21 - Fresh & frozen red meat (healthier)	-0.10	-0.03	-0.01	0.02	0.05	0.06	0.05	-0.01	0.03	0.09	0.03	0.05	-0.07	-0.02	0.00	0.03	-0.11	-0.05	-0.11	-0.25	-0.86	-0.02	0.03	-0.09	-0.22	-0.02
22 - Fresh & frozen red meat (less healthy)	-0.14	-0.01	0.02	0.08	0.05	0.01	0.30	-0.12	-0.03	0.10	0.11	-0.05	-0.16	-0.02	0.00	0.06	-0.15	-0.20	-0.13	-0.14	0.01	-0.86	0.14	-0.14	0.13	0.00
23 - Snacks - savoury	0.05	0.02	-0.02	0.00	-0.02	-0.01	-0.16	0.06	-0.02	-0.03	-0.01	0.02	0.02	0.00	0.00	-0.03	0.08	0.04	0.00	0.08	0.01	-0.02	-0.97	0.04	-0.16	0.01
24 - Snacks - sweet	-0.09	0.04	-0.06	-0.09	-0.04	0.02	-0.31	0.25	-0.02	-0.05	-0.03	0.02	0.01	-0.04	0.00	-0.10	0.02	0.13	0.00	-0.01	-0.03	0.02	-0.24	-0.86	-0.46	0.03
25 - Vegetables	0.00	-0.01	0.00	0.03	0.02	0.00	0.17	-0.09	0.00	0.02	0.01	0.00	0.01	0.01	0.00	0.03	-0.01	-0.03	-0.07	0.00	-0.03	-0.01	0.11	-0.04	-0.81	-0.02
26 - Fresh & frozen white meat & fish, eggs	0.04	-0.01	-0.01	0.00	0.02	0.00	-0.02	-0.07	0.01	0.00	0.02	-0.03	0.02	-0.01	0.00	0.01	0.01	0.07	-0.07	0.01	0.00	-0.03	0.03	0.01	0.01	-0.89

5 Health Model – SMPH Obesity and Diabetes Prevention Model

5.1 Overview

5.1.1 Model overview

This individual patient simulation models annual health trajectories until death. It tracks metabolic risk factors and GP screenings for diabetes, hypertension, and dyslipidaemia. The model estimates the risk of chronic conditions, including cardiovascular events, cancer, dementia, and mortality. Finally, it calculates healthcare and social care costs, quality-adjusted life years (QALYs), and economic activity.

5.1.2 Key assumptions

- **Diet changes last a lifetime:** Any changes to a person's diet and health risks are assumed to last for the rest of their life.
- **Indirect health benefits:** Better nutrition does not directly prevent major health events. Instead, it improves heart and metabolic health markers (like BMI, blood pressure or cholesterol), which then lowers long-term disease risks.
- **Diabetes management:** At diagnosis of type 2 diabetes all patients initiation metformin, and follow a smooth HbA1c trajectory with new lines of treatment added at pre-determined thresholds.
- **No future population trends:** Future heart and metabolic health risks are predicted using only a person's starting health levels, characteristics and history, ignoring any predicted future changes in the wider population.
- **Tooth decay progression:** At age 16, teenagers with any tooth decay are assigned an average number of cavities, which can then increase as they get older.
- **No new people enter the study**: The model uses a "closed cohort," meaning it starts with a fixed group of individuals and tracks them over time (usually until they die) without adding any new people to the simulation along the way.
- **Diabetes and Cardiovascular risks**: Individuals are only at risk of cardiovascular disease and type 2 diabetes from the age of 25.
- **Cancer, osteoarthritis and dementia risks**: Individuals are only at risk of osteoarthritis from the age of 45, cancers from the age of 50 and dementia from the age of 60.

5.1.3 Developing the Conceptual Model

The conceptual model was originally developed according to a conceptual modelling framework for complex public health models (47). In line with this framework the conceptual model was developed in collaboration with a project stakeholder group comprising health economists, public health specialists, research collaborators from other SPHR groups, diabetologists, local commissioners and lay members. The initial broad scope for the conceptual model was based on the structure of previous diabetes prevention models used for National Institute for Health and Care Excellence public health guidance (48, 49) and discussions with experts in diabetes prevention modelling. The model was further extended to include Dementia as a health outcome for individuals aged over 60 years in the model (50).

A second phase of conceptual modelling was conducting to support the adaptation of the model to evaluation food tax interventions (51).

5.2 Model Structure

We developed an individual patient simulation that estimates individuals' health in yearly cycles until death. The simulation draws baseline demographic and clinical status for individuals sampled from the Health Survey for England (HSE) 2018 and 2019 (9, 10). The simulation estimates yearly changes in metabolic risk factors based upon the individuals' baseline characteristics. Within each annual cycle the individuals may be screened for hypertension, dyslipidaemia or diabetes during a visit to the General Practitioner (GP). Opportunistic screening is used to determine diabetes diagnosis or the initiation of anti-hypertensive treatment or statins. Baseline characteristics and metabolic risk factors determine the individuals' probability of cardiovascular events, diabetes microvascular complications, cancer, osteoarthritis and depression. Individuals within the model may die in any cycle as a result of cardiovascular disease, cancer, or from other causes.

Figure S5.2.1 illustrates the risk factors, health events and outcomes that are estimated and updated within a cycle of the model. The individual's health profile is updated in each annual cycle of the model.

Within the risk factor module each cycle of the model updates the age of the individual and how many times the individual attends the GP. The model extracts a new BMI for the individual from their simulated natural history. Individuals aged 24 and over update their HbA1c, systolic blood pressure and cholesterol each cycle, conditional on their current health status in relation to type 2 diabetes, hypertension and cardiovascular risk respectively. If the individual has been diagnosed with dementia their mini-mental state examination (MMSE) score is updated.

Within the events module individuals aged more than 24 may undergo assessment for diabetes, hypertension and dyslipidaemia during a GP consultation. These individuals may experience diabetes, cardiovascular outcomes and heart failure. If the individual has a history of cardiovascular disease (CVD history=1), they follow a different pathway to those without a history of cardiovascular disease (CVD history=0). Individuals with HbA1c greater than 48 mmol/mol (6.5%) are assumed to be at risk of diabetes related complications. Individuals aged more than 50 are at risk of cancer diagnosis and those with a diagnosis of cancer are at risk of mortality due to cancer. Individuals aged more than 50 may develop osteoarthritis. From the age of 59 individuals may be diagnosed with dementia. Finally, all individuals are at risk of dying due non cardiovascular or cancer mortality.

The model records the healthcare related costs associated with GP attendance, and the health events listed above. Social care costs are simulated and associated with BMI and other health events. Health related quality of life is simulated in adulthood, children are assumed to have perfect health, and combined with life years to calculate Quality Adjusted Life Years. Economic activity and social benefit income are associated with health related quality of life.

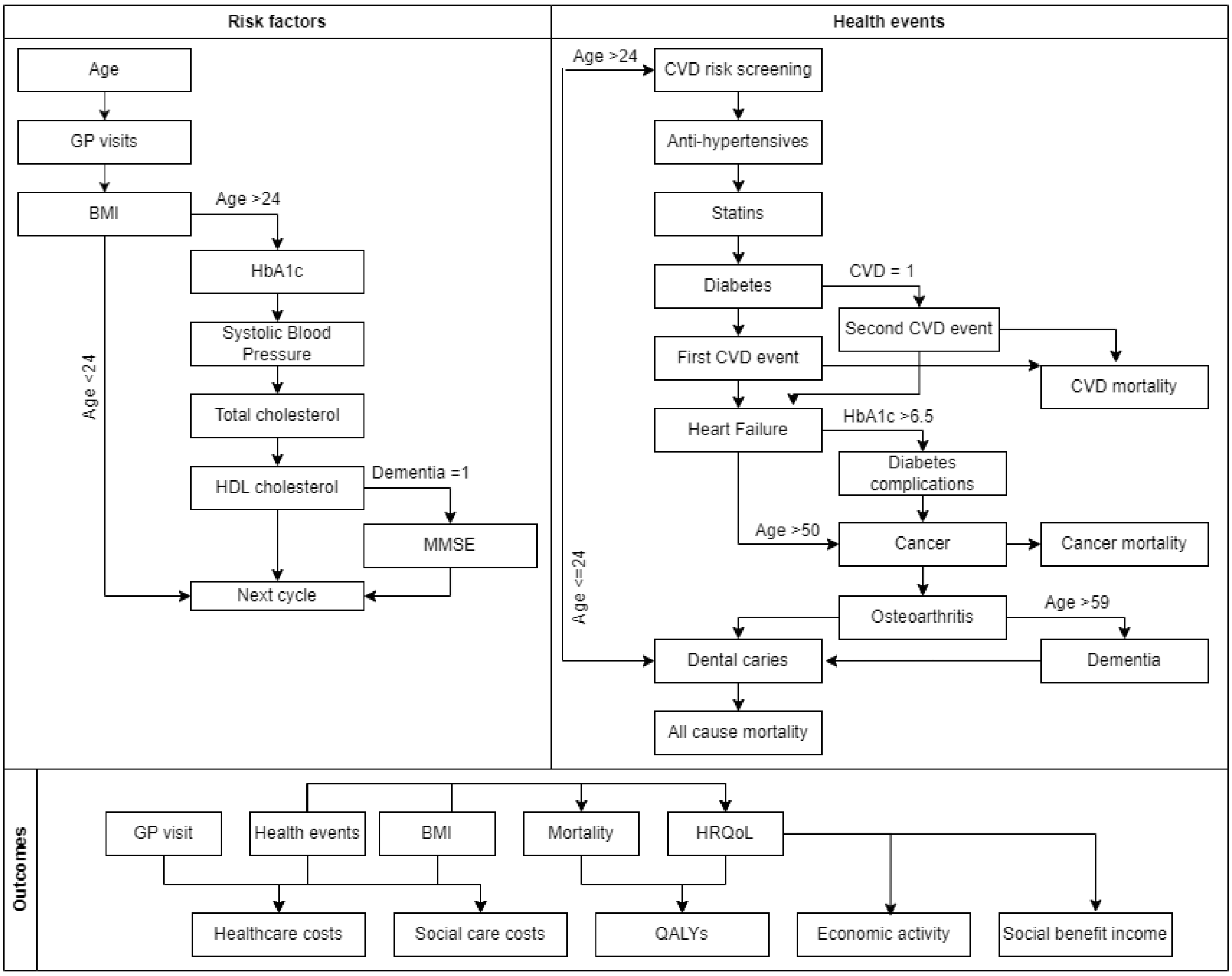

Figure S5.2.1: Model Schematic

5.3 Base Year for Data input and Analysis

Table 5.3.1: Source and year of key data inputs

	**Data Source Year**	**Data reference**
Population counts	UK Counts 2022<br>Proportions of nssec by age and sex England Wales age-sex 2022<br>Scotland nssec-age-sec 2022<br>NI nssec by age and sex 2021<br>England Wales age-nssec 2021	(14-17) MYE2: Females by single year of age and sex for local authorities in the UK, mid-2022
Costs Healthcare	BNF 2025<br>PSSRU 2023<br>Other Inflated to 2022	See Costs section
Food Prices (at home)	2022	
Out of Home food prices	2023	
Consumption	2009-2019	

5.4 GP Attendance in the General Population

GP visit frequency was simulated in the dataset to estimate healthcare utilisation for the general population and the costs associated with these GP appointments. A statistical model to estimate number of GP attendance per year, conditional on characteristics, such as age, ethnicity and comorbidities, was developed. If those with life limiting health concerns visit the GP more, then a change in the presence of these conditions will change the number attending GP appointments and thus creating an overall change in primary-care costs.

A negative binomial model specification was used to generate count and a skewed distribution was observed in the dataset.

$$\mu_i = \exp(x_i \beta)$$

Where μ_i is the expected mean number of GP visits for individual i, denoted Y_{i},. x_i represents a vector of individual characteristics for individual i, and β is a vector of regression coefficients. The dispersion parameter of the Negative Binomial distribution v_i was sampled from a gamma distribution with mean 1 and variance α reported in Table 5.4.2. The conditional probability of observing y_i GP visits given individual characteristics x_i is expressed as:

$$p(Y_i = y_i |, x_i, v_i) = \frac{(v_i \mu_i)^{y_i} e^{-(v_i \mu_i)}}{y_i!}$$

where the simulated counts Y_i were generated from a Poisson distribution with individual-specific mean $v_i \mu_i$.

We used data from the Health Survey for England 2019 (10), which collected a patient reported variable on the number times they attended a GP in the last year. It also collected demographic and health related information, including whether they had diabetes or not. This would allow the GP attendance model to represent attendance associated with diabetes. While explicit variables indicating comorbidities were not included, variables indicating which related medications they are taken is reported. Whether the patient has a prescription for antidepressants, lipid lowering medication or anti-hypertensive medication is thought to have a relation with GP attendance, especially considering the policies relating to regular medication reviews. A variable indicating whether an individual has a prescription for these medications is included in the model. Finally, to account for any comorbidities, a variable indicating whether they have a life limiting illness was included. The population included were adults above 20 years old. The characteristics of the study population are reported in Table 5.4.1.

Table 5.4.1: Characteristics of HSE 2019 (10)

Variable	Observations	Mean	SD
GP Visits	7,746	2.96	2.99
Age (20+)	7,855	52.52	17.69
Male	7,855	0.46	0.50
Black	7,826	0.03	0.18
Asian	7,826	0.10	0.30
Ethnic Minority	7,826	0.17	0.37
BMI	6,470	26.24	6.60
Diabetes	7,848	0.08	0.27
Life Limiting Illness	7,847	0.24	0.43
Lipid Lowering Medication	4,774	0.15	0.36
Anti-Hypertensive Medication	4,768	0.24	0.43
Antidepressant	4,774	0.10	0.30

The coefficients of the Negative Binomial model, described in Table 5.4.2, were used to calculate the first parameter of the Negative Binomial distribution μ_i based on an individual's characteristics (x_i). The dispersion parameter (v_i) sampled from the gamma distribution using α reported in Table 5.4.2. Three regression specifications are reported in Table 5.4.2. Model 1 regresses GP visits against age, sex, ethnicity, BMI, whether they have diabetes and whether they have a life limiting illness. Model 2 regresses GP visits against all parameters in model 1, but with the additional variable indicating if they are prescribed antidepressant

medications. Finally, Model 3 included whether they are prescribed CVD medication (lipid lowering and anti-hypertensive medication) on top of Model 2.

Table 5.4.2: GP attendance reported in the HSE 2019 (10)

	Model 1		Model 2		Model 3	
	Mean	SE	Mean	SE	Mean	SE
Age	0.00263***	(0.000725)	0.00195**	(0.000880)	-0.00221**	(0.000989)
Male	-0.211***	(0.0246)	-0.177***	(0.0295)	-0.220***	(0.0297)
Black	0.135*	(0.0726)	0.296***	(0.0906)	0.303***	(0.0902)
Asian	0.211***	(0.0438)	0.247***	(0.0562)	0.242***	(0.0557)
BMI	0.0105***	(0.00207)	0.00866***	(0.00246)	0.00548**	(0.00247)
Diabetes	0.282***	(0.0438)	0.238***	(0.0517)	0.112**	(0.0533)
Life Limiting Illness	0.700***	(0.0263)	0.587***	(0.0320)	0.553***	(0.0319)
Antidepressants			0.406***	(0.0417)	0.407***	(0.0413)
Lipid lowering Medication					0.207***	(0.0424)
Anti-Hypertensive Medication					0.224***	(0.0392)
Constant	0.422***	(0.0704)	0.480***	(0.0844)	0.727***	(0.0883)
Alpha α	0.547	(0.0180)	0.494	(0.0206)	0.476	(0.0202)
LnAlpha $\ln(\alpha)$	-0.603***	(0.0330)	-0.705***	(0.0417)	-0.741***	(0.0424)
N	6,378		4,215		4,209	
Pseudo R^2	0.0357		0.0392		0.0434	
Standard errors in parentheses (*** p<0.01, ** p<0.05, * p<0.1)						

The average number of GP visits was approximately 3 times in the year. The number of GP visits was statistically significantly related with all variables included. Individuals that are Black or Asian are more likely to attend the GP, while males are less likely. Those with a higher BMI, a life limiting illnesses or are taking CVD medication and antidepressants go to the GP more often. The large positive relationships between GP attendance and indicators of ill health justify their inclusion. An unexpected negative relationship is seen in age when including CVD medication variables. This could be explained by the strong positive correlation between age and taking CVD related medication.

Model 3 was chosen to model GP utilisation due to the additional explanatory power for medication use, high statistical significance of all variables and the greater Pseudo R^2 value. Parameter samples were taken from a multivariate normal distribution defined by the variable covariance matrix from Model 3, reported in Table 5.4.3.

Table 5.4.3: Variance-covariance matrix for GP Attendance, Model 3

	Age	Male	Black	Asian	BMI	Diabetes	Life Limiting Illness	Antidepressants	Lipid Lowering Med	Anti-Hypertensive Med	Constant	LnAlpha
Age	0.0000											
Male	-0.0000	0.0009										
Black	0.0000	0.0001	0.0081									
Asian	0.0000	0.0000	0.0003	0.0031								
BMI	0.0000	-0.0000	-0.0000	0.0000	0.0000							
Diabetes	-0.0000	-0.0000	-0.0002	-0.0001	-0.0000	0.0028						
Life Limiting Illness	-0.0000	0.0000	-0.0000	0.0000	-0.0000	-0.0001	0.0010					
Antidepressants	0.0000	0.0001	0.0002	0.0002	-0.0000	-0.0001	-0.0003	0.0017				
Lipid lowering Medication	-0.0000	-0.0002	0.0001	-0.0000	-0.0000	-0.0005	-0.0000	-0.0001	0.0018			
Anti-Hypertensive Medication	-0.0000	-0.0000	-0.0000	-0.0000	-0.0000	-0.0002	-0.0001	0.0000	-0.0005	0.0015		
Constant	-0.0001	-0.0003	-0.0005	-0.0009	-0.0002	0.0004	0.0000	-0.0001	0.0006	0.0008	0.0078	
**LnAlpha**	0.0000	-0.0000	-0.0000	0.0000	0.0000	0.0000	0.0000	0.0000	0.0000	0.0000	-0.0000	0.0018

5.5 Longitudinal Trajectories of weight, height and BMI

The method uses cross-sectional data from the Health Survey for England 2018 and 2019 to fit distributions that describe variability in weight and BMI through childhood to adulthood (9, 10). From these distributions it is possible to estimate the expected changes in weight and BMI as individuals age. In order to maintain heterogeneity in weight throughout an individual's lifetime, it is important that we assume that the changes in weight are different across individuals in the model, because we know that some individuals will gain more weight than others over time. With this method, we assume that individuals weight changes over time, but their position on the weight distribution for those of similar age is constant throughout their life. For example, if an individual has a weight on the 5th percentile aged 20, then their estimated change in weight from age 20 to 25 will be estimated as the difference in weight in the 5th percentile for those aged 25 and the 5th percentile for those aged 20.

The advantages of this method are that it uses the most recent health survey for England data on the distributions of BMI across England. We are aware that following the Covid-19 pandemic the health profile and lifestyles of individuals in the UK have change dramatically, so methods that use retrospective data have limitations. Secondly, this method provides a single method to describe weight changes that can be implemented at any age in the model, rather than methods that aim to combine multiple methods across age categories, which can lead to inconsistencies in model structures, differences in variable measurement and adds considerable computation burdens. Finally, this method provides a method that can be relatively easily updated with new data from the Health Survey for England, so provides a method that can be efficiently updated with new information over time.

There are limitations in this method. The Health Survey for England will capture differences in age categories, but because this data is cross-sectional, rather than longitudinal, will not be able to forecast future changes in BMI based on the distributions found in current ages. For example, we know that in recent years more children are exceeding the thresholds for overweight and obesity. Therefore, it is unlikely that the distribution of weight for those currently aged 50, will be a good representation of the future BMI at age 50 for the children entering the model.

5.6 Longitudinal Trajectories of Metabolic Risk Factors

Two sets of statistical analyses were used to describe metabolic trajectories (excluding BMI) for individuals in the model. The trajectories for systolic blood pressure, total cholesterol, and HDL cholesterol in the model from age 25 onwards were simulated statistical models from the Whitehall II cohort for adults aged less than 60 and English Longitudinal Study of Ageing for those aged over 60. HbA1c trajectories are simulated from the English Longitudinal Study of Ageing, due to HbA1c only being collected in two timepoints in the Whitehall II analysis. A summary for each set of the metabolic trajectory models are provided below.

5.6.1 Whitehall II Data Analysis

Changes in total cholesterol, HDL cholesterol and SBP were estimated from statistical analysis of the Whitehall II cohort (11). The growth factors for all 5 risk factors were estimated using parallel latent growth modelling. Following the estimation of the growth trajectory for BMI, the 10 year growth rate in BMI was used to estimate growth rates for the other metabolic trajectories. This enabled the growth factors for BMI to be implemented as covariates for the growth processes of glycaemia, systolic blood pressure, and total cholesterol[1]. The structural assumptions of the analysis are described in more detail below.

[1]The model did not converge when BMI slope was included as a predictor for HDL growth.

In the Whitehall II data analysis it was assumed that individuals have an underlying level of glycaemia, which cannot be observed but can be measured by HbA1c, FPG, and 2-hour glucose. This underlying propensity for diabetes is referred to as latent glycaemia. The statistical model estimated the unobservable latent glycaemia, and from this identified associations with test results for HbA1c, FPG, and 2-hour glucose. The longitudinal changes in BMI, glycaemia, SBP, total cholesterol and HDL cholesterol could then be estimated through statistical analysis.

These growth factors are conditional on several individual characteristics including age, sex, ethnicity, smoking, family history of CVD, and family history of type 2 diabetes. We related the effect of changes in BMI to changes in glycaemia, SBP and total cholesterol. However, if an intervention is known to be effective in reducing BMI and the other metabolic risk factors, the Whitehall II model is adjusted to temporarily remove the indirect effect of the intervention through BMI. This ensures that the effectiveness of the intervention is not over-estimated. Unobservable heterogeneity between individual growth factors not explained by patient characteristics was incorporated into the growth models as random error terms. Correlation between the random error terms for glycaemia, total cholesterol, HDL cholesterol and systolic blood pressure was estimated from the Whitehall II cohort. This means that in the simulation, an individual with a higher growth rate for glycaemia was more likely to have a higher growth rate of total cholesterol and SBP.

An advantage of this parallel growth analysis is that it was able to estimate the effect of growth in BMI on the other metabolic risk factors. The statistical analysis also described the correlation between changes in glycaemia, SBP, total cholesterol and HDL cholesterol. As a consequence, the growth factor random error terms were not assumed to be independent and were sampled from a multivariate normal distribution $\boldsymbol{\upsilon} \sim N(0, \Omega)$. Estimates for the covariance matrix are derived from the covariance estimates reported in the statistical analysis.

The baseline observations for HbA1c, SBP, cholesterol and HDL cholesterol were extracted from the Health Survey for England in order to simulate a representative sample of the UK population. The predicted intercept for these metabolic risk factors was estimated using the Whitehall II analysis to give population estimates of the individuals' starting values, conditional on their characteristics. The difference between the simulated and observed baseline risk factors was taken to estimate the individuals' random deviation from the population expectation. The individual random error in the slope trajectory was sampled from a conditional multivariate normal distribution to allow correlation between the intercept and slope random errors.

After the individual slops factors have been estimated the estimates for systolic blood pressure, total cholesterol, and HDL cholesterol are used in the model. The slope variables for glycaemia are not used.

Figure S5.6.1: Path analysis of final statistical analysis of the Whitehall II cohort (11)

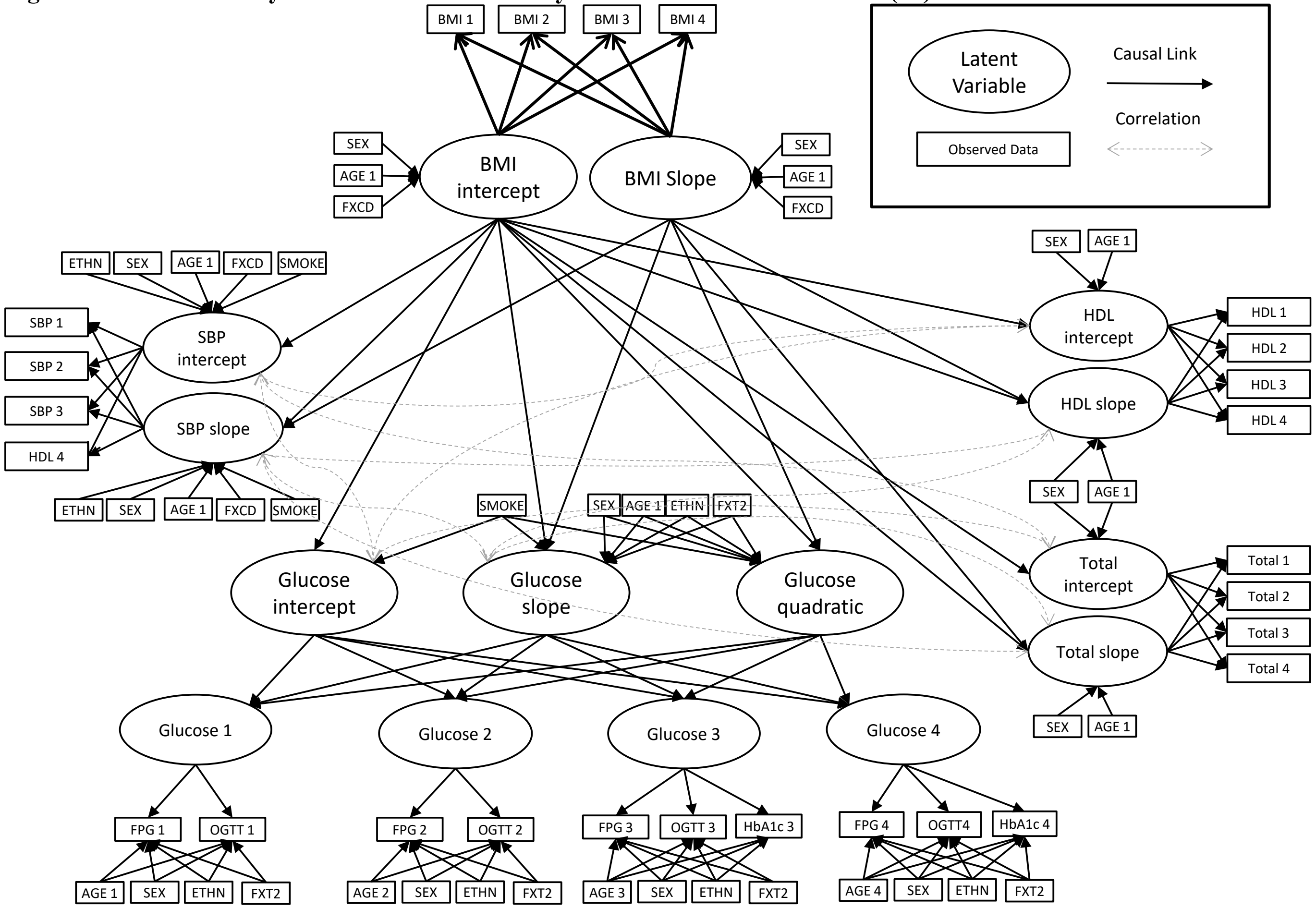

5.6.2 ELSA Data Analysis

Changes in HbA1c, systolic blood pressure, total cholesterol and HDL cholesterol were estimated from a statistical analysis of the ELSA cohort. The changes with age were estimated using independently estimate random coefficient growth models in Stata 13. The growth trajectory models for the metabolic risk factor were estimated under the statistical framework of growth curve modelling (GCM) (52). GCM is an approach to using longitudinal data to estimate shape and rate of change over time. GCM was chosen because it can allow modelling of variability in participants fixed and slope parameters. The growth factors for the metabolic risk factors were assumed to vary between individuals to allow unobservable random effects to describe the heterogeneity in intercept and slope parameters. Assessment of the data indicated that there was significant variance in the intercept (risk factor starting value) and slope (change in risk factor over time) for all metabolic risks. The growth factor models without covariates were specified as.

$$Y_{ij} = (\beta_1 + \zeta_{1j}) + (\beta_2 + \zeta_{2j})t_{ij} + \epsilon_{ij}$$

Where Y describes the observed metabolic risk factor for individual j at occasion i, at age, t. β_1 is the population mean intercept, and β_2 population mean slope. The random factors ζ_{1j} and ζ_{2j} describe the random variability across individuals in the intercept and slopes respectively. The statistical models were weighted for selection bias using nurse visit weights for BMI and systolic blood pressure and blood sample weights for Total Cholesterol, HDL Cholesterol and HbA1c supplied by the ELSA dataset to improve the representativeness of the analysis for an English population.

HbA1c, systolic blood pressure, total cholesterol and HDL cholesterol were assumed to be linear with time. All model intercepts and slopes were adjusted for sex, smoking status, deprivation and ethnicity. Anti-hypertensive treatment was included as an additional covariate in the systolic blood pressure model. Unfortunately, data on statin were not available for any wave except wave 6, therefore we did not include this as a covariate in the analysis. Covariates were included in the final model if the variable was statistically significant with a p-value less than 0.1. A description of each model is described below and presented in Table 5.6.1. Parameters within each model are sampled from a multivariate normal distribution defined by the respective variance-covariance matrix.

<u>BMI model</u>

The model will a random coefficient quadratic growth curve model. BMI was log transformed to correct for right skewness in observations. Where log BMI has been observed on subjects j on occasions i.

$$LN(BMI)_{ij} = (\beta_1 + \zeta_{1j}) + (\beta_2 + \zeta_{2j})t_{ij} + \beta_3 t_{ij}^2 + \epsilon_{ij}$$

In addition, covariates for gender, ethnicity, smoking, deprivation are included in the model as covariates for the intercept and slope equations. ζ_1j and ζ_2j are assumed to have a bi-variate normal distribution with unstructured covariance matrix.

<u>Systolic Blood Pressure model</u>

The model had a random coefficient linear growth curve model. Where systolic blood pressure has been observed on subjects *j* on occasions *i*.

$$SBP_{ij} = (\beta_1 + \zeta_{1j}) + (\beta_2 + \zeta_{2j})t_{ij} + \epsilon_{ij}$$

In addition, covariates for gender and BMI are included in the model as covariates for the intercept and slope equations. ζ_{1j} and ζ_{2j} are assumed to have a bi-variate normal distribution with unstructured covariance matrix.

<u>Total Cholesterol model</u>

The model had a random coefficient linear growth curve model. Where Total Cholesterol has been observed on subjects *j* on occasions *i*.

$$CHOL_{ij} = (\beta_1 + \zeta_{1j}) + (\beta_2 + \zeta_{2j})t_{ij} + \epsilon_{ij}$$

In addition, covariates for gender, ethnicity, BMI and deprivation are included in the model as covariates for the intercept and slope equations. ζ_{1j} and ζ_{2j} are assumed to have a bi-variat normal distribution with unstructured covariance matrix.

<u>HDL Cholesterol model</u>

The model had a random coefficient linear growth curve model. Where HDL cholesterol has been observed on subjects *j* on occasions *i*.

$$HDL_{ij} = (\beta_1 + \zeta_{1j}) + (\beta_2 + \zeta_{2j})t_{ij} + \epsilon_{ij}$$

In addition, covariates for gender, BMI and deprivation are included in the model as covariates for the intercept and slope equations. ζ_{1j} and ζ_{2j} are assumed to have a bi-variate normal distribution with unstructured covariance matrix.

<u>HbA1c model</u>

The model had a random coefficient linear growth curve model. Where HbA1c has been observed on subjects *j* on occasions *i*.

$$HbA1c_{ij} = (\beta_1 + \zeta_{1j}) + (\beta_2 + \zeta_{2j})t_{ij} + \epsilon_{ij}$$

In addition, covariates for gender, ethnicity, smoking, BMI and deprivation are included in the model as covariates for the intercept and slope equations. ζ_{1j} and ζ_{2j} are assumed to have a bi-variate normal distribution with unstructured covariance matrix.

Table 5.6.1: Growth Curve Models of body mass index and cardiometabolic risk factors.

	**Log BMI Growth model**	**SBP Growth Model**	**Total Cholesterol**	**HDL cholesterol**	**HbA1c**
N	9742	9293	8496	8509	8160
β1 [intercept]	2.76255 (0.0293)***	92.327 (1.994)***	5.37859 (0.427)***	1.3093 (0.168)***	3.6399 (0.056)***
β2 [age]	0.0161 (0.0009)***	0.4168 (0.028)***	0.01389 (0.007)*	0.01562 (0.003)***	0.0194 (0.0006)***
β3 [age2]	-0.0001 (0.0000)***				
β4 [male]	0.04251 (0.012)**	14.44545 (2.232)***	1.0948 3(0.144)***	0.04588 (0.056)	
β5 [male_age]	-0.00059 (0.0002)**	-0.1918 (0.035)	-0.02416 (0.002)***	-0.0051 (0.001)*	
β6 [ethnicity]			-0.35081 (0.079)***		0.38067 (0.061)***
B7 [Smoking]	-0.04629 (0.003)				0.348282 (0.123)**
B8 [smoking_age]					-0.00365 (0.002)*
B9 [IMD2]	0.00082 (0.002)			-0.00957 (0.009)	
β10 [IMD3]	0.00566 (0.003)*			-0.01343 (0.010)	
β11 [IMD4]	0.01099 (0.003)***			-0.0457 (0.011)***	
β12 [IMD5]	0.0189 (0.004)***		0.40574 (0.239)*	-0.06969 (0.013)***	0.0367 (0.016)*
β13 [IMD5_age]			-0.00722 (0.004)*		
B14 BMI]		0.4213 (0.032)***	0.03589 (0.015)**	-0.00063 (0.006)	0.02723 (0.001)***
B15 [BMI_age]			-0.00067 (0.0002)**	-0.00031 (0.0001)***	
stdev intercept	0.33217	47.3207 (1.943)	2.543 (0.156)	1.33309 (0.048)	1.438147 (0.176)
stdev age	0.00493	0.7272	0.0375 (0.003)	0.02045 (0.001)	0.0188 (0.002)
Correlation	-0.8746	-0.9819	-0.9668	-0.979	-0.94904
Stdev error	1.364	13.564	0.815	0.239	0.232
Log likelihood	-58885	-77840	-23391	-6203	-8194

5.6.3 HbA1c Trajectories

Glycaemic Trajectory in Non-Diabetics

At baseline, HbA1c estimates for HbA1c are used to determine an individual's HbA1c and glycaemic status. The ELSA statistical model is used to estimate an individual's linear changes in HbA1c every year. Random coefficient growth factors are re-estimated using the bivariate covariance structure from the ELSA HbA1c growth model. The Whitehall II analysis was not used for the HbA1c trajectory due to the limited number of observations for HbA1c in this analysis.

HbA1c trajectory in undiagnosed diabetes

At diabetes onset HbA1c is assumed to increase at a rate of 0.318% per year based on analyses of undiagnosed diabetes patients in the UK Biobank (53). In this study they identified a group of undiagnosed diabetes cases. These cases of undiagnosed diabetes were followed up from GP records to identify the time and median HBA1c score at diagnosis. On study entry the median HbA1c was 6.8% (IQR 6.6-7.4). Median time to diabetes diagnosis was 2.2 years (IQR 2.0 – 2.4), and mean HbA1c was 7.5% (IQR 6.8-9.5).

HbA1c trajectory in type 2 diagnosed diabetics

Following a diagnosis of diabetes in the simulation all individuals experience an initial fall in HbA1c due to changes in diet and lifestyle as observed in the UKPDS trial (54). We have estimated the expected change in HbA1c conditional on HbA1c at diagnosis by fitting a simple linear regression to three aggregate outcomes reported in the study. These showed that the change in HbA1c increases for higher HbA1c scores at diagnosis. The regression parameters to estimate change in HbA1c are reported in Table 5.6.2. In the PSA parameters were sampled from a multivariate normal distribution with the covariance matrix reported below.

Table 5.6.2: Estimated change in HbA1c following diabetes diagnosis

	Mean	Standard error	Covariance matrix	
Change in HbA1c Intercept	-2.99	.048	0.00558644	
HbA1c at baseline	0.55	0.40	-0.0390114	0.27987476

After this initial reduction in HbA1c the longitudinal trajectory of HbA1c is estimated using the UKPDS outcomes model (54) rather than the Whitehall II statistical analysis. The UKPDS dataset is made up of a newly diagnosed diabetic population. As part of the UKPDS Outcomes model, longitudinal trial data were analysed using a random effects model. The coefficients of the model are reported in Table 5.6.3. Individual coefficients were sampled from a multivariate normal distribution using the standard errors reported below and correlation matrix provided from bootstrapped samples reported in the UKPDS Outcomes model.

Table 5.6.3: Coefficient estimates for HbA1c estimated from UKPDS data

	Mean Coefficient	Coefficient standard error
Intercept	-0.024	0.017
Log transformation of year since diagnosis	0.144	0.009
Binary variable for year after diagnosis	-0.333	0.05
HbA1c score in last period	0.759	0.004
HbA1c score at diagnosis	0.085	0.004

The model can be used to predict HbA1c over time from the point of diagnosis. The model suggests that HbA1c increases with time. A graph illustrating change in HbA1c over time from two different HbA1c levels at diagnosis is illustrated in Figure 5.6.2.

Figure 5.6.2: Trajectory of HbA1c estimated from UKPDS longitudinal model

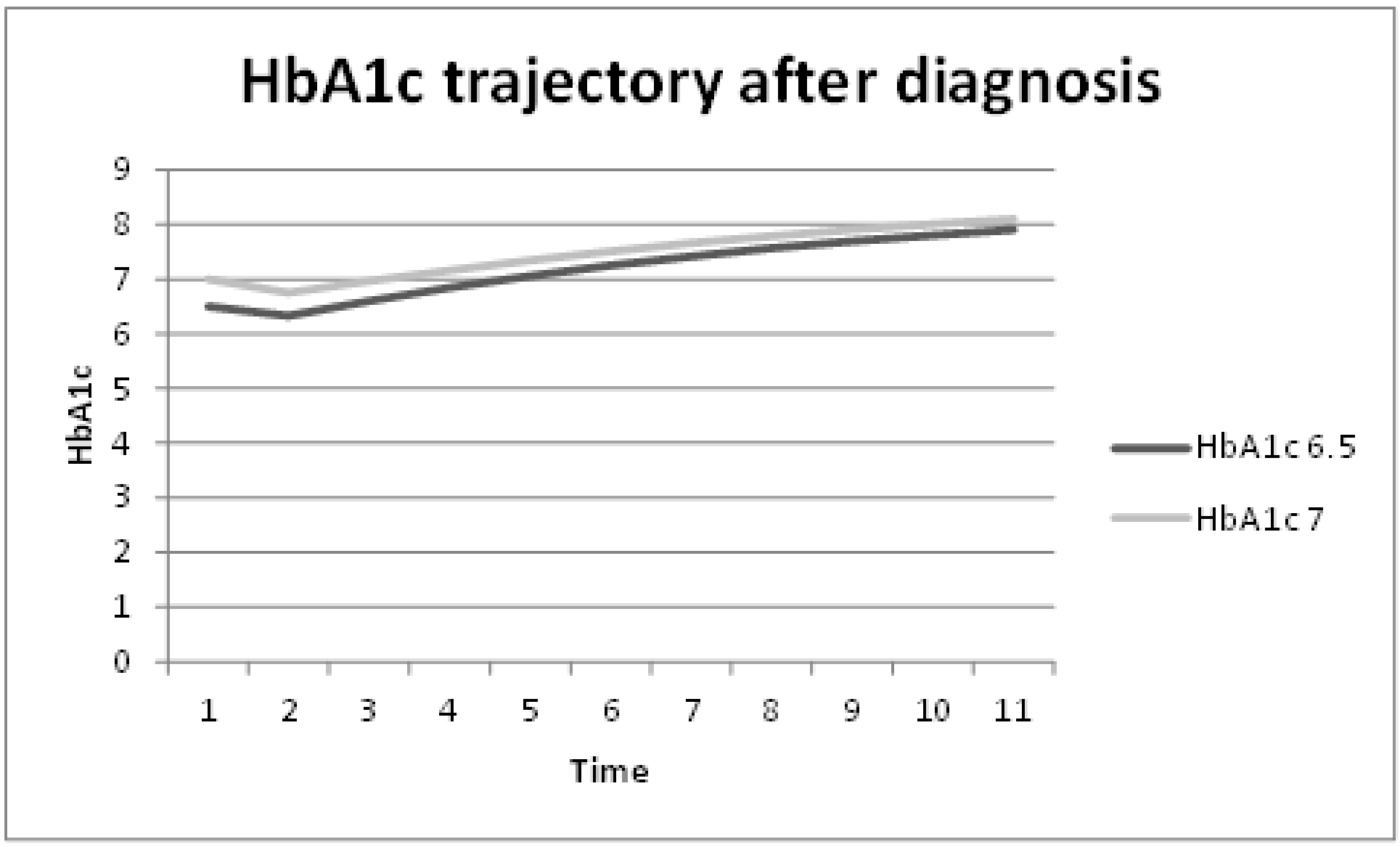

5.6.4 Cholesterol Trajectories

Total Cholesterol and HDL Cholesterol Trajectories in Individuals not receiving Statins

At baseline, an individual's total and HDL cholesterol is determined from the HSE data. In the simulation, individuals aged 60 years and younger have annual changes in total and HDL cholesterol according to the estimates from the statistical analysis of the Whitehall II cohort. The slope of total and HDL cholesterol are assumed to be linear with time. These growth factors are estimated in the model to be conditional on cholesterol at baseline, age at baseline, sex, and an error parameter to reflect unobservable variability in growth trajectories between individuals. As with latent glycaemia, changes in total cholesterol are also influenced by the trajectory of BMI. For individuals aged 61 and over the ELSA statistical models for Total and HDL cholesterol are used to estimate annual change in cholesterol. As individuals transition between the trajectory models the random coefficient factors are updated allowing current observations to inform the trajectories in Total and HDL cholesterol.

Total Cholesterol and HDL Cholesterol Trajectories in Individuals receiving Statins

During the simulation process, individuals are prescribed statins to reduce their risk of cardiovascular disease. It is assumed within the model that the statins are effective in reducing an individual's total cholesterol, and an average effect is applied to all patients receiving statins. A recent HTA reviewed the literature on the effectiveness and cost-effectiveness of statins in individuals with acute coronary syndrome (55). This report estimated the change in LDL cholesterol for four statin treatments and doses compared with placebo from a Bayesian meta-analysis. The analysis estimated a reduction in LDL cholesterol of -1.45 for simvastatin. This estimate was used to describe the effect of statins in reducing total cholesterol. It was assumed that the effect was instantaneous upon receiving statins and maintained as long as the individual receives statins. It was also assumed that individuals receiving statins no longer experienced annual changes in total cholesterol. HDL cholesterol was assumed constant over time if patients receive statins.

Non-adherence to statin treatment is a common problem. Two recent HTAs reviewed the literature on continuation and compliance with statin treatment. They both concluded that there was a lack of adequate reporting, but that the proportion of patients fully compliant with treatment appears to decrease with time,

particularly in the first 12 months after initiating treatment, and can fall below 60% after five years (18, 55). Although a certain amount of non-compliance is included within trial data, clinical trials are not considered to be representative of continuation and compliance in general practice. A yearly reduction in statin compliance used in the HTA analysis is reported in Table 5.6.4. It is based on the published estimate of compliance for the first five years of statin treatment for primary prevention in general clinical practice (55). Compliance declines to a minimum of 65% after five years of treatment. It is assumed that there is no further drop after five years.

Table 5.6.4: Proportion of patients assumed to be compliant with statin treatment, derived from Table 62 in (55)

Year after statin initiation	1	2	3	4	5
Proportion compliant	0.8	0.7	0.68	0.65	0.65

In the simulation, we assume in the base case that only 65% of individuals initiate statins when they are deemed eligible. However, those that initiate statins remain on statins for their lifetime. Those who refuse statins may be prescribed them again at a later date.

5.6.5 Blood Pressure Trajectories

SBP Trajectories in Individuals Not receiving Anti-hypertensive treatment

At baseline an individual's SBP is determined from the HSE data. In the simulation, individuals' aged 60 and younger experience SBP changes every year according to the estimates from the statistical analysis of the Whitehall II cohort. The annual change in SBP is assumed to be linear with time. The growth factors are estimated in the model to be conditional on SBP at baseline, age at baseline, sex, ethnicity, family history of cardiovascular disease, smoking and an error parameter to reflect unobservable variability in growth trajectories between individuals. From ages 61 onwards the ELSA statistical model for systolic blood pressure is used to estimate annual changes. The random coefficient factors are updated using the bivariate covariance matrix for intercept and slope factors.

SBP Trajectories in Individuals receiving anti-hypertensive treatment

During the simulation process, if individuals are identified as having SBP higher than 160mm Hg, or SBP higher than 140mm Hg with comorbid diabetes, cardiovascular disease, or 10 year risk of cardiovascular disease greater than 20%, they will be prescribed anti-hypertensive treatment in line with the National Institute for Health and Care Excellence (NICE) guidelines (56). The change in SBP following initiation of calcium channel blockers was estimated in a meta-analysis of anti-hypertensive treatments (57). This study identified an average change in SBP of -8.4 for monotherapy with calcium channel blockers. In the simulation model it is assumed that this reduction in SBP is maintained for as long as the individual receives anti-hypertensive treatment. Once an individual is receiving anti-hypertensive treatment it is assumed that their SBP is stable and does not change over time, which implicitly assumes that patients continue to be well managed for their hypertension. For simplicity we do not explicitly simulate treatment switching. The assumed zero flat trajectory in systolic blood pressure whilst receiving anti-hypertensives is supported by the analysis of the ELSA dataset in which self-reported use of anti-hypertensives was included as a covariate for age-related change in systolic blood pressure. The analysis found that most of the observed changes in systolic blood pressure were removed if individuals were taking anti-hypertensives.

5.7 Metabolic Risk factor screening

We assume that individuals eligible for statins and anti-hypertensive treatment will be identified through opportunistic screening if they meet certain criteria and attend the GP for at least one visit in the simulation period. The base case has been designed to represent a health system with moderate levels of screening for hypertension, and dyslipidaemia. Alternative assumptions for more or less intensive opportunistic screening can be assumed.

5.8 Diagnosis and Treatment Initiation

It is assumed that there are three, non-mutually exclusive outcomes from the vascular checks or opportunistic screening. Firstly, that the patient receives statins to reduce cardiovascular risk. Secondly, that the patient has high blood pressure and should be treated with anti-hypertensive medication. The following threshold estimates were used to determine these outcomes.

1. Statins are initiated if the individual has greater than or equal to 20% 10 year CVD risk estimated from the QRISK2 2012 algorithm (12).
2. Anti-hypertensive treatment is initiated if systolic blood pressure is greater than 160. If the individual has a history of CVD, diabetes or a CVD risk >10%, the threshold for systolic blood pressure is 140 (56).

5.9 Diabetes Diagnosis

The HSE repeatedly demonstrates that a large proportion of the English population have a HbA1c test score above 6.5%, and do not report a diagnosis of diabetes. Therefore, it is important to allow some undiagnosed cases of diabetes to persist in the model. Diabetes onset is simulating using the QDiabetes 2018 risk algorithm (58). The model estimates the QDiabetes score, with HBA1c covariates, for undiagnosed individuals within the population in each annual cycle. This score describes the individuals 10 year probability of being diagnosed with diabetes conditional on demographic, socioeconomic, ethnicity, medical history and HbA1c score. The QDiabetes 2018 model was chosen to describe individual’s risk of diabetes in the model to describe differential risks across individuals. The one-year probability of diabetes uses the coefficients from the QDiabetes 2018 tool, applied to a baseline risk calibrated to observed data on the rate of diabetes diagnoses in this population.

The QDiabetes 2018 risk equation can be used to calculate the probability of type 2 diabetes. The equation estimates the probability of a diabetes in the next period conditional on the coefficients listed in Table 5.9.1. Parameter samples were taken according to a normal distribution defined by respective standard errors. The equation for the probability of an event in the next period is calculated as

$$p(Y = 1) = 1 - S(1)^{\theta}$$

$$\theta = \sum \beta X$$

The probability of an event is calculated from the survival function, S(1), at 1 year raised to the power of θ, where θ is the sum product of the coefficients, β and covariates, X.

Underlying survival curves for men and women were extracted from the QDiabetes 2018 open source file. Mean estimates for the continuous variables were also reported in the open source files.

All individuals have a simulated HbA1c at diagnosis test result to reflect HbA1c at diagnosis observed in the population with median 7.5% (53). In each cycle an individual with diabetes onset is diagnosed with Diabetes if they meet the following criteria.

- Diagnosed hypertension
- Micro/macro vascular diabetes complication
- HbA1c above simulated HbA1c at diagnosis, median 7.5% (53)

Table 5.9.1: Coefficients from the 2018 QDiabetes risk equation and estimated standard errors (58)

Estimated coefficients adjusting for individual characteristics									
	Women		Men			Women		Men	
**Covariates**	**Mean**	**Standard error**	**Mean**	**Standard error**	**Interaction terms**	**Mean**	**Standard error**	**Mean**	**Standard error**
White	0.0000	10%	0.0000	10%	Age1*former smoker	-0.8125	0.035	-1.0013	0.776
Indian	0.5991	10%	0.6757	10%	Age1*light smoker	-0.9085	0.066	-0.8916	3.341
Pakistani	0.7832	10%	0.8315	10%	Age1*moderate smoker	-1.8558	0.231	-1.7075	3.075
Bangladeshi	1.1947	10%	1.0969	10%	Age1*Heavy smoker	0.6023	0.308	0.4507	3.529
Other Asian	0.7142	10%	0.7682	10%	Age1*AF	-0.0345	0.922	-0.1085	1.406
Caribbean	0.1195	10%	0.2090	10%	Age1*renal disease	-0.2728	0.528	-0.6141	3.403
Black African	0.0137	10%	0.3809	10%	Age1*hypertension	25.4412	0.450	27.6706	6.793
Chinese	0.5709	10%	0.3424	10%	Age1*Diabetes	-6.8076	0.369	-7.4006	2.558
Other	0.1709	10%	0.2205	10%	Age1*BMI	0.0005	0.617	0.0002	0.654
Non-smoker	0.0000	10%	0.0000	10%	Age1*family history diabetes	0.0009	0.050	0.0007	3.584
Former smoker	0.0658	10%	0.1159	10%	Age1*SBP	0.0023	0.003	0.0014	0.030
Light smoker	0.1458	10%	0.1462	10%	Age1*Townsend	-0.0043	0.007	-0.0012	0.510
Moderate smoker	0.1526	10%	0.1078	10%	Age2*former smoker	0.0001	10%	0.0002	10%
Heavy smoker	0.3079	10%	0.1985	10%	Age2*light smoker	0.0004	10%	0.0005	10%
Age 1*	3.5655	10%	4.0193	10%	Age2*moderate smoker	-0.0523	10%	-0.0592	10%
Age 2*	-0.0056	10%	-0.0048	10%	Age2*Heavy smoker	0.0141	10%	0.0156	10%
BMI*	2.5043	10%	0.8183	10%	Age2*AF	-0.8125	10%	-1.0013	10%
Ratio Total / HDL chol	-0.0429	10%	-0.1256	10%	Age2*renal disease	-0.9085	10%	-0.8916	10%
SBP	8.7368	10%	8.0512	10%	Age2*hypertension	-1.8558	10%	-1.7075	10%
Townsend	-0.0782	10%	-0.1465	10%	Age2*Diabetes	0.6023	10%	0.4507	10%
AF	0.0359	10%	0.0252	10%	Age2*BMI	-0.0345	10%	-0.1085	10%
Rheumatoid arthritis	0.5498	10%	0.4554	10%	Age2*family history diabetes	-0.2728	10%	-0.6141	10%
Renal disease	0.1687	10%	0.1382	10%	Age2*SBP	25.4412	10%	27.6706	10%
Hypertension	0.1644	10%	0.1455	10%	Age2*Townsend	-6.8076	10%	-7.4006	10%
Diabetes	1.1250	10%	0.2596	10%					
Family history of Diabetes	0.2891	10%	0.2852	10%					
AF Atrial Fibrillation SBP systolic blood pressure * covariates transformed with fractional polynomials									

5.10 Comorbid Outcomes and Mortality

In every model cycle individuals within the model are evaluated to determine whether they have a clinical event, including mortality, within the cycle period. In each case the simulation estimates the probability that an individual has the event and uses a random number draw to determine whether the event occurred.

5.10.1 Cardiovascular Disease

First Cardiovascular event

Several statistical models for cardiovascular events were identified in a review of economic evaluations for diabetes prevention (59). The UKPDS outcomes model (60), Framingham risk equation (61) and QRISK2 (62) have all been used in previous models to estimate cardiovascular events. The Framingham risk equation was not adopted because, unlike the QRISK2 model, it is not estimated from a UK population. The UKPDS outcomes model would be ideally suited to estimate the risk of cardiovascular disease in a population diagnosed with type 2 diabetes. Whilst this is an important outcome of the cost-effectiveness model, there was concern that it would not be representative of individuals with normal glucose tolerance or impaired glucose regulation. Recent analyses show that the UKPDS over-predicts cardiovascular outcomes in newly diagnosed diabetes patients (63). It was important that reductions in cardiovascular disease risk in these populations were represented to capture the population-wide benefits of public health interventions. The QRISK2 model was selected for use in the cost-effectiveness model because it is a validated model of cardiovascular risk in a up to date UK population and could be used to generate probabilities for diabetic and non-diabetic populations. We considered using the UKPDS outcomes model specifically to estimate cardiovascular risk in patients with type 2 diabetes. However, it would not be possible to control for shifts in absolute risk generated by the different risk scores due to different baselines and covariates. This would lead to some individuals experiencing counterintuitive and favourable shifts in risk after onset of type 2 diabetes. Therefore, we decided to use diabetes as a covariate adjustment to the QRISK2 model to ensure that the change in individual status was consistent across individuals. In recent years new cardiovascular risk models have been developed, however none are more relevant to the UK setting than the QRISK functions.

The probability of the first cardiovascular event is estimated from the QRISK2 predicted model of cardiovascular disease (20). The QRISK2 is a validated risk prediction algorithm to identify individuals at high risk of cardiovascular disease. The algorithm was developed from UK data and incorporates social deprivation and ethnicity. We accessed the coefficients and algorithm from the online QRISK website http://www.qrisk.org/ (accessed 2019). The QRISK2 equation estimates the probability of a cardiovascular event in the next 10 years conditional on ethnicity, smoking status, age, BMI, ratio of total/HDL cholesterol, Townsend score, atrial fibrillation, rheumatoid arthritis, renal disease, hypertension, diabetes, and family history of cardiovascular disease. Data on all these variables was available from the HSE.

First Cardiovascular event calibration

In version 4 of the model the QRISK2 algorithm was updated with 2014 QRISK coefficients. The 2014 version did not report the 1 year survival function. For the 2014 version we estimated the 1 year parameter using a simple calibration process to match the CVD incidence in a simulated adult population matching the WRAP trial. CVD rates were calibrated to 5 years incidence of CVD events. A simple calibration approach was taken to identify a parameter estimate for the 1 year survival parameter for men and women that would simulate the estimated CVD incidence reported from the 5 year follow-up data within a 2% margin for error. This data was based on cardiovascular events to include, MI, percutaneous coronary intervention (PCI), Experienced bypass (CABG), invasive cardiovascular procedure, Transient Ischaemic Attack or stroke reported in GP records.

At 5 years follow-up GP records for CVD history were available for 859 women, and 408 men. Of those 14 and 18 had experienced a CVD event respectively. For the calibration were identified survival parameters for men and women that would generate an incidence of 3.26 per 1000 person years for women and 8.824 per 1000

person years for men in the brief intervention simulation. These parameters were estimated with a simple iterative process simulating 20,000 randomly selected individuals. The final estimates were tested against other random samples. Starting values were selected from using the survival parameters reported in the QRISK 2012 source code.

First Cardiovascular event inputs

Table 5.10.1 reports the coefficient estimates for the QRISK2 algorithm. The standard errors were not reported within the open source code. Where possible, standard errors were imputed from a previous publication of the risk equation (12). Coefficients that were not reported in this publication were assumed to have standard errors of 20%. Parameters were sampled from a normal distribution defined by their respective standard errors.

Table 5.10.1: Coefficients from the 2014 QRISK2 risk equation and estimate standard errors

Estimated coefficients adjusting for individual characteristics									
	**Women**		**Men**			**Women**		**Men**	
**Covariates**	**Mean**	**Standard error**	**Mean**	**Standard error**	**Interaction terms**	**Mean**	**Standard error**	**Mean**	**Standard error**
White	0.0000	0.0000	0.0000	0.0000	Age1*smoke1	0.6891	0.035	0.9244	0.776
Indian	0.2672	0.0537	0.2785	0.0425	Age1*smoke2	0.6943	0.066	1.9598	3.341
Pakistani	0.7148	0.0698	0.6068	0.0547	Age1*smoke3	-1.6952	0.231	2.9994	3.075
Bangladeshi	0.3703	0.1073	0.7104	0.0727	Age1*smoke4	-1.2150	0.308	5.0371	3.529
Other Asian	0.2074	0.1071	0.8626	0.0845	Age1*AF	-3.5855	0.922	8.2354	1.406
Caribbean	-0.1744	0.0619	3.8735	0.0641	Age1*renal disease	-3.0767	0.528	-3.9747	3.403
Black African	-0.3272	0.1275	0.1347	0.1094	Age1*hypertension	-4.0295	0.450	7.8738	6.793
Chinese	-0.2201	0.1721	-0.1558	0.1538	Age1*Diabetes	-3.3145	0.369	5.0624	2.558
Other	-0.2090	0.0793	-3.7728	0.0734	Age1*BMI 1	-5.5934	0.617	33.5438	0.654
Non-smoker	0.0000	0.0000	0.1526	0.0000	Age1*BMI 2	64.3636	0.050	-129.9767	3.584
Former smoker	0.1947	0.0152	0.0132	0.0108	Age1*family history CVD	0.8605	0.050	1.9280	3.584
Light smoker	0.6229	0.0220	0.0644	0.0166	Age1*SBP	-0.0509	0.003	0.0523	0.030
Moderate smoker	0.7406	0.0178	1.4235	0.0148	Age1*Townsend	0.1519	0.007	-0.1731	0.510
Heavy smoker	0.9134	0.0194	0.3021	0.0143	Age2*smoke1	-0.1765	0.001	-0.0034	1.594
Age 1	3.8735		-17.6226		Age2*smoke2	-0.2324	0.000	-0.0051	4.737
Age 2	0.1347		0.0242		Age2*smoke3	0.2734	0.002	0.0003	4.627
BMI 1*	-0.1558	0.0423	1.7320	0.0299	Age2*smoke4	0.1433	0.003	0.0031	5.373
BMI2 *	-3.7728		-7.2312		Age2*AF	0.4987	0.010	0.0073	2.890
Ratio Total / HDL chol	0.1526	0.0044	0.1751	0.0022	Age2*renal disease	0.4393	0.007	-0.0262	5.654
SBP	0.0132	0.0045	0.0102	0.0046	Age2*hypertension	0.6904	0.005	0.0086	3.763
Townsend	0.0644	0.0068	0.0298	0.0048	Age2*Diabetes	0.4865	0.004	-0.0002	0.193
AF	1.4235	0.0310	0.9891	0.1018	Age2*BMI 1	1.5223	0.007	0.0812	2.110
Rheumatoid arthritis	0.3021	0.0319	0.2542	0.0445	Age2*BMI 2	-12.7413		-0.2559	
Renal disease	0.8615	0.0639	0.7950	0.0702	Age2*family history CVD	-0.2757	0.001	-0.0057	5.321
Hypertension	0.5889	0.0115	0.6229	0.0112	Age2*SBP	0.0074	0.000	-0.0001	0.058
Diabetes	1.1350	0.0199	0.9373	0.0175	Age2*Townsend	-0.0487	0.000	-0.0011	0.601
Family history of CVD	0.5134	0.0122	0.5923	0.0111					
AF Atrial Fibrillation CVD Cardiovascular disease SBP systolic blood pressure * covariates transformed with fractional polynomials									

The QRISK2 risk equation can be used to calculate the probability of a cardiovascular event including: coronary heart disease (angina or myocardial infarction), stroke, or transient ischaemic attacks, fatality due to cardiovascular disease. The equation estimates the probability of a cardiovascular event in the next period conditional on the coefficients listed in Table **5.10.1**.

The equation for the probability of an event in the next period is calculated as

$$p(Y = 1) = 1 - S(1)^{\theta}$$

$$\theta = \sum \beta X$$

The probability of an event is calculated from the survival function at 1 year, S(1), raised to the power of θ, where θ is the sum product of the coefficients reported in Table 5.10.1 multiplied by the individual's characteristics. Underlying survival curves for men and women were extracted from the QRISK2 open source file. Mean estimates for the continuous variables were also reported in the open source files.

We modified the QRISK2 assumptions regarding the relationship between IGR, diabetes and cardiovascular disease. Firstly, we assumed that individuals with HbA1c>6.5 have an increased risk of cardiovascular disease even if they have not received a formal diagnosis. Secondly, risk of cardiovascular disease was assumed to increase with HbA1c for test results greater than 6.5 to reflect observations from the UKPDS that HbA1c increases the risk of MI and Stroke (60). Thirdly, prior to type 2 diabetes (HbA1c>6.5) HbA1c is linearly associated with cardiovascular disease. A study from the EPIC Cohort has found that a unit increase in HbA1c increases the risk of coronary heart disease by a hazard ratio of 1.25, after adjustment for other risk factors (64). We apply this risk ratio to linearly increase risk above the mean HBA1c observed in the HSE 2011 cohort. A linear risk reduction was applied at HbA1c levels below the HSE mean.

The QRISK2 algorithm identifies which individuals experience a cardiovascular event but does not specify the nature of the event. The nature of the cardiovascular event was determined independently. A targeted search of recent Health Technology appraisals of cardiovascular disease was performed to identify a model for the progression of cardiovascular disease following a first event. A Health Technology Assessment (HTA) assessing statins gives age and sex specific distributions of CVD, which were used to assign all QRISK2 events (18). Table 5.10.2 reports the probability of cardiovascular outcomes by age and gender. Stakeholders suggested that there may be different relationships between the risk factors and the different types of CVD (e.g. hypertension is more of a risk factor for stroke). However, we decided not to incorporate these differential factors in evaluating the risk of cardiovascular event types into the model due to a lack of evidence.

Table 5.10.2: The probability distribution of cardiovascular events by age and gender

	**Age**	**Stable angina**	**Unstable angina**	**MI rate**	**Fatal CHD**	**TIA**	**Stroke**	**Fatal CVD**
**Men**	45-54	0.307	0.107	0.295	0.071	0.060	0.129	0.030
	55-64	0.328	0.071	0.172	0.086	0.089	0.206	0.048
	65-74	0.214	0.083	0.173	0.097	0.100	0.270	0.063
	75-84	0.191	0.081	0.161	0.063	0.080	0.343	0.080
	85+	0.214	0.096	0.186	0.055	0.016	0.351	0.082
**Women**	45-54	0.325	0.117	0.080	0.037	0.160	0.229	0.054
	55-64	0.346	0.073	0.092	0.039	0.095	0.288	0.067
	65-74	0.202	0.052	0.121	0.081	0.073	0.382	0.090
	75-84	0.149	0.034	0.102	0.043	0.098	0.464	0.109
	85+	0.136	0.029	0.100	0.030	0.087	0.501	0.117

Subsequent Cardiovascular events

After an individual has experienced a cardiovascular event, it is not possible to predict the transition to subsequent cardiovascular events using QRISK2. As with assigning first CVD events, the probability of subsequent events was estimated from the HTA evaluating statins (18). This study reported the probability of future events conditional on the nature of the previous event. Table 5.10.3 reports an example of the probabilities within a year of transitioning from stable angina, unstable angina, myocardial infarction (MI), transient ischemic attack (TIA) or stroke for individuals by age group. The input parameters and covariance matrices used to generate the transition probabilities in Table 5.10.3 are detailed in Table 5.10.4.

Table 5.10.3: Probability of cardiovascular event conditional on age and status of previous event (column 1)

	Stable angina	Unstable angina 1	Unstable angina 2	MI 1	MI 2	TIA	Stroke 1	Stroke 2	CHD death	CVD death
Age 45										
Stable angina	0.9946	0.0013	0	0.0032	0	0	0	0	0.0009	0
Unstable angina (1st yr)	0	0	0.9127	0.0495	0	0	0	0	0.0362	0.0016
Unstable angina (subsequent)	0	0	0.9729	0.0186	0	0	0	0	0.0081	0.0004
MI (1st yr)	0	0	0	0.128	0.8531	0	0.0015	0	0.0167	0.0007
MI (subsequent)	0	0	0	0.0162	0.978	0	0.0004	0	0.0052	0.0002
TIA	0	0	0	0.0016	0	0.9912	0.0035	0	0.0024	0.0013
Stroke (1st yr)	0	0	0	0.0016	0	0	0.0431	0.9461	0.0046	0.0046
Stroke (subsequent)	0	0	0	0.0016	0	0	0.0144	0.9798	0.0021	0.0021
Age 55										
Stable angina	0.9874	0.0029	0	0.0062	0	0	0	0	0.0035	0
Unstable angina (1st yr)	0	0	0.8859	0.0497	0	0	0	0	0.0617	0.0027
Unstable angina (subsequent)	0	0	0.9548	0.0348	0	0	0	0	0.01	0.0004
MI (1st yr)	0	0	0	0.1152	0.8483	0	0.0032	0	0.0319	0.0014
MI (subsequent)	0	0	0	0.0179	0.9716	0	0.001	0	0.0091	0.0004
TIA	0	0	0	0.0031	0	0.9626	0.0181	0	0.0092	0.007
Stroke (1st yr)	0	0	0	0.0031	0	0	0.0459	0.9288	0.0111	0.0111
Stroke (subsequent)	0	0	0	0.0031	0	0	0.0186	0.9685	0.0049	0.0049
Age 65										
Stable angina	0.976	0.006	0	0.011	0	0	0	0	0.007	0
Unstable angina (1st yr)	0	0	0.8435	0.0488	0	0	0	0	0.1031	0.0046
Unstable angina (subsequent)	0	0	0.9244	0.0632	0	0	0	0	0.0119	0.0005
MI (1st yr)	0	0	0	0.1019	0.8287	0	0.0068	0	0.0599	0.0027
MI (subsequent)	0	0	0	0.0185	0.9634	0	0.0022	0	0.0152	0.0007
TIA	0	0	0	0.0055	0	0.9174	0.0423	0	0.0185	0.0163
Stroke (1st yr)	0	0	0	0.0055	0	0	0.0481	0.8944	0.026	0.026
Stroke (subsequent)	0	0	0	0.0055	0	0	0.0223	0.9514	0.0104	0.0104
Age 75										
Stable angina	0.9681	0.0091	0	0.0158	0	0	0	0	0.007	0
Unstable angina (1st yr)	0	0	0.7789	0.0466	0	0	0	0	0.1671	0.0074
Unstable angina (subsequent)	0	0	0.8733	0.1122	0	0	0	0	0.0139	0.0006
MI (1st yr)	0	0	0	0.0874	0.7849	0	0.0141	0	0.1088	0.0048
MI (subsequent)	0	0	0	0.0178	0.953	0	0.0047	0	0.0235	0.001
TIA	0	0	0	0.008	0	0.8588	0.0828	0	0.0185	0.0319
Stroke (1st yr)	0	0	0	0.008	0	0	0.0446	0.8302	0.0586	0.0586
Stroke (subsequent)	0	0	0	0.008	0	0	0.0246	0.9262	0.0206	0.0206
Age 85										
Stable angina	0.9601	0.0122	0	0.0207	0	0	0	0	0.007	0
Unstable angina (1st yr)	0	0	0.6873	0.0425	0	0	0	0	0.2587	0.0115
Unstable angina (subsequent)	0	0	0.7878	0.1955	0	0	0	0	0.016	0.0007
MI (1st yr)	0	0	0	0.0711	0.7053	0	0.0278	0	0.1875	0.0083
MI (subsequent)	0	0	0	0.016	0.9394	0	0.0091	0	0.034	0.0015
TIA	0	0	0	0.0104	0	0.838	0.0961	0	0.0185	0.037
Stroke (1st yr)	0	0	0	0.0104	0	0	0.0446	0.702	0.1215	0.1215
Stroke (subsequent)	0	0	0	0.0104	0	0	0.0252	0.8894	0.0375	0.0375

Table 5.10.4: Multivariate normal parameter inputs distributions for second cardiovascular events

	Mean	Covariance matrix					
Non fatal MI in 1st year) | MI - Age coeff	-0.0100	0.00013					
Non fatal MI in 1st year) | MI - Constant coeff	-1.4481	-0.008191	0.534802				
Non fatal stroke in 1st year) | MI - Age coeff	0.0786	0.000013	-0.00083	0.001832			
Non fatal stroke in 1st year) | MI - Constant coeff	-9.9099	-0.000822	0.054822	-0.13357	9.90642		
Vascular death in 1st year) | MI - Age coeff	0.0652	0.000013	-0.000847	0.000029	-0.00196	0.0002	
Vascular death in 1st year) | MI - Constant coeff	-6.8197	-0.00084	0.056003	-0.00195	0.13225	-0.0163	1.1841
Any event in yrs2+ |MI - Age coeff	0.0253	0.0002					
Any event in yrs2+ |MI - Constant coeff	-4.9566	-0.0121	0.8330				
Non fatal stroke in yr2+) | MI - Age coeff	0.0777	0.001201					
Non fatal stroke in yr2+) | MI - Constant coeff	-7.1720	-0.086667	6.3698				
Vascular death in yr2+) | MI - Age coeff	0.0475	0.000165	-0.010929	0.000294			
Vascular death in yr2+) | MI - Constant coeff	-3.2410	-0.010847	0.733099	-0.01993	1.38513		
Non fatal MI in 1st year) | USA - Age coeff	0.0032	0.000152					
Non fatal MI in 1st year) | USA - Constant coeff	-3.0591	-0.009735	0.649701				
Vascular death in 1st year) | USA - Age coeff	0.0562	0.0000085	-0.000542	0.000081			
Vascular death in 1st year) | USA - Constant coeff	-5.7140	-0.000544	0.035984	-0.00577	0.42026		
Any event in yrs2+ |USA - Age coeff	0.0515	0.000039					
Any event in yrs2+ |USA - Constant coeff	-5.9318	-0.002788	0.203419				
Vascular death in yr2+) | USA - Age coeff	-0.0418	0.00031					
Vascular death in yr2+) | USA - Constant coeff	1.0898	-0.020914	1.45419				
Non fatal stroke in 1st year) | Stroke - Age coeff	0.0080	0.000085					
Non fatal stroke in 1st year) | Stroke - Constant coeff	-3.4503	-0.00589	0.424039				
Vascular death in 1st year) | Stroke - Age coeff	0.0887	0.0000049	-0.000332	0.000083			
Vascular death in 1st year) | Stroke - Constant coeff	-8.6181	-0.000336	0.02368	-0.00648	0.51523		
Any event in yrs2+ |Stroke - Age coeff	0.0421	0.000047					
Any event in yrs2+ |Stroke - Constant coeff	-5.8803	-0.003401	0.253293				
Non fatal stroke in yr2+) | Stroke & any event - Age coeff	-0.0578	0.000262					
Non fatal stroke in yr2+) | Stroke & any event- Constant coeff	3.8253	-0.018884	1.38745				

Congestive Heart Failure

The review of previous economic evaluations of diabetes prevention cost-effectiveness studies found that only a small number of models had included congestive heart failure as a separate outcome. Discussion with the stakeholder group identified that the UKPDS Outcomes model would be an appropriate risk model for congestive heart failure in type 2 diabetes patients. However, it was suggested that this would not be an appropriate risk equation for individuals with normal glucose tolerance or impaired glucose tolerance. The Framingham risk equation was suggested as an alternative. As described above, switching from the Framingham risk score to the UKPDS was not possible due to differences in covariate selection. The main limitation of this equation is that it is quite old, based on a non-UK population, and include diabetes as a discrete health state rather than on a continuous scale.

Congestive heart failure was included as a separate cardiovascular event because it was not included as an outcome of the QRISK2. The Framingham Heart Study has reported logistic regressions to estimate the 4 year probability of congestive heart failure for men and women (65). The equations included age, diabetes diagnosis,

BMI and systolic blood pressure to adjust risk based on individual characteristics. We used this risk equation to estimate the probability of congestive heart failure in the SPHR diabetes prevention model. Table 5.10.5 describes the covariates for the logit models to estimate the probability of congestive heart failure in men and women.

Table 5.10.5: Logistic regression coefficients to estimate the 4-year probability of congestive heart failure from the Framingham study

Variables	Units	Regression Coefficient	OR (95% CI)	P
Men				
Intercept		-9.2087		
Age	10 y	0.0412	1.51 (1.31-1.74)	<.001
Left ventricular hypertrophy	Yes/no	0.9026	2.47 (1.31-3.77)	<.001
Heart rate	10 bpm	0.0166	1.18 (1.08-1.29)	<.001
Systolic blood pressure	20 mm Hg	0.00804	1.17 (1.04-1.32)	0.007
Congenital heart disease	Yes/no	1.6079	4.99 (3.80-6.55)	<.001
Valve disease	Yes/no	0.9714	2.64 (1.89-3.69)	<.001
Diabetes	Yes/no	0.2244	1.25 (0.89-1.76)	0.2
Women				
Intercept		-10.7988		
Age	10 y	0.0503	1.65 (1.42-1.93)	<.001
left ventricular hypertrophy	Yes/no	1.3402	3.82 (2.50-5.83)	<.001
Heart rate	100 cL	0.0105	1.11 (1.01-1.23)	0.03
Systolic blood pressure	10 bpm	0.00337	1.07 (0.96-1.20)	0.24
congenital heart disease	20 mm Hg	1.5549	4.74 (3.49-6.42)	<.001
Valve disease	Yes/no	1.3929	4.03 (2.86-5.67)	<.001
Diabetes	Yes/no	1.3857	4.00 (2.78-5.74)	<.001
BMI	kg/m2	0.0578	1.06 (1.03-1.09)	<.001
Valve disease and diabetes	Yes/no	-0.986	0.37 (0.18-0.78)	0.009
*OR indicates odds ratio; CI, confidence interval; LVH, left ventricular hypertrophy; CHD, congenital heart disease; and BMI, body mass index. Predicted probability of heart failure can be calculated as: p = 1/(1+exp(-xbeta)), where xbeta = Intercept + Sum (of regression coefficient*value of risk factor)				

Many of the risk factors included in this risk equation were not simulated in the diabetes model, therefore they could not be included in the model to predict CHD. We adjusted the baseline odds of CHD to reflect the expected prevalence of these symptoms in a UK population.

The proportion of the UK population with left ventricular hypertrophy was assumed to be 5% in line with previous analyses of the Whitehall II cohort (66). The heart rate for men was assumed to be 63.0bpm and for women 65.6bpm based on data from previous Whitehall II cohort analyses (67). The prevalence of congenital heart disease was estimated from an epidemiology study in the North of England. The study reports the prevalence of congenital heart disease among live births which was used to estimate the adult prevalence (68). This may over-estimate the prevalence, because the life expectancy of births with congenital heart disease is reduced compared with the general population. However, given the low prevalence it is unlikely to impact on the results. The prevalence of valve disease was estimated from the Echocardiographic Heart of England Screening study (69).

Using the estimated population values we adjusted the intercept values to account for the population risk in men and women. This resulted in a risk equation with age, systolic blood pressure, diabetes (diabetes diagnosis or HbA1c>6.5), and BMI in women to describe the risk of congestive heart failure for the policy analysis model.

5.10.2 Microvascular Complications

The review of previous economic evaluations identified that the UKPDS data was commonly used to estimate the incidence of microvascular complications (59). This data has the advantage of being estimated from a UK diabetic population. Given that the events described in the UKPDS outcomes model are indicative of late-stage microvascular complications, we did not believe it was necessary to seek an alternative model that would be representative of an impaired glucose tolerance population.

We adopted a simple approach to modelling microvascular complications. We used both versions of the UKPDS Outcomes model to estimate the occurrence of major events relating to these complications, including renal failure, amputation, foot ulcer, and blindness (60). These have the greatest cost and utility impact compared with earlier stages of microvascular complications, so are more likely to have an impact on the SPHR diabetes prevention outcomes.

As a consequence, we assumed that microvascular complications only occur in individuals with HbA1c>48 mmol/mol (6.5%). Whilst some individuals with hyperglycaemia (HbA1c>42 mmol/mol [6.0%]) may be at risk of developing microvascular complications, it is unlikely that they will progress to renal failure, amputation or blindness before a diagnosis of diabetes. Importantly, we did not assume that only individuals who have a formal diagnosis of diabetes are at risk of these complications. This allows us to incorporate the costs of undetected diabetes into the simulation.

The UKPDS includes four statistical models to predict foot ulcers, amputation with no prior ulcer, amputation with prior ulcer and a second amputation (60). In order to simplify the simulation of neuropathy outcomes we consolidated the models for first amputation with and without prior ulcer into a single equation. The parametric survival models were used to generate estimates of the cumulative hazard, H, in the current (t) and previous period (t-1). From which the probability of organ damage, p(Event), being diagnosed was estimated.

$$p(Event) = 1 - \exp\left(-\left(H(t) - H(t-1)\right)\right) \quad (1.1)$$

The functional form for the microvascular models included exponential and Weibull.

Retinopathy

We used the UKPDS outcomes model v2 to estimate the incidence of blindness in individuals with HbA1c>48 mmol/mol (6.5%) (60). The exponential model assumes a baseline hazard λ, which can be calculated from the model coefficients, β, reported in Table 5.10.6 and the individual characteristics for $\boldsymbol{X}$. These parameters are sampled from a normal distribution defined by their standard errors.

$$\lambda = exp\left(\beta_0 + \boldsymbol{X\beta_k}\right)$$

Table 5.10.6: Parameters of the UKPDS2 Exponential Blindness survival model

	Mean coefficient	Standard error	Modified mean coefficient
Lambda	-11.607	0.759	-10.967
Age at diagnosis	0.047	0.009	0.047
HbA1c	0.171	0.032	0.171
Heart rate	0.080	0.039	
SBP	0.068	0.032	0.068
White Blood Count	0.052	0.019	
CHF History	0.841	0.287	0.841
IHD History	0.0610	0.208	0.061
SBP Systolic Blood Pressure; CHF Congestive Heart Failure; IHD Ischaemic Heart Disease			

The age at diagnosis coefficient was multiplied by age in the current year if the individual had not been diagnosed with diabetes, and by the age at diagnosis if the individual had received a diagnosis.

The expected values for the risk factors not included in the SPHR model (heart rate and white blood count) were taken from Figure 3 of the UKPDS publication in which these are described (60). Assuming these mean values, it was possible to modify the baseline risk without simulating heart rate and white blood cell count.

Neuropathy

We used the UKPDS outcomes model v2 to estimate the incidence of ulcer and amputation in individuals with HbA1c>48 mmol/mol (6.5%) (60). The parameters of the ulcer and first amputation models are reported in Table 5.10.7.

Table 5.10.7: Parameters of the UKPDS2 Exponential model for Ulcer, Weibull model for first amputation with no prior ulcer and exponential model for 1st amputation with prior ulcer

	Ulcer		1st Amputation no prior ulcer		1st Amputation prior ulcer		2nd Amputation	
	Logistic		Weibull		Exponential		Exponential	
	Mean	Standard error	Mean	Standard error	Mean	Standard error	Mean	Standard error
Lambda	-11.295	1.130	-14.844	1.205	-0.881	1.39	-3.455	0.565
Rho			2.067	0.193				
Age at diagnosis	0.043	0.014	0.023	0.011	-0.065	0.027		
Female	-0.962	0.255	-0.0445	0.189				
Atrial fibrillation			1.088	0.398				
BMI	0.053	0.019						
HbA1c	0.160	0.056	0.248	0.042			0.127	0.06
HDL			-0.059	0.032				
Heart rate			0.098	0.050				
MMALB			0.602	0.180				
PVD	0.968	0.258	1.010	0.189	1.769	0.449		
SBP			0.086	0.043				
WBC			0.040	0.017				
Stroke History			1.299	0.245				

The exponential model assumes a baseline hazard λ, which can be calculated from the model coefficients, β, reported in Table 5.10.7 and the individual characteristics for $\boldsymbol{X}$.

$$\lambda = exp\left(\beta_0 + \boldsymbol{X\beta}\right)$$

The Weibull model for amputation assumes a baseline hazard:

$$h(t) = \rho t^{\rho-1} \exp(\lambda)$$

where λ is also conditional on the coefficients and individual characteristics at time t. ρ is the shape parameter.

The logistic model for ulcer is described below.

$$\Pr(y = 1|\mathbf{X}) = \frac{\exp(\mathbf{X\beta})}{1 + \exp(\mathbf{X\beta})}$$

Where the probability of the individual having an ulcer is conditional on their covariates, X, and the model coefficients, β. The ulcer and amputation models include a number of covariates that were not included in the simulation. As such it was necessary to adjust the statistical models to account for these measures. We estimated a value for the missing covariates and added the value multiplied by the coefficient to the baseline hazard.

The expected values for the risk factors not included in the SPHR model (heart rate, white blood count, micro-/macroalbuminuria, peripheral vascular disease and atrial fibrillation) were taken from Figure 3 of the UKPDS publication in which these are described (60). In the ulcer model we assumed that 2% of the population had peripheral vascular disease.

The amputation risk model with a history of ulcer was not included in the simulation, but was used to estimate an additional log hazard ratio to append onto the amputation model without a history of ulcer. The log hazard was estimated for each model assuming the same values for other covariates. The difference in the log hazard between the two models was used to approximate the log hazard ratio for a history of ulcer in the amputation model (10.241). The final model specifications are reported in Table 5.10.8. Parameters were sampled from a normal distribution specified by their standard errors.

Table 5.10.8: Coefficients estimates for Ulcer and 1st Amputation

	**Ulcer**		**1st Amputation**		**2nd Amputation**	
	**Logistic**		**Weibull**		**Exponential**	
	**Mean**	**Standard error**	**Mean**	**Standard error**	**Mean**	**Standard error**
Lambda	-11.276	1.13	-13.954	1.205	-3.455	0.565
Rho			2.067	0.193		
Age at Diagnosis	0.043	0.014	0.023	0.011		
Female	-0.962	0.255	-0.445	0.189		
BMI	0.053	0.019				
HbA1c	0.160	0056	0.248	0.042	0.127	0.06
HDL			-0.059	0.032		
Stroke			1.299	0.245		
Foot Ulcer			10.241			

Nephropathy

We used the UKPDS outcomes model v1 to estimate the incidence of renal failure in individuals with HbA1c>48 mmol/mol (6.5%) (54). Early validation analyses identified that the UKPDS v2 model substantially overestimated the incidence of renal failure in the SPHR model. The Weibull model for renal failure assumes a baseline hazard:

$$h(t) = \rho t^{\rho-1} \exp(\lambda)$$

where λ is also conditional on the coefficients and individual characteristics at time t. ρ is the shape parameter. The parameters of the renal failure risk model are reported in Table 5.10.9. Regression coefficient parameters were sampled from a normal distribution specified by their standard errors.

Table 5.10.9: Parameters of the UKPDS2 Weibull renal failure survival model

	**Mean**	**Standard error**
Lambda	-10.016	0.939
Shape parameter	1.865	0.387
SBP	0.404	0.106
BLIND History	2.082	0.551

5.10.3 Cancer

The conceptual model identified breast cancer and colorectal cancer risk as being related to BMI. However, these outcomes were not frequently included in previous cost-effectiveness models for diabetes prevention. Discussion with stakeholders identified the EPIC Norfolk epidemiology cohort study as a key source of information about cancer risk in a UK population. Therefore, we searched publications from this cohort to identify studies reporting the incidence of these risks. In order to obtain the best quality evidence for the relationship between BMI and cancer risk we searched for a recent systematic review and meta-analysis using key terms 'Body Mass Index' and 'Cancer', filtering for meta-analysis studies.

Breast cancer

Incidence rates for breast cancer in the UK were estimated from the European Prospective Investigation of Cancer (EPIC) cohort. This is a large multi-centre cohort study looking at diet and cancer. In 2004 the UK incidence of breast cancer by menopausal status was reported in a paper from this study investigating the relationship between body size and breast cancer (70). The estimates of the breast cancer incidence in the UK are reported in Table 5.10.10, in the simulation we assume the risk of breast cancer starts from age 50.

Table 5.10.10: UK breast cancer incidence

	Number of Cases	Person Years	Mean BMI	Incidence Rate of per person-year	Standard error	Reference
UK pre-menopause	102	103114.6	24	0.00099	0.00009	(70)
UK post-menopause	238	84214.6	24	0.00283	0.00004	(70)

A large meta-analysis that included 221 prospective observational studies has reported relative risks of cancers per unit increase in BMI, including breast cancer by menopausal status (71). We included a risk adjustment in the model so that individuals with higher BMI have a higher probability of pre-and post-menopausal breast cancer (71). In the simulation we adjusted the probability of breast cancer according to the difference in the individual's BMI and the average BMI reported in the EPIC cohort. The relative risk and confidence intervals per 5mg/m^2 increase in BMI are reported in Table 5.10.11. The relative risk parameters were sampled from a log-normal distribution defined by standard error calculated from confidence intervals, then used to estimated model inputs.

Table 5.10.11: Relative risk of Breast cancer by BMI

	Mean Relative risk	2.5th Confidence Interval	97.5th Confidence Interval	Reference
UK pre-menopause	0.89	0.84	0.94	(71)
UK post-menopause	1.09	1.04	1.14	(71)

Colorectal cancer

Incidence rates for colorectal cancer in the UK were reported from the European Prospective Investigation of Cancer (EPIC) cohort. The UK incidence of colorectal cancer is reported by gender in a paper from this study investigating the relationship between body size and colon and rectal cancer (72). The estimates of the colorectal cancer incidence are reported in Table 5.10.12, in the simulation we assume the risk of colorectal cancer starts from age 50. Inclidence input parameters were sampled from a normal distribution in the PSA.

Table 5.10.12: UK colorectal cancer incidence

	Number of Cases	Person Years	Mean Age	Mean BMI	Incidence Rate of per person-year	Standard error	Reference
Male	125	118468	53.1	25.4	0.00106	0.0001	(72)
Female	145	277133	47.7	24.5	0.00052	0.0002	(72)

The risk of colorectal cancer has been linked to obesity. We included a risk adjustment in the model to reflect observations that the incidence of breast cancer is increased in individuals with higher BMI. A large meta-analysis that included 221 prospective observational studies has reported relative risks of BMI and cancers, including colon cancer by gender (71). We selected linear relative risk estimates estimated from pooled European and Australian populations. In the simulation we adjusted the incidence of colorectal cancer by adjusting the probability of colorectal cancer by the difference in the individual's BMI and the average BMI reported in the EPIC cohort. The relative risk and confidence intervals per 5mg/m^2 increase in BMI are reported in Table 5.10.13. The relative risk parameters were sampled from a log-normal distribution defined by standard error calculated form confidence intervals, then used to estimated model inputs.

Table 5.10.13: Relative risk of colon cancer by BMI

	Mean Relative risk	2.5th Confidence Interval	97.5th Confidence Interval	Reference
Male	1.21	1.18	1.24	(71)
Female	1.04	1.00	1.07	(71)

5.10.4 Osteoarthritis

Stakeholders suggested that diabetes and BMI should be included as independent risk factors for osteoarthritis. Osteoarthritis had not been included as a health state in previous cost-effectiveness models. The stakeholder group requested that BMI and diabetes be included as risk factors for osteoarthritis based on recent evidence (73). A search for studies using key words 'Diabetes', 'Osteoarthritis' and 'Cohort Studies' did not identify a UK based study with diabetes and body mass index included as independent covariates in the risk model. Therefore, the Italian study was used in the model.

A study from the Bruneck cohort, a longitudinal study of inhabitants of a town in Italy reported diabetes and BMI as independent risk factors for osteoarthritis (73).

The cohort may not be representative of a UK cohort. However, the individuals are from a European country, the study has a large sample size and has estimated the independent effects of BMI and diabetes on the risk of osteoarthritis. No UK based studies identified in our searches met these requirements. The data used to estimate the incidence of osteoarthritis in adults over the age of 45 is reported in Table 5.10.14. We did not identify any studies that described diabetes risk on a continuous scale. The incidence was sampled from a normal distribution in the PSA, and the hazard ratios from the log-normal distribution using the standard errors reported in the study.

Table 5.10.14: Incidence of osteoarthritis and estimated risk factors

	No. cases	Person years	Mean BMI	Incidence rate	Standard error	Reference
No diabetes	73	13835	24.8	0.0053	0.0006	(73)
	**Hazard ratio**	**2.5th**	**97.5th**			Reference
HR Diabetes	2.06	1.11	3.84			(73)
HR BMI	1.076	1.023	1.133			(73) Personal communication

5.10.5 Depression

Depression was not included as a health state in previous cost-effectiveness models for diabetes prevention. However, a member of the stakeholder group identified that a relationship between diabetes and depression was included in the CORE diabetes treatment model (74). Therefore, the references used in this model were used.

Depression was included as a health state in the model. However, the severity of depression was not modelled. Some individuals enter the simulation with depression at baseline according to individual responses in the Health Survey for England 2014 questionnaire. Depression is described in the simulation as a chronic state from which individuals do not completely remit. We did not estimate the effect of depression on the longitudinal changes for BMI, glycaemia, SBP and cholesterol. As a consequence, it was not possible to relate the impact of depression to the incidence of diabetes and cardiovascular risk.

In the simulation, individuals can develop depression in any cycle of the model. The baseline incidence of depression among all individuals without a history of depression was estimated from a study examining the bidirectional association between depressive symptoms and type 2 diabetes (75). Although the study was not from a UK population, the US cohort included ethnically diverse men and women aged 45 to 84 years. We assumed that diagnosis of diabetes and/or CVD increased the incidence of depression in individuals who do not have depression at baseline. We identified a method for inflating risk of depression for individuals with diabetes from the US cohort study described above (75). The risk of depression in individuals who have had a stroke was also inflated according to a US cohort study (76). Odds of depression and odds ratios for inflated risk of depression due to diabetes or stroke are presented in Table 5.10.15. In the PSA depression incidence was sampled from a normal distribution and the log-odds sampled from a log-normal distribution.

Table 5.10.15: Baseline incidence of depression

	**Mean**	**Standard error**	
Depression cases in NGT	336		
Person years	9139		
Odds of depression	0.0382	0.002	
Log odds of depression	-3.266		
**Inflated risk for Diabetes**			
	**Mean**	**2.5th CI**	**97.5th CI**
Odds ratio of diabetes	1.52	1.09	2.12
Log odds ratio of diabetes	0.419		
**Inflate risk of stroke**			
Odds ratio of stroke	6.3	1.7	23.2
Log odds ratio stroke	1.8406		
NGT Normal Glucose Tolerance			

5.11 Dementia

The risk dementia diagnosis is estimated from risk models estimated from the THIN database (77). The THIN dementia risk score uses data from The Health Improvement Network (THIN) database from across the UK. Routinely collected data was used to predict 5-year risk of recorded diagnosis of Dementia for those aged 60-79 and 80+. The sample size is large and the risk scores are representative of the United Kingdom and diagnosis practices between 2000-2011. The disadvantage of these risk scores are the relatively short follow-up of

patients, the low predictive power of the older risk score, and narrow scope to predict dementia diagnosis but not dementia onset.

The parameters for the THIN 60-79 year old and 80-99 risk models are reported in Table 5.11.1. These parameters are sampled from a normal distribution defined by their standard errors.

Table 5.11.1: THIN dementia risk models

THIN 60-79 Risk Score			THIN 80-99 Risk Score		
**Parameter label**	**mean**	**Standard error**	**Parameter label**	**mean**	**Standard error**
Baseline hazard	0.9969		Baseline hazard	-0.9277	
Age	0.2092	0.0047	Age	0.055	0.0041
Age^2	-0.0034	0.0003	Age^2	-0.005	0.0010
Female	0.1285	0.0278	Female	0.16	0.0286
Calendar Year	0.0448	0.0050	Calendar Year	0.074	0.0056
Townsend quintile 2	0.0134	0.0390	BMI	-0.05	0.0066
Townsend quintile 3	0.1179	0.0392	Anti-hypertensives	-0.249	0.0265
Townsend quintile 4	0.2018	0.0402	Systolic Blood Pressure	-0.006	0.0010
Townsend quintile 5	0.2255	0.0447	Lipid ratio	0.042	0.0495
BMI	-0.0616	0.0038	Past Smoker	-0.178	0.0281
BMI^2	0.0025	0.0003	Smoker	-0.134	0.0485
Anti-hypertensives	-0.1320	0.0296	Alcohol Problems	0.256	0.1352
Past Smoker	-0.0679	0.0301	Diabetes	0.183	0.0413
Smoker	-0.0866	0.0415	Stroke	0.242	0.0332
Alcohol problems	0.4435	0.0799	Atrial Fibrillation	0.057	0.0383
Diabetes	0.2867	0.0417	Depression	0.4	0.0332
Depression	0.8336	0.0325	Anxiety	0.136	0.0520
Stroke	0.5772	0.0394	NSAIDs use	-0.157	0.0408
Atrial Fibrillation	0.2207	0.0514	Aspirin use	0.092	0.0281
Aspirin use	0.2528	0.0326			

For the SPHR prevention model the 5-year Dementia risk was transformed into 1 year individual probabilities. The Dementia risk scores include fixed and time-varying patient characteristics. As a consequence, it is not possible to use standard methods of transforming probabilities over different time-horizons (78). We used a simple calibration technique to modify the baseline hazard to reflect simulated changes in the populations risk profile over 5 years. We calibrate the simulated 5 year incidence of dementia against the predicted incidence for each age group in the THIN database (77).

For each risk model we simulated 20,000 randomly sampled patients aged 60-79 and 80-95 in 50 model runs. For each sample we repeated simulations multiple times, in each simulation the baseline hazard was adjusted until the incidence of Dementia equalled the THIN risk score prediction based on baseline characteristics. The baseline hazard adjustment was estimated by averaging the adjustments needed for each of the 20 simulation runs to match the THIN prediction. The calibration was designed to calibrate to the predicted incidence, rather than the reported incidence from the THIN dataset to account for any differences in the baseline characteristics of the THIN data and HSE sample. For example, the mean age for the development cohort of the 60-79 model were 65.6, whereas the mean simulated ages was 70. Age is an important predictor of Dementia incidence so it is important to adjust for differences in baseline age between the observed and simulated data.

The THIN database reports a crude incidence of 1.88 per 1,000 persons years for 60-79 and 16.53 per 1,000 person years for ages 80-99 (column 2 Table 5.11.2). The 5 year risk score for individuals sampled from the Health Survey for England reports a 5 year incidence of 0.00255 and 0.01523 (column 3 Table 5.11.2). Using the adjustment factor identified by calibration (column 5 Table 5.11.2), we simulated a 5 year incidence of 0.00255 and 0.01510 (column 4 Table 5.11.2). The adjustment factor is applied to the baseline hazards of the THIN dementia risk scores. The adjustment factor parameters are sampled from a normal distribution defined by their standard errors.

Table 5.11.2: Dementia incidence rates used to derive the adjustment factor

	5 year crude incidence from QResearch	5 year risk score (SD) for Health Survey for England population	5 year simulated incidence (SD) with adjustment factor	Adjustment factor (SE)
THIN risk model 60-79	0.00188	0.00255	0.00255	7.628 (0.104)
THIN risk model 80+	0.01653	0.01523	0.01510	4.557 (0.020)

5.11.1 Dementia Diagnosis

A Swedish registry reporting MMSE scores at dementia diagnosis was identified (79). This study reported a mean MMSE score at diagnosis of 21.2 (SD 5.2). This data was used to generate MMSE scores at diagnosis in the model because it reflects cognitive function in a cohort diagnosed in routine care. In order to generate heterogeneity in cognitive function at diagnosis a Gamma distribution was fitted to this mean and standard distribution and patients MMSE score at diagnosed was sampled from this distribution. Sampled value outside the limits of the MMSE score, assuming a maximum score at diagnosis of 25, were re-sampled from a uniform distribution within these limits. The resulting distribution was compared against summary data from CFAS for an incident cohort to validate the simulated MMSE scores against this dataset (80). In the PSA the mean MMSE score at diagnosis was sampled from the normal distribution to update the alpha and beta coefficients for the Gamma distribution.

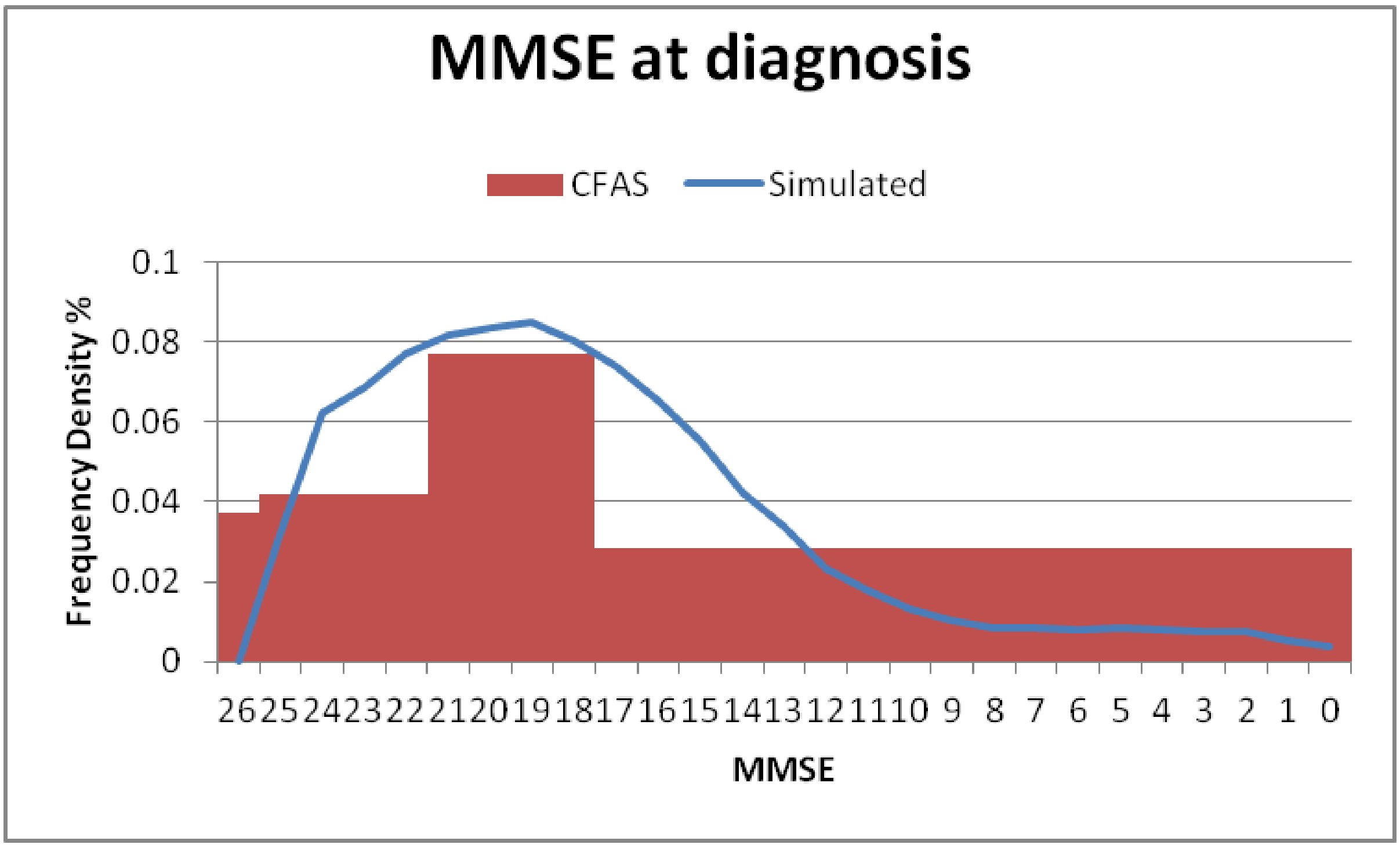

5.11.2 Disease Progression

Dementia disease progression was characterized by a deterioration in MMSE score. This relatively simple characterization of the disease is sufficient to capture major cost escalations and quality of life deterioration. A more complex structure that explicitly models institutionalisation was considered (81). However, it was concluded that the data on institutionalisation was out of date. Therefore, the modelling structure was aligned to the cost estimates for dementia (82).

Changes in MMSE score over time were estimated using data from a recent cost-effectiveness model for Donepezil (83). Although the data are from Canada and are relatively old, the sample size is large and model specification allows for detailed characterization of MMSE decline. The regression allows rate of change in MMSE to be conditional on age at baseline and includes splines to describe a different rate of change at different levels of MMSE. For example, the decline in MMSE slows as the score declines below 9.

5.12 Dental Caries

Dental caries are assumed to affect both children and adults in the model dependent on sugar consumption. Dental caries are simulated using separate models for those under 16 year olds and those 16 years and older.

5.12.1 Children Dental Model

Whether a child under 16 years old has had treatment for dental decay is simulated using odds ratios from an analysis of survey data relating to sugar intake and dental health in Scotland (84). The data captures individuals from ages 3 to 17 and is applied to all individuals under 16 years old in the model. The model predicts probability of the individuals receiving dental treatment for decay based on non-milk-extrinsic sugar (NMES) consumption in tertiles (Table 5.12.1).

The probability of receiving treatment is adjusted for age using data from the Childrens Dental Health survey (2013) and from NDNS years 9 to 11 (19, 85). This probability for each individual is estimated by applying the log odds ratio of receiving dental treatment according to NMES consumption tertile to the log odds of receiving dental treatment when consuming within tertile 1. This log odds of dental caries in tertile 1 is calculated for ages 5, 8, 12, and 15 years using the odds ratios, the overall probability of have dental decay by each age group taken from Childrens Dental Health survey (2013) and the proportions that consume sugar within the tertiles by each age group described in logistic analysis within the National Diet and Nutrition Survey (2018) (Table 5.12.2 and Table 5.12.3), with the equations below. The probability simulated is compared to a random number to determine if they have had treatment for dental decay.

The log odds of having dental caries is estimated using the log odds of having dental caries in consumption tertile 1 and adjusted using the log odds ratio of having dental caries for consumption tertile 2 and 3 (Table 5.12.1) such that,

$$Estimated\ Log\ Odds\ of\ Dental\ Caries = Log\ Odds\ of\ Dental\ Caries\ of\ tertile\ 1 + \ln(Odds\ ratio)$$

Where, for each age group,

$$\begin{aligned} Log\ Odds\ of\ Dental\ Caries\ of\ tertile\ 1 \\ &= Base\ Log\ Odds\ of\ Dental\ Caries\ - Proportion\ in\ consumption\ tertile\ 2 \\ &* Log\ Odds\ of\ dental\ caries\ for\ consumption\ tertile\ 2 \\ &- Proportion\ in\ consumption\ tertile\ 3 \\ &* Log\ Odds\ \ of\ dental\ caries\ for\ consumption\ tertile\ 3 \end{aligned}$$

and

$$Base\ Log\ Odds\ of\ Dental\ Caries = \ln\left(\frac{Probability\ of\ Dental\ Caries}{1 - Probability\ of\ Dental\ Caries}\right)$$

The log odds are converted back to a probability using,

$$Probability = \frac{\exp(Estimated\ Log\ Odds\ of\ Dental\ Caries)}{1 + \exp(Estimated\ Log\ Odds\ of\ Dental\ Caries)}$$

Samples of the odds ratios are taken using a normal distribution and the standard errors calculated using the 95% confidence intervals (Table 5.12.1). The overall proportions of children with dental decay by age group also sampled with a normal distribution (Table 5.12.2). Samples of the proportions of sugar consumption in each tertile for tertile 2 and 3 assuming a standard error obtained from the binomial distribution (78) (Table 5.12.3). The remaining proportion is calculated by taking the proportion in consumption tertile 2 and 3 from 1.

Table 5.12.1: Adjusted odds ratios of having dental caries conditional on non-milk-extrinsic sugar consumption (83)

		**Adjusted Odds ratio**	**95% CI**	**Calculated SE**	**Distribution**
**NMES Tertile**	**Grams/day**				
Tertile 1	<=80.2	1			
Tertile 2	80.2–102.6	1.14	0.97,1.59	0.18	Normal
Tertile 3	>= 102.6	1.65	1.68,2.86	0.29	Normal

Table 5.12.2: Proportion of children with dental caries by age group from Childrens Dental Health Survey (2013) (85).

**CDHS**		**Proportion (%)**	**SE**	**Distribution**
**Type of Teeth**	**Age**			
Primary	5	0.9	0.1	Normal
Permanent	8	13.3	1.2	Normal
Permanent	12	33.5	2.2	Normal
Permanent	15	46.0	2.7	Normal

Table 5.12.3: Proportion of children within the NDNS consuming within each tertile of NMES consumption that was previously defined (84), by age group (19).

**AGE**	**NMES - Tertile**	**Grams/day**	**N**	**%**	**Calculated SE**	**Distribution**
5	T1	<=80.2	453	94%		
	T2	80.2–102.6	16	3%	0.01%	Normal
	T3	>= 102.6	11	2%	0.00%	Normal
8	T1	<=80.2	270	87%		
	T2	80.2–102.6	27	9%	0.03%	Normal
	T3	>= 102.6	15	5%	0.01%	Normal
12	T1	<=80.2	297	80%		
	T2	80.2–102.6	46	12%	0.03%	Normal
	T3	>= 102.6	30	8%	0.02%	Normal
15	T1	<=80.2	268	78%		
	T2	80.2–102.6	31	9%	0.02%	Normal
	T3	>= 102.6	45	13%	0.03%	Normal

5.12.2 Adult Dental Model

On entrance into the model, the adults within the model population are given the average number of decayed, missing or filled teeth (DMFT) for their age group as found in the Adult Dental Health Survey (2009) (86). The survey suggests 1% of those with reported number of DMFT have no decay. During the simulation, when individuals transition to the adult dental model at age 16, if they have not received dental treatment during childhood, they are assumed to have no decayed teeth and a DMFT of 0. If they were simulated to have received treatment for decay, they are assumed to have the average number of DMFT for those with decay, 6.51, as reported in the adult dental health survey for 16- to 25-year-olds. This mean value of DMFT is sampled assuming a normal distribution and a standard error of 0.16. Table 5.12.4 reports the mean DMFT and standard error by age group. These averages are sampled from a normal distribution using the mean and standard error.

Table 5.12.4: Number of decayed, missing or filled teeth by age group in the Adult Dental Health Survey

Age group	Mean	SE
16 to 24	6.3	0.16
25 to 34	8.7	0.19
35 to 44	12.5	0.17
45 to 54	17.1	0.17
55 to 64	20.1	0.16
65 to 74	21.6	0.19

DMFT is modelled to change dependent on sugar consumption by 0.1 per 10g of sugar across 11 years, linearly estimated in analysis of survey data from Finland (87). DMFT is increasing with time, but at a lower rate for individuals who consume less sugar. While there is evidence that dental caries can be reversed during the early stages, this is not considered within the simulation. The coefficient between consumption of sugar (grams per day) and DMFT is sampled assuming a normal distribution with the standard error of 0.028 calculated using the confidence intervals reported in the analysis (87). DMFT increases incrementally each year cycle and costs are applied to this change in DMFT. Figure 5.12.1 demonstrated how a temporary change in sugar consumption (either increase or decrease of 60g per day from average consumption or no change) that declines back to average consumption linearly over 5 years, results in reduced decay after 10 years.

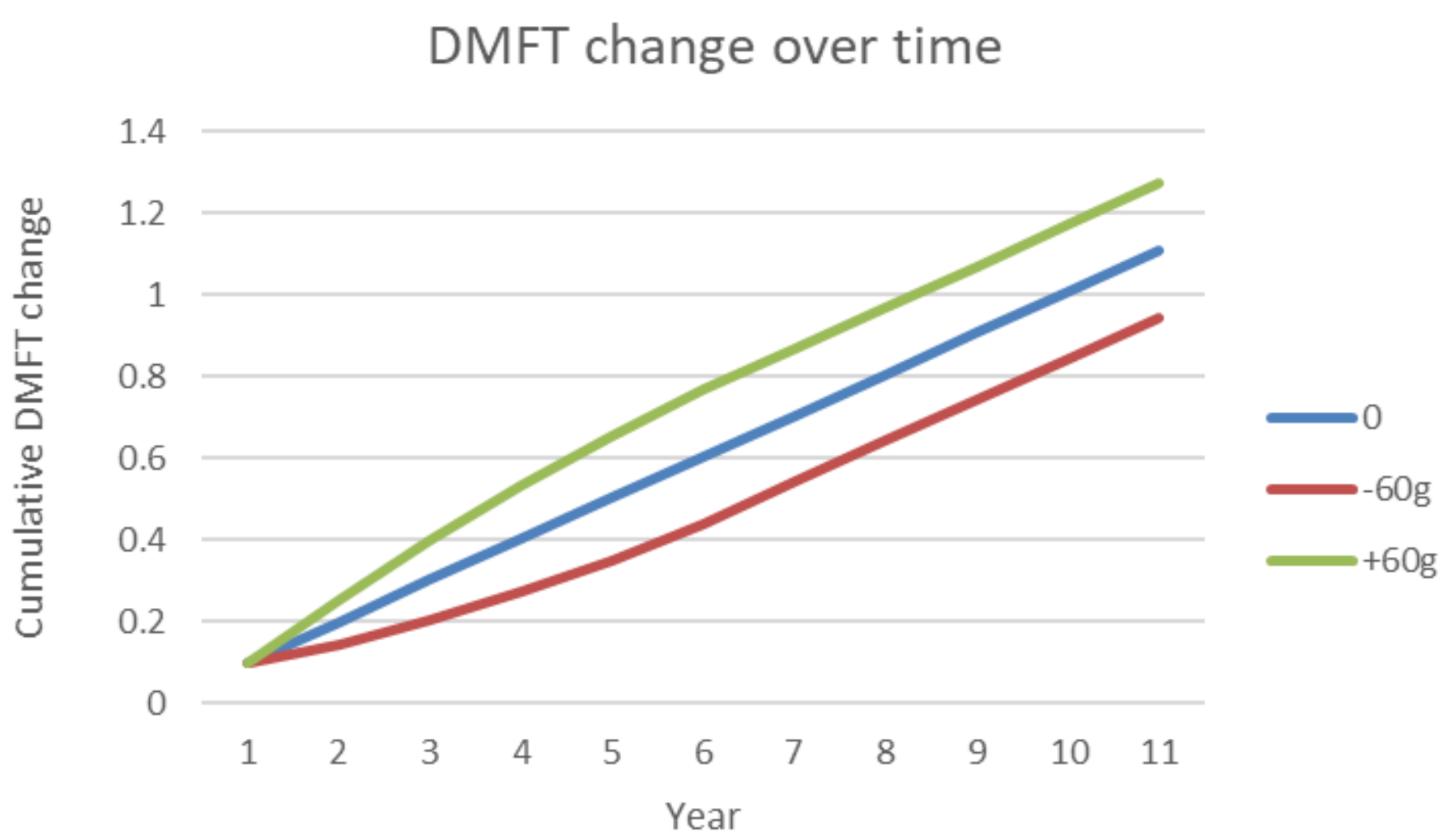

Figure 5.12.1: Change in decayed, missing, filled teeth over 11 years, adjusted for a temporary change in sugar consumption (no change, 60g reduction, or 60g increase in total sugar consumed per day).

5.13 Mortality

5.13.1 Cardiovascular Mortality

Cardiovascular mortality is included as an event within the QRISK2 (12) and the probability of subsequent cardiovascular events obtained from an HTA assessing statins (18), as described in the Cardiovascular disease section above.

5.13.2 Cancer Mortality

Cancer mortality rates were obtained from the Office of National statistics (88). The ONS report one and five year net survival rates for various cancer types, by age group and gender. Net survival was an estimate of the probability of survival from the cancer alone. It can be interpreted as the survival of cancer patients after taking into account the background mortality that the patients would have experienced if they had not had cancer.

The age-adjusted 5-year survival rate for breast cancer and colorectal cancer were used to estimate an annual risk of mortality assuming a constant rate of mortality. We assume that the mortality rate does not increase due to cancer beyond 5 years after cancer diagnosis. The five year survival rate for breast cancer is 84.3%, which translated into a 3.37% annual probability of death from breast cancer. The five year survival rate for persons with colorectal cancer is 55.3%, which translated into a 11.16% annual probability of death from colorectal cancer.

5.13.3 Other cause Mortality (including diabetes and Dementia risk)

Other cause mortality describes the risk of death from any cause except CVD, and cancer. All-cause mortality rates by age and sex were extracted from the 2018-2020 Office of National Statistics life tables (89, 90). The mortality statistics report the number of deaths by ICD codes for 5-year age groups. We subtracted the number of cardiovascular disease, diabetes, dementia, breast and colorectal cancer related deaths from the all-cause mortality total to estimate other cause mortality rates by age and sex (Table 5.13.1: All cause and derived other cause mortality from the Office of National statistics). Life table statistics were not sampled in the PSA.

Table 5.13.1: All cause and derived other cause mortality from the Office of National statistics

Age (years)	All cause	All cause	Other cause	Other cause	Age (years)	All cause	All cause	Other cause	Other cause
	Men	Women	Men	Women		Men	Women	Men	Women
**0**	0.0042	0.0035	0.0042	0.0035					
**1**	0.0002	0.0002	0.0002	0.0002	**51**	0.0037	0.0024	0.0028	0.0018
**2**	0.0001	0.0001	0.0001	0.0001	**52**	0.0039	0.0025	0.0029	0.0019
**3**	0.0001	0.0001	0.0001	0.0001	**53**	0.0043	0.0027	0.0032	0.0020
**4**	0.0001	0.0001	0.0001	0.0001	**54**	0.0046	0.0028	0.0034	0.0021
**5**	0.0001	0.0001	0.0001	0.0001	**55**	0.0049	0.0032	0.0036	0.0023
**6**	0.0001	0.0001	0.0001	0.0001	**56**	0.0054	0.0035	0.0039	0.0026
**7**	0.0001	0.0001	0.0001	0.0000	**57**	0.0059	0.0038	0.0043	0.0028
**8**	0.0001	0.0001	0.0001	0.0000	**58**	0.0065	0.0042	0.0047	0.0031
**9**	0.0001	0.0001	0.0001	0.0001	**59**	0.0070	0.0045	0.0051	0.0033
**10**	0.0001	0.0001	0.0001	0.0001	**60**	0.0077	0.0050	0.0056	0.0038
**11**	0.0001	0.0001	0.0001	0.0001	**61**	0.0084	0.0054	0.0061	0.0041
**12**	0.0001	0.0001	0.0001	0.0001	**62**	0.0093	0.0062	0.0068	0.0047
**13**	0.0001	0.0001	0.0001	0.0001	**63**	0.0102	0.0066	0.0074	0.0050
**14**	0.0001	0.0001	0.0001	0.0001	**64**	0.0110	0.0071	0.0079	0.0053
**15**	0.0002	0.0001	0.0002	0.0001	**65**	0.0122	0.0078	0.0089	0.0060
**16**	0.0002	0.0001	0.0002	0.0001	**66**	0.0135	0.0085	0.0098	0.0065
**17**	0.0003	0.0002	0.0003	0.0002	**67**	0.0145	0.0092	0.0105	0.0070
**18**	0.0004	0.0002	0.0004	0.0002	**68**	0.0160	0.0103	0.0117	0.0079
**19**	0.0004	0.0002	0.0004	0.0002	**69**	0.0176	0.0110	0.0128	0.0084
**20**	0.0005	0.0002	0.0005	0.0002	**70**	0.0188	0.0124	0.0137	0.0094
**21**	0.0005	0.0002	0.0005	0.0002	**71**	0.0203	0.0132	0.0148	0.0100
**22**	0.0005	0.0002	0.0004	0.0002	**72**	0.0222	0.0150	0.0162	0.0113

**23**	0.0005	0.0002	0.0005	0.0002	**73**	0.0253	0.0168	0.0185	0.0127
**24**	0.0005	0.0002	0.0005	0.0002	**74**	0.0279	0.0191	0.0204	0.0144
**25**	0.0005	0.0003	0.0005	0.0002	**75**	0.0315	0.0210	0.0229	0.0152
**26**	0.0006	0.0003	0.0005	0.0002	**76**	0.0350	0.0237	0.0255	0.0172
**27**	0.0006	0.0003	0.0006	0.0003	**77**	0.0393	0.0272	0.0286	0.0197
**28**	0.0006	0.0003	0.0006	0.0003	**78**	0.0442	0.0305	0.0322	0.0221
**29**	0.0007	0.0003	0.0006	0.0003	**79**	0.0491	0.0349	0.0357	0.0253
**30**	0.0007	0.0004	0.0007	0.0003	**80**	0.0550	0.0387	0.0390	0.0269
**31**	0.0008	0.0004	0.0007	0.0003	**81**	0.0610	0.0438	0.0433	0.0304
**32**	0.0008	0.0004	0.0007	0.0004	**82**	0.0680	0.0492	0.0482	0.0341
**33**	0.0009	0.0005	0.0008	0.0004	**83**	0.0759	0.0560	0.0539	0.0389
**34**	0.0009	0.0005	0.0008	0.0005	**84**	0.0858	0.0638	0.0609	0.0443
**35**	0.0010	0.0006	0.0009	0.0004	**85**	0.0963	0.0726	0.0669	0.0484
**36**	0.0010	0.0006	0.0009	0.0005	**86**	0.1091	0.0832	0.0758	0.0554
**37**	0.0013	0.0007	0.0011	0.0006	**87**	0.1216	0.0945	0.0845	0.0630
**38**	0.0012	0.0008	0.0011	0.0006	**88**	0.1365	0.1066	0.0948	0.0710
**39**	0.0014	0.0008	0.0012	0.0006	**89**	0.1532	0.1200	0.1064	0.0800
**40**	0.0015	0.0008	0.0012	0.0006	**90**	0.1621	0.1347	0.1155	0.0900
**41**	0.0016	0.0009	0.0013	0.0007	**91**	0.1816	0.1517	0.1294	0.1014
**42**	0.0017	0.0011	0.0014	0.0008	**92**	0.1986	0.1696	0.1416	0.1133
**43**	0.0019	0.0011	0.0015	0.0009	**93**	0.2224	0.1882	0.1585	0.1258
**44**	0.0021	0.0013	0.0017	0.0010	**94**	0.2442	0.2059	0.1740	0.1376
**45**	0.0023	0.0014	0.0018	0.0011	**95**	0.2696	0.2282	0.1921	0.1525
**46**	0.0024	0.0015	0.0019	0.0011	**96**	0.2925	0.2517	0.2084	0.1682
**47**	0.0026	0.0017	0.0021	0.0012	**97**	0.3142	0.2771	0.2239	0.1852
**48**	0.0028	0.0019	0.0022	0.0014	**98**	0.3352	0.2985	0.2389	0.1994
**49**	0.0031	0.0020	0.0025	0.0015	**99**	0.3754	0.3193	0.2675	0.2134
**50**	0.0034	0.0022	0.0026	0.0016	**100**	0.3974	0.3488	0.2832	0.2331

The rate of other cause mortality by age and sex was treated as the baseline hazard. Following input from stakeholders, an increased risk of mortality was assigned to individuals with diabetes using data from a published meta-analysis (91). This study used data from 820,900 people from 97 prospective studies to calculate hazard ratios for cause-specific death, according to baseline diabetes status (91). Cause of death was separated into vascular disease, cancer and other cause mortality. From this study we estimated that individuals with a diagnosis of diabetes have a fixed increased risk of other cause mortality (Hazard ratio 1.8 (95% CI 1.71-1.9)). The estimates reported in the meta-analysis include increased risk of death from renal disease, therefore mortality from renal disease was not simulated separately to avoid double counting of benefits. In the PSA the HR for diabetes mortality was sample from a log-normal distribution.

Mortality risk increases with the onset of dementia. As a consequence, all cause mortality was inflated after diagnosis of dementia. The hazard ratio of mortality was estimated from analysis of two United States cohorts (92). Participants were recruited to the studies without known dementia at baseline and received annual clinical evaluation and brain donation at death. The analysis included 2566 persons over 8 years and found a hazard ratio of death with all dementia of 4.54 (CI 3.54-5.83) for ages 75-84 and 2.77 (CI 2.37-3.23) for ages 85 and older (92). These hazard ratios were applied in the model to all cause mortality to describe mortality at younger ages 60-84 and older 85+ ages. In the PSA the HR were sample from a log-normal distribution.

Mortality rates for individuals with diabetes and undiagnosed diabetes are also at increased risk of mortality, which is applied to all cause mortality. Given the correlation between risk factors of diabetes and dementia, and the high prevalence of multi-comorbidities in later life, it is necessary to adjust mortality risk for individuals with both dementia and diabetes. We believe that applying both mortality hazard ratios would over-estimate the mortality burden in these patients. In the model indiviudals with Dementia and Type 2 diabetes have the higher hazard ratio for Dementia applied.

5.14 Direct Health Care Costs

At any given time period of the model individuals can have multiple health complications that incur direct healthcare costs. Some of the health states are mutually exclusive; however an individual can accrue multiple complications within the model. Each health state is associated with an average cost, which is accrued by all individuals for every time period for which the state is indicated. Resource use for each comorbidity is added together and no savings are assumed to be made from the use of the same resources for two or more comorbidities for an individual.

In some instances we have adopted costs and prices from old sources, and in other instances updated drug and healthcare costs to the most recent source available We have inflated or deflated all prices and costs to 2022/23 prices using inflation indices reported in the Personal Social Services Research Unit (PSSRU) (93). This documents health related inflation up to 2023/24 prices. The retail price index was used to inflate costs to 2022/23 prices.

Primary care and community care costs were sought from the Personal Social Services Research Unit (PSSRU) (93), and secondary care costs from UK reference costs (94). Drug costs were obtained from the British National Formulary (95). In most instances costs for long term health outcomes were sought from recent Health Technology Appraisals as this was thought to be the best source of evidence for costs and resource use by disease area in the UK. If an HTA appraisal was not identified, searches for good quality cost-effectiveness analyses for the relevant disease area were conducted to identify the appropriate UK costs.

5.14.1 GP attendance

The costs of each visit to a General Practitioner were estimated at £41 from the Personal Social Services Research Unit (PSSRU), assuming 9.22 minute appointments (93).

Diabetes diagnosis incurred a cost of £17.65 (2022 prices) in line with costs used for a previous evaluation of a Diabetes Prevention Programme (49).

Guidelines for hypertension recommend that hypertension be confirmed with ambulatory blood pressure monitoring (ABPM) (56). The cost of ABPM assessment is included in the cost of diagnosis (£49.32) (96), however, we assume that the test does not alter the initial diagnosis.

The cost of identifying individuals to receive statins is assumed to be negligible because cases are detected using existing cardiovascular risk programmes used by the GP.

5.14.2 Diabetes Medication

We were advised by stakeholders to model a simplified diabetes treatment pathway. It was recommended that a single annual cost of prescriptions be applied to all patients diagnosed with diabetes. Initially we explored this as an option but concluded that the timing of more costly treatments for type 2 diabetes is important because treatment costs will be discounted. The model assesses interventions that lower HbA1c and so have the potential to impact on the level of treatment required.

We decided to implement a three stage treatment regimen as a trade-off between model simplicity and capturing key cost differences between the interventions. At diagnosis all patients are prescribed low cost treatments, such

as Metformin and Sulfonylurea. We chose Metformin, 500mg/day to describe the average cost of these medications. If HbA1c increases above a threshold the individual is prescribed the more expensive Gliptins in addition to Metformin. The individual continues to receive Metformin plus Gliptins for a period of time until they require insulin.

Metformin Monotherapy

Cost estimates from the British National Formulary indicate that the cost of Metformin is approximately £17.50 per annum, using a combination of standard and modified release (95).

Other resource use costs and resource utilisation assumptions for diabetics receiving Metformin monotherapy are detailed in Table 5.14.1. Drug costs was assumed to not vary, while the other costs were assumed to have a standard error equal to 1/10th their mean.

Table 5.14.1: Drug costs and resource utilisation costs for low cost diabetes monotherapy

Resource	Assumption for costs	Source	Unit Cost 2022 prices	Annual utilisation	SE	Distribution	Cost per year
Metformin	500mg *bid* standard (85% of patients) or modified release (15%) tablets	(95)	0.024	730		CONSTANT	17.50
Nurse at GP	Advanced GP Nurse per hour for 15 minute appointment	(93)	50.81	0.25	5.08	GAMMA	12.70
GP Nurse	Band 4 – Qualified Nurse at GP practice per hour for 10 minute appointment	(93)	38.64	0.167	3.86	GAMMA	6.44
Urine sample	Clinical Biochemistry	(94)	1.55	1	0.16	GAMMA	1.55
Eye screening	Non-Consultant Lead Ophthalmology	(94)	106.42	1		CONSTANT	106.42
HbA1c	Haematology	(94)	2.96	1	0.30	GAMMA	2.96
Lipids	Clinical Biochemistry	(94)	1.55	1	0.16	GAMMA	1.55
Liver function	Clinical Biochemistry	(94)	1.55	1	0.16	GAMMA	1.55
B12	Clinical Biochemistry	(94)	1.55	1	0.16	GAMMA	1.55
			Total annual cost				152.22

The cost of diabetes in the year after diagnosis is assumed to be greater than subsequent years because the individual will receive more contact time whilst their diabetes is being controlled. The additional costs of diabetes in the year after diagnosis are reported in Table 5.14.2.

Table 5.14.2: Drug costs and resource utilisation costs for the first year after diabetes diagnosis

Resource	Assumption for costs	Source	Unit Cost 2022 prices	SE	Distribution	Annual utilisation	Cost per year
Nurse at GP	Advanced GP Nurse per hour for 15 minute appointment	(93)	50.81	5.08	GAMMA	0.5	25.41
GP Nurse	Band 4 – Qualified Nurse at GP practice per hour for 10 minute appointment	(93)	38.64	3.86	GAMMA	0.33	12.88
Urine sample	Clinical Biochemistry	(94)	1.55	0.16	GAMMA	2	3.10
HbA1c	Haematology	(94)	2.96	0.30	GAMMA	2	5.92
Lipids	Clinical Biochemistry	(94)	1.55	0.16	GAMMA	2	3.10
Liver function	Clinical Biochemistry	(94)	1.55	0.16	GAMMA	2	3.10
B12	Clinical Biochemistry	(94)	1.55	0.16	GAMMA	2	3.10
Smoking Cessation	Nicotine replacement therapy (Assumed 20% smoking prevalence and 50% uptake of smoking cessation services)	(93)	139.97	14.00	GAMMA	0.1	14.00
					Total annual cost		70.72

Metformin plus Gliptins

Simulated individuals experience an annual increase in HbA1c. Gillett et al. (2012) assume that individuals switch to dual treatment if HbA1c increases above 7.4% (97). Within the model, the individual is switched to a dual treatment in the first annual cycle in which HbA1c exceeds 7.4%. For costing purposes the second drug to be added to Metformin was Sitagliptin, which is reported in the British National Formulary to cost £416.40 per year (95). Belsey et al. (2009) report that 48% of patients used monitoring strips at a mean weekly consumption of 3.3 (98). Table 5.14.3 reports the other resource use costs and utilisation assumptions for diabetics receiving Metformin plus Gliptins.

Table 5.14.3: Drug costs and resource utilisation costs for Metformin and Gliptins

Resource	Assumption for costs	Source	Unit Cost 2022 prices	SE	Distribution	Annual utilisation	Cost per year
Sitagliptin	100mg per day from packet of 28-tabs of 100mg	(95)	1.14		CONSTANT	365	416.4
Metformin	500mg *bid* standard (85% of patients) or modified release (15%) tablets	(95)	0.024		CONSTANT	730	17.50
Self-monitoring strips	50 strip pack from National Diagnostic Products, 11p per strip	(95)	0.11		CONSTANT	82.2	8.67
Nurse at GP	Advanced GP Nurse per hour for 15 minute appointment	(93)	50.81	5.08	GAMMA	0.25	12.70
GP Nurse	Band 4 – Qualified Nurse at GP practice per hour for 10 minute appointment	(93)	38.64	3.86	GAMMA	0.167	6.44
Urine sample	Clinical Biochemistry	(94)	1.55	0.16	GAMMA	1	1.55
Eye screening	Non-Consultant Lead Ophthalmology	(94)	106.42	1	CONSTANT	1	106.42
HbA1c	Haematology	(94)	2.96	0.30	GAMMA	1	2.96
Lipids	Clinical Biochemistry	(94)	1.55	0.16	GAMMA	1	1.55
Liver function	Clinical Biochemistry	(94)	1.55	0.16	GAMMA	1	1.55
B12	Clinical Biochemistry	(94)	1.55	0.16	GAMMA	1	1.55
					Total annual cost		£577.29

Insulin plus Oral Anti-diabetics

The second major treatment change is assumed to be initiation of insulin. Gillett et al. (2012) assumed that individuals switch to insulin if HbA1c increases above 8.5% (97). Within the model the individual is switched to insulin in the first annual cycle at which HbA1c exceeds 8.5%. The insulin Glargine was chosen to represent insulin treatment in the UK and is consistent with Gillett et al. (2012) (97). The total resource use and costs of this health state are reported in Table 5.14.4 & Table 5.14.5.

Table 5.14.4: Costs of insulin treatment

	Cost before Inflation (2006 prices)	Inflation	Unit cost 2022 prices	Source
Insulin Glargine	£628.44			(99)
Oral anti-diabetics	£43.68			
Reagent test strips	£221.43			
Hypoglycaemic rescue	£23.43			
Pen delivery devices	£54.79			
Sharps	£68.82			
Total cost per year	£1,013.51	1.43	1444.62	

Table 5.14.5: Drug costs and resource utilisation costs for insulin and oral anti-diabetics

Resource	Assumption for costs	Source	Unit Cost 2022 prices	SE	Distribution	Annual utilisation	Cost per year

Insulin Treatment Costs	Total Annual Cost	See Table 5.14.4	1444.62	808.34	GAMMA	1	1444.62
Nurse at GP	Advanced GP Nurse per hour for three 15 minute appointment	(93)	50.81	5.08	GAMMA	0.75	38.11
GP Nurse	Band 4 – Qualified Nurse at GP practice per hour for three 10 minute appointments	(93)	38.64	3.86	GAMMA	0.5	19.32
Urine sample	Clinical Biochemistry	(94)	1.55	0.16	GAMMA	3	4.65
Eye screening	Non-Consultant Lead Ophthalmology	(94)	106.42		CONSTANT	1	106.42
HbA1c	Haematology	(94)	2.96	0.30	GAMMA	3	8.88
Lipids	Clinical Biochemistry	(94)	1.55	0.16	GAMMA	3	4.65
Liver function	Clinical Biochemistry	(94)	1.55	0.16	GAMMA	3	4.65
B12	Clinical Biochemistry	(94)	1.55	0.16	GAMMA	3	4.65
					Total annual cost		1636.13

5.14.3 Statins

We assumed that individuals who are prescribed statins receive a daily dose of 40mg of generic Simvastatin. The British National Formulary reports a cost of approximately 3p per 20mg tablet, generating an average annual cost of statins of £19.71 (95). The individual remains on statins for the rest of their life. The cost of GP attendance was not included in the cost of statins to avoid double counting of GP attendance.

5.14.4 Anti-hypertensives

The estimated annual costs of hypertension were taken from the NICE guidelines for hypertension in adults (NG136) (100). The guideline reports the annual cost of medication, which we assume to be maintained in subsequent years of having hypertension, and annual monitorying costs for both the first year and subsequent years. The costs associated with hypertension are reported in Table 5.14.6 and Table 5.14.7.

Table 5.14.6: Annual cost of anti-hypertensive treatment expenditure per patient in first year (£)

Resource	Assumption for costs	Source	Unit Cost before inflation	Unit cost 2022 prices	SE	Distribution	Annual utilisation	Cost per year
Drug Costs	Weight averages of BNF drug costs based on distribution of number and class of drugs.	(100)	18.02	20.33	2.03	GAMMA	1	20.33
Monitoring Costs	Monitoring costs during first year of treatment	(100)	121.5	137.09	13.71	GAMMA	1	137.09
						Total annual cost		157.42

Table 5.14.7: Annual cost of anti-hypertensive treatment expenditure per patient in subsequent years (£)

Resource	Assumption for costs	Source	Unit Cost 2019 prices	Unit cost 2022 prices	SE	Distribution	Annual utilisation	Cost per year
Drug Costs	Weight averages of BNF drug costs based on distribution of number and class of drugs.	(100)	18.02	20.33	2.03	GAMMA	1	20.33
Monitoring Costs	Monitoring costs during subsequent years of treatment	(100)	75	84.62	8.46	GAMMA	1	84.62
						Total annual cost		104.95

5.14.5 Cardiovascular Events

The costs associated with myocardial infarction, unstable angina and transient ischemic attack were obtained from a cohort study using Clinical Practice Research Datalink records (101). However, the cost of stable angina

was unavailable within this source, and instead was estimated using an HTA for high dose lipid-lowering therapy with updated costs (55). Table 5.14.9 described the costs and assumptions used to estimate the cost of stable angina. The costs of stroke were obtained from a Cost and Cost-effectiveness analysis or the Sentinel Stroke National Audit Programme (102, 103). The costs of congestive heart failure were estimated from the UKPDS costing study for complications related to diabetes (104). These unit costs for cardiovascular events are detailed in Table 5.14.8.

Table 5.14.8: Unit costs for Cardiovascular cost estimates

Event		Assumption	Mean cost	Source	Mean cost 2022 prices	SE	Distribution
Stable Angina				See Table 5.14.9	£547.34		
Unstable Angina	Event year	First 6 month cost plus 6 * monthly average cost	£2244.93	(101)	£2727.64	272.76	GAMMA
	Subsequent Years	12* Monthly average cost	£131.38	(101)	£159.63	15.96	GAMMA
Transient Ischemic Attack	Event year	First 6 month cost plus 6 * monthly average cost	£1677.83	(101)	£1696.08	169.61	GAMMA
	Subsequent Years	12* Monthly average cost	£281.90	(101)	£342.52	34.25	GAMMA
Stroke	Event year	First year cost	£13459.00	(103)	£16287.80	1628.78	GAMMA
	Subsequent Years	Average of remaining 4 years of costs	£1118.00	(103)	£1352.98	135.30	GAMMA
MI	Event year	First 6 month cost plus 6 * monthly average cost	£4459.90	(101)	£5418.86	541.89	GAMMA
	Subsequent Years	12* Monthly average cost	£368.97	(101)	£448.31	44.83	GAMMA
Congestive Heart Failure	Event year	Inpatient + non-inpatient minus subsequent year costs		(104)	£2136.80	213.68	GAMMA
Congestive Heart Failure	Subsequent Years	Inpatient + non-inpatient		(104)	£3031.67		GAMMA
Fatal CHD	Event year		£2071	(105)	£2610.52	261.05	GAMMA
Fatal stroke	Event year		£1737	(105)	£2189.51	218.95	GAMMA

Table 5.14.9: Unit costs for Stable Angina

Resource	Assumption	Mean	Source	Mean (2022 prices)	SE	Distribution	Utilisation	Annual Cost
Initial Appointment	3 GP visits (55)	£123	(93)	£117.91		CONSTANT	1	£117.91
Subsequent Appointment	Weighted average of all CC scores, cardiac non-consultant outpatients surgery	£235.48	(94)	£235.48	23.55	GAMMA	1	£235.48
Glytrin Spray	90% of patients receive glytrin spray, isobide monoitrate, one of verapamil or atenolol, and aspirin (55)	£3.85	(95)	£3.80		CONSTANT	0.9	£179.33
Isosorbide mononitrate		£170.82	(95)	£163.77		CONSTANT		
Verapamil		£39.12	(95)	£37.50	5.22	GAMMA		
Atenolol		£7.95	(95)	£7.62	3.76	GAMMA		
Aspirin		£9.52	(95)	£9.13	0.91	GAMMA		

Ramipril	90% of patients receive rampiril (55)	£15.52	(95)	£14.88	1.49	GAMMA	0.9	£13.39
ARB (Candesartan)	10% of patients receive ARB (55)	£12.78	(95)	£12.25	1.23	GAMMA	0.1	£1.23
**Stable Angina**	**Total cost is assumed to be a combination of initial and subsequent appointments plus average medication use.**							**£547.34**

5.14.6 Renal Failure

The cost of renal failure taken from NICE Health economic model report on type 2 diabetes (106). for the UK using relevant published studies. The reported healthcare costs in the first year of occurring the event are £20,897 and the costs or subsequent years after the event are £8,332 at 2020 prices. These costs were inflated to £23,005 and £9,172 in 2022/23 prices. Both costs are assumed to have a standard error equal to 1/10th of the mean and sampled using a gamma distribution.

5.14.7 Foot Ulcers

The annual cost of foot ulcers was taken from a study using Hospital Episodic Statistics data for England to estimate healthcare costs of diabetic foot disease (107). The costs were inflated to 2022 prices and an annual NHS cost of £4,276 was applied in the model. This cost was sampled, assuming a standard error of 1/10th the mean, using a gamma distribution.

5.14.8 Amputation

The cost of amputation in the first year of surgery and subsequent years has been reported in a UKPDS costing study (104). The costs were extracted and inflated to 2022 prices. The cost of amputation in the first year was £15,177 and in subsequent years was £4,218. The unit costs for amputation are reported in Table 5.14.10.

Table 5.14.10: Unit costs associated with amputation.

Unit Cost	Assumption	Mean	Source	Mean (2022 prices)	SE	Distribution	Annual Cost
Inpatient	Event Year	9546	(104)	£11831.70	1979.31	GAMMA	£15,177
Non-inpatient	Event Year	2699	(104)	£3345.25	815.75	GAMMA	
Inpatient	Subsequent Years	1792	(104)	£2221.08	462.89	GAMMA	£4,218
Non-inpatient	Subsequent Years	1611	(104)	£1996.74	264.33	GAMMA	

5.14.9 Blindness

The cost of blindness in the first year of surgery and subsequent years has been reported In a UKPDS costing study (104). The costs were extracted and inflated to 2022 prices. The cost of blindness in the first year was £3,898 and in subsequent years was £1,476, the sum of inpatient and non-inpatient costs. The unit costs for blindness are reported in Table 5.14.11.

Table 5.14.11: Unit costs of Blindness

Resource	Assumption	Mean	Source	Mean (2022 prices)	SE	Distribution	Annual Cost
Inpatient	Event Year	1355	(104)	1679.44	594.43	GAMMA	£3,898

Non-inpatient	Event Year	1790	(104)	2218.60	576.72	GAMMA	
Inpatient	Subsequent Years	453	(104)	561.47	87.27	GAMMA	£1,476
Non-inpatient	Subsequent Years	738	(104)	914.71	102.44	GAMMA	

5.14.10 Cancer

The cost of breast and colorectal cancer is estimated as a one-off fixed cost at diagnosis in the model. This simplifying assumption means that the cost of cancer treatment is independent of survival. We acknowledge that this assumption will affect the timing of costs because all costs are imposed in the first year and subject to less discounting. However, we anticipate that the impact on overall outcomes will not be substantial. A large proportion of costs will be incurred in the first year of treatment (surgery, chemotherapy, radiotherapy). Costs in subsequent years will be lower for patients who achieve remission and survival will be short in patients who relapse. Therefore, the costs are likely to be skewed to the early years post diagnosis.

The cost of breast and colorectal cancer were taken from a retrospective cohort study which estimated the total cost of care for breast and colorectal cancer for 9 years post diagnosis (108). The study reported cost by age category and by stage of cancer. These were weighted by the proportion of patients facing each reported in the paper. Table 5.14.12 reports the overall cost of breast cancer by stage of disease at diagnosis and age. Table 5.14.13 reports the overall cost of colorectal cancer by stage of disease at diagnosis and age. Samples of total cost were taken using an assumed standard error of 1/10th of the mean cost, using a gamma distribution.

Table 5.14.12: Estimated cost of breast cancer

Resource component	Annual price	Price Year	Inflation	Proportion	Weighted Average Cost
Total cost (9 years after diagnosis) for stages 1-2 for ages 18-64	£22,502	2010	1.29	0.49	£11,054.65
Total cost (9 years after diagnosis) for stages 3-4 for ages 18-64	£35,244	2010	1.29	0.07	£2,316.45
Total cost (9 years after diagnosis) for stages 1-2 for ages 65+	£19,479	2010	1.29	0.35	£7,205.38
Total cost (9 years after diagnosis) for stages 3-4 for ages 65+	£25,698	2010	1.29	0.07	£1,878.40
Proportion stages 1-2 for ages 18-64	0.882				
Proportion stages 1-2 for ages 65+	0.835				
Proportion aged 65+	0.443				
		Total cost (2010 prices)			£22,454.87
		Total Inflated Cost (2022 prices)			£28899.01

Table 5.14.13: Estimated cost of colorectal cancer

Resource component	Total Cost	Price Year	Inflation	Proportion	Weighted Average Cost
Total cost (9 years after diagnosis) for stages 1-2 for ages 18-64	£12,938	2010	1.29	0.12	£1,582.14
Total cost (9 years after diagnosis) for stages 3-4 for ages 18-64	£21,128	2010	1.29	0.15	£3,057.52
Total cost (9 years after diagnosis) for stages 1-2 for ages 65+	£13,843	2010	1.29	0.38	£5,276.40
Total cost (9 years after diagnosis) for stages 3-4 for ages 65+	£17,282	2010	1.29	0.35	£6,080.50
Proportion stages 1-2 for ages 18-64	0.46				
Proportion stages 1-2 for ages 65+	0.52				
Proportion aged 65+	0.73				
	Total cost (2010 prices)				£15,996.55

	Total Inflated Cost (2022 prices)	£20,587.27

5.14.11 Osteoarthritis

The annual cost of osteoarthritis were estimated in a report in 2010 (109). In this report the authors estimated the expected cost of osteoarthritis from three previous costing studies. The costs include GP attendance, nurse consultations, replacement surgery, help at home and prescription medications. The estimated annual cost of osteoarthritis was £783 in 2008. In the study 93% of the costs were attributable to direct medical costs and 7% to social care. Therefore, cost of direct medical care in 2022 prices is £973.49. Samples are taken assuming a standard error that is 1/10th of the mean cost, using a gamma distribution.

5.14.12 Depression

Depression is modelled as a chronically recurrent disorder, with patients experiencing further depressive episodes after remission. In the model it is assumed that patients continue to incur costs of depression following an initial diagnosis. These costs reflect ongoing resource use to deal with relapse and prevention of relapse.

A recent trial to prevent secondary depressive episodes collected comprehensive cost data from a sample of individuals with depression (110). The resource uses identified in the control arm were extracted to estimate the costs of depression, with staff costs updated using NHS unit cost collection (93). The costs from this data (inflated or deflated to 2022 prices) were not implemented directly into the SPHR diabetes prevention model as this would have over-estimated the number of GP visits. The model already accounts for GP attendance due to depression. Therefore, a revised estimate of the cost of depression, excluding GP consultation was estimated using updated unit costs. All costs used were sampled assuming a standard error that is 1/10th of the mean, using a gamma distribution. The resource use estimates and revised unit cost estimates used to generate a cost of depression excluding GP utilisation are reported in Table 5.14.14.

Table 5.14.14: Depression utilisation of services and total estimated cost

	**Assumption for costs**	**Unit cost**	**Source of cost**	**Price year**	**Inflation**	**Unit cost 2022 prices**	**Annual utilisation**	**Source of utilisation**	**Cost per year**
Practice nurse at surgery	GP nurse face to face assume 10 mins	£8.83	(93)	2023	0.96	£8.47	1.52	(110)	£12.87
Practice nurse at home visit	GP nurse face to face assume 30 mins	£26.50	(93)	2023	0.96	£25.41	0.02	(110)	£0.51
Practice nurse telephone	GP nurse face to face assume 10 mins	£8.83	(93)	2023	0.96	£8.47	0.11	(110)	£0.93
Health visitor	GP nurse face to face assume 30 mins	£26.50	(93)	2023	0.96	£25.41	0.05	(110)	£1.27
District nurse	GP nurse face to face assume 30 mins	£26.50	(93)	2023	0.96	£25.41	0.01	(110)	£0.25
Other nurse	GP nurse face to face assume 10 mins	£8.83	(93)	2023	0.96	£8.47	0.13	(110)	£1.10
HCA phlebotomist	Clinical support worker 10 mins	£2.17	(110)	2009	1.33	£2.88	0.31	(110)	£0.89
Other primary care	Advanced nurse with qualifications	£14.48	(110)	2009	1.33	£19.19	0.19	(110)	£3.65
Out of hours	Inflated of trial costs	£5.13	(110)	2009	1.33	£6.80	0.23	(110)	£1.56
NHS direct	Inflated of trial costs	£1.89	(110)	2009	1.33	£2.51	0.09	(110)	£0.23
Walk-in centre	Inflated of trial costs	£6.77	(110)	2009	1.33	£8.97	0.21	(110)	£1.88
Prescribed medications	Inflated of trial costs	£61.87	(110)	2009	1.33	£82.01	7.74	(110)	£634.79

Secondary care	Emergency Medicine, Any Investigation	£23.85	(110)	2009	1.33	£31.62	0.26	(110)	£8.22
							Total Annual Cost		£668.16

5.14.13 Dementia

Cost of Diagnosis

A one off cost of diagnosis is incurred in the first year of the disease to account for the costs associated with assessing and diagnosing patients. The most recent cost study of Dementia for the UK estimated the cost of Dementia diagnosis at £650 in 2012/13 prices (82) inflated to £805.64.

Ongoing healthcare costs

The direct health care costs of dementia to the NHS were estimated in an Alzheimer's UK report in 2014 (82). The costs were estimated from a modelling study based on PSSRU aggregate long term care model and PSSRU dementia care model. The report describes costs of care for patients with dementia in 2012 GBP (£). Full details of the costing model are reported elsewhere. Samples were taken assuming a standard error that is 1/10th of the mean, using a gamma distribution. Table 5.14.15 reports the costs of dementia for individuals in community or residential care according to MMSE cognitive score. In the model it is assumed that healthcare costs are met entirely by the NHS. These costs are applied in the model to patients with a dementia diagnosis on an annual basis.

Table 5.14.15: Average annual direct healthcare dementia costs

	**Healthcare costs**		**Proportion of patients in residential care**	**Total cost 2012 prices**	**Total Costs 2022 prices**
	Community	Residential			
Mild (MMSE 21-26)	2,751	4,504	10.4%	2932	£3634.04
Moderate (MMSE 10-20)	2,695	9,438	76.2%	7837	£9713.50
Severe (MMSE 0-9)	11,258	8,689	76.2%	9300	£11526.80

5.14.14 Dental Caries

Children's Dental Costs

While decay is simulated at one time point in the model, the cost of decay is then spread over the following years until age 16. Once they are determined to have treatment for decay, an annual cost is applied each year. This average dental treatment cost per tooth was estimated using the assumptions for the proportion of extractions or fillings completed for each decayed tooth, as well as the cost of treatment for these procedures, taken from a NICE economic evaluation (111). The average cost per decayed tooth is found by finding a weight average cost of these treatment pathways. The average cost per tooth is applied incrementally each year from the point of receiving dental treatment. A linear increase in the number of teeth decayed was estimated using data from The Children's Dental Health Survey (85). The linear relationship was interpolated using the mean number of caries in individuals with some dental caries at age 8 and age 15 and extrapolated to age 5 and age 16. The estimated change in the number of caries each year was 0.16 teeth, thus, the annual cost of decay was estimated at £41 (Table 5.14.16).

Table 5.14.16: Unit costs of treatment for dental decay in individuals under 16 years old.

		**Reported Mean**	**Price Year**	**Source**	**Inflated Mean (2022 prices)**	**Distribution**
Cost of one unit of dental activity (UDA)		£25	2012	(112)	£30.99	GAMMA (Mean,1)
Number of UDA per band 2 treatment		3		(113)		

Cost of filling or extraction in dental practice	3 times the cost of 1 UDA	£75			£92.96	
Cost of dental extraction under general anaesthesia		£1,160	2012	(112)	£1,437.75	CONSTANT
Proportion of Newly Decayed teeth extracted under general anaesthesia		13.9%		(112)		
Proportion of Newly decayed teeth filled or extracted in dental practice (Band 2 treatment)	Assuming all remaining treatment after extraction under GA	86.1%				
Average treatment cost per decayed tooth (DMFT)	Weighted average of treatment pathways.				£279.88	
Average annual treatment cost of dental decay	0.157 teeth per year multiplied by cost per tooth.				£43.98	

Adults Dental Costs

The average cost of dental treatment per newly decayed tooth for adults was estimated using NICE economic evaluation for Oral Health Guidance and NHS dental statistics (2022) (111, 114). The economic evaluation assumed extractions or filling completed in a dental practice were a band 2 treatment, which consists of 3 units of dental activity (111). This assumption is supported by the high proportion of these treatments being band 2 recorded in NHS dental statistics (114). The cost per unit of dental activity will be the same as that used for the Children's dental model, with a mean of £30.99 (inflated from £25 to 2022/23 prices). This cost is sampled using a gamma distribution with alpha of 30.99 and beta of 1. This mean price is supported by the estimated average cost per UDA at £28, considering band 2 (for exempt treatment) within the National Dental Statistics (2022/23).

This unit cost, multiplied by 3 to represent the cost of a band 2 course of treatment as before. This unit cost is assumed to be the cost to the NHS per NHS band 2 treatment. A proportion of patients use private dental services, a proportion pay NHS dental charges per treatment and a portion are exempt from NHS dental charges (115) (Table 5.14.17). A weighted average cost, considering NHS treatment utilisation and payment exemption, is applied per 1 DMFT. The annual cost applied in the model will be £28.28 multiplied by their change in DMFT from the previous year. This treatment cost does not account for extractions under general anaesthesia or for the likely need for refilling over the course of the decayed tooth's life, therefore it will likely be an underestimation.

Table 5.14.17: Unit costs per DMFT for individuals 16 years old and above.

Payment category	N (115)	Assumption	Proportion	Payment to NHS	Cost To NHS (2022 prices)	Net Cost to NHS	Weighted Cost per band 2 treatment
Pay all NHS dental charges	8,549		0.57	£70.70 (80)	£92.96	£22.26	£12.69
Private dentist and pay for treatment	2,731		0.18	£0	£0	£0	£0.00
Exempt from all NHS dental charges	2,119		0.14	£0	£92.96	£92.96	£13.01
Pay all NHS charges and pay for some treatments privately	1,185	50% NHS with charges and 50% private	0.08	0.5 * £70.70 + 0.5 * £0	0.5 * £92.96 + 0.5 * £0	£11.13	£0.89
Partial exemption from NHS dental charges	409	50% NHS with charges and 50% NHS exempt from charges.	0.03	0.5 * £70.70 + 0.5 * £0	£92.96	£57.61	£1.73
Total							£28.28

5.15 Social Care costs

In this analysis the social care costs account for the costs of formal and informal home help, such as home care workers and sheltered warden managers, or family and friends. Whether an individuals received social care is dependent on their BMI and demographics. Additional social care costs, such as residential care or occupational therapy that are associated with dementia and stroke are also accounted for from the point these events occur. Additional social care costs associated with the other health outcomes of the model are not included in this estimate. However, reliable social care costs for other conditions are very hard to obtain because they are less commonly incurred in the prevalent patient population and more likely to be attributed to other factors or ageing more generally.

5.15.1 Social Care Model Conditional on BMI

To simulated social care utilisation and costs, a cross-sectional logistic regression analysis relating social care use to BMI was reproduced from a previous analysis with updated data (116). The analysis used Health Survey for England data from 2017 to 2019 (9, 10, 117), which collected information relating to social care for individuals aged 65 years and over that are not living in institutions such as care homes. Definition of social care includes "home care workers/home helps/personal assistants; members of the reablement/intermediate care staff team; wardens/sheltered housing managers; and council maintenance workers", and excludes "occupational therapists/physiotherapists; voluntary helpers; and cleaners" (116). Summary statistics of the data used within this analysis and the proportion receiving help is provided in Table 5.15.1 and Table 5.15.2.

Table 5.15.1: Summary Statistics of Health Survey for England data age 65 and over

		N	Mean or Count	SD or %
Help Received	No	6714	5303	79%
	Yes	6714	1411	21%
BMI (Valid)		5277	28.24	5.08
Age	65-69	6714	1918	29%
	70-74	6714	1888	28%
	75-79	6714	1315	20%
	80-84	6714	869	13%
	85-89	6714	514	8%
	90+	6714	210	3%
Sex	Male	6714	3126	47%
	Female	6714	3588	53%
Ethnicity	Black	6714	82	1%
	Asian	6714	180	3%
	Other	6714		
Limiting Longstanding Illness	No	6711	3916	58%
	Yes	6711	2795	42%
IMD	1 (Least Deprived)	6714	1595	24%
	2	6714	1619	24%
	3	6714	1401	21%
	4	6714	1155	17%
	5 (Most Deprived)	6714	944	14%

Table 5.15.2: Proportion of data source receiving help by BMI, Age and IMD population groups.

		Didn't Receive Help	Received Help	% of group received help
BMI Category	Underweight/Healthy Weight (<25)	1220	185	13%
	Overweight (25-30)	1926	303	14%
	Obese 1 (30-35)	948	179	16%

	Obese 2 (35-40)	287	103	26%
	Obese 3 (40+)	922	641	41%
Age	65-69	1667	251	13%
	70-74	1630	258	14%
	75-79	1044	271	21%
	80-84	604	265	30%
	85-89	281	233	45%
	90+	77	133	63%
IMD	1 (Least Deprived)	1,350	245	15%
	2	1,361	258	16%
	3	1,109	292	21%
	4	858	297	26%
	5 (Most Deprived)	625	319	34%

A logistic regression was performed to determine the association between whether an individual received social care, formal and/or informal, and BMI, age, sex, deprivation, and ethnicity. A limiting illness indicator was not included in the regression models due to concerns of collinearity. Three regression specifications were assessed and the model with the best model fit was chosen (Table 5.15.3). Regression specification 3 was then performed with multiple imputation, to accommodate missing data, using multiple imputation by chained equations (MICE) in R using the "mice" package. The regression coefficients were used to find a fitted value for each individual based on their characteristics, and this was converted to a probability the equations below. This probability was compared to a random number to determine if they receive social care.

$$P = \frac{1}{1 + \exp(-(Fitted\ Value)}$$

Where,

$$Fitted\ Value = \beta_0 + \beta_1 BMI + \beta_2 BMI^2 + \beta_3 BMI^3 + \beta_4 AGE\ 70\ to\ 74 + \beta_5 AGE\ 75\ to\ 79 + \beta_6 AGE\ 80\ to\ 84 + \beta_7 AGE\ 85\ over$$

Samples of each coefficient (Table 5.15.3) were sampled from a multivariate normal distribution using the variance-covariance from regression specification 3 (Table 5.15.4).

Table 5.15.3: Regression outputs associated with social care utilisation across 3 model specifications

	Model 1		Model 2		Model 3		Model 3 - MI	p-value	
**Variable**	**Coefficients**		**Coefficients**		**Coefficients**		**Coefficients**		
Intercept	5.2590	**	3.2079		1.8758		2.4908	0.1774	
	1.8245		1.8445		1.8542		1.8429		
BMI	-0.7384	***	-0.6050	***	-0.5203	**	-0.5334	0.0020	**
	0.1667		0.1673		0.1678		0.1712		
BMI2	0.0216	***	0.0182	***	0.0156	**	0.0160	0.0021	**
	0.0049		0.0049		0.0049		0.0051		
BMI3	-0.0002	***	-0.0002	**	-0.0001	**	-0.0001	0.0076	**
	0.0000		0.0000		0.0000		0.0000		
Male	ref	ref	ref	ref	ref	ref			
Female			0.6739	***	0.6938	***	0.6116	0.0000	***
			0.0837		0.0846		0.0672		
65-69 years old	ref	ref	ref	ref	ref	ref			
70-74 years old	0.0540		0.0621		0.0644		0.1047	0.2883	
	0.1173		0.1182		0.1191		0.0986		

75-79 years old	0.5371	***	0.5371	***	0.5624	***	0.6443	0.0000	***
	0.1204		0.1216		0.1228		0.1006		
80-84 years old	1.1228	***	1.1691	***	1.1902	***	1.2405	0.0000	***
	0.1271		0.1287		0.1302		0.1058		
85-89 years old	1.6107	***	1.6323	***	1.6505	***	1.9247	0.0000	***
	0.1457		0.1477		0.1499		0.1192		
90+ years old	2.4244	***	2.4505	***	2.5115	***	2.7318	0.0000	***
	0.2136		0.2171		0.2205		0.1676		
Black			0.3154		-0.0034		0.2336	0.3664	
			0.3419		0.3426		0.2586		
Asian			0.6371	**	0.5177	*	0.4974	0.0072	**
			0.2273		0.2300		0.1849		
IMD score 1	ref	ref	ref	ref	ref	ref			
IMD score 2					0.2481		0.0982	0.3423	
					0.1305		0.1034		
IMD score 3					0.5183	***	0.3816	0.0002	***
					0.1297		0.1026		
IMD score 4					0.7385	***	0.6773	0.0000	***
					0.1332		0.1039		
IMD score 5					1.1371	***	1.0854	0.0000	***
					0.1348		0.1066		
HSE Year 2018	-0.0171		-0.00778		-0.0226		-0.0887	0.2662	
	0.0977		0.098581		0.0997		0.0797		
HSE Year 2019	-0.0011		0.012318		-0.0189		-0.0760	0.3386	
	0.0972		0.098151		0.0995		0.0794		
obs	5276		5270		5270				
R2	0.075		0.092		0.111				
AIC	4210		4136		4058				
'***' 0.001 '**' 0.01 '*' 0.05									

Table 5.15.4: Variance – Covariance matrix of regression specification 3.

part a	Intercept	BMI	BMI2	BMI3	Female	70-74 years	75-79 years	80-84 years	85-89 years	90+ years
**Intercept**	3.3964	-0.3122	0.0091	-0.0001	-0.0127	0.0016	0.0091	0.0092	0.0143	0.0121
**BMI**	-0.3122	0.0293	-0.0009	0.0000	0.0008	-0.0006	-0.0014	-0.0015	-0.0021	-0.0021
**BMI2**	0.0091	-0.0009	0.0000	0.0000	0.0000	0.0000	0.0000	0.0000	0.0001	0.0001
**BMI3**	-0.0001	0.0000	0.0000	0.0000	0.0000	0.0000	0.0000	0.0000	0.0000	0.0000
**Female**	-0.0127	0.0008	0.0000	0.0000	0.0045	0.0000	0.0000	0.0002	0.0002	0.0005
**70-74 years**	0.0016	-0.0006	0.0000	0.0000	0.0000	0.0097	0.0050	0.0051	0.0051	0.0051
**75-79 years**	0.0091	-0.0014	0.0000	0.0000	0.0000	0.0050	0.0101	0.0052	0.0052	0.0054
**80-84 years**	0.0092	-0.0015	0.0000	0.0000	0.0002	0.0051	0.0052	0.0112	0.0054	0.0056
**85-89 years**	0.0143	-0.0021	0.0001	0.0000	0.0002	0.0051	0.0052	0.0054	0.0142	0.0058
**90+ years**	0.0121	-0.0021	0.0001	0.0000	0.0005	0.0051	0.0054	0.0056	0.0058	0.0281
**Black**	0.0022	0.0000	0.0000	0.0000	-0.0001	0.0005	-0.0002	-0.0002	-0.0001	0.0005
**Asian**	0.0031	-0.0006	0.0000	0.0000	0.0002	0.0010	0.0011	0.0016	0.0013	0.0019
**IMD 2**	-0.0032	-0.0003	0.0000	0.0000	0.0001	0.0001	0.0003	0.0003	0.0004	0.0003
**IMD 3**	-0.0013	-0.0003	0.0000	0.0000	0.0001	0.0000	0.0001	0.0002	0.0002	0.0002
**IMD 4**	-0.0057	0.0001	0.0000	0.0000	0.0001	0.0000	0.0002	0.0003	0.0005	0.0007
**IMD 5**	-0.0123	0.0006	0.0000	0.0000	0.0002	0.0001	0.0004	0.0005	0.0008	0.0012
**2018**	-0.0054	0.0002	0.0000	0.0000	0.0000	0.0001	0.0001	0.0001	0.0001	0.0000
**2019**	-0.0044	0.0001	0.0000	0.0000	0.0000	0.0000	0.0000	0.0000	0.0000	0.0000

part b	Black	Asian	IMD 2	IMD 3	IMD 4	IMD 5	2018	2019
**Intercept**	0.0022	0.0031	-0.0032	-0.0013	-0.0057	-0.0123	-0.0054	-0.0044
**BMI**	0.0000	-0.0006	-0.0003	-0.0003	0.0001	0.0006	0.0002	0.0001
**BMI2**	0.0000	0.0000	0.0000	0.0000	0.0000	0.0000	0.0000	0.0000
**BMI3**	0.0000	0.0000	0.0000	0.0000	0.0000	0.0000	0.0000	0.0000
**Female**	-0.0001	0.0002	0.0001	0.0001	0.0001	0.0002	0.0000	0.0000
**70-74 years**	0.0005	0.0010	0.0001	0.0000	0.0000	0.0001	0.0001	0.0000
**75-79 years**	-0.0002	0.0011	0.0003	0.0001	0.0002	0.0004	0.0001	0.0000
**80-84 years**	-0.0002	0.0016	0.0003	0.0002	0.0003	0.0005	0.0001	0.0000
**85-89 years**	-0.0001	0.0013	0.0004	0.0002	0.0005	0.0008	0.0001	0.0000
**90+ years**	0.0005	0.0019	0.0003	0.0002	0.0007	0.0012	0.0000	0.0000
**Black**	0.0669	0.0013	-0.0001	-0.0004	-0.0014	-0.0027	-0.0002	-0.0001
**Asian**	0.0013	0.0342	0.0002	-0.0005	-0.0010	-0.0004	-0.0001	0.0001
**IMD 2**	-0.0001	0.0002	0.0107	0.0055	0.0055	0.0055	-0.0002	0.0001
**IMD 3**	-0.0004	-0.0005	0.0055	0.0105	0.0055	0.0056	-0.0003	0.0000
**IMD 4**	-0.0014	-0.0010	0.0055	0.0055	0.0108	0.0057	-0.0001	0.0001
**IMD 5**	-0.0027	-0.0004	0.0055	0.0056	0.0057	0.0114	-0.0003	-0.0003
**2018**	-0.0002	-0.0001	-0.0002	-0.0003	-0.0001	-0.0003	0.0064	0.0031
**2019**	-0.0001	0.0001	0.0001	0.0000	0.0001	-0.0003	0.0031	0.0063

The average number of hours of formal and informal social care in individuals receiving some sort of social care was applied to the model population who were simulated to receive social care (Table 5.15.5). This average was found using Health Survey England 2019 (10). The definition of formal social care included care received from home care workers/home helps/personal assistants; members of the reablement/intermediate care staff team; wardens/sheltered housing managers; and council maintenance workers (116). Informal care was defined as any help received by partners, family members or friends (116). Samples of the hours received were taken assuming a log normal distribution.

Table 5.15.5: Average number of hours of social care received per week, estimated from Health Survey for England (2019).

	Obvs	mean	SD	minimum	maximum
Hours of informal Social Care received last week	404	28	33	0.5	200
Hours of formal Social Care received last week	72	10	20	0.5	168

The hourly cost of formal care was assumed to be the hourly weekday cost of a home care worker at £27, sampled using a gamma distribution with a standard error of 7.2 (118). To cost informal health care an opportunity cost approach was taken. The hourly cost of informal health care was assumed to be the average hourly wage of £ 17.74. This was calculated as the average over 4 quarters from July 2022 to June 2023 using data published using the Labour Force Survey (119).

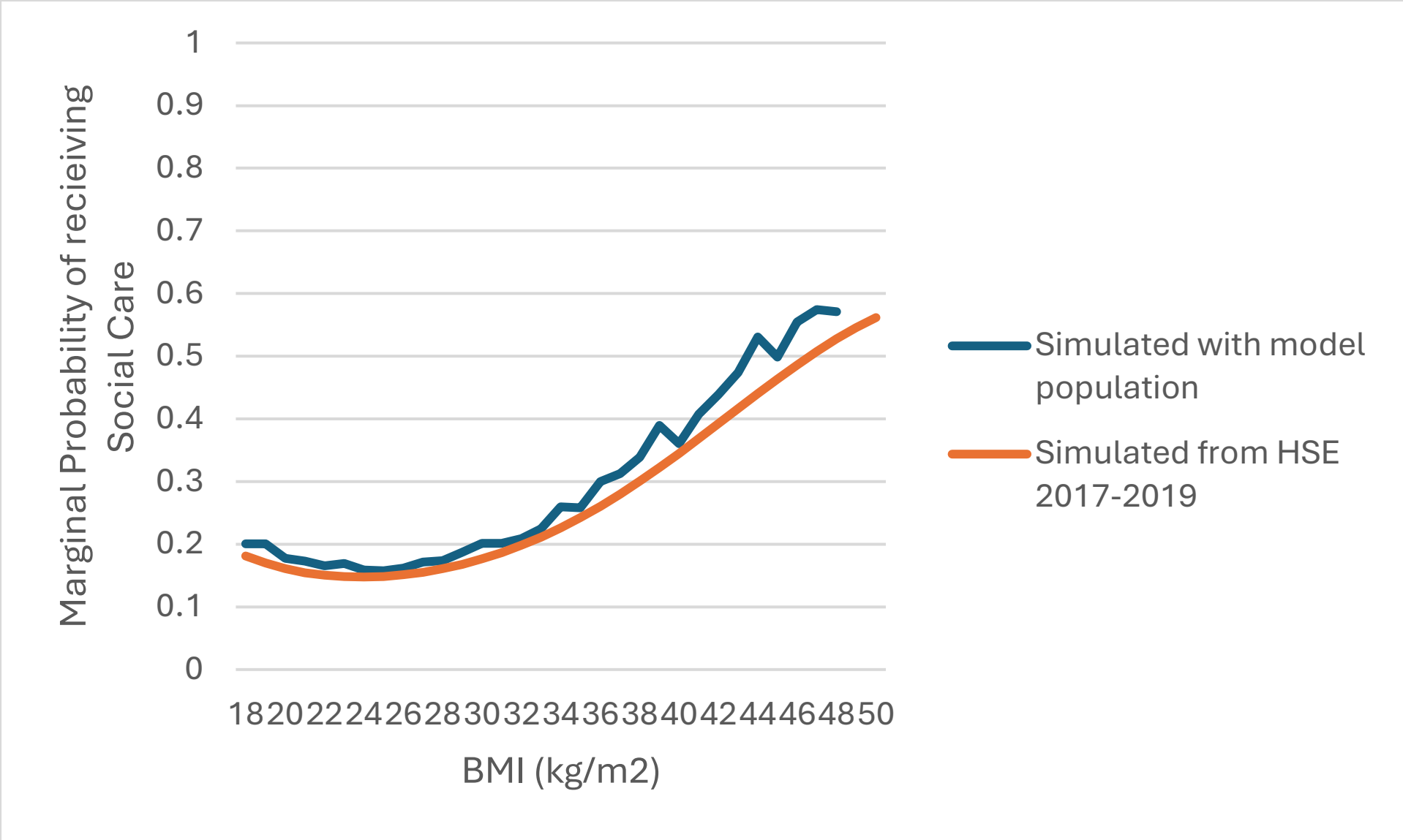

Figure S5.15.1: Plot of probability of receiving social care by BMI.

5.15.2 Stroke

The community costs in the first year following stroke were estimated in a cost-effectiveness analysis using the Sentinel Stroke National Audit Program (66). They report the social care cost associated with residential care. The 5-year cost of residential home care for all stroke types was reported as £10,992. This was assumed to spread evenly over the 5 years and inflated to 2022/23 prices, resulting in an annual cost used of £2,660. This cost was assumed to have a standard error of 1/10th of the mean and sampled using a gamma distribution.

5.15.3 Dementia

The social care costs of dementia were estimated in an Alzheimers UK report in 2014 (82). The costs were estimated from a modelling study based on PSSRU aggregate long term care model and PSSRU dementia care model. The report describes costs of care for patients with dementia in 2012 prices. Table 5.15.6 reports the costs of dementia for individuals in community or residential care according to MMSE cognitive score. We used estimates from the Alzheimers UK report to estimate the public and private social care costs of dementia in line with the methods used in this report. To avoid double counting home help, only the residential costs, weighted by the proportions using residential care conditional on MMSE score, are assigned to all those with Dementia. These costs are applied in the model to patients with a dementia diagnosis on an annual basis.

Table 5.15.6: Average annual dementia costs

	**Social care costs**	**Proportion of patients residential care**	**Total cost 2012 prices**	**Total Costs 2022 prices**
	Residential			
Mild (MMSE 21-26)	£24,737	10.4%	£2573	£3189
Moderate (MMSE 10-20)	£25,715	76.2%	£19595	£24287
Severe (MMSE 0-9)	£25,874	76.2%	£19716	£24437

5.16 Labour market Outcomes

To simulate labour market outcomes conditional on health, an analysis using both Health Survey for England and UK Hoursehold Longitudinal Study data was utilised (9, 120). A linear fixed effects regression estimated the relationship between EQ-5D and social benefit income, while a random effects probit model estimated the probability of receiving any income benefits or being economically inactive or unemployed, also conditional on EQ-5D. EQ-5D is an indicator of health status in the model as it is dependent on health events, aging and BMI. This model is applied to all individuals aged between 16 and 65 years old.

These analyses are considered exploratory, as they are likely affected by unobserved confounding, reverse causality, and specific institutional features. While alternative approaches could aim to identify disease-specific effects, this was not feasible using the household survey data available. Furthermore, relying on published literature to isolate these effects would fail to account for multimorbidity and would risk double counting outcomes.

To estimate each individuals probability of being economically inactive, a simple linear relationship was formed using the coefficient dscribing how EQ-5D is associated with economic inactivity, the mean EQ-5D and proportion of inactive individuals recorded in Health Survey for England 2018. The regression took the form:

$$\Pr(Inactive) = \frac{1}{1+EXP(-(\beta_0+\beta_{EQ5D}(EQ5D)))}.$$

To find the intercept term, β_0, the probability of being inactive was assumed to be the proprtion of individuals recorded as inactive or unemployed, 0.21 (standard error, 0.005), and the mean reported EQ-5D, 0.872 (standard error, 0.002) (9). The standard error of the coefficient of EQ-5D is reported in the paper, while the samples of the intercept are generated from samples of the probabiliy of being inactive and the mean EQ-5D accoridng to their standard errors and a normal distribution. assumed to be $1/10^{th}$ of the intercept. Samples of each coeffcient are taken using these standard errors. The coefficients used to estimate the probability of being economically inactive are reported in Table 5.16.1.

Table 5.16.1: Linear model associating the probability of being inactive with EQ-5D.

Variable	Description	Coefficient	SE
β_0	Intercept	-0.754	
β_1	EQ-5D	-0.673	0.074

The probability estimated for being inactive is compared to a randon number to determine whether an individuals is defined as being inactive.

For all adult individuals, a linear relationship determined the amount of benefits they received last month based on their EQ-5D. To find the intercept term, β_0, the average amount of benefits received in the previous month for all individuals in UKHLS (2020/21) per person, £268.47 (standard error, 4.40) (120), at the mean reported EQ-5D, 0.87 (9).

$$\ln(Social\ benefits\ income) = \beta_0 + \beta_{EQ5D}EQ5D$$

The regression coefficients used within the model are reported in Table 5.16.2. The standard error of the coefficient of EQ-5D is reported in the paper and the coefficient is sampled according to a normal distribution. Samples of the average benefits received and the EQ-5D from a normal distribution based on standard errors from the data sources are used to estimate the intercept term.

Table 5.16.2: Linear model associating benefit income with EQ-5D.

Variable	Description	Coefficient	SE

β_0	Intercept	5.65	
β_1	EQ-5D	-0.065	0.017

Monthly total benefit income is simulated for each individual based on EQ-5D and this is multiplied by 12 to estimate annual benefit income.

The simulated mean marginal probability of inactivity or unemployment and mean benefit incomes, dependent on EQ-5D in the population are plotted in Figure 5.16.1. The average EQ-5D in the population is slightly lower than that reported in HSE (2018).

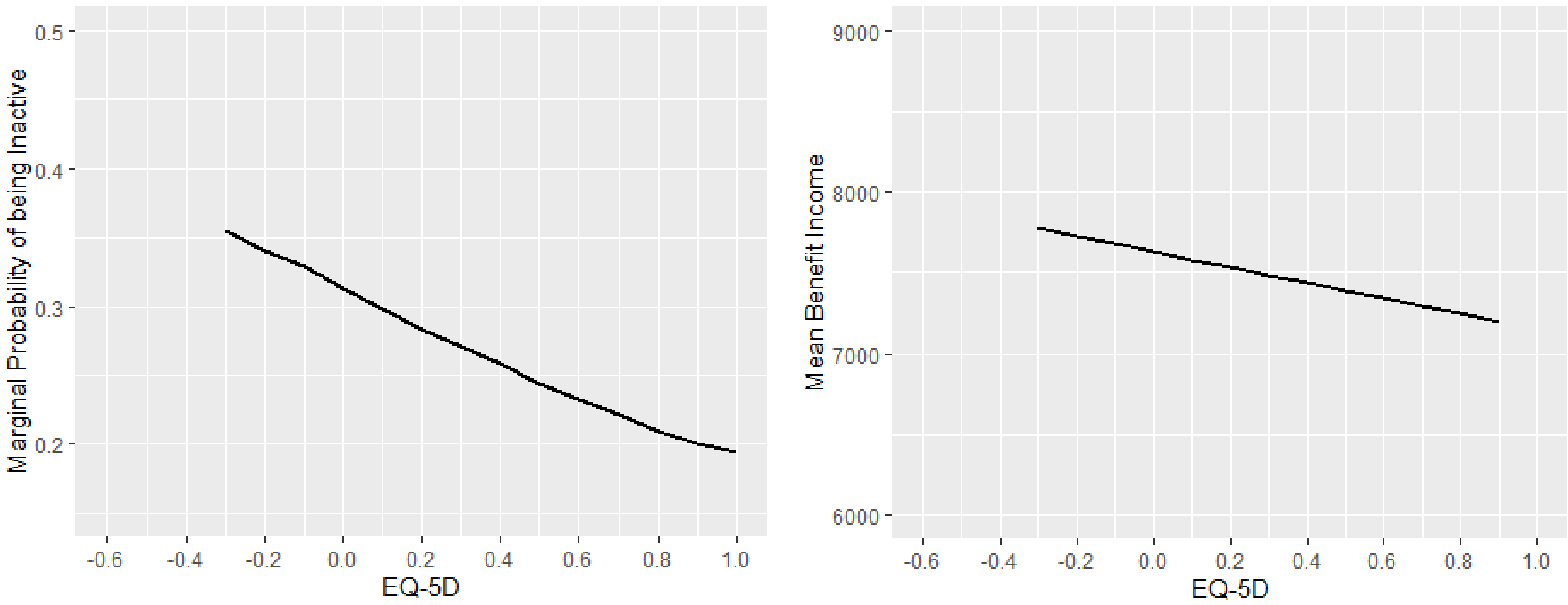

Figure 5.16.1: Plot of simulated labour market outcomes by EQ-5D|

5.17 Utilities

5.17.1 Baseline Utility

Baseline utilities for all individuals in the cohort were extracted from the HSE 2018, EQ-5D was not collected in 2019. Utility was assumed to decline due to ageing independent of health status. In the simulation, utility declines by an absolute decrement of 0.004 per year for those over 22 years old. This estimate is based on previous HTA modelling in cardiovascular disease (18). Utility estimates are also adjusted for changes to BMI. In line with evidence from longitudinal follow-up of individuals seeking weight loss, we apply a utility adjustment to weight loss or weight gain through a relationship with BMI (121). Weight gain over time is associated with worsening utility at -0.0111(s.e. 0.0012) per unit change in BMI, whereas weight loss is associated with equivalent improvement in utility. This change to utility is only applied to individuals in the model with BMI>25.

5.17.2 Utility Decrements

The utility decrements for long term chronic conditions were applied to the age adjusted EQ-5D score. In consultation with stakeholders, we assumed that a diagnosis of diabetes was not associated with a reduction in EQ-5D independent of the utility decrements associated with complications, comorbidities or depression. Dental caries were not associated with a reduction in health-related quality of life. Cardiovascular disease, renal failure, amputation, foot ulcers, blindness, cancer, osteoarthritis and depression were all assumed to result in utility decrements. The utility decrements are measured as a multiplicative factor which is applied to the individual's age adjusted baseline. If individuals have multiple chronic conditions the utility decrements are multiplied together to give the individual's overall utility decrement from comorbidities and complications, in line with NICE guidelines for combining comorbidities (122).

Due to the number of health states it was not practical to conduct a systematic review to identify utility decrements for all health states. A pragmatic approach was taken to search for health states within existing health technology assessments for the relevant disease area or by considering studies used in previous economic models for diabetes prevention. Discussions with experts in health economic modelling were also used to identify prominent sources of data for health state utilities.

Two sources of data were identified for diabetes related complications. A study from the UKPDS estimated the impact of changes in health states from a longitudinal cohort (123). They estimated the impact of myocardial infarction, ischaemic heart disease, stroke, heart failure, amputation and blindness on quality of life using seven rounds of EQ-5D questionnaires administered between 1997 and 2007. This data was used to estimate the utility decrement for amputation and congestive heart failure. The absolute decrement for amputation was converted into utility decrement factors that could be multiplied by the individuals' current EQ-5D to estimate the relative effect of the complication. Blindness was included in the statistical model used for this analysis however the UKPDS analysis reported an increase in health state utility following a diagnosis with blindness. Discussions with the authors highlighted that this was due to treatment following formal classification with blindness and it was decided that this increase in health state utility should not be included in the cost-effectiveness model.

Utility decrements for renal failure and foot ulcers were not available from the UKPDS study described above. A study by Coffey et al. (2000) was used to estimate utility decrements for renal failure and foot ulcers (124). In this study, 2,048 subjects with type 1 and type 2 diabetes were recruited from specialty clinics. The Self-Administered Quality of Well Being index (QWB-SA) was used to calculate a health utility score.

A meta-analysis of utility values for diabetes and diabetes related complications estimated utility decrements for amputation, ulcer, end stage renal failure and blindness (125). The study pooled utility measures using different health state valuation measures in a meta-analysis. Pooling health state utility values is problematic because of

the fact that different valuation methods and different preference-based measures (PBMs) can generate different values on exactly the same clinical health state (126). There were not sufficient studies in the meta-analysis to adjust for the effects of health state valuation measure on the result. This is a limitation of the analysis and we decided that it was preferable to use estimates from single studies.

Utility decrements for cardiovascular events were taken from an HTA assessing statins to reflect the utility decrements in all patients (18) rather than using the UKPDS, which is only representative of a diabetic population. The study conducted a literature review to identify appropriate utility multipliers for stable angina, unstable angina, myocardial infarction and stoke. We used these estimates in the model and assume that transient ischaemic attack is not associated with a utility decrement in line with this HTA.

We identified a systematic review of breast cancer utility studies following consultation with colleagues with experience in this area. The review highlighted a single burden of illness study with a broad utility decrement for cancer (127), rather than utilities by cancer type or disease status. This study was most compatible with the structure of the cost-effectiveness structure. Within this study 1823 cancer survivors and 5469 age-, sex-, and educational attainment-matched control subjects completed EQ-5D questionnaires to estimate utility with and without cancer.

The utility decrement for osteoarthritis was taken from a Health Technology Assessment that assessed the clinical effectiveness and cost-effectiveness of glucosamine sulphate/hydrochloride and chondroitin sulphate in modifying the progression of osteoarthritis of the knee (128).

A review of cost-effectiveness studies highlights the scarcity of studies of health-related quality of life in depression (129). The utility studies identified in the review described depression states by severity and did not adjust for comorbid conditions. Furthermore, the valuations were variable between studies suggesting poor consistency in the estimations. Therefore, it was difficult to apply these in the model. We decided to use a study which had used the EQ-5D in an RCT, for consistency with our utility measure (130). They report an average post treatment utility of 0.67, from which we estimated the utility decrement compared with the average utility reported in the HSE dataset. The decrement was then converted into a relative utility reduction.

The quality of life impact of dementia is estimated from a study by Jonsson and colleagues (131). These utility values were identified and used in the most recent NICE HTA for Alzheimer disease (81). A systematic review of health state utilities for Alzheimer's disease discusses differences in health related quality of life in different settings (96). It is often assumed that patients in institutional settings will be more disabled and have poorer quality of life. However, the studies that compared utility between settings did not identify a statistically significant difference. Therefore, we only related utility to MMSE.

Table 5.17.1 reports the multiplicative utility factors that are used in the model to describe health utility decrements from comorbid complications. The mean absolute decrement estimated in each study is reported alongside the baseline utility for each study. The utility factor was estimated by dividing the implied health utility with the comorbidity by the baseline utility.

Table 5.17.1: Utility decrement factors

	Mean Absolute decrement	St. error absolute decrement	Baseline Utility	Multiplicative Utility Factor	Source
BMI	-0.011	0.002			Breeze (121)
Foot ulcer	-0.099	0.013	0.689	0.856	Coffey (124)
Amputation	-0.172	0.045	0.807	0.787	UKPDS (123)
Blind				1.00	Assumption
Renal failure	-0.078	0.026	0.689	0.887	Coffey (124)
Stable Angina				0.801	Ward HTA (18)
Unstable Angina y1				0.770	Ward HTA (18)
Unstable Angina y2				0.770	Ward HTA (18)
Myocardial Infarction y1				0.760	Ward HTA (18)

Myocardial Infarction y2				0.760	Ward HTA (18)
Transient Ischaemic Attack				1.000	Ward HTA (18)
Stroke y1				0.629	Ward HTA (18)
Stroke y2				0.629	Ward HTA (18)
Breast Cancer	-0.060	0.008	0.791	0.913	Yabroff (127)
Colorectal Cancer	-0.060	0.008	0.791	0.913	Yabroff (127)
Osteoarthritis	-0.101	0.069	0.791		Black HTA (128)
Depression	-0.116		0.791	0.875	Benedict (130)
Congestive Heart Failure	-0.101	0.032		0.875	UKPDS (123)
MMSE 26-30			0.690		Jonsson (131)
MMSE 21-25	-0.05		0.690	0.93	Jonsson (131)
MMSE 15-20	-0.19		0.690	0.725	Jonsson (131)
MMSE 10-14	-0.20		0.690	0.710	Jonsson (131)
MMSE 0-9	-0.36		0.690	0.478	Jonsson (131)
UKPDS baseline utility 0.807; HSE baseline 0.7905					

5.18 Model Validation

The model has undergone a thorough process of error checking and internal and external validations. Validation of the model to predict metabolic data, diabetes and cardiovascular disease have been reported elsewhere. Verification of the Cumulative incidence of major health outcomes were aligned with expected health outcomes for in this population Figure 5.18.1.

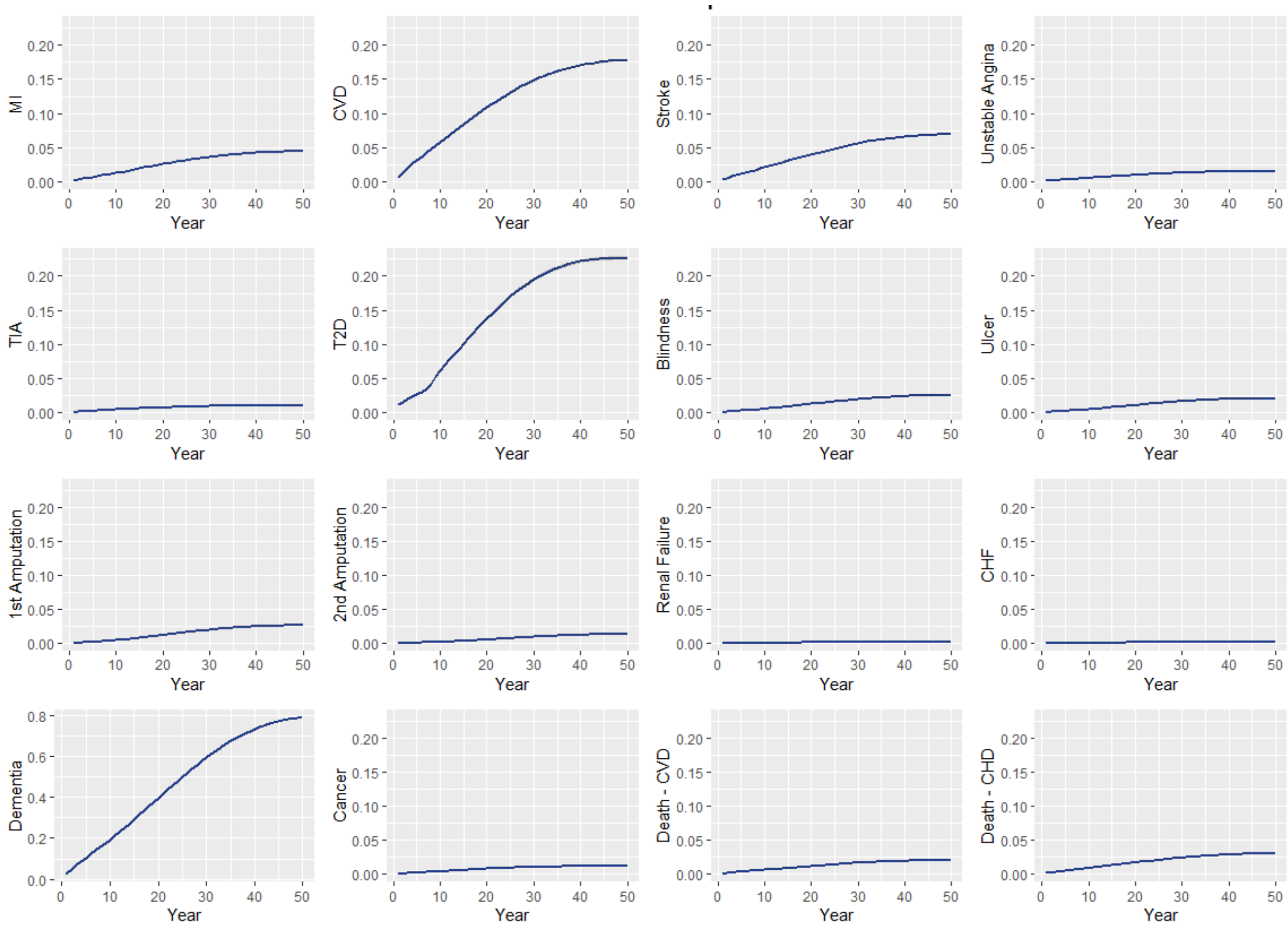

Figure 5.18.1: A plot of the cumulative incidence of complications and mortality over the model simulation.

For this analysis we conducted a series of validation tests to compare the simulated outcomes with the 5 year WRAP follow-up data (132). Given that the unadjusted survival function for the QRISK2 algorithm was calibrated to the 5-year trial data, we included an additional validation test to compare the cardiovascular and diabetes incidence rates to data from the Whitehall II cohort (133).

Table 5.18.1: Validation against cardiovascular and diabetes incidence rates

		**Observed**	**Time-period**	**Simulated**	**95%CI lower**	**95% CI upper**
Diabetes incidence per 1000 person years (132)	All	13.149	2 years	12.833	4.029	24.990
Cardiovascular disease incidence per 1000 person years	All	8.951	5 years	10.223	5.701	18.711
	Men	14.988	5 years	16.206	8.853	27.903
	Women	5.897	5 years	10.365	3.495	25.442
Diabetes incidence per 1000 person years in Whitehall II		18.10*	17 years	14.110	4.029	24.990

Cardiovascular disease incidence per 1000 person years in Whitehall II	12.728*	17 years	12.013	6.682	20.309
* Data obtained for overweight and obese subgroup at baseline with a median follow-up of 17.5 years					

5.19 Quality Adjusted Life years (QALYs)

QALYs are calculated from utility each annual cycle and are discounted at a rate of 3.5% as recommend by NICE (134). Mean discounted lifetime QALYs per person in a no intervention scenario is 15.64. Figure 5.19.1 plots the mean cumulative per person QALYs by age group cohorts. Mean discounted lifetime QALYs per person was 24.92 in those under 16 at baseline, 20.81 for those 16 and over and under 25 and 13.29 in those 25 and over.

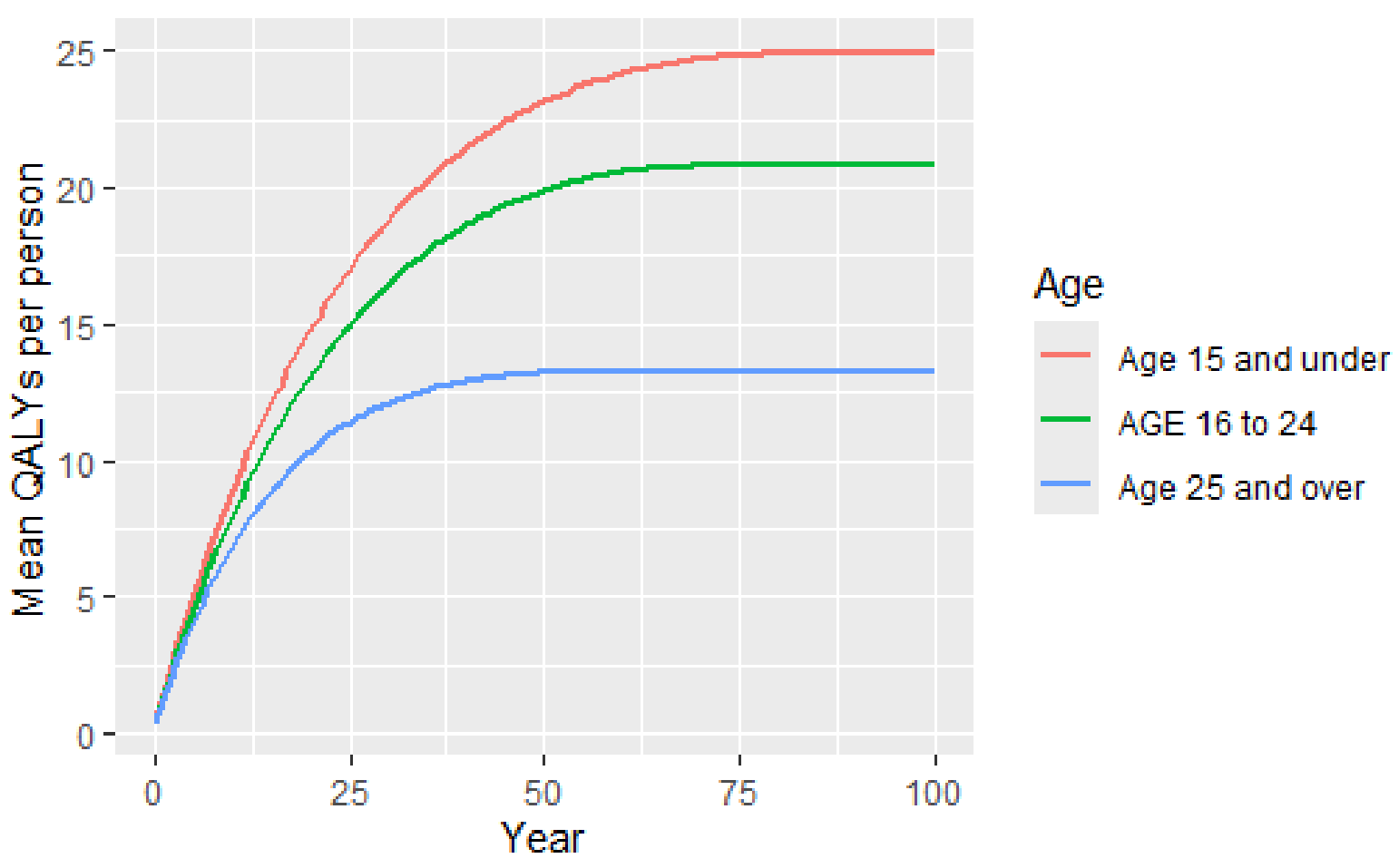

Figure 5.19.1: Mean cumulative QALYs per person over the model simulation by age groups.

6 Parameter Uncertainty

An exploratory probabilistic sensitivity analysis (PSA) of the base case was performed, in which the dietary and health outcomes of a subset of the whole synthetic population with 100,000 randomly selected individuals, are analysed across 400 samples of the parameter inputs. This analysis is considered exploratory due to the smaller number of parameter samples and full stability may have not been achieved. First, the individualised changes to weight and cardiometabolic risk factors for the population subset are estimated in the Demand Model (Supplementary Materials Section 4) across the 400 parameter samples. Second, the long run health outcomes associated with these risk factor changes are evaluated in the Health Model (Supplementary Materials Section 5), again across 400 samples of the parameter inputs. Plots of the cumulative mean of key outcomes across PSA runs are presented alongside the deterministic mean in Figure 6.1.1 and Figure 6.2.1.

The mean outcomes from the PSA analysis sometimes differ from the deterministic mean due to non-linearity in the model. This non-linearity has been observed in previous iterations of the model. The conclusions remain.

6.1 The Demand Model Parameter Uncertainty

Most key parameters were varied by uncertainty, described in Supplementary Materials Section 4. This included the proportion of consumption impacted by the tax, the price elasticities of demand and the relationships between nutritional changes and weight and cardiometabolic risk factors. The baseline population and consumption remain constant across the PSA, as such uncertainty in baseline consumption is not assessed. Additionally, the prices, the proportion of the population that are exposed to the tax for each food group, and the nutritional content of each food group were not varied.

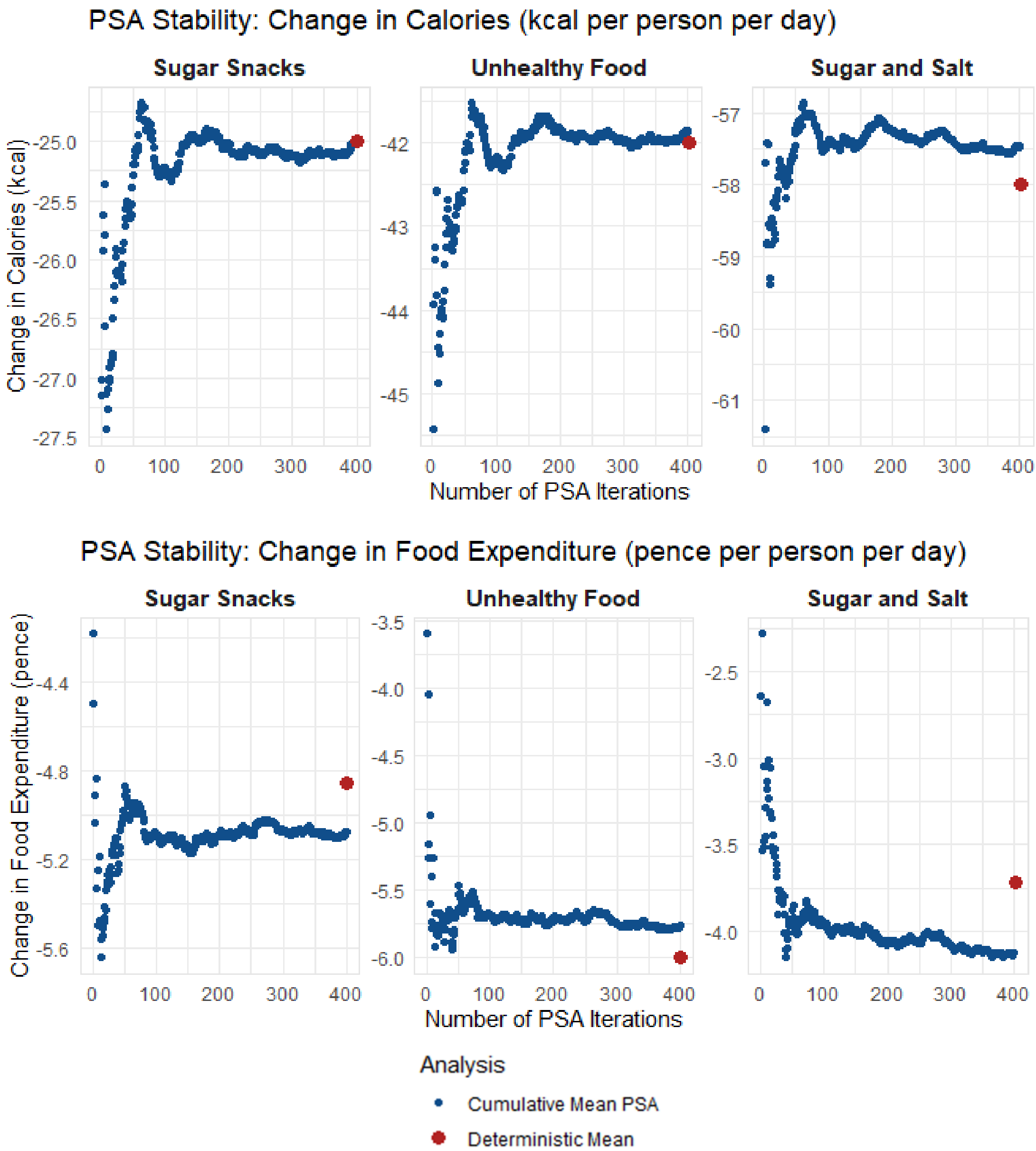

Figure 6.1.1: Model Stability across PSA parameter samples for key outcomes in the Demand Model.

6.2 The Health Model – SMPH Obesity and Diabetes Prevention Model

All parameters were varied according to the uncertainty and distributions described throughout Section 5. The individualised changes to weight, HbA1c, cholesterol, blood pressure and sugar consumption estimated in the Demand Model were applied.

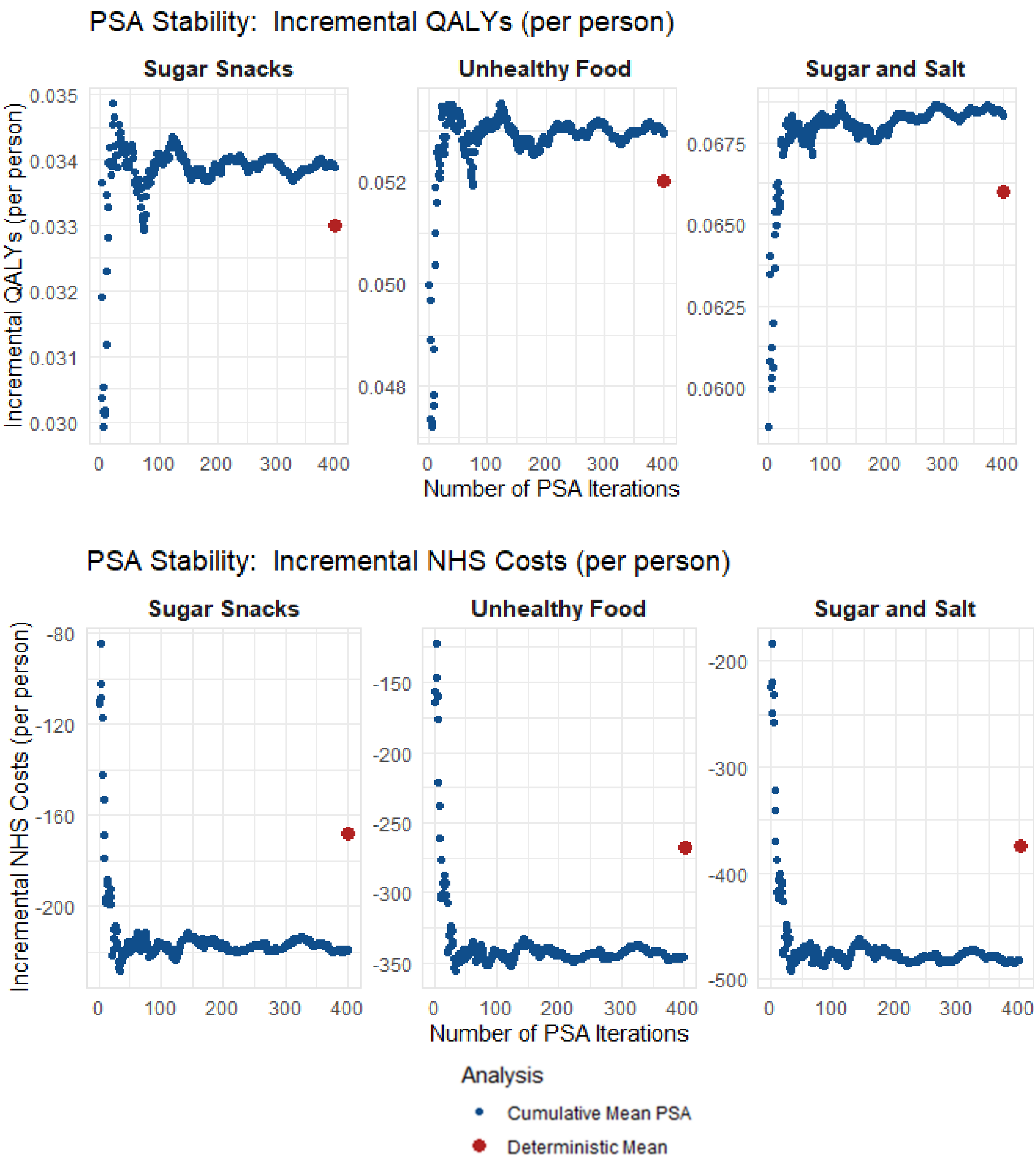

Figure 6.2.1: Model Stability across PSA parameter samples for key outcomes in the Health Model.

7 References

1. Breeze P, Brennan A, Lake A, Reynolds C, Pryce R, Wilson L, et al. Health Economic Analysis incorporating effects on
Labour outcomes, Households, Environment and
Inequalities for food taxes in the United Kingdom - Research Protocol. NIHR Public Health Research; 2024.
2. Research Register. Health Economic Analysis incorporating effects on Labour outcomes, Households, Environment and Inequalities (HEALTHEI) for food taxes in the United Kingdom 2022 [Available from: https://www.researchregistry.com/browse-the-registry/#home/registrationdetails/62fb9d49a0edbc002169adfb/.
3. HM Revenue and Customs. Soft Drinks Industry Levy uprating. GOV.UK; 2024.
4. The Food (Promotion and Placement) (England) Regulations 2021 (ISBN 978-0-348-22619-5): Schedule 1, (2021).
5. Office for Health Improvement and Disparities. Sugar, salt and calorie reduction and reformulation GOV.UK2025 [Available from: https://www.gov.uk/government/collections/sugar-reduction.
6. Nicholas J, Knowles B, Rayson Z. Salt reduction targets for 2024. In: Public Health England: London U, editor. 2020.
7. Office of Health Improvement and Disparities. Sugar reduction programme: report on industry progress 2015 to 2020. In: Office of Health Improvement and Disparities. London U, editor. 2022.
8. Pyne V, Montel S, Targett V, Little E, Owtram G, Tedstone A, et al. Calorie reduction: Technical report: guidelines for industry, baseline calorie levels and the next steps. In: Public Health England: London U, editor. 2017.
9. NatCen Social Research, University College London, Department of Epidemiology and Public Health. Health Survey for England, 2018.: UK Data Service; 2022.
10. NatCen Social Research, University College London, Department of Epidemiology and Public Health. Health Survey for England, 2019. UK Data Service.; 2019.
11. Breeze P, Squires H, Chilcott J, Stride C, Diggle PJ, Brunner E, et al. A statistical model to describe longitudinal and correlated metabolic risk factors: the Whitehall II prospective study. J Public Health (Oxf). 2015.
12. Hippisley-Cox J, Coupland C, Robson J, Brindle P. Derivation, validation, and evaluation of a new QRISK model to estimate lifetime risk of cardiovascular disease: cohort study using QResearch database. BMJ. 2010;341:c6624. doi: 10.1136/bmj.c6624.:c6624.
13. Lovelace R, Dumont M. Spatial microsimulation with R: Chapman and Hall/CRC; 2017.
14. Office for National Statistics. Scotland's Census 2022 - UV607a - National Statistics Socio-economic Classification (NS-SeC) by sex by age (16 groups). In: National Records of Scotland; Northern Ireland Statistics and Research Agency, editor. UK Data Service.
15. Northern Ireland Statistics and Research Agency (NISRA). Census 2021 - DT-0060 - National Statistics Socio-economic Classification - 10 Categories by Age - 12 Categories by Sex. 2021.
16. Office of National Statistics. Census 2021 - RM090 - NS-SEC by Age nomis2021.
17. Office for National Statistics. Mid-Year Population Estimates, UK, June 2022 (MYE22UK).
18. Ward S, Lloyd JM, Pandor A, Holmes M, Ara R, Ryan A, et al. A systematic review and economic evaluation of statins for the prevention of coronary events. Health Technol Assess. 2007;11(14):1-iv.
19. University of Cambridge MRC Epidemiology Unit NatCen Social Research. National Diet and Nutrition Survey Years 1-11, 2008-2019. In: Service. UD, editor. (2021).
20. Department for Environment Food and Rural Affairs. Family Foods Datasets. GOV.UK2024.
21. Blake D, Nied A. The demand for alcohol in the United Kingdom. Applied Economics. 1997;29(12):1655-72.
22. Department for Environment Food and Rural Affairs Office for National Statistics. Living Costs and Food Survey, 2006-2021: Secure Access. . In: Service UD, editor. 2023.
23. Deaton A, Muellbauer J. An almost ideal demand system. The American economic review. 1980;70(3):312-26.
24. Deaton A. Quality, quantity, and spatial variation of price. The American economic review. 1988:418-30.
25. Barten A. Maximum likelihood estimation of a complete system of demand equations. European economic review. 1969;1(1):7-3.
26. Luick M, Bandy L, Harrington R, Vijayan J, Adams J, Cummins S, et al. The impact of the UK soft drink industry levy on the soft drink marketplace, 2017–2020: An interrupted time series analysis with comparator series. . PloS one. 2024;19(6):e0301890.

27. Hall K, Sacks G, Chandramohan D, Chow C, Wang Y, Gortmaker S, et al. Quantification of the effect of energy imbalance on bodyweight. The Lancet. 2011;378(9793):826-37.
28. National Institute of Diabetes and Digestive and Kidney Diseases. Body Weight Planner: Balancing Your Food Activity [Available from: https://www.niddk.nih.gov/bwp.
29. Hall K, Butte N, Swinburn B, Chow C. Dynamics of childhood growth and obesity: development and validation of a quantitative mathematical model. The lancet Diabetes & endocrinology 2013;1(2):97-105.
30. Breeze P, Sworn K, McGrane E, Abraham S, Cantrell A. Relationships between sodium, fats and carbohydrates on blood pressure, cholesterol and HbA1c: an umbrella review of systematic reviews. . BMJ Nutrition, Prevention & Health. 2024;7(1):191.
31. Graudal N, Hubeck-Graudal T, Jürgens G. Effects of low-sodium diet vs. high-sodium diet on blood pressure, renin, aldosterone, catecholamines, cholesterol, and triglyceride (Cochrane Review). American journal of hypertension. 2012;25(1):1-15.
32. Qian F, Korat A, Malik V, Hu F. Metabolic effects of monounsaturated fatty acid–enriched diets compared with carbohydrate or polyunsaturated fatty acid–enriched diets in patients with type 2 diabetes: a systematic review and meta-analysis of randomized controlled trials. . Diabetes care. 2016;39(8):1448-57.
33. Imamura F, Micha R, Wu J, de Oliveira Otto MO, FO., Abioye A, Mozaffarian D. Effects of saturated fat, polyunsaturated fat, monounsaturated fat, and carbohydrate on glucose-insulin homeostasis: a systematic review and meta-analysis of randomised controlled feeding trials. . PLoS medicine 2016;13(7):e1002087.
34. Miller P, Van Elswyk M, Alexander D. Long-chain omega-3 fatty acids eicosapentaenoic acid and docosahexaenoic acid and blood pressure: a meta-analysis of randomized controlled trials. American journal of hypertension. 2014;27(7):885-96.
35. Abdelhamid A, Martin N, Bridges C, Brainard J, Wang X, Brown T, et al. Polyunsaturated fatty acids for the primary and secondary prevention of cardiovascular disease. The Cochrane database of systematic reviews. 2018(7).
36. Mensink RWhO. Effects of saturated fatty acids on serum lipids and lipoproteins: a systematic review and regression analysis. 2016.
37. Fechner E, Smeets E, Schrauwen P, Mensink R. The effects of different degrees of carbohydrate restriction and carbohydrate replacement on cardiometabolic risk markers in humans—a systematic review and meta-analysis. . Nutrients. 2020;12(4):991.
38. Reynolds A, Mann J, Cummings J, Winter N, Mete E, Te Morenga L. Carbohydrate quality and human health: a series of systematic reviews and meta-analyses. . The Lancet. 2019;393(10170):434-45.
39. Jovanovski E, Khayyat R, Zurbau A, Komishon A, Mazhar N, Sievenpiper J, et al. Should viscous fiber supplements be considered in diabetes control? Results from a systematic review and meta-analysis of randomized controlled trials. . Diabetes Care. 2019;42(5):755-66.
40. Te Morenga L, Howatson A, Jones R, Mann J. Dietary sugars and cardiometabolic risk: systematic review and meta-analyses of randomized controlled trials of the effects on blood pressure and lipids. The American journal of clinical nutrition. 2014;100(1):65-79.
41. Pidd K, Breeze P, Ahern A, Griffin S, Brennan A. Effects of weight loss and weight gain on HbA1c, systolic blood pressure and total cholesterol in three subgroups defined by blood glucose: a pooled analysis of two behavioural weight management trials in England. BMJ open. 2025;15(4):e095046.
42. Turner-Harris L, Mariani E, Bowes Byatt L, , Stewart I, Widdison C. The future of food: Opportunities to improve health through reformulation. NESTA; 2023.
43. McKenzie EL, SY. Sugar reduction methods and their application in confections: a review. Food Science and Biotechnology. 2022;31:387-98.
44. da Silva Faresin L, Devos R, Reinehr C, Colla L. Development of ice cream with reduction of sugar and fat by the addition of inulin, Spirulina platensis or phycocyanin. International Journal of Gastronomy and Food Science. 2022;27:100445.
45. Pineda E, Beaney T. Potassium-enriched salt: a new era for UK salt reduction? The Lancet Public Health. 2025;10:e436-7.
46. Yin X, Tian M, Sun L, Webster J, Trieu K, Huffman M, et al. Barriers and facilitators to implementing reduced-sodium salts as a population-level intervention: a qualitative study. Nutrients. 2021;13:3225.
47. Squires H, Chilcott J, Akehurst R, Burr J, Kelly MP. A framework for developing the structure of public health economic models. Value in Health. 2016;19(5):588-601.
48. National Institute for Health and Care Excellence. Type 2 diabetes prevention: population and community-level interventions. Public health guideline [PH35]. 2011.
49. National Institute for Health Clinical Excellence. Preventing type 2 diabetes: risk identification and interventions for individuals at high risk. NICE Guidelines PH38. 2012.

50. Breeze P, Thomas C, Thokala P, Lafortune L, Brayne C, Brennan A. The impact of including costs and outcomes of dementia in a health economic model to evaluate lifestyle interventions to prevent diabetes and cardiovascular disease. Medical Decision Making. 2020;40(7):912-23.
51. Breeze P, Lake A, Moore H, Connor N, Burrows A, Pidd K, et al. Developing a novel conceptual model of how UK food and drink tax policy impacts on consumption, health, environmental and economic outcomes. [Preprint].
52. Rabe-Hesketh S, Skrondal A. Multilevel and longitudinal modeling using Stata: STATA press; 2008.
53. Young KG, McGovern AP, Barroso I, Hattersley AT, Jones AG, Shields BM, et al. The impact of population-level HbA1c screening on reducing diabetes diagnostic delay in middle-aged adults: a UK Biobank analysis. Diabetologia. 2023;66(2):300-9.
54. Clarke P, Gray A, Briggs A, Farmer A, Fenn P, Stevens R, et al. A model to estimate the lifetime health outcomes of patients with type 2 diabetes: the United Kingdom Prospective Diabetes Study (UKPDS) Outcomes Model (UKPDS no. 68). Diabetologia. 2004;47(10):1747-59.
55. Ara R, Pandor A, Stevens J, Rees A, Rafia R. Early high-dose lipid-lowering therapy to avoid cardiac events: a systematic review and economic evaluation. Health Technology Assessment. 2009;13(34):1-118.
56. National Institute for Health Clinical Excellence. Hypertension: clinical management of primary hypertension in adults: NICE; 2011.
57. Wald DS, Law M, Morris JK, Bestwick JP, Wald NJ. Combination therapy versus monotherapy in reducing blood pressure: meta-analysis on 11,000 participants from 42 trials. The American journal of medicine. 2009;122(3):290-300.
58. Hippisley-Cox J, Coupland C. Development and validation of QDiabetes-2018 risk prediction algorithm to estimate future risk of type 2 diabetes: cohort study. bmj. 2017;359.
59. Watson P, Preston L, Squires H, Chilcott J, Brennan A. Modelling the economics of type 2 diabetes mellitus prevention: a literature review of methods. Applied health economics and health policy. 2014;12(3):239-53.
60. Hayes AJ, Leal J, Gray A, Holman R, Clarke P. UKPDS outcomes model 2: a new version of a model to simulate lifetime health outcomes of patients with type 2 diabetes mellitus using data from the 30 year United Kingdom Prospective Diabetes Study: UKPDS 82. Diabetologia. 2013;56(9):1925-33.
61. D'Agostino SR, Vasan R, Pencina M, Wolf P, Cobain M, Massaro J, et al. General cardiovascular risk profile for use in primary care: the Framingham Heart Study. Circulation. 2008;117(6):743-53.
62. Hippisley-Cox J, Coupland C, Vinogradova Y, Robson J, Minhas R, Sheikh A, et al. Predicting cardiovascular risk in England and Wales: prospective derivation and validation of QRISK2. Bmj. 2008;336(7659):1475-82.
63. McEwan P, Bennett H, Ward T, Bergenheim K. Refitting of the UKPDS 68 risk equations to contemporary routine clinical practice data in the UK. Pharmacoeconomics. 2015;33(2):149-61.
64. Khaw K-T, Wareham N, Luben R, Bingham S, Oakes S, Welch A, et al. Glycated haemoglobin, diabetes, and mortality in men in Norfolk cohort of European Prospective Investigation of Cancer and Nutrition (EPIC-Norfolk). Bmj. 2001;322(7277):15.
65. Kannel WB, D'Agostino RB, Silbershatz H, Belanger AJ, Wilson PW, Levy D. Profile for estimating risk of heart failure. Archives of internal medicine. 1999;159(11):1197-204.
66. Kaffashian S, Dugravot A, Brunner EJ, Sabia S, Ankri J, Kivimäki M, et al. Midlife stroke risk and cognitive decline: a 10-year follow-up of the Whitehall II cohort study. Alzheimer's & Dementia. 2013;9(5):572-9.
67. Johansen NB, Vistisen D, Brunner EJ, Tabák AG, Shipley MJ, Wilkinson IB, et al. Determinants of aortic stiffness: 16-year follow-up of the Whitehall II study. PloS one. 2012;7(5):e37165.
68. Dadvand P, Rankin J, Shirley MD, Rushton S, Pless-Mulloli T. Descriptive epidemiology of congenital heart disease in Northern England. Paediatric and perinatal epidemiology. 2009;23(1):58-65.
69. Davies M, Hobbs F, Davis R, Kenkre J, Roalfe A, Hare R, et al. Prevalence of left-ventricular systolic dysfunction and heart failure in the Echocardiographic Heart of England Screening study: a population based study. The Lancet. 2001;358(9280):439-44.
70. Lahmann PH, Hoffmann K, Allen N, Van Gils CH, Khaw KT, Tehard B, et al. Body size and breast cancer risk: findings from the European Prospective Investigation into Cancer And Nutrition (EPIC). International journal of cancer. 2004;111(5):762-71.
71. Pischon T, Lahmann PH, Boeing H, Friedenreich C, Norat T, Tjønneland A, et al. Body size and risk of colon and rectal cancer in the European Prospective Investigation Into Cancer and Nutrition (EPIC). Journal of the National Cancer Institute. 2006;98(13):920-31.

72. Renehan AG, Tyson M, Egger M, Heller RF, Zwahlen M. Body-mass index and incidence of cancer: a systematic review and meta-analysis of prospective observational studies. The lancet. 2008;371(9612):569-78.
73. Schett G, Kleyer A, Perricone C, Sahinbegovic E, Iagnocco A, Zwerina J, et al. Diabetes is an independent predictor for severe osteoarthritis: results from a longitudinal cohort study. Diabetes care. 2013;36(2):403-9.
74. Palmer AJ, Roze S, Valentine WJ, Minshall ME, Foos V, Lurati FM, et al. The CORE Diabetes Model: projecting long-term clinical outcomes, costs and costeffectiveness of interventions in diabetes mellitus (types 1 and 2) to support clinical and reimbursement decision-making. Current medical research and opinion. 2004;20(sup1):S5-S26.
75. Golden SH, Lazo M, Carnethon M, Bertoni AG, Schreiner PJ, Roux AVD, et al. Examining a bidirectional association between depressive symptoms and diabetes. Jama. 2008;299(23):2751-9.
76. Whyte EM, Mulsant BH, Vanderbilt J, Dodge HH, Ganguli M. Depression after stroke: a prospective epidemiological study. Journal of the American Geriatrics Society. 2004;52(5):774-8.
77. Walters K, Hardoon S, Petersen I, Iliffe S, Omar R, Nazareth I, et al. Predicting dementia risk in primary care: development and validation of the Dementia Risk Score using routinely collected data. BMC medicine. 2016;14(1):6.
78. Briggs A, Sculpher M, Claxton K. Decision modelling for health economic evaluation: Oup Oxford; 2006.
79. Wimo A, Religa D, Spångberg K, Edlund AK, Winblad B, Eriksdotter M. Costs of diagnosing dementia: results from SveDem, the Swedish Dementia Registry. International journal of geriatric psychiatry. 2013;28(10):1039-44.
80. Xie J, Brayne C, Matthews FE. Survival times in people with dementia: analysis from population based cohort study with 14 year follow-up. bmj. 2008;336(7638):258-62.
81. Bond M, Rogers G, Peters J, Anderson R, Hoyle M, Miners A, et al. The effectiveness and cost-effectiveness of donepezil, galantamine, rivastigmine and memantine for the treatment of Alzheimer's disease (review of Technology Appraisal No. 111): a systematic review and economic model. Health technology assessment (Winchester, England). 2012;16(21):1.
82. Prince M, Knapp M, Guerchet M, McCrone P, Prina P, Comas-Herrera A, et al. Dementia UK Update. Cognitive Function and Ageing Study [Internet]. 2014. Available from: http://www.cfas.ac.uk/files/2015/07/P326_AS_Dementia_Report_WEB2.pdf.
83. Getsios D, Blume S, Ishak K, Maclaine G. Cost effectiveness of donepezil in the treatment of mild to moderate Alzheimer's disease: a UK evaluation using discrete-event simulation. Pharmacoeconomics. 2010;28(5):411-27.
84. Masson LF BA, Sheehy C, Craig LC, Macdiarmid JI, Holmes BA, McNeill G. Sugar intake and dental decay: results from a national survey of children in Scotland. . British journal of nutrition. 2010;104(10):1555-64.
85. Pitts N, Chadwick B, Anderson T. Children’s Dental Health Survey 2013. Report 2: Dental Disease and Damage in Children: England, Wales and Northern Ireland NHS Digital; 2015.
86. Adult Dental Health Survey, 2009. [data collection]. 2nd Edition. UK Data Service. SN: 6884. In: Office for National Statistics. Social Survey Division ICfHaSC, editor. 2012.
87. Bernabé E, Vehkalahti MM, Sheiham A, Lundqvist A, Suominen AL. The shape of the dose-response relationship between sugars and caries in adults. Journal of Dental Research. 2016;95(2):167-72.
88. Cancer Survival in England: Patients Diagnosed, 2006–2010 and Followed up to 2011. Office of National Statistics [Internet]. 2012. Available from: http://www.ons.gov.uk/ons/publications/re-reference-tables.html?edition=tcm%3A77-277733.
89. National life tables: UK. Office of National Statistics [Internet]. 2022. Available from: https://www.ons.gov.uk/peoplepopulationandcommunity/birthsdeathsandmarriages/lifeexpectancies/datasets/nationallifetablesunitedkingdomreferencetables.
90. Mortality Statistics: Deaths registered in England and Wales (Series DR), 2022. Office of National Statistics [Internet]. 2022. Available from: http://webarchive.nationalarchives.gov.uk/20160105160709/http://www.ons.gov.uk/ons/publications/re-reference-tables.html?edition=tcm%3A77-378961.
91. Seshasai S, Kaptoge S, Thompson A, Di AE, Gao P, Sarwar N, et al. Diabetes mellitus, fasting glucose, and risk of cause-specific death. N Engl J Med. 2011;364(9):829-41.
92. James B, Leurgans S, Hebert L, Scherr P, Yaffe K, Bennett D. Contribution of Alzheimer disease to mortality in the United States. Neurology. 2014;82(12):1045-50.

93. Jones KC, Weatherly H, Birch S, Castelli A, Chalkley M, Dargan A, et al. Unit Costs of Health and Social Care 2024 Manual. 2025.
94. 2023/24 National Cost Collection Data Publication [Internet]. NHS England.
95. British National Formulary. http://www bnf org/ [Internet]. 2023.
96. CG127 Hypertension: costing template. National Institute for Care and Clinical Excellence [Internet]. 2011. Available from: http://guidance.nice.org.uk/CG127/CostingTemplate/xls/English.
97. Gillett M, Royle P, Snaith A, Scotland G, Poobalan A, Imamura M, et al. Non-pharmacological interventions to reduce the risk of diabetes in people with impaired glucose regulation: a systematic review and economic evaluation. Health Technol Assess. 2012;16(33):1-iv.
98. Belsey JD, Pittard JB, Rao S, Urdahl H, Jameson K, Dixon T. Self blood glucose monitoring in type 2 diabetes. A financial impact analysis based on UK primary care. Int J Clin Pract. 2009;63(3):439-48.
99. Poole C, Tetlow T, McEwan P, Holmes P, Currie C. The prescription cost of managing people with type 1 and type 2 diabetes following initiation of treatment with either insulin glargine or insulin determir in routine general practice in the UK: a retrospective database analysis. Current Medical Research and Opinion. 2007;23(1):S41-S8.
100. National Institute for Health and Care Excellence. Hypertension in adults: diagnosis and management. Cost effectiveness analysis: Treatment initiation threshold for people with stage 1 hypertension.; 2019.
101. Danese M, Gleeson M, Kutikova L, Griffiths R, Azough A, Khunti K, et al. Estimating the economic burden of cardiovascular events in patients receiving lipid-modifying therapy in the UK. . BMJ open 2016;6(8):e011805.
102. Luengo-Fernandez R, Gray AM, Rothwell PM. A population-based study of hospital care costs during 5 years after transient ischemic attack and stroke. Stroke. 2012;43(12):3343-51.
103. National Guideline Centre & SSNAP. Sentinel Stroke National Audit Programme: Cost and Cost-effectiveness analysis (Technical Report)2016. Available from: https://www.strokeaudit.org/SupportFiles/Documents/Health-economics/Health-economic-report-2016.aspx.
104. Alva ML, Gray A, Mihaylova B, Leal J, Holman RR. The impact of diabetes-related complications on healthcare costs: new results from the UKPDS (UKPDS 84). Diabetic medicine. 2015;32(4):459-66.
105. Walker S, Asaria M, Manca A, Palmer S, Gale C, Shah A, et al. Long-term healthcare use and costs in patients with stable coronary artery disease: a population-based cohort using linked health records (CALIBER). . European Heart Journal–Quality of Care and Clinical Outcomes. 2016;2(2):125-40.
106. National Institute for Health and Care Excellence. NG28 Health Economic Model Report2015 18.10.2023. Available from: https://www.nice.org.uk/guidance/ng28/evidence/health-economic-model-report-pdf-10959500845.
107. Kerr M, Barron E, Chadwick P, Evans T, Kong W, Rayman G, et al. The cost of diabetic foot ulcers and amputations to the National Health Service in England. Diabetic Medicine. 2019;36(8):995-1002.
108. Laudicella M, Walsh B, Burns E, Smith P. Cost of care for cancer patients in England: evidence from population-based patient-level data. . British journal of cancer 2016;114(11):1286-92.
109. The economic costs of arthritis for the UK economy. Oxford Economics [Internet]. 2014. Available from: https://www.oxfordeconomics.com/publication/open/222531.
110. Chalder M, Wiles NJ, Campbell J, Hollinghurst SP, Searle A, Haase AM, et al. A pragmatic randomised controlled trial to evaluate the cost-effectiveness of a physical activity intervention as a treatment for depression: the treating depression with physical activity (TREAD) trial. Health Technol Assess. 2012;16(10):1-iv.
111. Lord J, Longworth L, Singh J, Onyimadu O, Fricke J, Bayliss S, et al. Oral Health Guidance – Economic analysis of oral health promotion approaches for dental teams. National Institute for Health and Care Excellence,; 2015.
112. National Institute for Health and Care Excellence. Oral Health Guidance – Economic analysis of oral health promotion approaches for dental teams (NG30). National Institute for Health and Care Excellence; 2015.
113. National Health Service Business Services Authority. How many units of activity (UDA / UOA) does a course of treatment (COT) receive? : National Health Service Business Services Authority,; 2024 [Available from: https://faq.nhsbsa.nhs.uk/knowledgebase/article/KA-01976.
114. Domain PC, NHS England. NHS Dental Statistics for England - 2022/23: National Overview. NHS England2023.
115. Public Health England. Oral health survey of adults attending dental practices 2018. 2020.
116. Copley VR, Cavill N, Wolstenholme J, Fordham R, Rutter H. Estimating the variation in need for community-based social care by body mass index in England and associated cost: population-based cross-sectional study. BMC Public Health. 2017;17:1-11.

117. University College London, Department of Epidemiology and Public Health, National Centre for Social Research. Health Survey for England, 2017.: UK Data Service.; 2017.
118. Jones K BA. Unit costs of health and social care. PSSRU; 2021.
119. Labour Market Team. EARN08: Distribution of gross hourly earnings of employees. In: Statistics OoN, editor. 2024.
120. University of Essex Institute for Social and Economic Research. Understanding Society: Waves 1-13, 2009-2022 and Harmonised BHPS: Waves 1-18, 1991-2009. [data collection]. 18th Edition. ed. UK Data Service.2023.
121. Breeze P, Gray LA, Thomas C, Bates SE, Brennan A. Estimating the impact of changes in weight and BMI on EQ-5D-3L: a longitudinal analysis of a behavioural group-based weight loss intervention. Quality of Life Research. 2022;31(11):3283-92.
122. Ara R, Wailoo A. NICE DSU Technical Support Document 12: The use of health state utility values in decision models. 2011.
123. Alva M, Gray A, Mihaylova B, Clarke P. The Effect of Diabetes Complications on Health-Related Quality of Life: The importance of longitudinal data to address patient heterogeneity. Health economics. 2014;23(4):487-500.
124. Coffey JT, Brandle M, Zhou H, Marriott D, Burke R, Tabaei BP, et al. Valuing health-related quality of life in diabetes. Diabetes Care. 2002;25(12):2238-43.
125. Lung TW, Hayes AJ, Hayen A, Farmer A, Clarke PM. A meta-analysis of health state valuations for people with diabetes: explaining the variation across methods and implications for economic evaluation. Qual Life Res. 2011;20(10):1669-78.
126. Peasgood T, Brazier J. Is Meta-Analysis for Utility Values Appropriate Given the Potential Impact Different Elicitation Methods Have on Values? Pharmacoeconomics. 2015;33(11):1101-5.
127. Yabroff K, Lawrence W, Clauser S, Davis W, Brown M. Burden of illness in cancer survivors: findings from a population-based national sample. J Natl Cancer Inst. 2004;96(17):1322-30.
128. Black C, Clar C, Henderson R, MacEachern C, McNamee P, Quayyum Z, et al. The clinical effectiveness of glucosamine and chondroitin supplements in slowing or arresting progression of osteoarthritis of the knee: a systematic review and economic evaluation. Health Technol Assess. 2009;13(52):1-148.
129. Zimovetz EA, Wolowacz SE, Classi PM, Birt J. Methodologies used in cost-effectiveness models for evaluating treatments in major depressive disorder: a systematic review. Cost Eff Resour Alloc. 2012;10(1):1-10.
130. Benedict A, Arellano J, De Cock E, Baird J. Economic evaluation of duloxetine versus serotonin selective reuptake inhibitors and venlafaxine XR in treating major depressive disorder in Scotland. J Affect Disord. 2010;120(1-3):94-104.
131. Jonsson L, Andreasen N, Kilander L, Soininen H, Waldemar G, Nygaard H, et al. Patient- and proxy-reported utility in Alzheimer disease using the EuroQoL. Alzheimer Dis Assoc Disord. 2006;20(1):49-55.
132. Ahern AL, Breeze P, Fusco F, Sharp SJ, Islam N, Wheeler GM, et al. Effectiveness and cost-effectiveness of referral to a commercial open group behavioural weight management programme in adults with overweight and obesity: 5-year follow-up of the WRAP randomised controlled trial. The Lancet Public Health. 2022;7(10):e866-e75.
133. Hinnouho GM, Czernichow S, Dugravot A, Nabi H, Brunner EJ, Kivimaki M, et al. Metabolically healthy obesity and the risk of cardiovascular disease and type 2 diabetes: the Whitehall II cohort study. Eur Heart J. 2015;36(9):551-9.
134. National Institute of Health and Care Excellence. NICE health technology evaluations: the manual (PMG36).
